# Genome-wide characterization and expression analysis of the *HAK* gene family in response to abiotic stresses in *Medicago*

**DOI:** 10.1186/s12864-022-09009-2

**Published:** 2022-12-01

**Authors:** Qian Li, Wenxuan Du, Xinge Tian, Wenbo Jiang, Bo Zhang, Yuxiang Wang, Yongzhen Pang

**Affiliations:** 1 Institute of Animal Science, Chinese Academy of Agricultural Sciences, 100193 Beijing, China; 2 West Arid Region Grassland Resource and Ecology Key Laboratory, College of Grassland and Environmental Sciences, Xinjiang Agricultural University, 830052 Urumqi, China; 3 Qinghai Academy of Agriculture and Forestry Sciences, Qinghai University, 810016 Xining, Qinghai, China

**Keywords:** Abiotic stress, *HAK genes*, *Medicago sativa*, *Medicago truncatula*, WGCNA

## Abstract

The high-affinity K^+^ transporter (HAK) family plays a vital role in K^+^ uptake and transport as well as in salt and drought stress responses. In the present study, we identified 22 *HAK* genes in each *Medicago truncatula* and *Medicago sativa* genome. Phylogenetic analysis suggested that these HAK proteins could be divided into four clades, and the members of the same subgroup share similar gene structure and conserved motifs. Many *cis*-acting elements related with defense and stress were found in their promoter region. In addition, gene expression profiles analyzed with genechip and transcriptome data showed that these *HAK* genes exhibited distinct expression pattern in different tissues, and in response to salt and drought treatments. Furthermore, co-expression analysis showed that 6 homologous *HAK* hub gene pairs involved in direct network interactions. RT-qPCR verified that the expression level of six *HAK* gene pairs was induced by NaCl and mannitol treatment to different extents. In particular, *MtHK2*/*7*/*12* from *M. truncatula* and *MsHAK2*/*6*/*7* from *M. sativa* were highly induced. The expression level of *MsHAK1*/*2*/*11* determined by RT-qPCR showed significantly positive correlation with transcriptome data. In conclusion, our study shows that *HAK* genes play a key role in response to various abiotic stresses in *Medicago*, and the highly inducible candidate *HAK* genes could be used for further functional studies and molecular breeding in *Medicago*.

**Supplementary Information:**

The online version contains supplementary material available at 10.1186/s12864-022-09009-2.

## Introduction

Potassium (K^+^) is one of the most essential mineral nutrients for plant growth and development, and it is also the most abundant monovalent cation in plants, accounting for approximately 2–10% of plant dry weight [1]. The role of K^+^ is not only essential for plants to maintain normal physiological and biochemical processes such as stomatal movement, photosynthesis, but also involved in responses to biotic and abiotic stresses [2–4]. The storage of K^+^ in vacuoles plays an important role in maintaining the concentration of K^+^ in cytoplasm. However, the optimal concentration of K^+^ must be maintained for the cells to function normally. In the cytoplasm, K^+^ concentration is related to the tolerance of plants to different stresses, such as drought [5] and salinity [6]. In plants, roots are the first place to sense the lack of K^+^ and has the ability to sense the change of external K^+^ concentration [7]. K^+^ is firstly absorbed by the root, then transferred to the aerial part and distributed to different organs within the cell [8]. As a fixed organism, plants have evolved an efficient K^+^ transport system to maintain the optimal growth state under lower K^+^ levels [9].

According to their structure and function, K^+^ transport proteins in plants can be divided into five families: (1) Shaker-like K^+^ channels; (2) tandem-pore K^+^ (TPK) channels; (3) HAK/KUP/KT K^+^ transporters; (4) HKT transporters; and (5) cation-proton antiporters (CPAs) [10]. Among them, the HAK/KUP/KT family is the largest families and KUP was first found in bacteria and HAK in fungi [11, 12]. In plants, based on gene homology, the HAK family members were initially identified in barley (HAK1) [13], and KUP1/KT1 and KUP2/KT2 in *Arabidopsis* [14, 15]. However, HAKs are absent in animal. Therefore, name of HAK/KUP/KT was widely used for the whole transporter family in plants [8, 10]. Subsequently, HAK/KUP/KT genes were found in other plants, such as maize [16], *Ipomoea* [17], tea [18], and *Gossypium hirsutum* [19].

HAK transporters play diverse roles in K^+^ uptake and transport, salt and drought stress responses, and morphological development of roots and shoots [10]. So far, the physiological functions of several plant K^+^ transporters have been elucidated. In *Arabidopsis*, *AtHAK5* is involved in high-affinity K^+^ uptake and it is capable of absorbing K^+^ in solutions below 10 µM [8, 9]. *AtHAK5* is required for *Arabidopsis* growth and K^+^ acquisition from low K^+^ solutions under saline conditions [9]. In rice, the expression of *OsHAK5* was up-regulated under K^+^ deficiency conditions, and over-expression of *OsHAK5* increased salt stress tolerance by K^+^/Na^+^ ratio [20]. Under normal K^+^ supply conditions, the expression of *OsHAK1* is up-regulated under salt stress. However, when K^+^ is deficient, salt stress reduced net K^+^ uptake rate of *OsHAK1* mutants, indicating that *OsHAK1* plays a key role in enhancing salt tolerance [21]. When *OsHAK1* was over-expressed in rice, it enhanced drought tolerance with lower level of lipid peroxidation and higher activities of antioxidant enzymes, and it positively regulated the expression levels of genes involved in K^+^ homeostasis and stress responses [22]. While *OsHAK21* was knockout, the ratio of K^+^ uptake and the salt tolerance decreased [23]. These findings implied *HAK*/*KUP*/*KT* genes had potential functions in promoting drought and salt tolerance in plants. In addition, it was also reported that the *HAK* gene expression level is also related to the roots, and the expression level of *AtHAK5* was induced in root under K^+^-limitation conditions [24]. It was also found that *AtKUP5* was only expressed in root hairs and could be used as a K^+^ flux sensor [25].

Although genome-wide identification of *HAK* gene family has been accomplished in rapeseed [26] and cassava [27], but not in *Medicago*. The release of the *Medicago sativa* genome data and the newly improved *Medicago truncatula* annotated reference genome assembly provided the possibility for genome-wide identification of the *HAK* gene family of these two representative species of the *Medicago* genus. The molecular basis and stress resistance mechanisms of K^+^ transport and homeostasis in *M. sativa* are largely unknown. In the present study, we identified 22 *HAK* genes from *M. truncatula* and 22 from *M. sativa* in their genomes, and analyzed their phylogenetic relationships, conserved motifs and domains, gene structure, *cis*-acting elements, syntenic relationships, tissue expression patterns and expression profiling in responses to salt stress and osmotic stress. The data provided in this study are reliable to screen key candidate genes from the HAK family in *Medicago* for further functional investigation at molecular level, and for molecular breeding of *M. sativa* with stress tolerance.

## Results

### Genome survey to identify *HAK* genes in *M. truncatula* and *M. sativa*

Based on comparative genomics, a total of 44 *HAK* candidate genes were identified from *M. truncatula* and *M. sativa* genome. Characteristics of *HAK* genes, including TIGR locus, homologous gene, protein length, number of intron, transmembrane domains (TM), isoelectric point (pI), molecular weight (MW), and putative subcellular localization, were shown in Tables 1 and 2. *MtHAK* and *MsHAK* genes encode proteins ranging from 395 to 871, 385 to 855 amino acids in length, respectively. The genomic sequences of *MtHAK* and *MsHAK* contained 3–9, 4–12 exons, respectively. All MtHAK members contain 20 TM structural domains except MtHAK8 and MtHAK16 that have 13 and 19 TM domains respectively. Members of MsHAK family contained 8–20 TM domains. Subcellular location analyses showed that HAK proteins from *M. truncatula* and *M. sativa* were all predicated to be located in plasma membrane.

**Table 1:** Properties of the predicted HAK proteins in *M. truncatula*

Name	Gene ID	Name	Homologous Gene	Length(aa)	Intron	TM	pI	MW(kDa)	Subcellular localization
*MtHAK1*	MtrunA17Chr2g0279131	MsHAK1	MsG0280006462.01.T01	849	9	20	5.58	94.7.	Plasma membrane
*MtHAK2*	MtrunA17Chr2g0297291	MsHAK2	MsG0280008140.01.T01	789	8	20	8.37	88.5.	Plasma membrane
*MtHAK3*	MtrunA17Chr2g0297311	MsHAK2	MsG0280008140.01.T01	789	7	20	8.64	88.6	Plasma membrane
*MtHAK4*	MtrunA17Chr3g0128831	MsHAK2	MsG0280008140.01.T01	794	9	20	6.8	89.3	Plasma membrane
*MtHAK5*	MtrunA17Chr4g0052401	MsHAK7	MsG0480022804.01.T01	787	8	20	8.22	87.9	Plasma membrane
*MtHAK6*	MtrunA17Chr4g0055471	MsHAK17	MsG0780041281.01.T01	815	8	20	8.66	90.9	Plasma membrane
*MtHAK7*	MtrunA17Chr5g0413721	MsHAK11	MsG0580025786.01.T01	871	8	20	8.45	97.5	Plasma membrane
*MtHAK8*	MtrunA17Chr5g0413751	MsHAK3	MsG0380016724.01.T01	395	3	13	8.69	44.6	Plasma membrane
*MtHAK9*	MtrunA17Chr5g0429991	MsHAK12	MsG0580028415.01.T01	849	9	20	5.47	95.0	Plasma membrane
*MtHAK10*	MtrunA17Chr5g0430551	MsHAK14	MsG0580029633.01.T01	725	5	20	6.18	81.5	Plasma membrane
*MtHAK11*	MtrunA17Chr5g0430731	MsHAK14	MsG0580029633.01.T01	754	8	20	6.58	84.3	Plasma membrane
*MtHAK12*	MtrunA17Chr6g0451081	MsHAK2	MsG0280008140.01.T01	776	7	20	7.58	87.1	Plasma membrane
*MtHAK13*	MtrunA17Chr6g0461871	MsHAK2	MsG0280008140.01.T01	785	8	20	8.74	87.9	Plasma membrane
*MtHAK14*	MtrunA17Chr7g0268981	MsHAK17	MsG0780041281.01.T01	773	7	20	7.57	87.2	Plasma membrane
*MtHAK15*	MtrunA17Chr8g0344551	MsHAK22	MsG0880045041.01.T01	766	8	20	7.16	85.5	Plasma membrane
*MtHAK16*	MtrunA17Chr8g0344581	MsHAK22	MsG0880045041.01.T01	634	9	19	8.79	70.7	Plasma membrane
*MtHAK17*	MtrunA17Chr8g0365001	MsHAK12	MsG0580028415.01.T01	840	8	20	6.11	93.1	Plasma membrane
*MtHAK18*	MtrunA17Chr8g0365041	MsHAK22	MsG0880045041.01.T01	745	7	20	8.5	83.5	Plasma membrane
*MtHAK19*	MtrunA17Chr8g0375411	MsHAK4	MsG0480022271.01.T01	698	7	20	8.09	77.9	Plasma membrane
*MtHAK20*	MtrunA17Chr8g0380281	MsHAK17	MsG0780041281.01.T01	782	8	20	9	87.2	Plasma membrane
*MtHAK21*	MtrunA17Chr8g0387661	MsHAK2	MsG0280008140.01.T01	792	8	20	9.26	88.6	Plasma membrane
*MtHAK22*	MtrunA17Chr8g0393241	MsHAK14	MsG0580029633.01.T01	799	9	20	8.62	88.7	Plasma membrane

**Table 2:** Properties of the predicted HAK proteins in *M. sativa*

Name	Gene ID	Name	Homologous Gene	Length(aa)	Intron	TM	pI	MW(kDa)	Subcellular localization
*MsHAK1*	MsG0280006462.01.T01	MtHAK1	MtrunA17Chr2g0279131	855	12	20	5.11	94.7	Plasma membrane
*MsHAK2*	MsG0280008140.01.T01	MtHAK2	MtrunA17Chr2g0297291	789	8	20	8.22	88.4	Plasma membrane
*MsHAK3*	MsG0380016724.01.T01	MtHAK4	MtrunA17Chr3g0128831	576	6	14	5.85	64.9	Plasma membrane
*MsHAK4*	MsG0480022271.01.T01	MtHAK19	MtrunA17Chr8g0375411	700	7	20	8.67	78.2	Plasma membrane
*MsHAK5*	MsG0480022272.01.T01	MtHAK19	MtrunA17Chr8g0375411	685	7	20	8.78	76.8	Plasma membrane
*MsHAK6*	MsG0480022802.01.T01	MtHAK5	MtrunA17Chr4g0052401	413	2	11	8.25	47.1	Plasma membrane
*MsHAK7*	MsG0480022804.01.T01	MtHAK5	MtrunA17Chr4g0052401	788	7	20	8.06	88.0	Plasma membrane
*MsHAK8*	MsG0480022868.01.T01	MtHAK19	MtrunA17Chr8g0375411	452	7	13	9.15	51.3	Plasma membrane
*MsHAK9*	MsG0480022869.01.T01	MtHAK19	MtrunA17Chr8g0375411	685	7	20	8.78	76.8	Plasma membrane
*MsHAK10*	MsG0480023156.01.T01	MtHAK16	MtrunA17Chr8g0344581	536	9	8	6.46	59.5	Plasma membrane
*MsHAK11*	MsG0580025786.01.T01	MtHAK7	MtrunA17Chr5g0413721	532	7	17	8.47	58.9	Plasma membrane
*MsHAK12*	MsG0580028415.01.T01	MtHAK9	MtrunA17Chr5g0429991	849	9	20	5.47	94.9	Plasma membrane
*MsHAK13*	MsG0580028458.01.T01	MtHAK11	MtrunA17Chr5g0430731	767	7	16	7.75	85.9	Plasma membrane
*MsHAK14*	MsG0580029633.01.T01	MtHAK11	MtrunA17Chr5g0430731	687	6	16	6.42	76.8	Plasma membrane
*MsHAK15*	MsG0680031788.01.T01	MtHAK13	MtrunA17Chr6g0461871	510	9	13	8.37	57.1	Plasma membrane
*MsHAK16*	MsG0680031789.01.T01	MtHAK13	MtrunA17Chr6g0461871	535	9	14	7.26	60.3	Plasma membrane
*MsHAK17*	MsG0780041281.01.T01	MtHAK14	MtrunA17Chr7g0268981	722	8	20	6.77	80.9	Plasma membrane
*MsHAK18*	MsG0880042781.01.T01	MtHAK15	MtrunA17Chr8g0344551	391	4	14	5.39	44.4	Plasma membrane
*MsHAK19*	MsG0880042786.01.T01	MtHAK15	MtrunA17Chr8g0344551	431	8	13	6.17	48.7	Plasma membrane
*MsHAK20*	MsG0880042787.01.T01	MtHAK15	MtrunA17Chr8g0344551	385	4	16	7.54	42.7	Plasma membrane
*MsHAK21*	MsG0880042817.01.T01	MtHAK15	MtrunA17Chr8g0344551	535	5	16	6.65	60.3	Plasma membrane
*MsHAK22*	MsG0880045041.01.T01	MtHAK18	MtrunA17Chr8g0365041	745	7	20	8.36	83.5	Plasma membrane

### Multiple sequence alignment, phylogenetic analysis and classification of *HAK* genes in *Medicago*

In order to better understand the characteristics of HAK protein sequence, the most conservative region covering potassium ion transporter HAK were analyzed using MEGA-X, and displayed via jalview (Additional Fig. S2). The conserved amino acid sequences of six KUP/HAK/KT domains are GDLGTSPLY, ANDDNGEGG, GDGVLTPAIS, GSEAMFADLGHF, AYGIAVV, and FRCIVIYGYKD, respectively. All HAK members contain at least two KUP/HAK/KT domains, which were similar to those *HAKs* members from *Gossypium raimondii* [28] and *Cajanus cajan* [29].

**Fig. 1:**
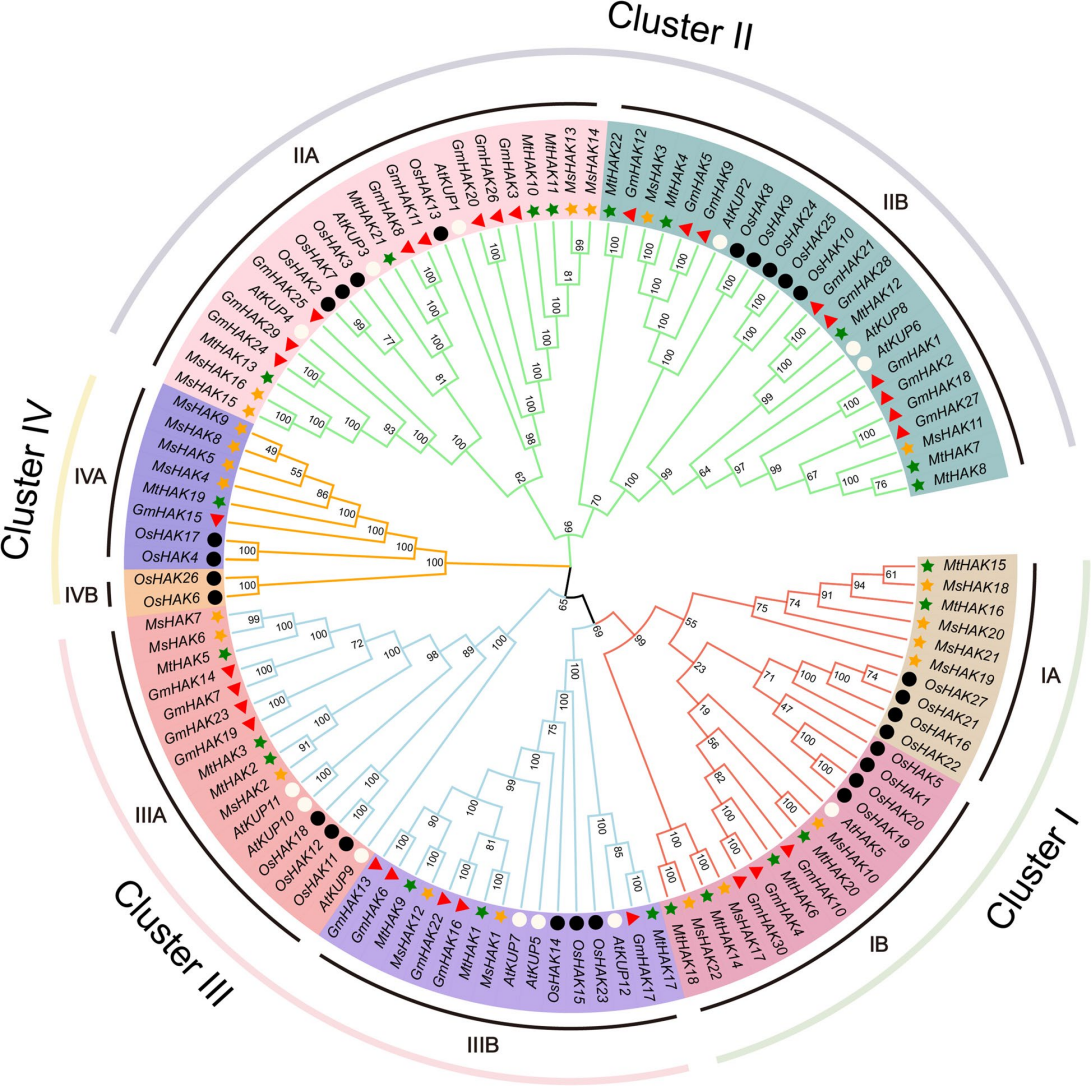
Phylogenetic analysis of HAK families across *Medicago*, *Arabidopsis*, *Glycine max* and *Oryza sativa*. Full-length protein sequences of HAKs were constructed using MEGA-X based on the Neighbor-Joining (NJ) method; bootstrap was 1,000 replicates. Four clusters (I, II, III, IV) are subdivided into two sub-clusters A and B. The green solid pentagrams, orange solid pentagrams, hollow circles, red triangle and black square represent HAK proteins from *M. truncatula* (Mt), *M. sativa* (Ms), *A. thaliana* (At), *G. max* (Gm) and *O. sativa* (Os); Phylogenetic trees were designed using MEGA7.0 according to the maximum likelihood method and performed bootstrap testing with 1000 replicates

To analyze the phylogenetic relationship and evolution of the HAK family in *Medicago* and different plants, we used 113 *HAK* genes from several plants to construct a phylogenetic tree by using neighbor-joining (NJ) method, including *A. thaliana*, *M. truncatula*, *M. sativa*, *Oryza sativa*, and *Glycine max* (Fig. 1). These HAK proteins could be divided into four clades as in a previous report [30], and each of them could be further subdivided into sub-cluster A and B. Cluster I consisted of six *MtHAKs*, seven *MsHAKs* and only one *AtHAK* (*AtHAK5*), Cluster II contained the most HAKs members (47) with 15 members from *Medicago*. Cluster III has two subclusters (IIIA and IIIB) with the same number (Fig. 1), and three *AtKUP* members within each subcluster. Cluster IV has the least HAK with only 10 members. Notably, subcluster IVB has only two HAK members from rice (*OsHAK26* and *OsHAK6*). This indicated that the amplification rate in distinct groups varies, which might reflect the specific function during the process of evolution.

### Analysis of gene structure and conserved motifs of *HAK* genes in Medicago

**Fig. 2:**
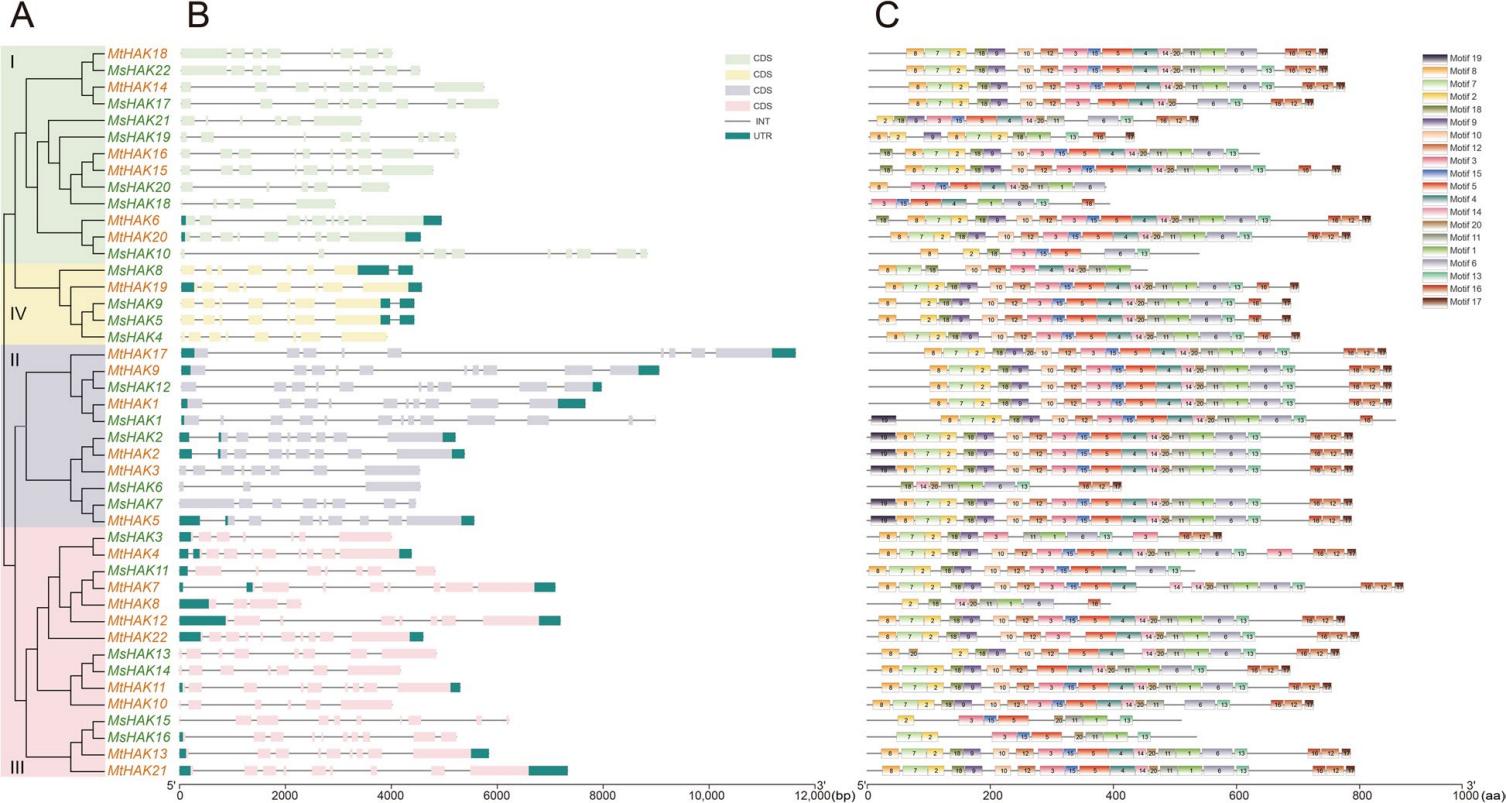
Phylogenetic relationships, gene structure and motifs of *HAK* genes from *M. truncatula* and *M. sativa* (**A**, **B**, and **C**) using TBtools. The groups and their color in phylogenetic tree were the same as in Fig. 1. Black boxes indicate 5′- and 3′- untranslated regions; boxes with the same color as in panel A indicate exons; black lines indicate introns. The motifs were indicated in different colored boxes with different numbers and the sequence information for each motif was provided in Additional Fig. S1

To comprehensively study the function of *HAK* genes, we performed analysis on gene structure and conserved motifs (Fig. 2). Over long evolutionary time intervals, exon and intron positions are generally conserved in orthologous genes, whereas intron/exon structure of HAK family varied, but sufficiently conserved in paralogous genes [31, 32]. The *HAK* gene structure analyzed in *Medicago* revealed the intron/exon organization and conservation among them. Cluster I contains 5–10 exons and none of them contain UTRs except *MtHAK6* and *MtHAK20* (Fig. 2A, B). In cluster II, great diversity was observed in exon length. Cluster III and cluster IV contain 4–10 and 7–8 exons, respectively, and the gene structure and the number of the cluster IV is highly consistent (Fig. 2A, B).

We identified 20 conserved motifs with sizes ranging from 15 to 50 residues in width, which were annotated as K^+^ potassium transporter motif (Additional Fig. S3). Generally, the motifs were almost evenly distributed, and a similar number of motifs were present in HAK proteins from each of the four clusters (Fig. 2 A, C). Motifs 1–10, 12 and 16–18 were conserved in all four HAK clusters, with a few exceptions where a particular motif was missing in 3–5 genes (Fig. 2C). Together, the common K^+^ potassium transporter motifs and similar gene structure in the same cluster supported the phylogenetic classification of the HAK family and implied functional similarities among these *HAK* genes.

### Analyses of chromosomal distribution and synteny of *HAK* genes in *Medicago*

**Fig. 3:**
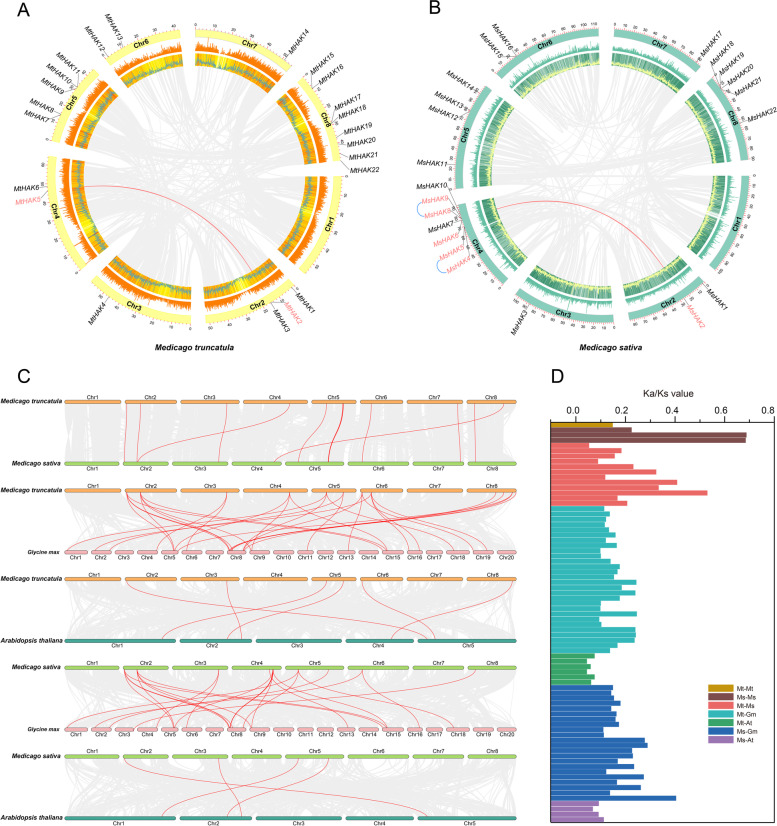
Chromosome distributions of HAKs in *M. truncatula* and *M. sativa* using TBtools. The chromosomal location and interchromosomal relationship of *M. truncatula* (**A**) and *M. sativa* (**B**). The segmentally duplicated and tandem duplicated genes are connected by red curves. **C**. Synteny analysis of *HAK* genes between *M. truncatula* and *M. sativa*, *M. truncatula* and *G. max*, *M. truncatula* and *A. thaliana*, *M. sativa* and *G. max*, *M. sativa* and *A. thaliana*. Gray lines in the background indicate the collinear blocks, and the red lines highlight the syntenic *HAK* gene pairs. **D**. The Ka/Ks values of *HAK* gene pairs for *Mt-Mt*, *Ms-Ms*, *Mt-Ms*, *Mt-Gm*, *Mt-At*, *Ms-Gm* and *Ms-At.*

The genomic distribution of the *HAK* genes in *Medicago* was determined by mapping the ORFs of all identified genes onto their corresponding chromosome (Fig. 3). It was shown that the distribution of *HAK* genes were uneven in both *M. truncatula* and *M. sativa*, and they were distributed on chromosomes 2–7, but none on chromosome 1. In *M. truncatula* and *M. sativa*, 1, 2, 1 *HAK* members were found on chromosomes 3, 6, and 7, respectively, and these members were in approximately the same chromosomal position in both species (Fig. 3). Differently, most *MtHAK* members were found on chromosome 8 with eight members, followed by chromosome 5 with five members (Fig. 3A). Most *MsHAK* members were found on chromosome 4 with six members, followed by chromosome 8 with five members (Fig. 3B).

Tandem, segmental and whole-genome duplication are the main impetus for gene family expansion [33]. Two pairs of segmental duplication were found in *M. truncatula* (*MtHAK2*/*MtHAK5*) and *M. sativa* (*MsHAK2*/*MsHAK6*), respectively (Fig. 3A, B and Table S2). In addition, only two pair of tandem repeat events (*MsHAK4*/*MsHAK5* and *MsHAK8*/*MsHAK9*) were found in *M. sativa* (Fig. 3B and Table S2), while this event was absent in *M. truncatula* (Fig. 3A).

Furthermore, comparative syntenic maps of *M. sativa*, *G. max* and *A. thaliana* associated with *M. truncatula*, and *G. max* and *A. thaliana* associated with *M. sativa* were constructed to illustrate the evolution relationship of *HAK* gene family (Fig. 3C). Notably, 10, 28 and 6 orthologous pairs were found between *M. truncatula* and *M. sativa*, *M. truncatula* and *G. max*, *M. truncatula* and *A. thaliana*, respectively (Fig. 3C and Table S2). Three genes in *M. truncatula* (*MtHAK1*, *4* and *7*) showed a collinear relationship with those in *M. sativa*, *G. max* and *A. thaliana* (Fig. 3C and Table S2). Meanwhile, 22 and 4 orthologous pairs were found between *M. sativa* and *G. max*, *M. sativa* and *A. thaliana*, respectively (Fig. 3C and Table S2), and four genes in *M. sativa* (*MsHAK1*, *3*, *11* and *7*) showed a collinear relationship with those in *A. thaliana* and *G. max* (Fig. 3C and Table S2). These genes may play irreplaceable role in evolution of the *HAK* family.

To better understand the evolutionary selection pressure during the formation of *HAK* gene family, the Ka/Ks values of *HAK* gene pairs were analyzed for *Mt-Mt*, *Ms-Ms*, *Mt-Ms*, *Mt-Gm*, *Mt-At*, *Ms-Gm* and *Ms-At* (Fig. 3D and Table S2). The Ka/Ks values of these orthologous gene pairs were all less than 1, indicating that *HAK* genes may have undergone strong purification selection pressure during evolution.

### Analysis of *cis*-acting elements in the promoter sequences of *HAK* genes in *Medicago*

**Fig. 4:**
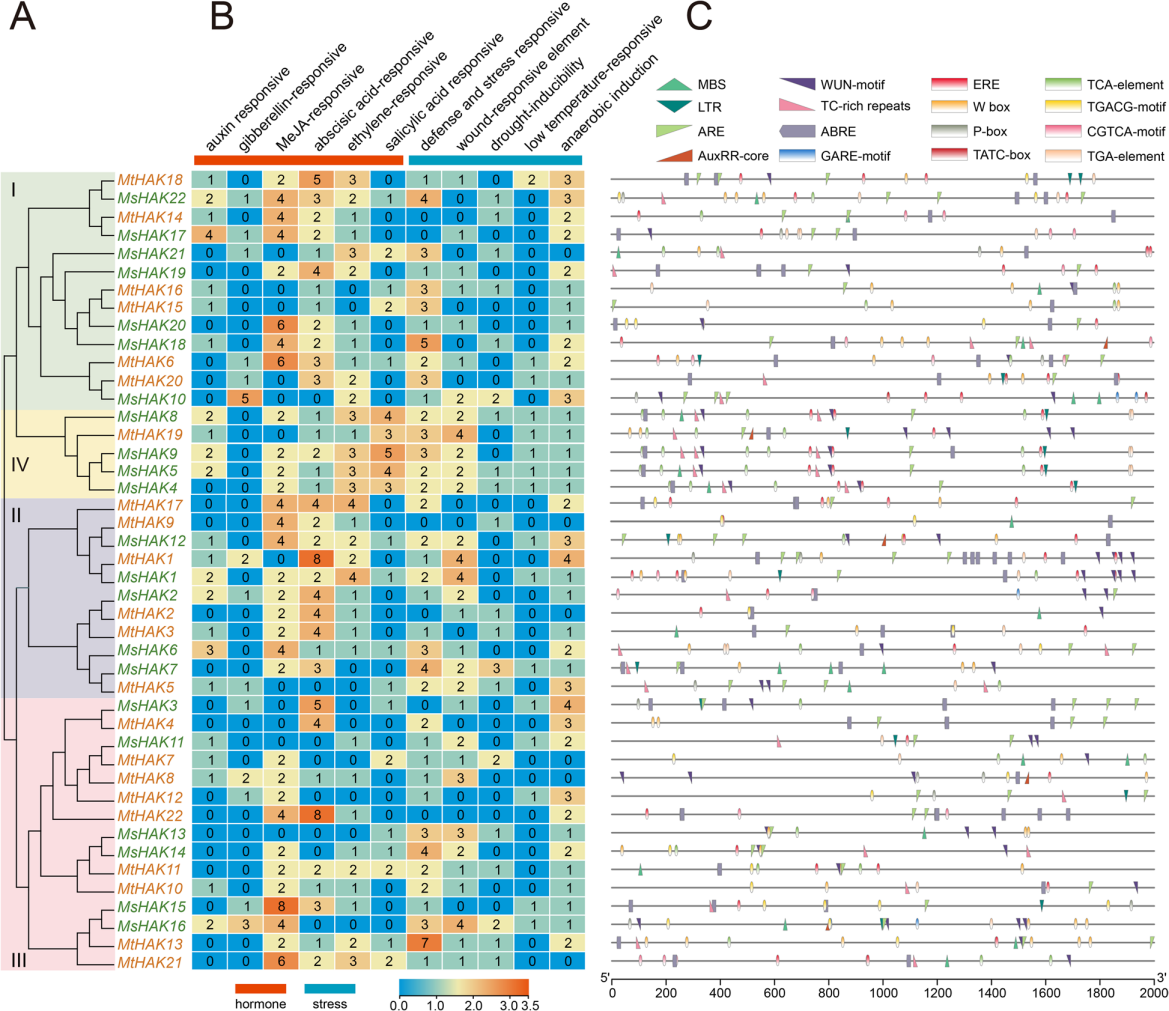
Putative *cis*-acting elements and transcription factor binding sites in the promoter regions of *HAK* genes from *M. truncatula* and *M. sativa* using TBtools. **A**. The groups and color are indicated as in Fig. 1**B**. The color and number of the grid indicated numbers of different *cis*-acting elements in these *HAK* genes. **C**. The colored block represented different types of *cis*-acting elements and their locations in each *HAK* gene

*Cis*-acting elements serve as potential regulators of abiotic stress. The online tool PlantCARE was utilized to identify several *cis*-acting elements in *HAK* genes. The promoter sequence of 2,000 bp for the 22 *MtHAK* and 22 *MsHAK* genes were analyzed. The *cis*-acting elements identified were functionally categorized into 11 categories, including: auxin responsive (AuxRE-core), gibberellin-responsive (GARE-motif, P-box, TATC-box), MeJA-responsive (TGACG-motif, CGTCA-motif), abscisic acid-responsive (ABRE), defense and stress responsiveness (TC-rich repeats, W-box), MYB binding site involved in drought-inducibility (MBS), ethylene-responsive (ERE), salicylic acid responsiveness (TCA-element), wound responses (WUN motif), low temperature-responsive (LTR), and anaerobic induction (ARE) (Fig. 4 and Table S3).

The promoters of *HAK* genes contained various *cis*-acting elements with different numbers. In particular, most *HAK* genes contain ARE elements and they may play a crucial role in anaerobic induction response in roots of *Medicago*. Methyl jasmonate (MeJA), as a wounding-related phytohormone, it is able to stimulate the expression of defense-related genes [34]. Interestingly, *HAK* genes have relatively more MeJA-responsive elements than the other types (Fig. 4B), in particular *MsHAK15* with 8 MeJA-responsive elements, indicating that *HAK* with more MeJA-responsive elements maybe play a specific role in wounding stress resistance induced by MeJA. It is generally known that three *cis*-acting elements, ABRE, MBS and W-box, are related with responsiveness to drought-induced signaling and regulation of downstream gene expression [35]. Our results showed that many *HAK* genes contained more ABRE were grouped in cluster I, II, IV (Fig. 4B, C), indicating that *HAK* gene family plays a role in drought resistance in *Medicago*. Notably, salicylic acid responsiveness elements were presented with high numbers in cluster IV, indicating that these five genes of this cluster play a key role in resistance to salicylic acid-related stress (Fig. 4A, B).

### Expression patterns of *HAK* genes in different tissues and under stress treatments

**Fig. 5:**
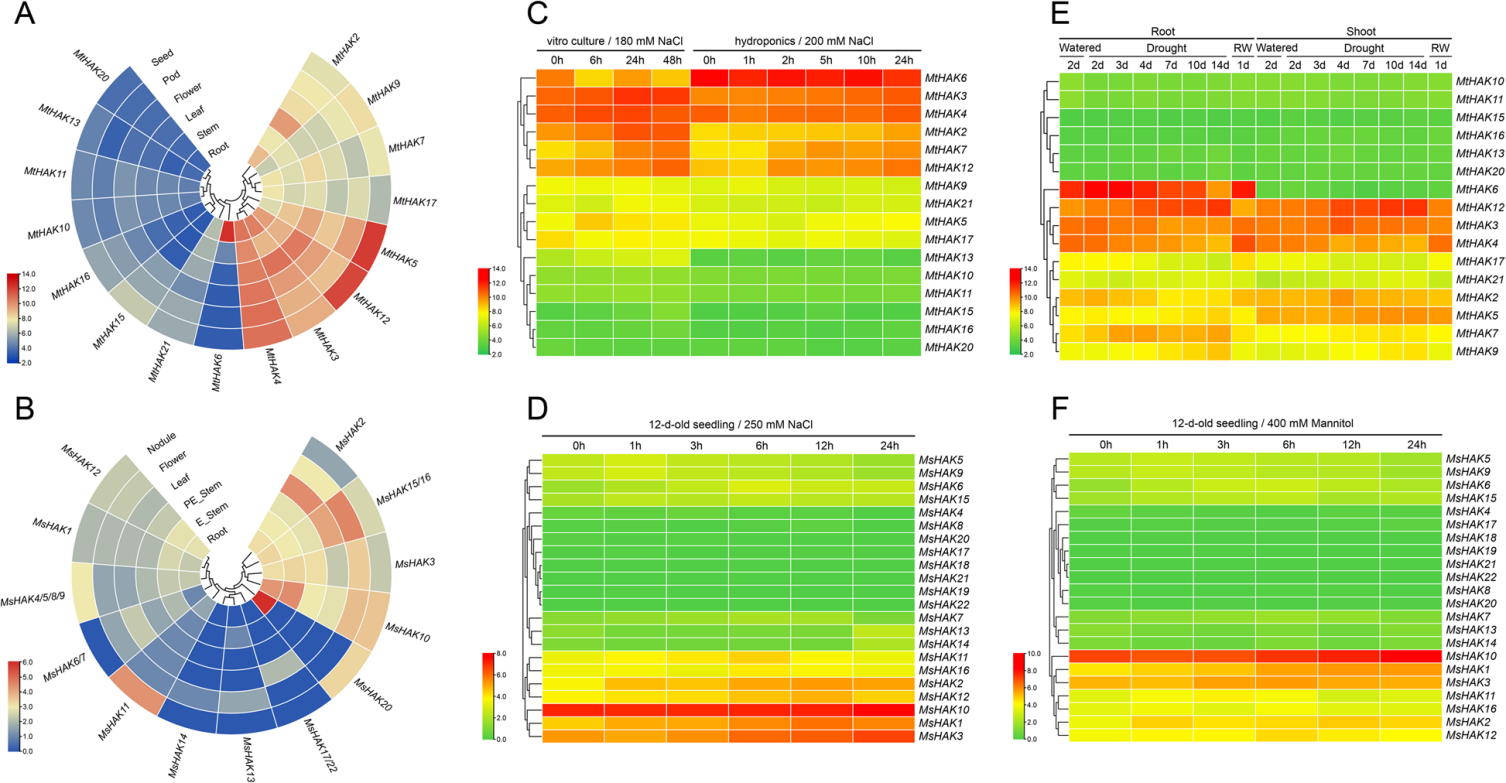
Expression profiles of *HAK* genes from *Medicago* using TBtools. **A**, **B**: Expression profiles of *MtHAK* and *MsHAK* genes in different tissues retrieved from genechip dataset and transcriptome, respectively. **C**: expression level of *MtHAK* genes under NaCl treatment (in vitro culture and hydroponics culture) at different treatment time. **D**: Expression level of *MsHAK* genes under NaCl treatment (12-d-old seeding) at different treatment time. **E**: Expression level of *MtHAK* genes under drought treatment in roots and in shoots at different treatment time. F: Expression level of *MsHAK* genes under mannitol treatment (12-d-old seeding) at different treatment time. The relative expression levels are log_2_-transformed and visualized for heatmap. Red represents relatively high expression and blue (**A** and **B**) and green (**C**, **D**, **E** and **F**) represents relatively low expression

We investigated the expression patterns of *HAKs* in various tissues of *M. truncatula* with genechip dataset from the MtGEA web server, including roots, stems, leaves, flowers, pods and seeds (Fig. 5A, Table S4). Overall, half (8) of the 16 *MtHAK* genes with available probe sets were expressed at relatively low level in these tissues, and the other half were expressed at relatively high level (Fig. 5A). Among the eight genes with low expression level, *MtHAK15* was highly expressed in seeds (Fig. 5A). Among the other eight genes with high expression level, *MtHAK17* were expressed at relatively low level in seeds and flowers, and *MtHAK7* in pods (Fig. 5A). As for *M. sativa*, gene expression levels in six tissues were analyzed based on transcriptome data, including roots, elonged-stems, pre-elonged-stems, leaves, flowers and nodules (Fig. 5B, Table S4). According to their expression level, these 19 *MsHAK* genes could be divided into three categories, genes with high expression level in each tissue (*MsHAK2*,*15*/*16*,*3*,*10*), genes with low expression level (*MsHAK20*, *17*/*22*, *13*, *14*, *11*), and genes with moderate expression level (*MsHAK6*/*7*, *4*/*5*/*8*/*9*, *1*, *12*) (Fig. 5B). Notably, among genes with high expression level, *MsHAK2* was expressed with relatively low level in nodules. Among the genes with low expression level, *MsHAK20* was expressed at relatively high level in roots and nodules, and *MsHAK11* in nodules. Among genes with moderate expression level, *MsHAK6/7* were expressed with relatively low in nodules (Fig. 5B).

Expression profiles of *MtHAK* genes under stress were initially analyzed based on the data retrieved from the MtGEA web server, including samples from roots and shoots under drought treatment, and roots under in vitro culture salinity and under hydroponic salinity conditions (Fig. 5C, E, Table S4). The expression level of *MtHAK3*, *MtHAK2*, *MtHAK7* and *MtHAK12* were highly induced in both in vitro culture and hydroponic salinity conditions (Fig. 5C). In addition, the expression level of several genes were significantly increased under drought treatment, but decreased after re-watering, including *MtHAK12*, *MtHAK7* and *MtHAK5* in roots and *MtHAK12*, *MtHAK3* and *MtHAK2* in shoots (Fig. 5E).

As for *M. sativa*, the expression level of *MsHAK* genes were analyzed under NaCl and mannitol treatment with transcriptome data, and it was found that most genes were induced at different level (Fig. 5D, F, Table S4). Under both treatments, *MsHAK1*, *MsHAK3*, and *MsHAK10* maintained a relatively high level than all the other genes (Fig. 5D, F). The expression level of *MsHAK1*, *MsHAK2* and *MsHAK3* were also significantly increased under both treatments (Fig. 5D, F).

### Co-expression network analysis in *M. truncatula* under stress treatment

**Fig. 6:**
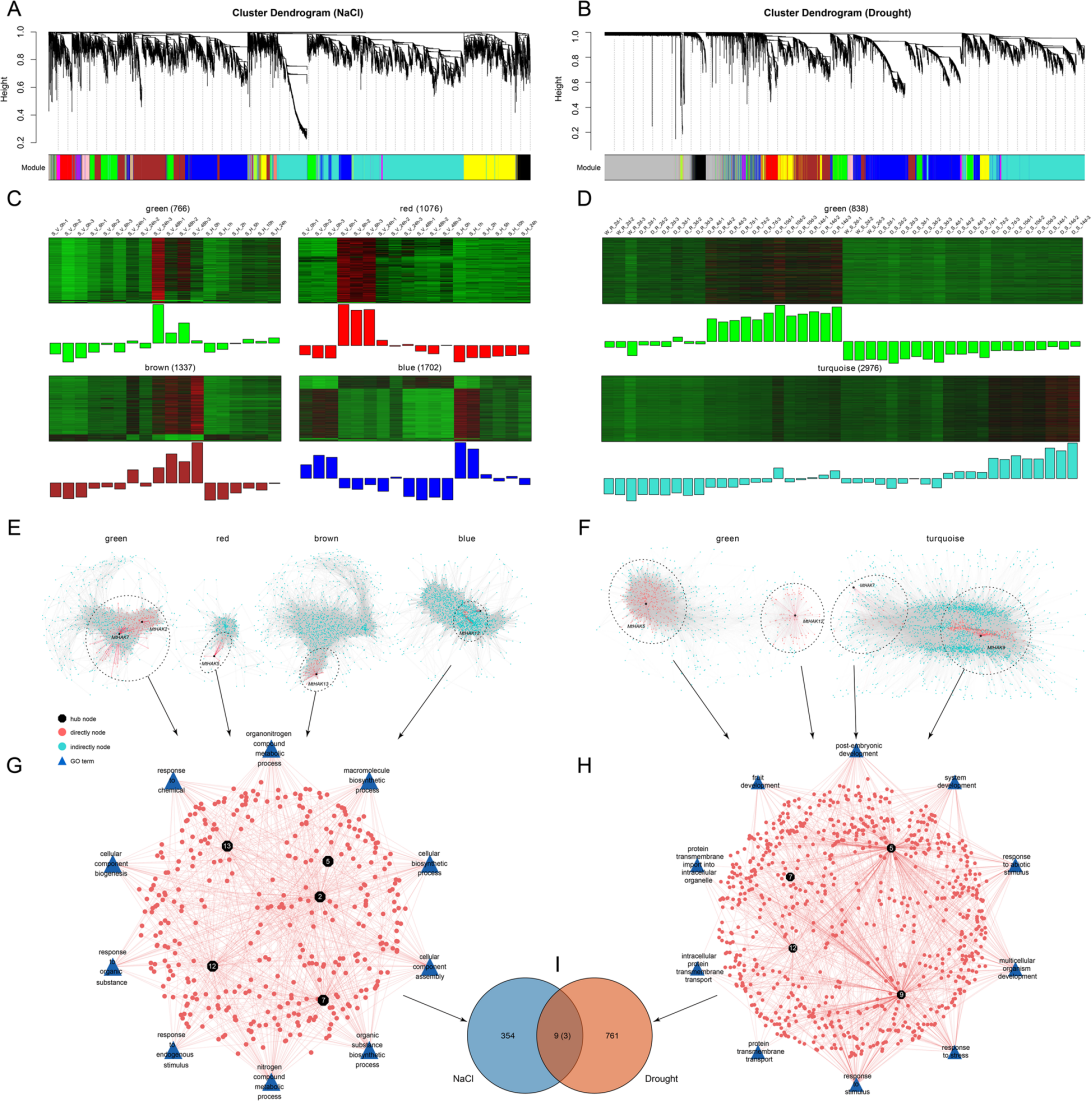
The co-expression network of *MtHAK* genes in DEGs under salt and drought stresses, and GO enrichment analysis. **A**, **B**: Hierarchical cluster tree showing co-expression modules identified by WGCNA in *M. truncatula* under salt and drought stresses, respectively. The color rows below dendrograms indicate different module memberships. **C**, **D**: Heatmaps showing the expression profile of all the co-expressed genes in specific modules. Each row in the heatmap corresponds to an individual gene. Bar graphs (below the heatmaps) indicate the consensus expression pattern of the co-expressed genes within each module. **E**: Co-expression network in green, red, brown and blue module, respectively. The black dots indicate *HAK* hub genes; the red dots indicate genes directly related to *MtHAK* genes, and blue dots indicate genes that are not directly related to *MtHAK* genes; **F**: Co-expression network in green and turquoise module, respectively. **G**, **H**: GO enrichment analysis on *HAK* genes directly related with network under salt or drought stress in *M. truncatula* using Cytoscape. The blue triangle represents the GO term. **I**: VEEN map of directly related *HAK* genes screened under salt and drought stress in *M. truncatula*

To further investigate the function of the *MtHAK* gene family under NaCl stress in *M. truncatula*, a weighted gene co-expression network analysis (WGCNA) was performed with 10,658 genes that were differentially expressed, and a total of 13 modules were generated (Fig. 6A). It is worth noting that three up-regulated modules were screened and shown in green, red, and brown, and one down-regulated module was found and shown in blue (Fig. 6A). *MtHAK7* and *MtHAK2* were recognized in the green modules, *MtHAK5* in red modules, *MtHAK13* in brown modules, and *MtHAK12* in blue modules (Fig. 6E). Green module contained 766 genes and 22,362 interactions, with 92 and 123 genes are closely related with *MtHAK7* and *MtHAK2*, respectively. Red module contains 1076 genes and 32,555 interactions, 12 genes are closely related with *MtHAK5*. Brown module contained 1337 genes and 41,798 interactions, and 58 genes are closely related with *MtHAK13*. The blue module contains 1702 genes and 219,316 interactions, with 83 genes are closely related with *MtHAK12* (Fig. 6C, E). We found that in the co-expression network with 5 *MtHAK* genes, the main GO enrichment pathways included macromolecule biosynthetic process, response to endogenous stimulus, cellular component assembly and nitrogen compound metabolic process (Fig. 6G). The above results suggest that these five *MtHAK* genes may respond to salt stress by participating in these metabolic pathways.

In order to explore the response mechanism of *M. truncatula* to drought stress, the co-expression network analysis was conducted with 15,175 differentially expressed genes, resulting in a total of 12 modules (Fig. 6B). Among them, two up-regulated modules were screened and shown in green and turquoise, and they included 838 and 2,976 genes, and 47,581 and 307,210 interactions, respectively (Fig. 6D). *MtHAK5* and *MtHAK12* were identified in the green module, of which 342 and 83 genes were closely associated with *MtHAK5* and *MtHAK12*, and *MtHAK7* and *MtHAK9* were identified in the turquoise module, with 16 and 324 genes were directly related with *MtHAK7* and *MtHAK9*, respectively (Fig. 6F). The GO enrichment analysis of the co-expression network of the four identified *HAKs* revealed that they were mainly enriched in several pathways such as response to abiotic stimulus, response to stress and response to stimulus, indicating that these *HAK* genes are likely involved in these pathways (Fig. 6H). Taken together, among 363 and 770 genes that were screened under salt stress or drought stress, 9 genes were co-expressed in both treatments, including 3 *HAK* genes (*MtHAK5*, *MtHAK7*, and *MtHAK12*) (Fig. 6I, Table S5).

### Co-expression network analysis in *M. sativa* under stress treatment

**Fig. 7:**
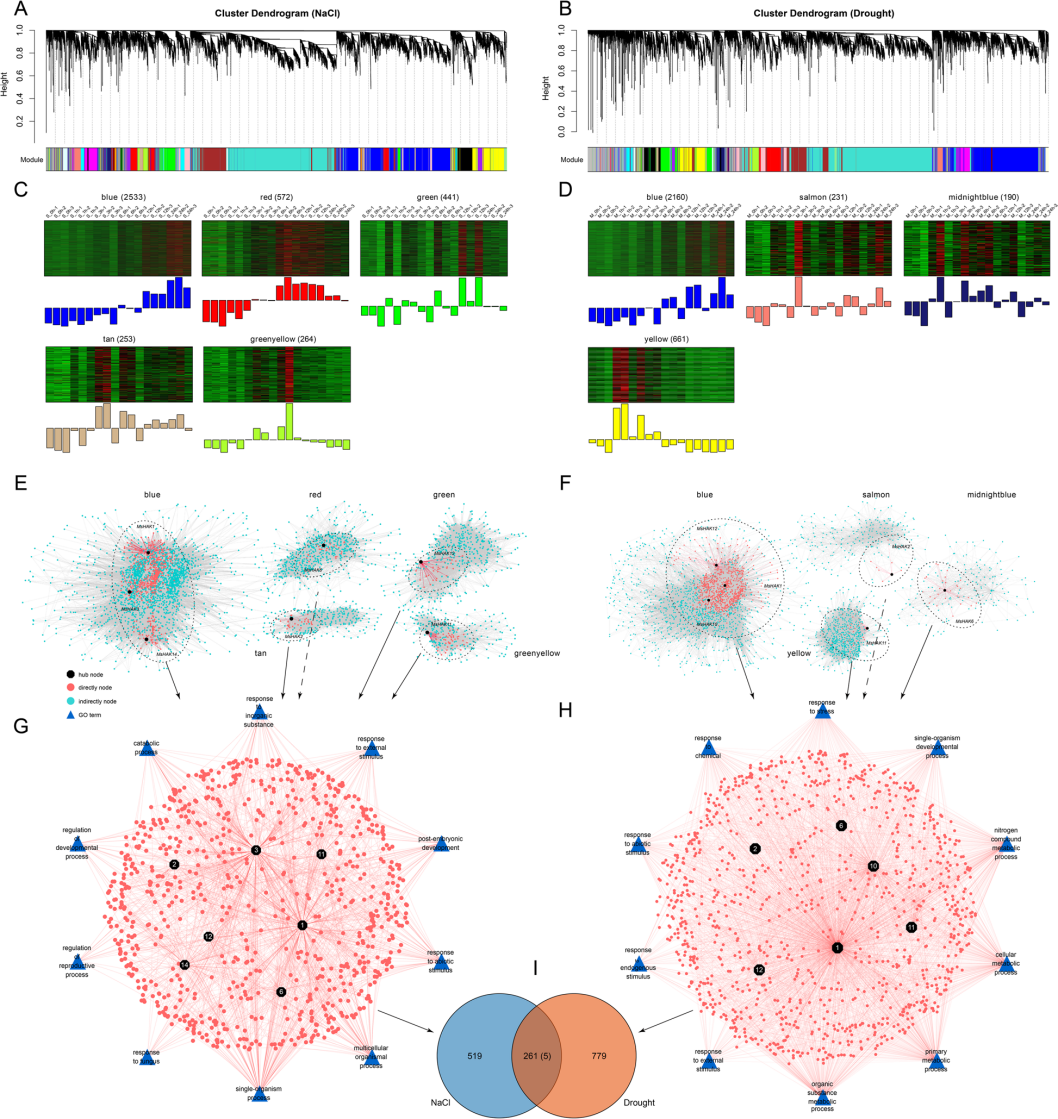
The co-expression network of *MsHAK* genes in differentially expressed genes under salt and drought stresses, and GO enrichment analysis. **A**, **B**: Hierarchical cluster tree showing co-expression modules identified by WGCNA in *M. sativa* under salt and drought stresses, respectively. The color rows below the dendrograms indicate different module memberships. **C**, **D**: Heatmaps showing the expression profile of all the co-expressed genes in specific modules. Each row in the heatmap corresponds to an individual gene. Bar graphs (below the heatmaps) indicate the consensus expression pattern of the co-expressed genes within each module. **E**: Co-expression network in blue, red and green module, respectively. The black dots indicate *HAK* hub genes; the red dots indicate genes directly related with *MsHAK* genes, and blue dots indicate genes that are not directly related with *MsHAK* genes; **F**: Co-expression network in blue, salmon and midnightblue module, respectively. **G**, **H**: GO enrichment analysis on *MsHAK* genes directly related with network using Cytoscape. The blue triangle represents the GO term. I: VEEN map of directly related *MsHAK* genes screened under salt and drought stress in *M. sativa*

In order to study the response mechanisms of *M. sativa* under salt and drought stress, co-expression network analysis was performed on 14,144 and 12,677 differentially expressed genes in *M. sativa*, respectively (Fig. 7). Under salt stress, a total of 27 expression modules were generated (Fig. 7A), and *HAK* genes were identified in 5 modules (Fig. 7C). A total of 2,533 genes and 334,585 interactions in the blue module, and a total of 572 genes and 20,049 interactions in the red module, 441 genes and 13,866 interactions in green module, 253 genes and 5,056 interactions in tan module, and 264 genes and 5,807 interactions in greenyellow module (Fig. 7C, E). *MsHAK1*/*3*/*14* were identified in the blue module, with 278/319/98 genes were closely related to *MsHAK1*/*3*/*14*, *MsHAK6*/*12*/*2*/*11* were identified in red/green/tan/greenyellow, with 31/28/30/48 genes closely related with them, respectively (Fig. 7E). The GO enrichment analysis showed that *M. sativ*a mainly responded to salt stress through catabolic process, response to external stimulus, response to abiotic stimulus, regulation of developmental process and regulation of reductive process (Fig. 7G).

A total of 23 modules were generated under drought stress in *M. sativa* (Fig. 7B). Finally, four modules of blue, salmon, midnightblue and yellow were identified to contain *HAK* genes. The blue module included 2,160 genes and 173,756 interactions. *MsHAK1*/*10*/*12* were found in this module, which were closely related to 860/282/76 genes, respectively. *MsHAK2* was identified in the salmon module, which was closely related with 7 genes; *MsHAK6* was identified in the midnightblue module, which was closely related with 28 genes. *MsHAK11* was identified in the yellow module and it was closely related with 37 genes (Fig. 7F). GO enrichment analysis mainly enriched metabolic processes such as metabolic process, developmental process, response to abiotic stimulus and response to stress (Fig. 7H), indicating *M. sativa* may respond to drought through these pathways. Of the 780 and 1,040 genes screened under salt or drought stress in *M. sativa*, 261 genes were induced by these two stresses simultaneously, in which 5 *HAK* genes (*MsHAK1*, *MsHAK2*, *MsHAK6*, *MsHAK11*, *MsHAK12*) were co-expressed in both treatments (Fig. 7I, Table S5).

### Validation of the expression profile of stress-responsive *HAK* genes by RT-qPCR

By co-expression analyses, we found that three genes *HAK* genes (*MtHAK5*, *MtHAK7*, and *MtHAK12)* in *M. truncatula* were induced by both salt and drought stress, and their homologous genes in *M. sativa* were *MsHAK2*, *MsHAK7*, and *MsHAK11* (Fig. 6I). Meanwhile, five genes were screened in the co-expression network from *M. sativa* (*MsHAK1*, *MsHAK2*, *MsHAK6*, *MsHAK11*, and *MsHAK12)*, and their homologous genes in *M. truncatula* are *MtHAK1*, *MtHAK2*, *MtHAK5*, *MtHAK7*, and *MtHAK9* (Fig. 7I). In these 16 *HAK* genes, 4 of them are duplicate genes (*MtHAK5*, *MtHAK7*, *MsHAK2* and *MsHAK11*), and 6 genes from *M. truncatula* (*MtHAK1*, *MsHAK2*, *MtHAK5*, *MtHAK7*, *MtHAK9*, and *MtHAK12*), and 6 genes from *M. sativa* (*MsHAK1*, *MsHAK2*, *MsHAK6*, *MsHAK7*, *MsHAK11*, and *MsHAK12)* were selected for further analyses.

**Fig. 8:**
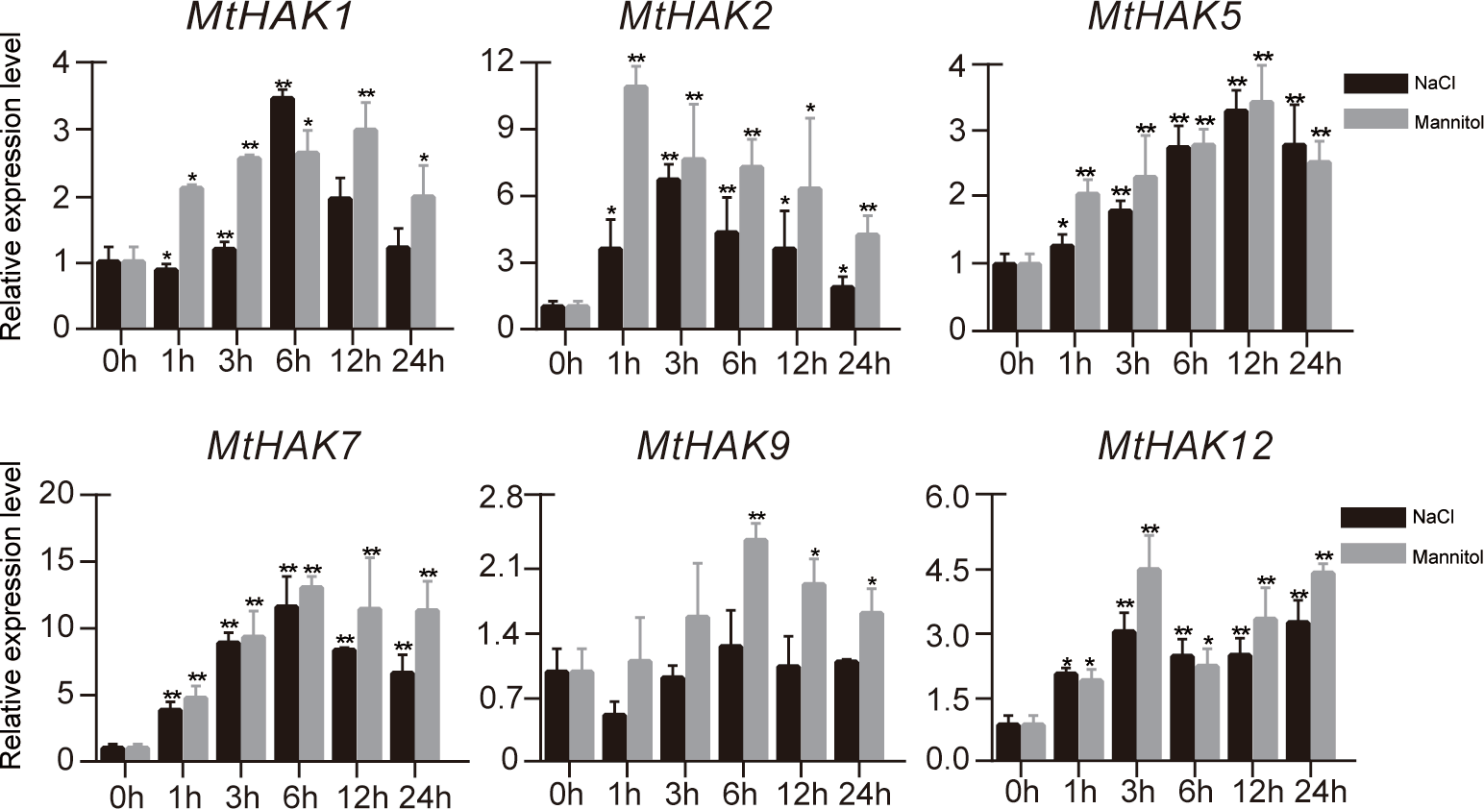
Quantification of gene expression levels of selected *HAK* genes from *M. truncatula* using RT-qPCR. Expression level of *MtHAK* genes under NaCl and mannitol stresses. *Actin* gene was used as house-keeping gene in RT-qPCR. Expression level of each gene at 0 h for each treatment was set as value of 1 and their expression levels at each time point were relative to 0 h. Data are average of three independent biological samples ± SE and vertical bars indicate standard deviation. ** indicates *P* < 0.01, and * indicates *P* < 0.05

**Fig. 9:**
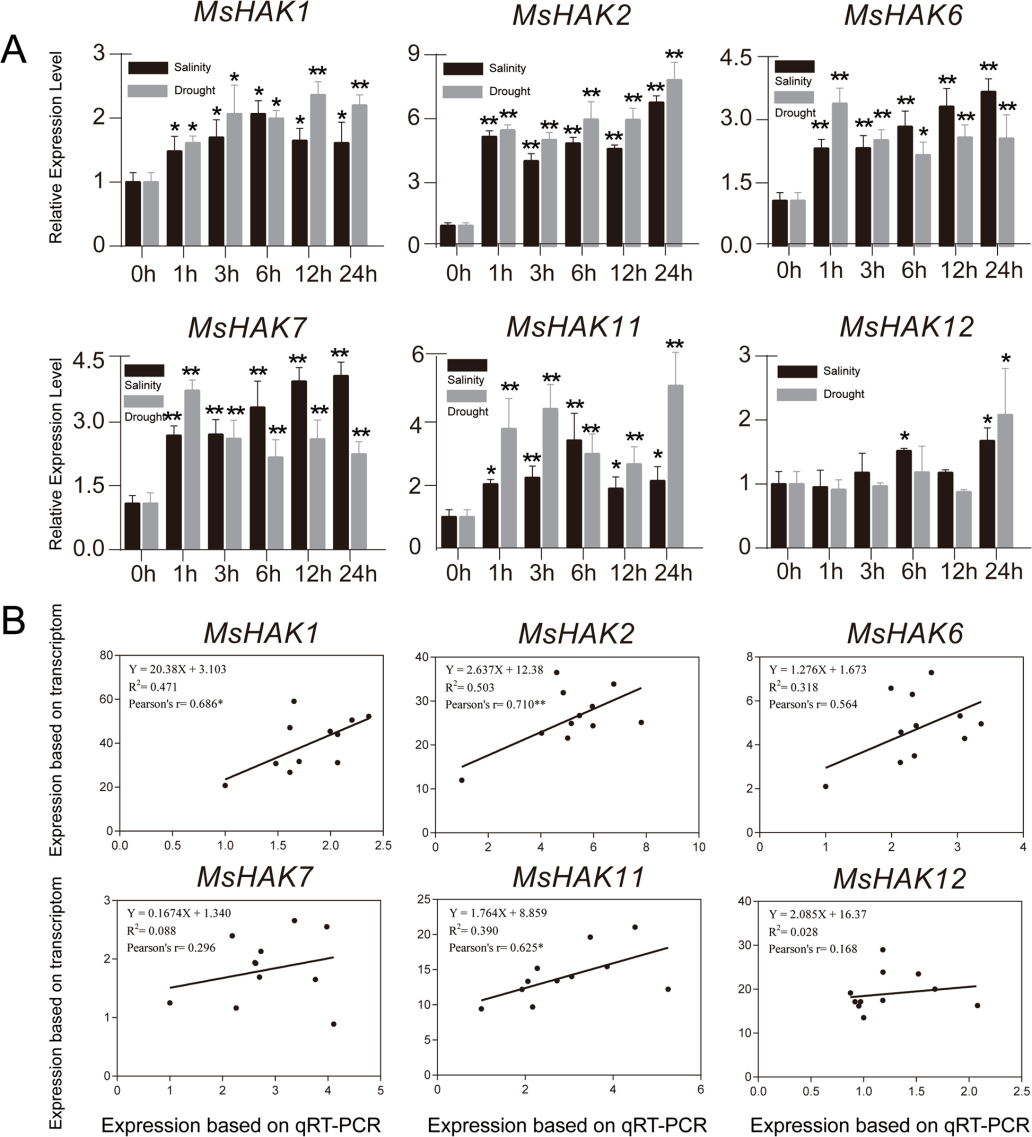
Quantification of gene expression levels of selected *HAK* genes from *M. sativa* using RT-qPCR. **A**: Expression level of *MsHAK* genes under NaCl and mannitol stress. *Actin* gene was used as house-keeping gene in RT-qPCR. Expression level of each gene at 0 h for each treatment was set as value of 1 and their expression levels at each time point were relative to 0 h. Data are average of three independent biological samples ± SE and vertical bars indicate standard deviation. ** indicates *P* < 0.01, and * indicates *P* < 0.05. **B**. Correlation analysis of RT-qPCR and transcriptome (NaCl and mannitol) data for *MsHAK* genes. Pearson’s value indicates the Pearson correlation coefficient

RT-qPCR were performed to verify the transcript abundance of selective *HAK* genes in seedlings of *M. truncatula* and *M. sativa* under salt and drought treatments at 0 h, 1 h, 3 h, 6 h, 12 h, and 24 h (Figs. 8 and 9). In *M. truncatula*, all genes were highly induced under both stresses except *MtHAK9* that was not induced under NaCl treatment. The expression levels of *MtHAK5*, *MtHAK7*, and *MtHAK12* increased gradually from 2 to 24 h for the two treatments. In particular, the expression level of *MtHAK7* was induced by more than 10 folds at 6 h for both treatments (Fig. 8). *MtHAK2* responded to salt stress rapidly by more than 10-fold increase at 1 h and maintained a relatively high level from 3 to 24 h (Fig. 8).

RT-qPCR data showed that all *MsHAK* genes were highly induced by salt and drought treatment, except *MsHAK12* that was only slightly induced by both treatment at 24 h, which is the same as its homologous *MtHAK9* (Fig. 9). *MsHAK1* and *MsHAK2* showed identical trends at different time points under the two stresses. Notably, expression level of *MsHAK6* was the highest 1 h under drought treatment, while its expression level was significantly higher and increased at 6, 12, 24 h induced by NaCl stress, which was the same for *MsHAK7*.

Since the treatment time points for RT-qPCR were the same as for the transcriptome data, these two datasets thus were also compared for correlation analysis (Fig. 9). All RT-qPCR for six *MsHAK* genes were positively correlated with transcriptome data, and *MsHAK2* had the highest correlation coefficient of 0.710 than for the other five genes.

## Discussion

The plant HAK/KUP/KT family showed a major function in root K^+^ acquisition and they also play a vital role in environmental stress adaptation and regulation of cell size [10]. K^+^ also play crucial roles in enzyme activation, anion neutralization, osmoregulation, and ultimate improvement of plant yield [7]. In the current study, we identified a total of 22 *HAK* genes in *M. truncatula* and 22 *HAK* genes in *M. sativa*. Multiple sequence alignment confirmed that almost all *M. truncatula* and *M. sativa* HAK members contained HAK/KUP/KT domains (Additional Fig. S2) and there is no clear preference for the N-terminus or C-terminus. They shared highly conserved amino acid length, which was consistent with those of the model plants such as *Arabidopsis* [36], rice [37] and maize [16]. *AtHAKs* and *OsHAKs* were divided into four clusters on the basis of phylogenetic analysis, with cluster I, II, II and IV, respectively. *HAK* genes of *Medicago* can also be classified into four clusters according to the classification criteria for *Arabidopsis* and rice, with cluster I (A, B), II (A, B), III (A, B), and IV (A, B) (Fig. 1), which implied the taxonomy and evolution of *HAK* gene family members were quite conservative in *Medicago.* Notably, cluster IA and IV do not contain members from *Arabidopsis*, and *OsHAK13* was positioned in cluster IIB previously [37], while it was positioned in cluster III B in this study. This inconsistency might be due to different methods or parameters were used for protein alignment and phylogenetic construction. Different cluster showed varied functions, the *HAK* genes in groups I was proven to be mainly involved in high-affinity K^+^ uptake and translocation. Some genes of Cluster IB (Fig. 2), such as *AtHAK5*, was associated with high-affinity K^+^ uptake and mostly expressed in root parts [24], which can help roots to absorb K^+^ under K^+^ deficiency stress environment. Remarkably, more genes in groups II, III, and IV have been reported to be associated with various plant developmental processes, for example, *AtKUP5* (IIIb) [25]. These genes served different functions in different evolutionary processes, providing theoretical reference for the understanding of functions of *HAK* gene in *Medicago.*

In plants, members of the *HAK* gene family display a low conservation in their exon/introns structures. The number of exons in *Mt*/*MsHAK* genes ranged from 4 to 10 (Fig. 2B), which is very similar to that of rice (2 to 10) [37] and maize (3 to 10) [16]. Mt/MsHAK family members with two or three homologies contained different exon numbers. For instance, *MsHAK7* and *MtHAK5* had 8 exons, whereas *MsHAK6* had three exons. This suggests that exon acquisition or loss might have occurred in this gene family during evolution, leading to various structures of homoeologous genes.

Duplication and divergence play critical role in the expansion and evolution of gene families [38, 39]. In previous studies, segmental duplications (SD) and tandem duplications (TD) events were reported as the major contributors to the large *HAK* gene expansion in many plant genomes [16, 40]. However, in this study, we found that not only intraspecies duplication events (Mt-SD, 10%; Ms-TD, 20%) (Fig. 3A, B) but also interspecies duplication (Mt-Gm, 28; Ms-Gm, 22) were the major contributors to the rapid gene expansion of the HAK family in *Medicago* (Fig. 3C). This same scenario was also demonstrated in *Medicago* (Mt-Ms, 10) HAK family (Fig. 3C), three genes in *M. truncatula* (*MtHAK1*, *4* and *7*) showed a collinear relationship with those in *M. sativa*, *G. max* and *A. thaliana* (Fig. 3C and Table S2); Four genes in *M. sativa* (*MsHAK1*, *3*, *11* and *7*) showed a collinear relationship with those in *A. thaliana* and *G. max* (Fig. 3C and Table S2). Finally, two pairs of homologous genes from *Medicago*, *MtHAK1*/*MsHAK1*, and *MtHAK7*/*MsHAK11*, are collinear with *A. thaliana* and *G. max.* Thus, the expansion of the *HAK* genes could be an indication that *HAK* genes play key roles in multiple biological processes. This study showed that around 46% (20/44) of *HAK* genes from *M. truncatula* and *M. sativa* were involved in duplicated genomic blocks, and the Ka/Ks values of all orthologous gene pairs are less than 1 (Fig. 3D, Table S2). This suggests that a strong purifying selection on the *Medicago* orthologous HAKs to remove deleterious mutations at protein level [39].

*Cis*-acting elements played pivotal function in the regulation of gene expression by controlling the efficiency of the promoters. Studies on *cis*-acting elements could provide key foundation for further functional characterization of the *HAK* gene family [16]. Previous studies have demonstrated that HAK plays an important role in salt and drought resistance [21, 22]. In our studies, TGACG-motif (MeJA-responsive), ABRE (abscisic acid-responsive), ERE (ethylene-responsive), TC-rich repeats, W-box (defense and stress responsiveness) and ARE (anaerobic induction) elements were found widely distributed in *HAK* genes (Fig. 4). *MtHAK9*, *MsHAK6* and *MsHAK12* contain 4 MeJA-responsive element, *MtHAK1*, *MtHAK2* and *MsHAK7* had 8, 4 and 3 abscisic acid-responsive element, respectively; *MsHAK7* contained 4 drought-inducibility elements (Fig. 4). And these results suggested that *HAK* genes of *Medicago* are likely involved in salt and drought stress.

Drought and salt are the most prevalent and severe abiotic stress factors for plant growth [41]. It is urgent to improve the salinity and drought tolerance of *M. sativa* to increase yield. Genechip data and transcriptome data under NaCl and drought treatments, along with expression profiles in various tissues suggested that the expressions of six homologous gene pairs (except for *MtHAK1*) were highly induced or drastically changed in *Medicago* (Fig. 5). In addition, the expression pattern of all six genes under NaCl and drought treatments were verified by RT-qPCR analyses in *M. truncatula* and *M. sativa* (Figs. 6 and 7A), and the results suggested that the vast majority genes are involved in NaCl and/or drought induction. Correspondingly, five genes pair (*MtHAK1*/*MsHAK1*, *MtHAK2*/*MsHAK2*, *MtHAK5*/*MsHAK7*, *MtHAK7*/*MsHAK11* and *MtHAK12*/*MsHAK2*) were significantly up-regulated under NaCl and mannitol treatments, and their expression pattern were the same in *M. truncatula* and *M. sativa*. Meanwhile, *MtHAK1*/*MsHAK1*, and *MtHAK7*/*MsHAK11* were identified by WGCNA analysis (Figs. 6I and 7I). These evidences indicated that these genes are likely key genes in response to NaCl and drought stress in *Medicago*.

Previous studies have confirmed that maintaining efficient K^+^ uptake is prerequisite for K^+^/Na^+^ homeostasis and salt tolerance when plants are exposed to salt stress [42]. In this study, the expression of five *HAK* genes pairs (except *MtHAK9*/*MsHAK12*) was up-regulated under salt stress (Figs. 6 and 7A), contributing to high K^+^/Na^+^ ratio and salt tolerance. The similar results were also observed for *OsHAKs* in rice [37]. *AtKUP7* is responsible for K^+^ uptake at high K^+^ concentrations, and may be also involved in K^+^ transport into xylem sap, affecting K^+^ translocation from roots to shoots [43]. The homologous gene pair *MtHAK1*/*MsHAK1* are more closely related with *AtKUP7*, and they exhibited high expression under salt and drought stress, indicating that these two genes are possibly involved in the transport of K^+^ under stress conditions. *HAKs* could enhance drought tolerance through absorbing and accumulating K^+^ to increase cytosolic ion concentration for plants [44, 45]. In the present study, the expression level of several *Mt*/*MsHAK* genes were up-regulated when plants were exposed to drought stress (Figs. 6 and 7 A), being consistent with the previous reports on *TaHAKs* in wheat [46] and *MeKUPs* in cassava [27]. In addition, *AtKUP2* mutation affects cell expansion and leads to developmental defects in shoots [47], and its close homologous gene *MtHAK12* was highly up-regulated under both treatments, suggesting that *MtHAK12* may also be involved in early stem development and stress responses.

## Conclusion

A total of 22 and 22 *HAK* genes were identified in *M. truncatula* and *M. sativa*, respectively. Phylogenetic analysis suggested that these HAK proteins could be divided into four groups, and the members of the same subgroup share similar gene structure characteristics and conserved motifs. Many *cis*-acting elements related with defense and stress, and anaerobic induction was found in their promoter region. In addition, gene expression profiles showed that these *HAK* genes exhibited distinct expression pattern in different tissues, and in response to salt and drought treatments. Furthermore, co-expression analysis showed that 6 homologous *HAK* hub gene pairs involved in direct network interactions. RT-qPCR verified that the expression level of six *HAK* gene pairs were induced by NaCl and mannitol treatment to different extent. In particular, *MtHK2*/*7*/*12* from *M. truncatula* and *MsHAK2*/*6*/*7* from *M. sativa* were dramatically induced. The expression level of *MsHAK1*/*2*/*11* determined by RT-qPCR showed significantly positive correlation with transcriptome data.

## Materials and methods

### Identification of *HAK* family members in Medicago

The genomic data of *M. truncatula* and *M. sativa* were downloaded from the website of https://figshare.com/articles/dataset/Medicago_sativa_genome_(accessed on 1 May 2022) and_annotation_files/12,623,960 and http://www.medicagogenome.org/ (accessed on 1 May 2022), respectively. We used the Hidden Markov Model (HMM) profile of the K^+^ transporter domain (PF02705) to query the candidate HAKs from *Medicago* genomic database using HMMER3.0 with a cutoff of 0.01 [48]. Then, we examined the candidate HAK protein sequences, which include the K^+^ transporter domain, using the Pfam database (http://pfam.xfam.org) (accessed on 5 May 2022) and SMART program (http://smart.embl-heidelberg.de/) (accessed on 5 May 2022). Further BLASTP search with an E-value cutoff of e^− 10^ was performed to sort HAK family members using *AtHAK* [36], *OsHAK* [37] and *ZmHAK* [16] as queries, which were retrieved from TAIR (http://www.arabidopsis.org) (accessed on 10 May 2022) and RICEDATA (http://www.ricedata.cn/gene) (accessed on 10 May 2022), respectively. In order to ensure the correctness of the selected genes, output putative HAK protein sequences were submitted to InterProScan (https://www.ebi.ac.uk/interpro/search/sequence-search) (accessed on 12 May 2022), CDD (https://www.ncbi.nlm.nih.gov/Structure/bwrpsb/bwrpsb.cgi) (accessed on 12 May 2022), Pfam (https://pfam.xfam.org/) (accessed on 15 May 2022), and SMART (http://smart.embl-heidelberg.de/) (accessed on 15 May 2022). Finally, all predicted protein sequences were curated manually by softberry (http://linux1.softberry.com/) (Additional Fig. S1). In total, 22 *MtHAK* and 22 *MsHAK* genes were identified, and assigned based on their locations on chromosome (Table 1). Correspondingly, ExPASy (https://web.expasy.org/compute_pi/) (accessed on 17 May 2022) was used to determine the isoelectric point (pI) and molecular weight (MW) of HAK proteins. Subcellular localization of HAK proteins were predicted by using the Softberry Home Page (http://linux1.softberry.com/berry.phtm) (accessed on 17 May 2022).

### Analyses on sequence, conserved motif and structural characterization

Conserved motifs were identified by selecting motifs from the MEME program (http://meme-suite.org/tools/meme) (accessed on 24 May 2022) with the motif number of HAK was set as 20, and the width range of 10 to 200 amino acids (aa). Subsequently, sequence alignment was carried out by using jalview (http://www.jalview.org/Web_Installers/install.htm). The visualization of exon-intron positions and conserved motifs were executed through the TBtools software.

### Analyses of phylogenetic relationship

The HAK protein sequences of *A. thaliana*, *O. sativa* and *G. max* were downloaded from Phytozome (https://phytozome-next.jgi.doe.gov/) (accessed on 20 May 2022). We constructed phylogenetic trees using MEGA7.0 according to the maximum likelihood method and performed bootstrap testing with 1000 replicates [49]. Meanwhile, subfamily clustering on phylogenetic tree was determined based on that of *Arabidopsis*. Subsequently, EvolView (https://evolgenius.info/evolview-v2/) (accessed on 25 May 2022) was used to view the phylogenetic tree.

### Analysis of chromosome locations and collinearity

The loci of *HAK* genes were obtained from the genome annotation data. TBtools software was applied to map the chromosome locations for each genes. Next, these sequences were analyzed to identify collinearity blocks against the whole genome using MCSCAN (http://chibba.agtec.uga.edu/duplication/mcscan/) (accessed on 26 May 2022) [50]. Moreover, intraspecific synteny relationship (*M. truncatula* and *M. sativa*) and interspecific synteny relationship (*M. truncatula* and *M. sativa*, *M. truncatula* and *G. max*, *M. truncatula* and *A. thaliana*, *M. sativa* and *G. max*, *M. sativa* and *A. thaliana*) were analyzed, and they were mapped to the chromosomes of *M. truncatula* and *M. sativa* using TBtools, respectively [33]. Lastly, the synonymous (Ks) and nonsynonymous (Ka) substitution rates were estimated using TBtools [51].

### Analyses of cis-acting elements and location of HAK genes in Medicago

*Cis*-acting elements were searched from upstream regions (2000 bp) of all *HAK* genes. The *cis*-acting elements in the promoter region were analyzed at the PlantCARE website (http://bioinformatics.psb.ugent.be/webtools/plantcare/html/) (accessed on 27 May 2022) [52]. The visualize models of *cis*-acting elements in the promoters were carried out with TBtools.

### Analysis of expression level of HAK genes

Genechip data from roots and shoots and those under drought and salt stress conditions for *MtHAK* genes were downloaded from the *M. truncatula* Gene Expression Atlas (https://Mtgea.noble.org/v3/) (accessed on 28 May 2022). Two-week-old *M*. *truncatula* seedlings (cv. Jemalong line A17) were treated with 200 mM salt solution at 0 h, 6 h, 24 and 48 h, with biological triplicates. The roots under hydroponics condition were treated with 200 mM salt solution at 0 h, 1 h, 2 h, 5 h, 10 and 24 h. When the fourth shoot emerging out, roots and shoots were harvested separately at 2 d, 3 d, 4 d, 7 d, 10 d and 14 d after water withholding, 2-day well-watered plants were treated as control, respectively, and all experiments were performed with biological triplicates. The expression level of *MtHAK* genes in root, stem, leaf, flower, pod, seed were also analyzed. Amazing HeatMap software was used to generate heatmap [51]. The original transcriptome data from *M. sativa* under NaCl and mannitol treatments at 0 h, 1 h, 3 h, 6 h, 12 h, 24 h (SRR7160314-15, 22–23, 25–49, 51–52, 56–57) were downloaded from NCBI database [53]. Twelve-day-old alfalfa (Zhongmu No. 1) seedlings under hydroponics condition were separated into three groups: (1) control (1/2 MS nutrient solution), (2) salt (250 mM NaCl), (3) drought (400 mM mannitol). The treatment time during were 1 h, 3 h, 6 h, 12 h, and 24 h with biological triplicates.

Then the data was converted to fastq files using SRA-Toolkit v2.9 [54]. Raw reads were trimmed using the Trimmomatic-0.39 [55]. Gene expression level was determined by mapping cleaned reads to the corresponding *M. sativa* reference genomes using the StringTie v2.1.3 package [56].

### Co-expression network construction and GO enrichment analysis of *Mt/MsHAK* gene family

The WGCNA package were used for co-expression network analysis, four co-expression networks were constructed, including *M. truncatula*/*M. sativa* response to NaCl/drought stresses, respectively. To identify differentially expressed genes, the probe sets of *M. truncatula* (RPKM less than 10) and the genes of *M. sativa* (FPKM less than 1) that show very low expression levels were removed. Then, genes with different expression levels were determined by comparing different stress treatments and control using DEseq by multiple-factor design (fold change greater than 2 and *Padj* < 0.05). After these filtering steps, 10,658/15,175 differential expressed genes of *M. truncatula* response to NaCl/drought stresses, and 14,144/12,677 differentially expressed genes of *M. sativa* response to NaCl/drought stresses remained. The co-expression gene network for those selected genes were constructed, a *β* (soft thresholding power) value of 9 was selected based on the scale-free topology criterion. The Pearson algorithm was used to calculate the correlation coefficient, and the results were stored as a signed co-expression matrix. The resulting adjacency matrix was converted to a topological overlap (TO) matrix via TOM similarity algorithm, and the genes were hierarchically clustered based on TO similarity. The parameters for TOMType were: MaxBlocksize was 60,000, minModuleSize was 30, deepSplit was 2, and mergeCutHeight was 0.25. Finally, network visualization for each module was performed with genes directly/indirectly related to *Mt*/*MsHAK* genes using the Cytoscape version 3.7.2, and the edges were filtered with weights below 0.1 to ensure reliability and readability of the results. Gene Ontology pathway enrichment analysis for the involved modules was performed using TBtools. The GO terms with a corrected *p*-value ≤ 0.05 were considered to be significantly enriched.

### Plant materials and treatments

*M. truncatula* (cv. Jemalong A17) and *M. sativa* (cv. Zhongmu No.1) plants used in this study were generated and stored at the Institute of Animal Sciences of Chinese Academy of Agricultural Sciences. Roots, stems, leaves, flowers of mature *M. truncatula* and *M. sativa* plants, were collected separately for RNA extraction and RT-qPCR analysis. To investigate the expression pattern of *HAK* genes in response to NaCl and mannitol stress, seeds were germinated and transferred into the MS solid medium (MS basal salts supplemented with 30 g/L sucrose, 0.8% (w/v) agar), then kept in a growth chamber at 25 °C under a photoperiod of 16/8 light/dark regime. When the third leaf was fully expanded, seedlings were transferred to fresh MS solid medium supplied with 300 mM NaCl and 15% mannitol, respectively, and the whole plants were collected at 0 h, 1 h, 3 h, 6 h, 12 and 24 h for each treatment. The sample were frozen in liquid nitrogen and stored at -80 °C for subsequent analysis.

### RNA extraction, analysis of the gene expression by RT-qPCR and correlation analyses

The detailed procedures of total RNA extraction, first strand cDNA synthesis and RT-qPCR were described according to the manufacturer’s instructions (TIANGEN, Beijing). Each reaction was performed in biological triplicates with *actin* gene as references and the data from RT-qPCR was analyzed using 2^−△△CT^ method. The results were analyzed by means ± standard deviation (student’s t test n = 3, * *p* < 0.05, ** *p* < 0.01). The primer sequences used in this study were listed in Table S1. GraphPad Prism 6.0 was used to draw scatter plots of gene expression, and linear regression analysis and Pearson correlation analysis were performed between the expression levels analyzed by transcriptome and by RT-qPCR.

## Supplementary Information

**Additional file 1:** **Fig. S1.** Conserved protein motif analysis of HAK family members before and after correction manually by softberry. ‘ori’ represents the original predicted gene sequence, and ‘new’ represents the new predicted gene sequence. **Fig. S2.** Multiple sequence alignment of MtHAKs and MsHAKs. The alignment were performed by MEGA and visualized by Jalview. Residues with more than 50% similarity were shaded. Conserved regions (KUP/HAK/KT) were indicated at the top. **Fig. 3.** The sequence information of 20 conserved motifs of HAK gene in Medicago, including the sequence logo and amino acids, as well as amino acids numbers of each motif.

**Additional file 2:** **Supplementary Table 1.** List of primers used in this research. **Supplementary Table 2.**  The synteny gene pairs of *Medicagotruncatula*, *Medicago sativa*, *Glycine max* and *Arabidopsisthaliana*, as well as the KaKs of comparative synteny gene pairs. **SupplementaryTable 3.**  List of all identified *cis*-acting elements in all *HAK*genes found in *Medicago. ***Supplementary Table4-1.** Detailed information on the available tissue expression levels of MtHAKgenes retrieved from microarray data for *M. truncatula. ***Supplementary Table4-2.** Detailed information on the available tissue expression levels of MsHAKgenes retrieved from transcriptome data for *M. sativa. ***Supplementary Table4-3.** Detailed information on the available expression levels of* MsHAK*genes response to salinity stress treatments retrieved from microarray data for*M. truncatula. ***Supplementary Table4.** Detailed information on the available expression levels of *MsHAK*genes response to drought stress treatments retrieved from microarray data for *M.truncatula*. **Supplementary Table4-5.** Detailed information on the available expression levels of *MsHAK*genes response to salinity stress treatment retrieved from transcriptome datafor *M. sativa. ***Supplementary Table4-6.** Detailed information on the available expression levels of MsHAK genesresponse to drought stress treatment retrieved from transcriptome data for *M.sativa. ***Supplementary Table5-1. **The co-expression network of* MtHAK* genes in DEGs under salt stress. **Supplementary Table5-2.** The co-expression network of MtHAK genes in DEGs under drought stress. **Supplementary Table5-3.** The co-expression network of *MsHAK *genes in DEGs under salt stress. **Supplementary Table5-4**. The co-expression network of *MsHAK* genes in DEGs under drought  stress. 

## Data Availability

All the data presented in this manuscript are available in the main text or in the supplementary material.
