# Supplementary material for: Genome-wide characterization and expression analysis of the HAK gene family in response to abiotic stresses in Medicago
Source: BMC Genomics. 2022 Dec 1;23:791. doi: 10.1186/s12864-022-09009-2 (PMC9714174; doi:10.1186/s12864-022-09009-2)
Supplement: Supplementary file 1 — Additional file 1: Fig. S1. Conserved protein motif analysis of HAK family members before and after correction manually by softberry. ‘ori’ represents the original predicted gene sequence, and ‘new’ represents the new predicted gene sequence. Fig. S2. Multiple sequence alignment of MtHAKs and MsHAKs. The alignment were performed by MEGA and visualized by Jalview. Residues with more than 50% similarity were shaded. Conserved regions (KUP/HAK/KT) were indicated at the top. Fig. 3. The sequence information of 20 conserved motifs of HAK gene in Medicago, including the sequence logo and amino acids, as well as amino acids numbers of each motif. [file 12864_2022_9009_MOESM1_ESM.docx]

**Supplementary Materials**


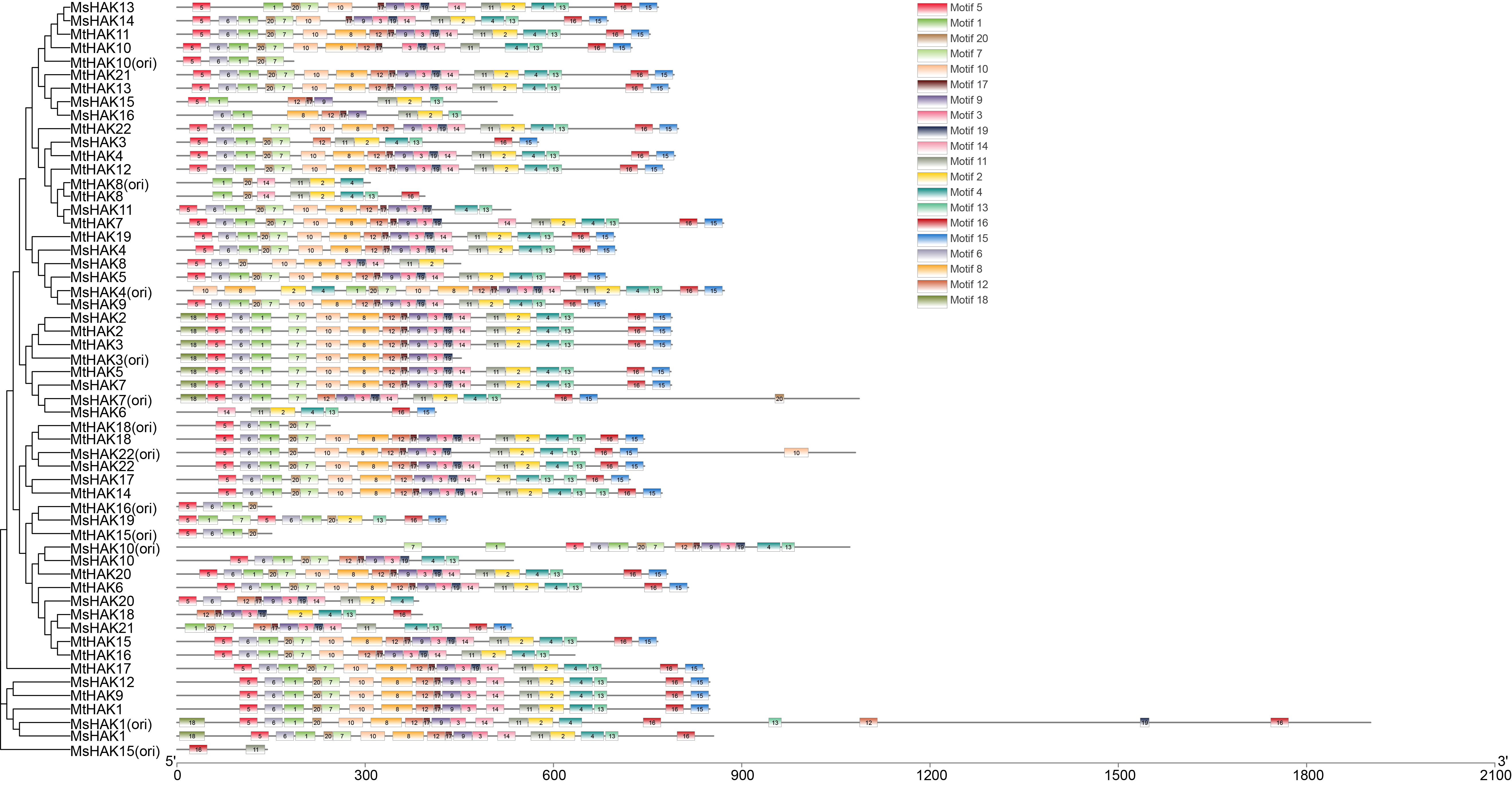


**Additional Fig. S1.** Conserved protein motif analysis of *HAK* family members before and after correction manually by softberry. ‘ori’ represents the original predicted gene sequence, and ‘new’ represents the new predicted gene sequence.


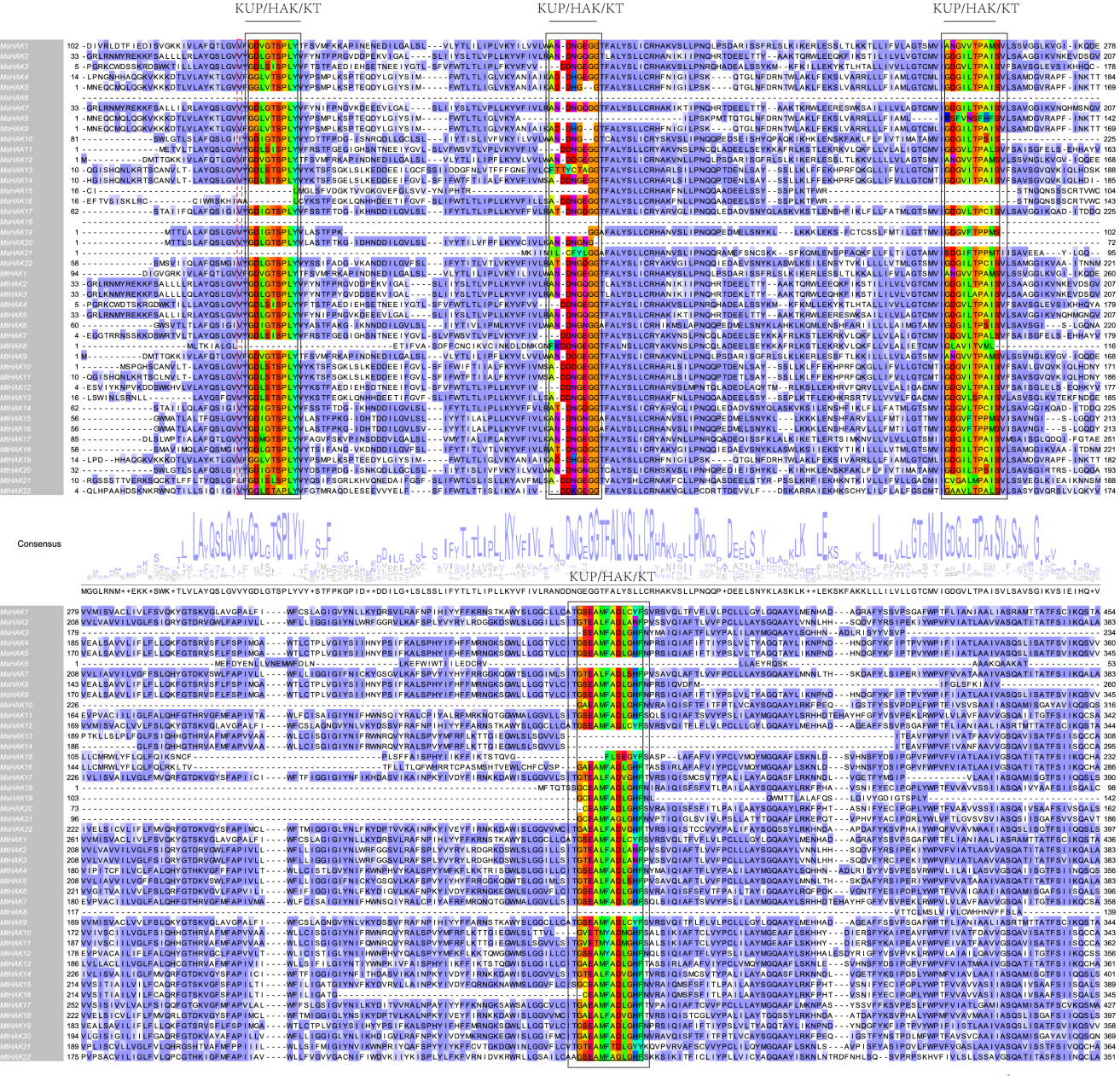


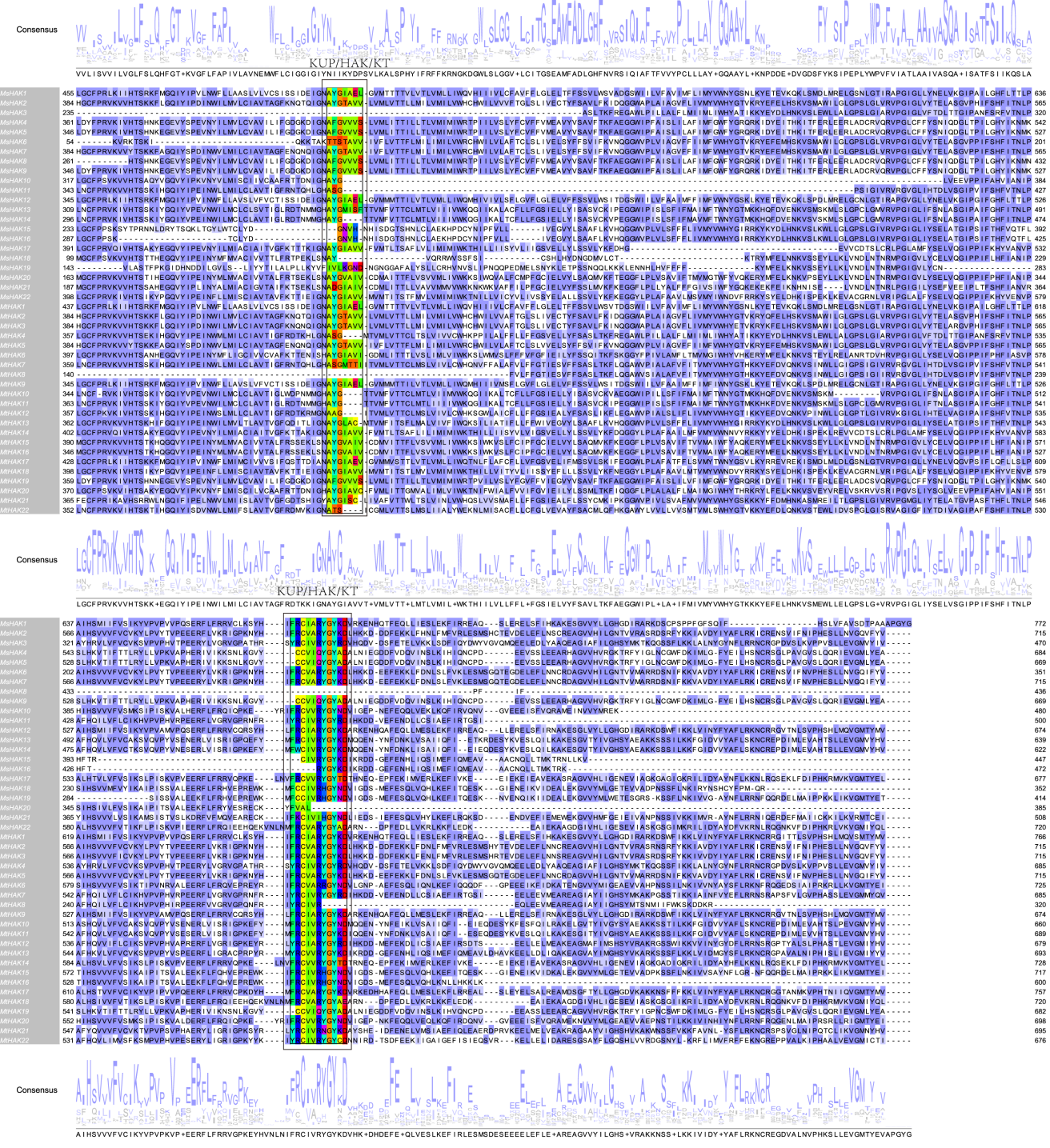

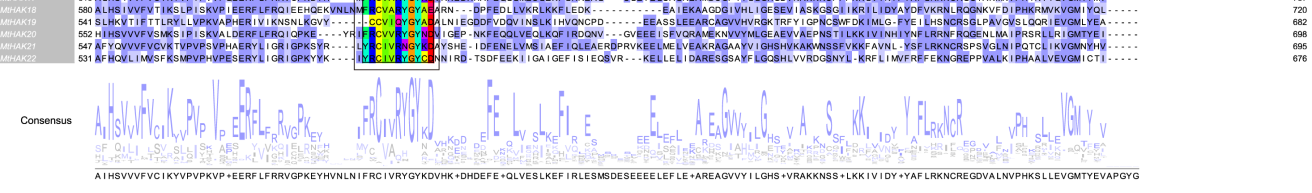


**Additional Fig. S2**

Multiple sequence alignment of *MtHAKs* and *MsHAKs*. The alignment were performed by MEGA and visualized by Jalview. Residues with more than 50% similarity were shaded. Conserved regions (KUP/HAK/KT) were indicated at the top.


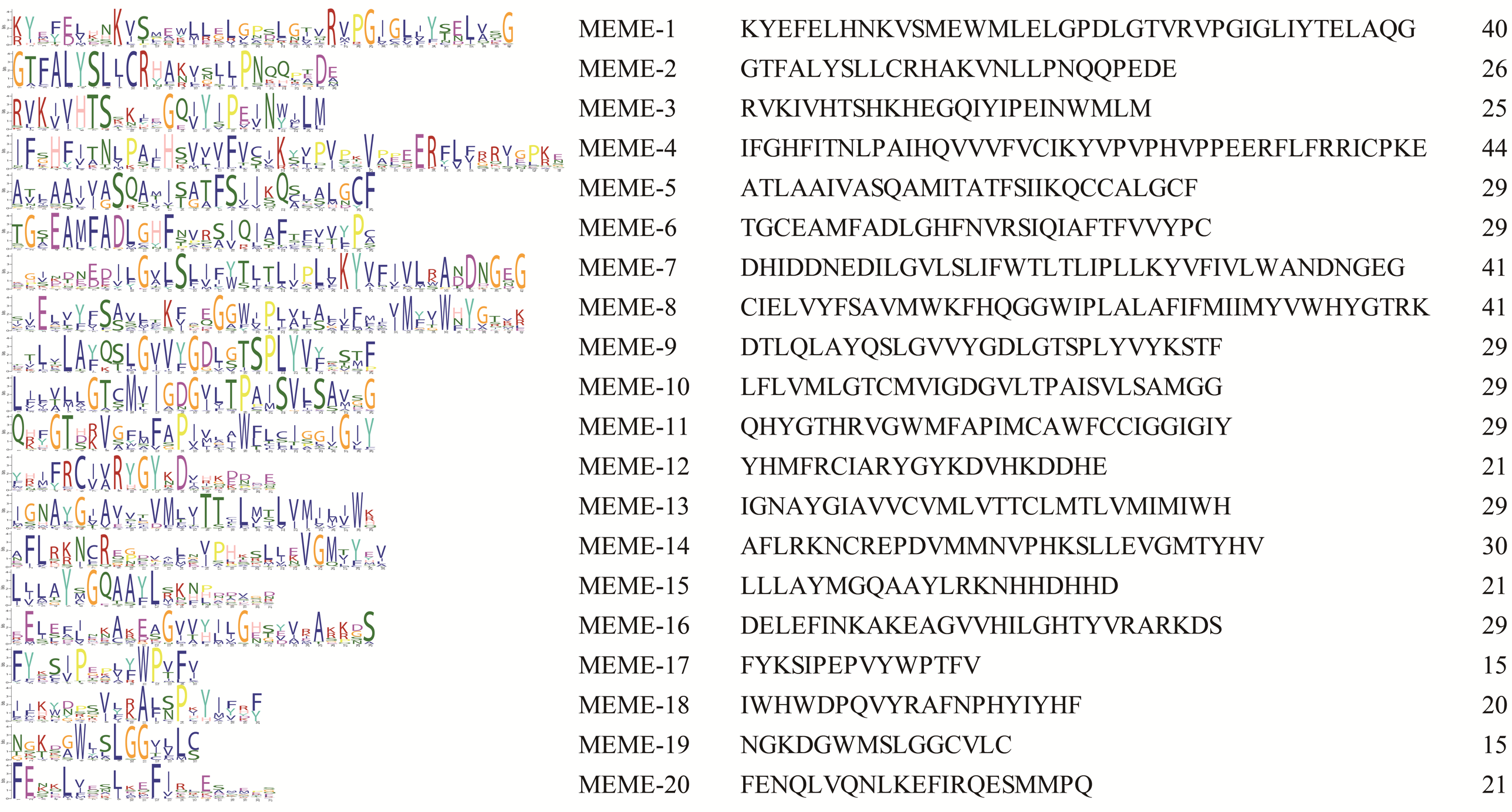


**Additional Fig. S3**

The sequence information of 20 conserved motifs of *HAK* gene in *Medicago*, including the sequence logo and amino acids, as well as amino acids numbers of each motif.
