# Supplementary material for: Genome-wide characterization and expression analysis of the HAK gene family in response to abiotic stresses in Medicago
Source: BMC Genomics. 2022 Dec 1;23:791. doi: 10.1186/s12864-022-09009-2 (PMC9714174; doi:10.1186/s12864-022-09009-2)
Supplement: Supplementary file 2 — Additional file 2: Supplementary Table 1. List of primers used in this research. Supplementary Table 2. The synteny gene pairs of Medicagotruncatula, Medicago sativa, Glycine max and Arabidopsisthaliana, as well as the KaKs of comparative synteny gene pairs. SupplementaryTable 3. List of all identified cis-acting elements in all HAKgenes found in Medicago. Supplementary Table4-1. Detailed information on the available tissue expression levels of MtHAKgenes retrieved from microarray data for M. truncatula. Supplementary Table4-2. Detailed information on the available tissue expression levels of MsHAKgenes retrieved from transcriptome data for M. sativa. Supplementary Table4-3. Detailed information on the available expression levels of MsHAKgenes response to salinity stress treatments retrieved from microarray data forM. truncatula. Supplementary Table4. Detailed information on the available expression levels of MsHAKgenes response to drought stress treatments retrieved from microarray data for M.truncatula. Supplementary Table4-5. Detailed information on the available expression levels of MsHAKgenes response to salinity stress treatment retrieved from transcriptome datafor M. sativa. Supplementary Table4-6. Detailed information on the available expression levels of MsHAK genesresponse to drought stress treatment retrieved from transcriptome data for M.sativa. Supplementary Table5-1. The co-expression network of MtHAK genes in DEGs under salt stress. Supplementary Table5-2. The co-expression network of MtHAK genes in DEGs under drought stress. Supplementary Table5-3. The co-expression network of MsHAK genes in DEGs under salt stress. Supplementary Table5-4. The co-expression network of MsHAK genes in DEGs under drought stress. [file 12864_2022_9009_MOESM2_ESM.docx]

| Supplementary Table 1 List of primers used in this research | |  |
| --- | --- | --- |
| Gene | Forward primer/ Reverse primer（5’-3’） | Size (bp) |
| MtActin | TGATGATGTTTGTTGGGCTTTGG | 114 |
|  | ATGTTGATGTTGATGTTGTTGTTACC |  |
| MtHAK1 | AAGTTCCACAACCTCCGATTCC | 136 |
|  | AGTCCTTCCTTGCTCTTATATCTCC |  |
| MtHAK2 | AGCCACAATCACCGCAACC | 292 |
|  | ACGCAACAACGAGAATCCAATG |  |
| MtHAK5 | CGCACCGATGAAGAACTAACAAC | 251 |
|  | AACAGCAATGAGAACTACAACACC |  |
| MtHAK7 | TGTGTCAATGTTGTGTCTTATCAGTG | 276 |
|  | TTGTGCGTGCCGTAGTGC |  |
| MtHAK9 | TTCTTGGAGCATTATCACTTGTCTTG | 97 |
|  | ACCTTCGCCGTCATCATTGG |  |
| MtHAK12 | ATGGTTATTGGTGATGGTGTTCTC | 159 |
|  | GAGTGCCGTAATGTTGAAGAGC |  |
| MsActin | CAAAAGATGGCAGATGCTGAGGAT | 114 |
|  | CATGACACCAGTATGACGAGGTCG |  |
| MsHAK1 | AAGTTCCACAACCTCCGATTCC | 136 |
|  | AGTCCTTCCTTGCTCTTATATCTCC |  |
| MsHAK2 | CATTTACTCTCGTGGTGTTTCCTTG | 184 |
|  | TGCGGTGATTGTGGCTTGG |  |
| MsHAK6 | TGTGGAATTGTCATACTTCTTCTCTG | 289 |
|  | AATGGATGGCTGGTAGGTTGG |  |
| MsHAK7 | CGCACCGATGAAGAACTAACAAC | 131 |
|  | GCCAGCAAGGACAAGAATAAGAATG |  |
| MsHAK11 | GGTTATTGGTGATGGTGTCCTTAC | 110 |
|  | GCAACTGGAACTTCTACATACGC |  |
| MsHAK12 | GGTTCCTGGTGCTCATACTCATC | 261 |
|  | ACCTTCGCCGTCATCATTGG |  |

| Supplementary Table. 2 The synteny gene pairs of *Medicago truncatula*, *Medicago sativa*, *Glycine max* and *Arabidopsis thaliana*, as well as the KaKs of comparative synteny gene pairs | | | | | | | | | | | | |
| --- | --- | --- | --- | --- | --- | --- | --- | --- | --- | --- | --- | --- |
| **Duplicated Type** | **Gene Name** | **Gene ID** | **Gene Name** | **Gene ID** | **Species comparison name** | **Ka** | **Ks** | **Ka_Ks** | **Effective Len** | **Average S-sites** | **Average N-sites** | **Selection pressure** |
| Segmental | *MtHAK2* | *MtrunA17_Chr2g0297291* | *MtHAK5* | *MtrunA17_Chr4g0052401* | Mt-Mt | 0.108 | 0.725 | 0.150 | 2349 | 566 | 1783 | Purifying selection |
| Segmental | *MsHAK2* | *MsG0280008140.01.T01* | *MsHAK6* | *MsG0480022802.01.T01* | Ms-Ms | 0.174 | 0.767 | 0.226 | 1146 | 266 | 880 | Purifying selection |
| Tandem | *MsHAK4* | *MsG0480022271.01.T01* | *MsHAK5* | *MsG0480022272.01.T01* | Ms-Ms | 0.008 | 0.012 | 0.688 | 2055 | 494 | 1561 | Purifying selection |
| Tandem | *MsHAK8* | *MsG0480022868.01.T01* | *MsHAK9* | *MsG0480022869.01.T01* | Ms-Ms | 0.054 | 0.079 | 0.685 | 1344 | 318 | 1026 | Purifying selection |
| Segmental | *MtHAK2* | *MtrunA17_Chr2g0297291* | *MsHAK2* | *MsG0280008140.01.T01* | Mt-Ms | 0.006 | 0.113 | 0.055 | 2367 | 574 | 1793 | Purifying selection |
| Segmental | *MtHAK1* | *MtrunA17_Chr2g0279131* | *MsHAK1* | *MsG0280006462.01.T01* | Mt-Ms | 0.024 | 0.129 | 0.185 | 2481 | 598 | 1883 | Purifying selection |
| Segmental | *MtHAK5* | *MtrunA17_Chr4g0052401* | *MsHAK2* | *MsG0280008140.01.T01* | Mt-Ms | 0.109 | 0.690 | 0.158 | 2349 | 566 | 1783 | Purifying selection |
| Segmental | *MtHAK4* | *MtrunA17_Chr3g0128831* | *MsHAK3* | *MsG0380016724.01.T01* | Mt-Ms | 0.007 | 0.076 | 0.091 | 1728 | 416 | 1312 | Purifying selection |
| Segmental | *MtHAK19* | *MtrunA17_Chr8g0375411* | *MsHAK9* | *MsG0480022869.01.T01* | Mt-Ms | 0.023 | 0.099 | 0.233 | 2055 | 494 | 1561 | Purifying selection |
| Segmental | *MtHAK7* | *MtrunA17_Chr5g0413721* | *MsHAK11* | *MsG0580025786.01.T01* | Mt-Ms | 0.018 | 0.056 | 0.325 | 1596 | 390 | 1206 | Purifying selection |
| Segmental | *MtHAK9* | *MtrunA17_Chr5g0429991* | *MsHAK12* | *MsG0580028415.01.T01* | Mt-Ms | 0.005 | 0.043 | 0.120 | 2547 | 599 | 1948 | Purifying selection |
| Segmental | *MtHAK10* | *MtrunA17_Chr5g0430551* | *MsHAK13* | *MsG0580028458.01.T01* | Mt-Ms | 0.067 | 0.163 | 0.409 | 2004 | 464 | 1540 | Purifying selection |
| Segmental | *MtHAK11* | *MtrunA17_Chr5g0430731* | *MsHAK13* | *MsG0580028458.01.T01* | Mt-Ms | 0.045 | 0.135 | 0.335 | 2079 | 482 | 1597 | Purifying selection |
| Segmental | *MtHAK13* | *MtrunA17_Chr6g0461871* | *MsHAK16* | *MsG0680031789.01.T01* | Mt-Ms | 0.190 | 0.358 | 0.531 | 1599 | 374 | 1226 | Purifying selection |
| Segmental | *MtHAK14* | *MtrunA17_Chr7g0268981* | *MsHAK17* | *MsG0780041281.01.T01* | Mt-Ms | 0.011 | 0.067 | 0.169 | 2166 | 511 | 1655 | Purifying selection |
| Segmental | *MtHAK15* | *MtrunA17_Chr8g0344551* | *MsHAK20* | *MsG0880042787.01.T01* | Mt-Ms | 0.053 | 0.257 | 0.208 | 1155 | 275 | 880 | Purifying selection |
| Segmental | *MtHAK1* | *MtrunA17_Chr2g0279131* | *GmHAK6* | *Glyma.05G117500* | Mt-Gm | 0.076 | 0.658 | 0.116 | 2514 | 601 | 1913 | Purifying selection |
| Segmental | *MtHAK2* | *MtrunA17_Chr2g0297291* | *GmHAK7* | *Glyma.05G131800* | Mt-Gm | 0.087 | 0.634 | 0.138 | 2358 | 570 | 1788 | Purifying selection |
| Segmental | *MtHAK1* | *MtrunA17_Chr2g0279131* | *GmHAK16* | *Glyma.08G179100* | Mt-Gm | 0.051 | 0.414 | 0.123 | 2517 | 602 | 1915 | Purifying selection |
| Segmental | *MtHAK1* | *MtrunA17_Chr2g0279131* | *GmHAK13* | *Glyma.08G072500* | Mt-Gm | 0.075 | 0.636 | 0.118 | 2517 | 603 | 1914 | Purifying selection |
| Segmental | *MtHAK2* | *MtrunA17_Chr2g0297291* | *GmHAK14* | *Glyma.08G086300* | Mt-Gm | 0.086 | 0.632 | 0.135 | 2358 | 570 | 1788 | Purifying selection |
| Segmental | *MtHAK2* | *MtrunA17_Chr2g0297291* | *GmHAK19* | *Glyma.09G052700* | Mt-Gm | 0.090 | 0.559 | 0.161 | 2355 | 570 | 1785 | Purifying selection |
| Segmental | *MtHAK1* | *MtrunA17_Chr2g0279131* | *GmHAK22* | *Glyma.15G053400* | Mt-Gm | 0.053 | 0.431 | 0.122 | 2517 | 603 | 1914 | Purifying selection |
| Segmental | *MtHAK2* | *MtrunA17_Chr2g0297291* | *GmHAK23* | *Glyma.15G159000* | Mt-Gm | 0.087 | 0.527 | 0.166 | 2355 | 569 | 1786 | Purifying selection |
| Segmental | *MtHAK4* | *MtrunA17_Chr3g0128831* | *GmHAK5* | *Glyma.04G221600* | Mt-Gm | 0.058 | 0.575 | 0.100 | 2361 | 572 | 1789 | Purifying selection |
| Segmental | *MtHAK4* | *MtrunA17_Chr3g0128831* | *GmHAK9* | *Glyma.06G143800* | Mt-Gm | 0.056 | 0.543 | 0.102 | 2361 | 572 | 1789 | Purifying selection |
| Segmental | *MtHAK5* | *MtrunA17_Chr4g0052401* | *GmHAK7* | *Glyma.05G131800* | Mt-Gm | 0.058 | 0.409 | 0.141 | 2352 | 564 | 1788 | Purifying selection |
| Segmental | *MtHAK5* | *MtrunA17_Chr4g0052401* | *GmHAK19* | *Glyma.09G052700* | Mt-Gm | 0.107 | 0.604 | 0.178 | 2349 | 563 | 1786 | Purifying selection |
| Segmental | *MtHAK5* | *MtrunA17_Chr4g0052401* | *GmHAK23* | *Glyma.15G159000* | Mt-Gm | 0.107 | 0.634 | 0.169 | 2349 | 563 | 1786 | Purifying selection |
| Segmental | *MtHAK7* | *MtrunA17_Chr5g0413721* | *GmHAK1* | *Glyma.01G031800* | Mt-Gm | 0.120 | 0.776 | 0.155 | 2319 | 559 | 1760 | Purifying selection |
| Segmental | *MtHAK10* | *MtrunA17_Chr5g0430551* | *GmHAK3* | *Glyma.02G228500* | Mt-Gm | 0.099 | 0.404 | 0.245 | 2160 | 501 | 1659 | Purifying selection |
| Segmental | *MtHAK7* | *MtrunA17_Chr5g0413721* | *GmHAK18* | *Glyma.08G288500* | Mt-Gm | 0.145 | 0.776 | 0.186 | 2319 | 554 | 1765 | Purifying selection |
| Segmental | *MtHAK10* | *MtrunA17_Chr5g0430551* | *GmHAK20* | *Glyma.11G169900* | Mt-Gm | 0.141 | 0.586 | 0.241 | 2166 | 501 | 1665 | Purifying selection |
| Segmental | *MtHAK7* | *MtrunA17_Chr5g0413721* | *GmHAK27* | *Glyma.18G136500* | Mt-Gm | 0.152 | 0.851 | 0.178 | 2319 | 551 | 1768 | Purifying selection |
| Segmental | *MtHAK12* | *MtrunA17_Chr6g0451081* | *GmHAK21* | *Glyma.13G170700* | Mt-Gm | 0.064 | 0.625 | 0.102 | 2322 | 552 | 1770 | Purifying selection |
| Segmental | *MtHAK13* | *MtrunA17_Chr6g0461871* | *GmHAK24* | *Glyma.16G046200* | Mt-Gm | 0.075 | 0.743 | 0.100 | 2349 | 570 | 1779 | Purifying selection |
| Segmental | *MtHAK13* | *MtrunA17_Chr6g0461871* | *GmHAK25* | *Glyma.16G148800* | Mt-Gm | 0.137 | 0.555 | 0.247 | 2346 | 566 | 1780 | Purifying selection |
| Segmental | *MtHAK12* | *MtrunA17_Chr6g0451081* | *GmHAK28* | *Glyma.19G010900* | Mt-Gm | 0.061 | 0.636 | 0.096 | 2322 | 553 | 1769 | Purifying selection |
| Segmental | *MtHAK13* | *MtrunA17_Chr6g0461871* | *GmHAK29* | *Glyma.19G105900* | Mt-Gm | 0.078 | 0.747 | 0.104 | 2349 | 571 | 1778 | Purifying selection |
| Segmental | *MtHAK21* | *MtrunA17_Chr8g0387661* | *GmHAK8* | *Glyma.05G213100* | Mt-Gm | 0.085 | 0.353 | 0.241 | 2364 | 575 | 1789 | Purifying selection |
| Segmental | *MtHAK21* | *MtrunA17_Chr8g0387661* | *GmHAK11* | *Glyma.08G019600* | Mt-Gm | 0.086 | 0.353 | 0.243 | 2364 | 575 | 1789 | Purifying selection |
| Segmental | *MtHAK22* | *MtrunA17_Chr8g0393241* | *GmHAK12* | *Glyma.08G056100* | Mt-Gm | 0.113 | 0.475 | 0.238 | 2346 | 552 | 1794 | Purifying selection |
| Segmental | *MtHAK19* | *MtrunA17_Chr8g0375411* | *GmHAK15* | *Glyma.08G091900* | Mt-Gm | 0.079 | 0.471 | 0.169 | 2082 | 504 | 1579 | Purifying selection |
| Segmental | *MtHAK17* | *MtrunA17_Chr8g0365001* | *GmHAK17* | *Glyma.08G288400* | Mt-Gm | 0.067 | 0.484 | 0.139 | 2511 | 592 | 1919 | Purifying selection |
| Segmental | *MtHAK7* | *MtrunA17_Chr5g0413721* | *AtKUP6* | *AT1G70300* | Mt-At | 0.189 | 2.464 | 0.077 | 2319 | 557 | 1762 | Purifying selection |
| Segmental | *MtHAK4* | *MtrunA17_Chr3g0128831* | *AtKUP2* | *AT2G40540* | Mt-At | 0.144 | 3.133 | 0.046 | 2343 | 570 | 1773 | Purifying selection |
| Segmental | *MtHAK11* | *MtrunA17_Chr5g0430731* | *AtKUP1* | *AT2G30070* | Mt-At | 0.174 | 2.847 | 0.061 | 2130 | 505 | 1625 | Purifying selection |
| Segmental | *MtHAK21* | *MtrunA17_Chr8g0387661* | *AtKUP3* | *AT4G23640* | Mt-At | 0.270 | 5.874 | 0.046 | 2313 | 560 | 1753 | Purifying selection |
| Segmental | *MtHAK1* | *MtrunA17_Chr2g0279131* | *AtKUP7* | *AT5G09400* | Mt-At | 0.137 | 1.781 | 0.077 | 2514 | 603 | 1911 | Purifying selection |
| Segmental | *MtHAK12* | *MtrunA17_Chr6g0451081* | *AtKUP8* | *AT5G14880* | Mt-At | 0.164 | 2.593 | 0.063 | 2301 | 553 | 1748 | Purifying selection |
| Segmental | *MsHAK1* | *MsG0280006462.01.T01* | *GmHAK6* | *Glyma.05G117500* | Ms-Gm | 0.100 | 0.660 | 0.151 | 2481 | 595 | 1886 | Purifying selection |
| Segmental | *MsHAK2* | *MsG0280008140.01.T01* | *GmHAK7* | *Glyma.05G131800* | Ms-Gm | 0.086 | 0.597 | 0.144 | 2358 | 570 | 1788 | Purifying selection |
| Segmental | *MsHAK1* | *MsG0280006462.01.T01* | *GmHAK13* | *Glyma.08G072500* | Ms-Gm | 0.098 | 0.634 | 0.155 | 2481 | 597 | 1884 | Purifying selection |
| Segmental | *MsHAK1* | *MsG0280006462.01.T01* | *GmHAK16* | *Glyma.08G179100* | Ms-Gm | 0.077 | 0.423 | 0.181 | 2481 | 596 | 1885 | Purifying selection |
| Segmental | *MsHAK2* | *MsG0280008140.01.T01* | *GmHAK14* | *Glyma.08G086300* | Ms-Gm | 0.085 | 0.586 | 0.144 | 2358 | 571 | 1788 | Purifying selection |
| Segmental | *MsHAK2* | *MsG0280008140.01.T01* | *GmHAK19* | *Glyma.09G052700* | Ms-Gm | 0.089 | 0.538 | 0.165 | 2355 | 570 | 1785 | Purifying selection |
| Segmental | *MsHAK2* | *MsG0280008140.01.T01* | *GmHAK23* | *Glyma.15G159000* | Ms-Gm | 0.087 | 0.536 | 0.161 | 2355 | 569 | 1786 | Purifying selection |
| Segmental | *MsHAK1* | *MsG0280006462.01.T01* | *GmHAK22* | *Glyma.15G053400* | Ms-Gm | 0.078 | 0.447 | 0.175 | 2481 | 597 | 1884 | Purifying selection |
| Segmental | *MsHAK3* | *MsG0380016724.01.T01* | *GmHAK5* | *Glyma.04G221600* | Ms-Gm | 0.072 | 0.648 | 0.112 | 1707 | 414 | 1293 | Purifying selection |
| Segmental | *MsHAK3* | *MsG0380016724.01.T01* | *GmHAK9* | *Glyma.06G143800* | Ms-Gm | 0.069 | 0.601 | 0.114 | 1707 | 414 | 1293 | Purifying selection |
| Segmental | *MsHAK6* | *MsG0480022802.01.T01* | *GmHAK7* | *Glyma.05G131800* | Ms-Gm | 0.161 | 0.579 | 0.279 | 1230 | 284 | 946 | Purifying selection |
| Segmental | *MsHAK6* | *MsG0480022802.01.T01* | *GmHAK14* | *Glyma.08G086300* | Ms-Gm | 0.169 | 0.583 | 0.290 | 1230 | 286 | 944 | Purifying selection |
| Segmental | *MsHAK8* | *MsG0480022868.01.T01* | *GmHAK15* | *Glyma.08G091900* | Ms-Gm | 0.143 | 0.627 | 0.228 | 1344 | 320 | 1024 | Purifying selection |
| Segmental | *MsHAK6* | *MsG0480022802.01.T01* | *GmHAK19* | *Glyma.09G052700* | Ms-Gm | 0.187 | 0.811 | 0.231 | 1230 | 285 | 945 | Purifying selection |
| Segmental | *MsHAK4* | *MsG0480022271.01.T01* | *GmHAK21* | *Glyma.13G170700* | Ms-Gm | 0.575 | 3.391 | 0.170 | 2061 | 498 | 1563 | Purifying selection |
| Segmental | *MsHAK6* | *MsG0480022802.01.T01* | *GmHAK23* | *Glyma.15G159000* | Ms-Gm | 0.184 | 0.778 | 0.236 | 1230 | 285 | 945 | Purifying selection |
| Segmental | *MsHAK11* | *MsG0580025786.01.T01* | *GmHAK1* | *Glyma.01G031800* | Ms-Gm | 0.117 | 0.937 | 0.124 | 1590 | 389 | 1201 | Purifying selection |
| Segmental | *MsHAK13* | *MsG0580028458.01.T01* | *GmHAK3* | *Glyma.02G228500* | Ms-Gm | 0.127 | 0.460 | 0.275 | 2079 | 483 | 1596 | Purifying selection |
| Segmental | *MsHAK11* | *MsG0580025786.01.T01* | *GmHAK18* | *Glyma.08G288500* | Ms-Gm | 0.130 | 0.779 | 0.167 | 1590 | 388 | 1202 | Purifying selection |
| Segmental | *MsHAK13* | *MsG0580028458.01.T01* | *GmHAK20* | *Glyma.11G169900* | Ms-Gm | 0.171 | 0.652 | 0.263 | 2085 | 484 | 1601 | Purifying selection |
| Segmental | *MsHAK11* | *MsG0580025786.01.T01* | *GmHAK27* | *Glyma.18G136500* | Ms-Gm | 0.122 | 0.877 | 0.139 | 1590 | 385 | 1205 | Purifying selection |
| Segmental | *MsHAK16* | *MsG0680031789.01.T01* | *GmHAK25* | *Glyma.06G148800* | Ms-Gm | 0.325 | 0.802 | 0.405 | 1602 | 374 | 1228 | Purifying selection |
| Segmental | *MsHAK1* | *MsG0280006462.01.T01* | *AtKUP7* | *AT5G09400* | Ms-At | 0.163 | 1.743 | 0.094 | 2484 | 598 | 1886 | Purifying selection |
| Segmental | *MsHAK3* | *MsG0380016724.01.T01* | *AtKUP2* | *AT2G40540* | Ms-At | 0.166 | 2.361 | 0.070 | 1689 | 411 | 1278 | Purifying selection |
| Segmental | *MsHAK11* | *MsG0580025786.01.T01* | *AtKUP6* | *AT1G70300* | Ms-At | 0.163 | 1.758 | 0.093 | 1596 | 390 | 1206 | Purifying selection |
| Segmental | *MsHAK13* | *MsG0580028458.01.T01* | *AtKUP1* | *AT2G30070* | Ms-At | 0.223 | 1.954 | 0.114 | 1962 | 467 | 1495 | Purifying selection |

| Supplementary Table. 3 List of all identified *cis*-acting elements in all *HAK* genes found in *Medicago*. | | | | | | | | | | | | | | | | | | | | |  |  |  |
| --- | --- | --- | --- | --- | --- | --- | --- | --- | --- | --- | --- | --- | --- | --- | --- | --- | --- | --- | --- | --- | --- | --- | --- |
| MsHAK1 | MsG0280006462.01.T01 | 1560,1575,TCA-element | 165,180,TGACG-motif | 165,180,CGTCA-motif | 613,628,LTR | 70,85,ERE | 236,251,ERE | 1712,1727,ERE | 1851,1866,ERE | 257,272,ABRE | 1443,1458,ABRE | 832,847,ARE | 103,118,W box | 266,281,W box | 1731,1746,WUN-motif | 1842,1857,WUN-motif | 1865,1880,WUN-motif | 1916,1931,WUN-motif | 431,446,TGA-element | 1495,1510,TGA-element |  |  |  |
| MsHAK2 | MsG0280008140.01.T01 | 736,751,ABRE | 738,753,ABRE | 739,754,ABRE | 743,758,ABRE | 1493,1508,GARE-motif | 1850,1865,ARE | 569,584,ERE | 1734,1749,WUN-motif | 1814,1829,WUN-motif | 17,32,TGACG-motif | 735,750,TGACG-motif | 17,32,CGTCA-motif | 735,750,CGTCA-motif | 422,437,TC-rich repeats |  |  |  |  |  |  |  |  |
| MsHAK3 | MsG0380016724.01.T01 | 506,521,WUN-motif | 322,337,LTR | 332,347,ARE | 1699,1714,ARE | 1825,1840,ARE | 1929,1944,ARE | 135,150,ABRE | 407,422,ABRE | 1219,1234,ABRE | 1220,1235,ABRE | 1620,1635,ABRE | 92,107,P-box | 690,705,TCA-element |  |  |  |  |  |  |  |  |  |
| MsHAK4 | MsG0480022271.01.T01 | 211,226,TCA-element | 288,303,TCA-element | 601,616,TCA-element | 218,233,TGACG-motif | 218,233,CGTCA-motif | 411,426,TC-rich repeats | 863,878,TC-rich repeats | 1211,1226,ARE | 220,235,ABRE | 1706,1721,LTR | 354,369,MBS | 834,849,ERE | 921,936,ERE | 1693,1708,ERE | 431,446,WUN-motif | 907,922,WUN-motif |  |  |  |  |  |  |
| MsHAK5 | MsG0480022272.01.T01 | 303,318,TC-rich repeats | 755,770,TC-rich repeats | 110,125,CGTCA-motif | 103,118,TCA-element | 180,195,TCA-element | 493,508,TCA-element | 1516,1531,TCA-element | 110,125,TGACG-motif | 1908,1923,TGA-element | 1914,1929,TGA-element | 323,338,WUN-motif | 799,814,WUN-motif | 1103,1118,ARE | 112,127,ABRE | 246,261,MBS | 726,741,ERE | 813,828,ERE | 1583,1598,ERE | 1596,1611,LTR |  |  |  |
| MsHAK6 | MsG0480022802.01.T01 | 24,39,TC-rich repeats | 1819,1834,TC-rich repeats | 916,931,CGTCA-motif | 1575,1590,CGTCA-motif | 690,705,TCA-element | 916,931,TGACG-motif | 1575,1590,TGACG-motif | 280,295,W box | 986,1001,WUN-motif | 411,426,TGA-element | 421,436,TGA-element | 1502,1517,TGA-element | 1258,1273,ERE | 914,929,ABRE | 1685,1700,ARE | 1920,1935,ARE |  |  |  |  |  |  |
| MsHAK7 | MsG0480022804.01.T01 | 31,46,CGTCA-motif | 54,69,TC-rich repeats | 31,46,TGACG-motif | 465,480,W box | 1288,1303,W box | 1329,1344,W box | 1398,1413,WUN-motif | 1399,1414,WUN-motif | 33,48,ABRE | 252,267,ABRE | 836,851,ABRE | 239,254,ARE | 611,626,MBS | 800,815,MBS | 996,1011,MBS | 86,101,LTR |  |  |  |  |  |  |
| MsHAK8 | MsG0480022868.01.T01 | 308,323,TC-rich repeats | 760,775,TC-rich repeats | 116,131,CGTCA-motif | 116,131,TGACG-motif | 109,124,TCA-element | 186,201,TCA-element | 497,512,TCA-element | 1519,1534,TCA-element | 1909,1924,TGA-element | 1915,1930,TGA-element | 328,343,WUN-motif | 804,819,WUN-motif | 118,133,ABRE | 1107,1122,ARE | 731,746,ERE | 818,833,ERE | 1585,1600,ERE | 252,267,MBS | 1598,1613,LTR |  |  |  |
| MsHAK9 | MsG0480022869.01.T01 | 109,124,TCA-element | 186,201,TCA-element | 497,512,TCA-element | 576,591,TCA-element | 1512,1527,TCA-element | 116,131,TGACG-motif | 116,131,CGTCA-motif | 257,272,TC-rich repeats | 308,323,TC-rich repeats | 757,772,TC-rich repeats | 118,133,ABRE | 1252,1267,ABRE | 1101,1116,ARE | 1592,1607,LTR | 729,744,ERE | 812,827,ERE | 1579,1594,ERE | 1908,1923,TGA-element | 1914,1929,TGA-element | 328,343,WUN-motif | 799,814,WUN-motif |  |
| MsHAK10 | MsG0480023156.01.T01 | 173,188,WUN-motif | 1621,1636,WUN-motif | 1861,1876,GARE-motif | 1931,1946,GARE-motif | 267,282,ARE | 381,396,ARE | 424,439,ARE | 1154,1169,ERE | 1286,1301,ERE | 1697,1712,MBS | 1792,1807,MBS | 400,415,TC-rich repeats | 1016,1031,TATC-box | 1967,1982,TATC-box | 87,102,P-box |  |  |  |  |  |  |  |
| MsHAK11 | MsG0580025786.01.T01 | 1113,1128,ARE | 1468,1483,ARE | 1084,1099,ERE | 1038,1053,LTR | 991,1006,TGA-element | 1536,1551,WUN-motif | 1558,1573,WUN-motif | 610,625,TC-rich repeats |  |  |  |  |  |  |  |  |  |  |  |  |  |  |
| MsHAK12 | MsG0580028415.01.T01 | 243,258,CGTCA-motif | 1070,1085,CGTCA-motif | 997,1012,AuxRR-core | 243,258,TGACG-motif | 1070,1085,TGACG-motif | 851,866,TCA-element | 862,877,WUN-motif | 1198,1213,WUN-motif | 249,264,W box | 1414,1429,W box | 202,217,LTR | 1074,1089,ERE | 1708,1723,ERE | 40,55,ARE | 376,391,ARE | 484,499,ARE | 1824,1839,ABRE | 1825,1840,ABRE |  |  |  |  |
| MsHAK13 | MsG0580028458.01.T01 | 1145,1160,MBS | 582,597,ARE | 563,578,WUN-motif | 1301,1316,WUN-motif | 1401,1416,WUN-motif | 573,588,W box | 1520,1535,W box | 1531,1546,W box | 678,693,TCA-element |  |  |  |  |  |  |  |  |  |  |  |  |  |
| MsHAK14 | MsG0580029633.01.T01 | 929,944,TC-rich repeats | 1530,1545,TC-rich repeats | 209,224,CGTCA-motif | 209,224,TGACG-motif | 232,247,TCA-element | 533,548,WUN-motif | 1443,1458,WUN-motif | 32,47,W box | 543,558,W box | 456,471,ERE | 514,529,ARE | 552,567,ARE |  |  |  |  |  |  |  |  |  |  |
| MsHAK15 | MsG0680031788.01.T01 | 62,77,ABRE | 369,384,ABRE | 782,797,ABRE | 1155,1170,ARE | 1578,1593,LTR | 463,478,ERE | 557,572,CGTCA-motif | 780,795,CGTCA-motif | 982,997,CGTCA-motif | 1825,1840,CGTCA-motif | 361,376,TC-rich repeats | 557,572,TGACG-motif | 780,795,TGACG-motif | 982,997,TGACG-motif | 1825,1840,TGACG-motif | 1910,1925,P-box |  |  |  |  |  |  |
| MsHAK16 | MsG0680031789.01.T01 | 91,106,WUN-motif | 1006,1021,WUN-motif | 1491,1506,WUN-motif | 1515,1530,WUN-motif | 1531,1546,W box | 1705,1720,W box | 1746,1761,W box | 1394,1409,TGA-element | 632,647,MBS | 989,1004,LTR | 999,1014,ARE | 801,816,GARE-motif | 1121,1136,GARE-motif | 202,217,CGTCA-motif | 798,813,CGTCA-motif | 788,803,AuxRR-core | 61,76,P-box | 202,217,TGACG-motif | 798,813,TGACG-motif |  |  |  |
| MsHAK17 | MsG0780041281.01.T01 | 621,636,P-box | 1614,1629,TGACG-motif | 1702,1717,TGACG-motif | 1614,1629,CGTCA-motif | 1702,1717,CGTCA-motif | 548,563,ERE | 19,34,ABRE | 890,905,ABRE | 739,754,ARE | 829,844,ARE | 134,149,WUN-motif | 642,657,TGA-element | 686,701,TGA-element | 692,707,TGA-element | 1562,1577,TGA-element |  |  |  |  |  |  |  |
| MsHAK18 | MsG0880042781.01.T01 | 1815,1830,AuxRR-core | 861,876,TGACG-motif | 1984,1999,TGACG-motif | 861,876,CGTCA-motif | 1984,1999,CGTCA-motif | 1323,1338,TC-rich repeats | 1543,1558,TC-rich repeats | 32,47,ERE | 1511,1526,MBS | 584,599,ARE | 1492,1507,ARE | 809,824,ABRE | 810,825,ABRE | 992,1007,W box | 1067,1082,W box | 1163,1178,W box |  |  |  |  |  |  |
| MsHAK19 | MsG0880042786.01.T01 | 1441,1456,ERE | 1783,1798,ERE | 727,742,ARE | 1718,1733,ARE | 164,179,ABRE | 535,550,ABRE | 537,552,ABRE | 627,642,ABRE | 864,879,WUN-motif | 1661,1676,TGACG-motif | 5,20,TC-rich repeats | 1661,1676,CGTCA-motif |  |  |  |  |  |  |  |  |  |  |
| MsHAK20 | MsG0880042787.01.T01 | 51,66,CGTCA-motif | 85,100,CGTCA-motif | 1368,1383,CGTCA-motif | 51,66,TGACG-motif | 85,100,TGACG-motif | 1368,1383,TGACG-motif | 333,348,W box | 326,341,WUN-motif | 7,22,ABRE | 1610,1625,ABRE | 1718,1733,ARE | 1784,1799,ERE |  |  |  |  |  |  |  |  |  |  |
| MsHAK21 | MsG0880042817.01.T01 | 1353,1368,P-box | 187,202,TCA-element | 388,403,TCA-element | 403,418,TC-rich repeats | 1970,1985,ERE | 1972,1987,ERE | 1983,1998,ERE | 19,34,MBS | 1523,1538,ABRE | 318,333,W box | 1434,1449,W box |  |  |  |  |  |  |  |  |  |  |  |
| MsHAK22 | MsG0880045041.01.T01 | 186,201,TC-rich repeats | 24,39,CGTCA-motif | 1560,1575,CGTCA-motif | 24,39,TGACG-motif | 1560,1575,TGACG-motif | 738,753,TCA-element | 38,53,P-box | 910,925,TGA-element | 1642,1657,TGA-element | 415,430,W box | 456,471,W box | 557,572,W box | 1015,1030,ARE | 1203,1218,ARE | 1733,1748,ARE | 1488,1503,ABRE | 1489,1504,ABRE | 1595,1610,ABRE | 673,688,ERE | 1675,1690,ERE | 529,544,MBS |  |
| MtHAK1 | MtrunA17_Chr2g0279131 | 1516,1531,TGA-element | 1786,1801,WUN-motif | 1846,1861,WUN-motif | 1869,1884,WUN-motif | 1914,1929,WUN-motif | 769,784,W box | 626,641,ARE | 682,697,ARE | 1037,1052,ARE | 1240,1255,ARE | 531,546,ABRE | 532,547,ABRE | 1296,1311,ABRE | 1324,1339,ABRE | 1344,1359,ABRE | 1406,1421,ABRE | 1462,1477,ABRE | 1658,1673,ABRE | 1595,1610,ERE | 1855,1870,ERE | 297,312,P-box | 693,708,P-box |
| MtHAK2 | MtrunA17_Chr2g0297291 | 1797,1812,WUN-motif | 1569,1584,MBS | 323,338,ERE | 502,517,ABRE | 504,519,ABRE | 505,520,ABRE | 509,524,ABRE | 501,516,CGTCA-motif | 501,516,TGACG-motif |  |  |  |  |  |  |  |  |  |  |  |  |  |
| MtHAK3 | MtrunA17_Chr2g0297311 | 898,913,W box | 1440,1455,TGA-element | 231,246,MBS | 1741,1756,ERE | 642,657,ARE | 517,532,ABRE | 991,1006,ABRE | 1249,1264,ABRE | 1250,1265,ABRE | 1253,1268,CGTCA-motif | 1253,1268,TGACG-motif |  |  |  |  |  |  |  |  |  |  |  |
| MtHAK4 | MtrunA17_Chr3g0128831 | 146,161,W box | 166,181,W box | 979,994,ARE | 1692,1707,ARE | 1813,1828,ARE | 868,883,ABRE | 1227,1242,ABRE | 1228,1243,ABRE | 1617,1632,ABRE |  |  |  |  |  |  |  |  |  |  |  |  |  |
| MtHAK5 | MtrunA17_Chr4g0052401 | 869,884,MBS | 431,446,ARE | 630,645,ARE | 783,798,ARE | 543,558,WUN-motif | 569,584,WUN-motif | 1261,1276,TGA-element | 302,317,P-box | 1424,1439,TCA-element | 121,136,TC-rich repeats | 1372,1387,TC-rich repeats |  |  |  |  |  |  |  |  |  |  |  |
| MtHAK6 | MtrunA17_Chr4g0055471 | 1674,1689,ARE | 1805,1820,ARE | 599,614,ABRE | 1346,1361,ABRE | 1614,1629,ABRE | 318,333,LTR | 167,182,ERE | 1457,1472,WUN-motif | 239,254,W box | 963,978,W box | 295,310,TGACG-motif | 1181,1196,TGACG-motif | 1665,1680,TGACG-motif | 1467,1482,TCA-element | 1584,1599,P-box | 295,310,CGTCA-motif | 1181,1196,CGTCA-motif | 1665,1680,CGTCA-motif |  |  |  |  |
| MtHAK7 | MtrunA17_Chr5g0413721 | 223,238,CGTCA-motif | 223,238,TGACG-motif | 1420,1435,TCA-element | 1961,1976,TCA-element | 1744,1759,WUN-motif | 1653,1668,W box | 1056,1071,TGA-element | 1509,1524,MBS | 1894,1909,MBS |  |  |  |  |  |  |  |  |  |  |  |  |  |
| MtHAK8 | MtrunA17_Chr5g0413751 | 1455,1470,CGTCA-motif | 1523,1538,AuxRR-core | 1122,1137,P-box | 1365,1380,P-box | 1455,1470,TGACG-motif | 1966,1981,W box | 29,44,WUN-motif | 280,295,WUN-motif | 1104,1119,WUN-motif | 1609,1624,ERE | 1489,1504,ABRE |  |  |  |  |  |  |  |  |  |  |  |
| MtHAK9 | MtrunA17_Chr5g0429991 | 407,422,ERE | 1470,1485,MBS | 1833,1848,ABRE | 1834,1849,ABRE | 403,418,CGTCA-motif | 1114,1129,CGTCA-motif | 403,418,TGACG-motif | 1114,1129,TGACG-motif |  |  |  |  |  |  |  |  |  |  |  |  |  |  |
| MtHAK10 | MtrunA17_Chr5g0430551 | 1121,1136,TGA-element | 1924,1939,WUN-motif | 787,802,W box | 1760,1775,ARE | 1584,1599,ABRE | 1603,1618,ERE | 510,525,CGTCA-motif | 1083,1098,TC-rich repeats | 510,525,TGACG-motif |  |  |  |  |  |  |  |  |  |  |  |  |  |
| MtHAK11 | MtrunA17_Chr5g0430731 | 837,852,W box | 1508,1523,W box | 827,842,WUN-motif | 389,404,ABRE | 390,405,ABRE | 846,861,ARE | 750,765,ERE | 977,992,ERE | 98,113,MBS | 510,525,CGTCA-motif | 510,525,TGACG-motif | 533,548,TCA-element | 911,926,TCA-element |  |  |  |  |  |  |  |  |  |
| MtHAK12 | MtrunA17_Chr6g0451081 | 1661,1676,TC-rich repeats | 954,969,CGTCA-motif | 954,969,TGACG-motif | 1183,1198,P-box | 1124,1139,ARE | 1460,1475,ARE | 1963,1978,ARE | 1889,1904,LTR |  |  |  |  |  |  |  |  |  |  |  |  |  |  |
| MtHAK13 | MtrunA17_Chr6g0461871 | 122,137,W box | 271,286,W box | 846,861,W box | 1542,1557,W box | 1718,1733,W box | 1759,1774,W box | 1501,1516,WUN-motif | 18,33,ABRE | 1514,1529,ARE | 1986,2001,ARE | 1412,1427,ERE | 1414,1429,ERE | 1482,1497,MBS | 1048,1063,CGTCA-motif | 90,105,TC-rich repeats | 1048,1063,TGACG-motif | 427,442,TCA-element |  |  |  |  |  |
| MtHAK14 | MtrunA17_Chr7g0268981 | 327,342,TCA-element | 1080,1095,TGACG-motif | 1222,1237,TGACG-motif | 1080,1095,CGTCA-motif | 1222,1237,CGTCA-motif | 865,880,MBS | 96,111,ERE | 630,645,ARE | 869,884,ARE | 1168,1183,ABRE | 1844,1859,ABRE |  |  |  |  |  |  |  |  |  |  |  |
| MtHAK15 | MtrunA17_Chr8g0344551 | 4,19,ARE | 1618,1633,ABRE | 1030,1045,W box | 1540,1555,W box | 1865,1880,W box | 350,365,TGA-element | 934,949,TCA-element | 1850,1865,TCA-element |  |  |  |  |  |  |  |  |  |  |  |  |  |  |
| MtHAK16 | MtrunA17_Chr8g0344581 | 955,970,W box | 1473,1488,W box | 1865,1880,W box | 1691,1706,WUN-motif | 144,159,TGA-element | 1572,1587,MBS | 1703,1718,ABRE | 856,871,ARE | 1850,1865,TCA-element |  |  |  |  |  |  |  |  |  |  |  |  |  |
| MtHAK17 | MtrunA17_Chr8g0365001 | 159,174,CGTCA-motif | 675,690,CGTCA-motif | 159,174,TGACG-motif | 675,690,TGACG-motif | 797,812,W box | 1209,1224,W box | 215,230,ERE | 775,790,ERE | 1019,1034,ERE | 1491,1506,ERE | 110,125,ABRE | 673,688,ABRE | 678,693,ABRE | 679,694,ABRE | 811,826,ARE | 1920,1935,ARE |  |  |  |  |  |  |
| MtHAK18 | MtrunA17_Chr8g0365041 | 1082,1097,W box | 574,589,WUN-motif | 1774,1789,TGA-element | 1684,1699,LTR | 1723,1738,LTR | 472,487,ERE | 925,940,ERE | 1156,1171,ERE | 269,284,ABRE | 270,285,ABRE | 379,394,ABRE | 380,395,ABRE | 1556,1571,ABRE | 316,331,ARE | 393,408,ARE | 792,807,ARE | 1527,1542,CGTCA-motif | 1527,1542,TGACG-motif |  |  |  |  |
| MtHAK19 | MtrunA17_Chr8g0375411 | 508,523,AuxRR-core | 126,141,TCA-element | 308,323,TCA-element | 633,648,TCA-element | 228,243,TC-rich repeats | 1184,1199,ERE | 864,879,LTR | 481,496,ARE | 572,587,ABRE | 1076,1091,WUN-motif | 1235,1250,WUN-motif | 1605,1620,WUN-motif | 1693,1708,WUN-motif | 63,78,W box | 102,117,W box |  |  |  |  |  |  |  |
| MtHAK20 | MtrunA17_Chr8g0380281 | 1606,1621,ERE | 1864,1879,ERE | 1437,1452,LTR | 1628,1643,ARE | 282,297,ABRE | 1201,1216,ABRE | 1854,1869,ABRE | 1390,1405,W box | 1512,1527,W box | 1451,1466,TATC-box | 560,575,TC-rich repeats |  |  |  |  |  |  |  |  |  |  |  |
| MtHAK21 | MtrunA17_Chr8g0387661 | 1357,1372,TCA-element | 1613,1628,TCA-element | 100,115,TGACG-motif | 188,203,TGACG-motif | 232,247,TGACG-motif | 1112,1127,TC-rich repeats | 100,115,CGTCA-motif | 188,203,CGTCA-motif | 232,247,CGTCA-motif | 224,239,ABRE | 1087,1102,ABRE | 1229,1244,MBS | 606,621,ERE | 937,952,ERE | 939,954,ERE | 1677,1692,WUN-motif |  |  |  |  |  |  |
| MtHAK22 | MtrunA17_Chr8g0393241 | 465,480,TGACG-motif | 1231,1246,TGACG-motif | 465,480,CGTCA-motif | 1231,1246,CGTCA-motif | 1108,1123,ARE | 1154,1169,ARE | 250,265,ABRE | 252,267,ABRE | 1189,1204,ABRE | 1191,1206,ABRE | 1192,1207,ABRE | 1437,1452,ABRE | 1570,1585,ABRE | 1675,1690,ABRE | 124,139,ERE |  |  |  |  |  |  |  |

| Supplementary Table 4-1. Detailed information on the available tissue expression levels of MtHAK genes retrieved from microarray data for *M. truncatula.* | | | | | | | |
| --- | --- | --- | --- | --- | --- | --- | --- |
| Probeset | Gene ID | Root | Stem | Leaf | Flower | Pod | Seed |
| Mtr.9321.1.S1_at | MtHAK2 | 485.0806667 | 199.7836667 | 860.691 | 274.795 | 225.921 | 172.2386667 |
| Mtr.41449.1.S1_at | MtHAK3 | 1368.776667 | 533.8563333 | 1748.78 | 870.418 | 596.058 | 629.211 |
| Mtr.10999.1.S1_at | MtHAK4 | 1672.726667 | 1162.64 | 1403.84 | 1644.946667 | 1888.033333 | 1781.583333 |
| Mtr.3419.1.S1_at | MtHAK5 | 155.936 | 409.3773333 | 741.1726667 | 395.9106667 | 820.7536667 | 4577.44 |
| Mtr.40509.1.S1_at | MtHAK6 | 5740.486667 | 22.78346667 | 9.09233 | 8.125513333 | 8.146866667 | 8.29617 |
| Mtr.34174.1.S1_at | MtHAK7 | 247.0936667 | 159.5193333 | 302.6753333 | 152.7266667 | 91.2839 | 194.445 |
| Mtr.18323.1.S1_at | MtHAK9 | 111.423 | 173.8213333 | 123.2886667 | 124.2683333 | 195.531 | 276.4176667 |
| Mtr.1970.1.S1_at | MtHAK10 | 18.66666667 | 23.03066667 | 23.82836667 | 30.96076667 | 18.93426667 | 24.41336667 |
| Mtr.1970.1.S1_at | MtHAK11 | 18.66666667 | 23.03066667 | 23.82836667 | 30.96076667 | 18.93426667 | 24.41336667 |
| Mtr.40771.1.S1_at | MtHAK12 | 801.432 | 585.418 | 1533.506667 | 808.8513333 | 456.5803333 | 3731.813333 |
| Mtr.42438.1.S1_at | MtHAK13 | 11.96363333 | 9.12426 | 13.2957 | 13.7233 | 9.450976667 | 20.3918 |
| Mtr.46517.1.S1_at | MtHAK15 | 7.583283333 | 7.316543333 | 8.351196667 | 38.0859 | 47.76683333 | 104.7089667 |
| Mtr.46518.1.S1_s_at | MtHAK16 | 8.239573333 | 9.496726667 | 12.3654 | 23.29886667 | 37.724 | 47.42996667 |
| Mtr.28473.1.S1_at | MtHAK17 | 236.8293333 | 135.701 | 165.233 | 94.50706667 | 151.7666667 | 74.60506667 |
| Mtr.24768.1.S1_s_at | MtHAK20 | 10.09062 | 11.78207667 | 12.77746667 | 12.47693333 | 12.4589 | 12.9986 |
| Mtr.45408.1.S1_at | MtHAK21 | 81.35013333 | 64.73303333 | 51.72356667 | 25.85066667 | 50.78206667 | 51.49683333 |

| Supplementary Table 4-2. Detailed information on the available tissue expression levels of *MsHAK* genes retrieved from transcriptome data for *M. sativa* | | | | | | | |
| --- | --- | --- | --- | --- | --- | --- | --- |
| ContigID | Gene ID | Root | E_Stem | PE_Stem | Leaf | Flower | Nodule |
| contig_64495 | MsHAK1 | 4 | 5 | 3 | 3 | 3 | 3 |
| contig_7563 | MsHAK2 | 8 | 7 | 6 | 21 | 7 | 2 |
| contig_111178 | MsHAK3 | 8 | 9 | 8 | 4 | 10 | 4 |
| contig_2139 | MsHAK4/5/8/9 | 4 | 4 | 3 | 2 | 2 | 7 |
| contig_14405 | MsHAK6/7 | 1 | 3 | 2 | 4 | 2 | 0 |
| contig_93843 | MsHAK10 | 19 | 21 | 7 | 6 | 12 | 11 |
| contig_38346 | MsHAK11 | 2 | 1 | 1 | 1 | 1 | 20 |
| contig_55648 | MsHAK12 | 6 | 6 | 4 | 3 | 4 | 4 |
| contig_71175 | MsHAK13 | 0 | 1 | 0 | 0 | 2 | 0 |
| contig_79772 | MsHAK14 | 0 | 0 | 0 | 0 | 1 | 0 |
| contig_54633 | MsHAK15/16 | 9 | 7 | 6 | 16 | 23 | 5 |
| contig_35614 | MsHAK17/22 | 0 | 0 | 0 | 3 | 0 | 0 |
| contig_15516 | MsHAK20 | 62 | 0 | 0 | 0 | 0 | 9 |

| Supplementary Table 4-3. Detailed information on the available expression levels of *MsHAK* genes response to salinity stress treatments retrieved from microarray data for *M. truncatula.* | | | | | | | | | | | |
| --- | --- | --- | --- | --- | --- | --- | --- | --- | --- | --- | --- |
| Probeset | Gene ID | vitro_NaCl_0h | vitro_NaCl_6h | vitro_NaCl_24h | vitro_NaCl_48 | hydroponics_NaCl_0h | hydroponics_NaCl_1h | hydroponics_NaCl_2h | hydroponics_NaCl_5h | hydroponics_NaCl_10h | hydroponics_NaCl_24h |
| Mtr.9321.1.S1_at | MtHAK2 | 822.3716667 | 1112.980667 | 2013.24 | 1894.043333 | 325.936 | 381.591 | 386.046 | 425.871 | 475.581 | 691.021 |
| Mtr.41449.1.S1_at | MtHAK3 | 1566.24 | 1941.82 | 3065.323333 | 2863.296667 | 982.081 | 1001.44 | 1077.6 | 1241.75 | 1484.57 | 1850.79 |
| Mtr.10999.1.S1_at | MtHAK4 | 1712.74 | 2262.723333 | 2387.673333 | 1805.653333 | 1723.92 | 1139.54 | 1424.95 | 1507.56 | 1424.21 | 1835.63 |
| Mtr.3419.1.S1_at | MtHAK5 | 182.4106667 | 413.9096667 | 301.7416667 | 272.9803333 | 159.153 | 151.186 | 112.146 | 178.232 | 194.686 | 221.269 |
| Mtr.40509.1.S1_at | MtHAK6 | 1152.644 | 340.822 | 766.9276667 | 437.623 | 5772.03 | 3809.36 | 4786.36 | 4051.66 | 4868.27 | 3121.07 |
| Mtr.34174.1.S1_at | MtHAK7 | 312.3053333 | 470.994 | 1049.316 | 1235.993667 | 291.624 | 287.656 | 540.233 | 838.598 | 741.663 | 884.806 |
| Mtr.18323.1.S1_at | MtHAK9 | 183.6516667 | 166.611 | 180.2303333 | 145.1281667 | 122.273 | 153.381 | 106.904 | 104.399 | 108.502 | 101.332 |
| Mtr.1970.1.S1_at | MtHAK10 | 28.13573333 | 28.6147 | 31.33766667 | 28.68766667 | 20.7012 | 18.0105 | 18.9727 | 17.8962 | 16.3697 | 18.4708 |
| Mtr.1970.1.S1_at | MtHAK11 | 28.13573333 | 28.6147 | 31.33766667 | 28.68766667 | 20.7012 | 18.0105 | 18.9727 | 17.8962 | 16.3697 | 18.4708 |
| Mtr.40771.1.S1_at | MtHAK12 | 645.1976667 | 874.8666667 | 1109.805667 | 1618.503333 | 451.126 | 369.299 | 912.221 | 808.793 | 929.019 | 1253.5 |
| Mtr.42438.1.S1_at | MtHAK13 | 51.96046667 | 84.17793333 | 105.1884 | 122.7649667 | 8.91799 | 10.3616 | 8.67703 | 11.1978 | 11.3625 | 8.69199 |
| Mtr.46517.1.S1_at | MtHAK15 | 8.3408 | 9.79893 | 13.12893333 | 21.53703333 | 7.45687 | 7.89982 | 8.08197 | 10.1665 | 7.8981 | 7.7233 |
| Mtr.46518.1.S1_s_at | MtHAK16 | 10.8044 | 10.6522 | 11.82687667 | 14.53 | 7.84781 | 7.91795 | 8.99963 | 7.82804 | 8.97189 | 10.543 |
| Mtr.28473.1.S1_at | MtHAK17 | 319.9193333 | 191.8733333 | 238.2703333 | 183.7103333 | 193.249 | 167.294 | 141.014 | 178.99 | 148.547 | 123.337 |
| Mtr.24768.1.S1_s_at | MtHAK20 | 12.63543333 | 10.66380667 | 9.849153333 | 10.19923333 | 10.3789 | 10.5899 | 10.4054 | 8.56921 | 8.13947 | 9.18388 |
| Mtr.45408.1.S1_at | MtHAK21 | 129.9252333 | 94.01566667 | 187.0153333 | 169.5993667 | 110.501 | 93.4638 | 94.1324 | 78.9843 | 106.44 | 105.446 |

| Supplementary Table 4. Detailed information on the available expression levels of *MsHAK* genes response to drought stress treatments retrieved from microarray data for *M. truncatula*. | | | | | | | | | | | | | | | | | |
| --- | --- | --- | --- | --- | --- | --- | --- | --- | --- | --- | --- | --- | --- | --- | --- | --- | --- |
| Probeset | Gene ID | RT_w_d2 | RT_d_2d | RT_d_3d | RT_d_4d | RT_d_7d | RT_d_10d | RT_d_14d | RT_d_14d_1d | SHT_w_2d | SHT_d_2d | SHT_d_3d | SHT_d_4d | SHT_d_7d | SHT_d_10d | SHT_d_14d | SHT_d_14d_1d |
| Mtr.9321.1.S1_at | MtHAK2 | 476.543 | 582.5436667 | 462.3173333 | 408.6313333 | 261.0126667 | 280.6983333 | 304.5583333 | 498.8373333 | 522.2496667 | 508.494 | 535.3976667 | 880.5476667 | 583.63 | 530.1176667 | 498.9783333 | 431.416 |
| Mtr.41449.1.S1_at | MtHAK3 | 1315.303333 | 1444.603333 | 1145.466667 | 1174.18 | 736.1956667 | 786.128 | 832.8933333 | 1331.45 | 1130.916667 | 1067.865 | 1202.106667 | 1885.996667 | 1304.813333 | 1293.8 | 1173.996667 | 968.129 |
| Mtr.10999.1.S1_at | MtHAK4 | 1496.02 | 1349.813333 | 1009.112333 | 891.5193333 | 801.4916667 | 627.768 | 482.7023333 | 2059.003333 | 1154.06 | 1119.586667 | 978.8816667 | 598.211 | 755.6853333 | 597.664 | 531.324 | 1354.613333 |
| Mtr.3419.1.S1_at | MtHAK5 | 229.955 | 249.0863333 | 227.6823333 | 189.4323333 | 248.8503333 | 295.08 | 351.785 | 245.393 | 621.7963333 | 610.8863333 | 579.8983333 | 672.6343333 | 815.7736667 | 870.8693333 | 863.1266667 | 774.7413333 |
| Mtr.40509.1.S1_at | MtHAK6 | 3358.383333 | 5615.26 | 5668.366667 | 3532.066667 | 2308.153333 | 2258.436667 | 791.288 | 4266.516667 | 10.70693 | 12.259 | 10.36476667 | 8.860723333 | 7.669033333 | 8.92676 | 8.803136667 | 10.23471667 |
| Mtr.34174.1.S1_at | MtHAK7 | 301.507 | 426.3303333 | 702.8283333 | 729.2736667 | 568.8153333 | 565.476 | 687.4993333 | 290.005 | 185.0573333 | 214.331 | 209.416 | 259.781 | 277.396 | 299.4556667 | 273.3096667 | 219.103 |
| Mtr.18323.1.S1_at | MtHAK9 | 169.854 | 161.2533333 | 182.876 | 229.2366667 | 285.6776667 | 339.949 | 430.6593333 | 156.0516667 | 116.3281333 | 128.424 | 140.6976667 | 193.057 | 217.005 | 273.307 | 298.5296667 | 148.6333333 |
| Mtr.1970.1.S1_at | MtHAK10 | 24.00646667 | 15.6081 | 16.26343333 | 18.5429 | 16.12196667 | 16.35723333 | 18.32643333 | 20.7338 | 20.5321 | 21.4839 | 20.5262 | 17.8218 | 17.2644 | 18.42343333 | 20.06233333 | 21.44583333 |
| Mtr.1970.1.S1_at | MtHAK11 | 24.00646667 | 15.6081 | 16.26343333 | 18.5429 | 16.12196667 | 16.35723333 | 18.32643333 | 20.7338 | 20.5321 | 21.4839 | 20.5262 | 17.8218 | 17.2644 | 18.42343333 | 20.06233333 | 21.44583333 |
| Mtr.40771.1.S1_at | MtHAK12 | 810.669 | 1089.516667 | 1180.77 | 1891.713333 | 2248.176667 | 2359 | 2886.873333 | 595.3283333 | 1074.016667 | 1129.91 | 1341.946667 | 2235.793333 | 2201.603333 | 2887.27 | 2983.076667 | 1055.356667 |
| Mtr.42438.1.S1_at | MtHAK13 | 9.4055 | 9.27824 | 10.31524333 | 10.32373 | 12.64826667 | 10.74966667 | 13.3028 | 9.70581 | 11.84256667 | 11.1267 | 10.87279333 | 13.38086667 | 11.67153333 | 10.56386667 | 10.98859 | 12.03346667 |
| Mtr.46517.1.S1_at | MtHAK15 | 7.236866667 | 7.446976667 | 7.79458 | 8.028193333 | 7.43416 | 7.79044 | 7.24512 | 7.90983 | 7.540926667 | 9.062363333 | 8.43492 | 7.846913333 | 8.487453333 | 8.438353333 | 9.420076667 | 9.091423333 |
| Mtr.46518.1.S1_s_at | MtHAK16 | 8.492006667 | 8.003436667 | 8.575303333 | 8.361143333 | 8.313663333 | 8.651426667 | 8.12621 | 8.60917 | 9.681083333 | 11.01306667 | 11.89469333 | 11.66996667 | 12.4137 | 10.59176 | 10.09627667 | 12.88953333 |
| Mtr.28473.1.S1_at | MtHAK17 | 231.7106667 | 225.3806667 | 224.0186667 | 143.016 | 101.8882333 | 101.9508 | 75.8434 | 319.3186667 | 152.5046667 | 160.1116667 | 137.2366667 | 95.9061 | 103.1119333 | 95.4751 | 93.57916667 | 183.6926667 |
| Mtr.24768.1.S1_s_at | MtHAK20 | 9.967993333 | 10.29548667 | 10.14134333 | 10.79374667 | 9.697276667 | 10.35753333 | 13.20553333 | 9.70801 | 10.9178 | 10.48858333 | 11.797 | 10.87316667 | 11.16870667 | 11.18376333 | 11.02683333 | 10.39264333 |
| Mtr.45408.1.S1_at | MtHAK21 | 122.251 | 96.53376667 | 80.22376667 | 108.3087333 | 106.4884333 | 118.733 | 117.8328333 | 103.7820667 | 49.30593333 | 70.3306 | 72.49136667 | 99.087 | 94.14596667 | 119.8936667 | 120.8123333 | 72.20836667 |

| Supplementary Table 4-5. Detailed information on the available expression levels of *MsHAK* genes response to salinity stress treatment retrieved from transcriptome data for *M. sativa* | | | | | | |
| --- | --- | --- | --- | --- | --- | --- |
| Gene ID | CK | S_1h | S_3h | S_6h | S_12h | S_24h |
| MsHAK1 | 20.77653396 | 30.78179437 | 31.70024426 | 44.06362284 | 59.11551596 | 47.06978502 |
| MsHAK2 | 11.96854504 | 24.91891986 | 22.72704921 | 31.91237056 | 36.53085376 | 33.87202307 |
| MsHAK3 | 37.78753519 | 32.20155392 | 42.0880412 | 64.81863429 | 73.17707861 | 96.95989121 |
| MsHAK4 | 0.406818409 | 0.265369612 | 0.476414646 | 0.130265324 | 0.151958659 | 0 |
| MsHAK5 | 4.371320732 | 6.33916019 | 4.360796844 | 3.880469368 | 3.276811411 | 2.147263407 |
| MsHAK6 | 2.100850281 | 3.197651241 | 4.575809337 | 7.291460176 | 5.317419008 | 4.954061429 |
| MsHAK7 | 1.251802185 | 1.691801126 | 2.13047279 | 2.656226397 | 2.551088349 | 0.889445687 |
| MsHAK8 | 0.082761324 | 0.121391789 | 0 | 0.104950154 | 0.23562757 | 0 |
| MsHAK9 | 4.371320732 | 6.33916019 | 4.360796844 | 3.880469368 | 3.276811411 | 2.147263407 |
| MsHAK10 | 139.1054627 | 131.1553141 | 133.2680623 | 141.3548295 | 142.5236168 | 192.8123468 |
| MsHAK11 | 9.427238116 | 13.34343055 | 15.19047009 | 19.64548245 | 12.1878774 | 9.701472468 |
| MsHAK12 | 13.52985912 | 16.16874487 | 17.45802628 | 23.48633565 | 28.97772818 | 20.00111291 |
| MsHAK13 | 1.483772491 | 0.753464515 | 0.736542616 | 0.845047471 | 0.902677061 | 4.349093834 |
| MsHAK14 | 1.040511771 | 0.70063993 | 0.637859201 | 0.633568964 | 0.676305664 | 3.084480134 |
| MsHAK15 | 2.65632871 | 4.247745187 | 3.624298185 | 4.607198255 | 3.877242199 | 3.811573915 |
| MsHAK16 | 10.40224497 | 11.2213614 | 9.275197807 | 14.95508693 | 11.88674632 | 9.931086894 |
| MsHAK17 | 0.040550272 | 0.070540674 | 0.020094937 | 0.041438664 | 0.064132618 | 0.041784061 |
| MsHAK18 | 0 | 0 | 0 | 0.038214482 | 0 | 0 |
| MsHAK19 | 0 | 0.033297874 | 0 | 0 | 0 | 0 |
| MsHAK20 | 0.115711218 | 0.045157248 | 0.081232996 | 0 | 0 | 0 |
| MsHAK21 | 0 | 0 | 0 | 0.027947905 | 0.026402896 | 0 |
| MsHAK22 | 0.013348446 | 0 | 0 | 0 | 0 | 0 |
| Supplementary Table 4-6. Detailed information on the available expression levels of MsHAK genes response to drought stress treatment retrieved from transcriptome data for *M. sativa* | | | | | | |
| Gene ID | CK | D_1h | D_3h | D_6h | D_12h | D_24h |
| MsHAK1 | 20.77653396 | 26.75134935 | 31.16429406 | 45.41994639 | 52.26585209 | 50.57134412 |
| MsHAK2 | 11.96854504 | 26.72454473 | 21.59941461 | 24.39838737 | 28.77982557 | 25.16355474 |
| MsHAK3 | 37.78753519 | 34.0279387 | 46.91709075 | 49.95932294 | 42.98178525 | 40.91957834 |
| MsHAK4 | 0.406818409 | 0.179464369 | 0.127969323 | 0.054709552 | 0.192628426 | 0.130685933 |
| MsHAK5 | 4.371320732 | 5.668862426 | 5.099733297 | 4.374812862 | 4.109929035 | 2.624007885 |
| MsHAK6 | 2.100850281 | 4.292808185 | 6.293538517 | 6.577434825 | 4.871458456 | 3.494206391 |
| MsHAK7 | 1.251802185 | 1.651925987 | 1.927031155 | 2.395181849 | 1.939250359 | 1.162947842 |
| MsHAK8 | 0.082761324 | 0 | 0 | 0.080141429 | 0.084082114 | 0 |
| MsHAK9 | 4.371320732 | 5.668862426 | 5.099733297 | 4.374812862 | 4.109929035 | 2.624007885 |
| MsHAK10 | 139.1054627 | 114.012548 | 121.0189131 | 166.1713352 | 207.8098393 | 295.407953 |
| MsHAK11 | 9.427238116 | 14.44478058 | 18.05587469 | 14.02554304 | 7.417373176 | 9.216335869 |
| MsHAK12 | 13.52985912 | 17.14929371 | 17.1551288 | 23.87741029 | 19.15078867 | 16.29831291 |
| MsHAK13 | 1.483772491 | 0.855076368 | 0.726936508 | 1.075732942 | 0.647185443 | 1.276252504 |
| MsHAK14 | 1.040511771 | 0.572296744 | 0.542236052 | 1.078866544 | 0.620875449 | 1.048552588 |
| MsHAK15 | 2.65632871 | 3.968864953 | 4.531955487 | 5.095230929 | 3.045024249 | 3.756034795 |
| MsHAK16 | 10.40224497 | 13.01322262 | 9.675193603 | 14.03095795 | 9.746858342 | 9.440324094 |
| MsHAK17 | 0.040550272 | 0.098591239 | 0.058378394 | 0.062552906 | 0.085522502 | 0.22751002 |
| MsHAK18 | 0.01 | 0.037456293 | 0 | 0 | 0 | 0 |
| MsHAK19 | 0.01 | 0 | 0.035300787 | 0 | 0 | 0 |
| MsHAK20 | 0.115711218 | 0.029913336 | 0.034919222 | 0 | 0.039195087 | 0.040176357 |
| MsHAK21 | 0.01 | 0 | 0 | 0 | 0 | 0 |
| MsHAK22 | 0.013348446 | 0.010681358 | 0.014107252 | 0.014684989 | 0.013995656 | 0 |

|  | Supplementary Table 5-1. The co-expression network of *MtHAK* genes in DEGs under salt stress. | | |
| --- | --- | --- | --- |
|  | fromNode | toNode | weight |
| 1 | Mtr.40771.1.S1_at | Mtr.47465.1.S1_at | 0.100188456 |
| 2 | Mtr.40771.1.S1_at | Mtr.10316.1.S1_at | 0.110478689 |
| 3 | Mtr.40771.1.S1_at | Mtr.10478.1.S1_at | 0.136748567 |
| 4 | Mtr.40771.1.S1_at | Mtr.10503.1.S1_at | 0.100567878 |
| 5 | Mtr.40771.1.S1_at | Mtr.10884.1.S1_at | 0.103073564 |
| 6 | Mtr.40771.1.S1_at | Mtr.12254.1.S1_x_at | 0.143937229 |
| 7 | Mtr.40771.1.S1_at | Mtr.12369.1.S1_at | 0.137934601 |
| 8 | Mtr.40771.1.S1_at | Mtr.12491.1.S1_at | 0.112446565 |
| 9 | Mtr.40771.1.S1_at | Mtr.12773.1.S1_at | 0.131032889 |
| 10 | Mtr.40771.1.S1_at | Mtr.12973.1.S1_at | 0.118067176 |
| 11 | Mtr.40771.1.S1_at | Mtr.13023.1.S1_at | 0.109188514 |
| 12 | Mtr.40771.1.S1_at | Mtr.14839.1.S1_at | 0.126429781 |
| 13 | Mtr.40771.1.S1_at | Mtr.15486.1.S1_at | 0.136017953 |
| 14 | Mtr.40771.1.S1_at | Mtr.15524.1.S1_at | 0.10835671 |
| 15 | Mtr.40771.1.S1_at | Mtr.16504.1.S1_at | 0.106870692 |
| 16 | Mtr.40771.1.S1_at | Mtr.16553.1.S1_at | 0.102166529 |
| 17 | Mtr.40771.1.S1_at | Mtr.16815.1.S1_at | 0.116741018 |
| 18 | Mtr.40771.1.S1_at | Mtr.17463.1.S1_at | 0.101853682 |
| 19 | Mtr.40771.1.S1_at | Mtr.17795.1.S1_x_at | 0.124085658 |
| 20 | Mtr.40771.1.S1_at | Mtr.18000.1.S1_at | 0.109045968 |
| 21 | Mtr.40771.1.S1_at | Mtr.18244.1.S1_at | 0.117610543 |
| 22 | Mtr.40771.1.S1_at | Mtr.18666.1.S1_at | 0.11470863 |
| 23 | Mtr.40771.1.S1_at | Mtr.19355.1.S1_s_at | 0.1058021 |
| 24 | Mtr.40771.1.S1_at | Mtr.19730.1.S1_s_at | 0.113540319 |
| 25 | Mtr.40771.1.S1_at | Mtr.20483.1.S1_at | 0.113326497 |
| 26 | Mtr.40771.1.S1_at | Mtr.20733.1.S1_at | 0.100949754 |
| 27 | Mtr.40771.1.S1_at | Mtr.20870.1.S1_at | 0.119956136 |
| 28 | Mtr.40771.1.S1_at | Mtr.20870.1.S1_s_at | 0.149739056 |
| 29 | Mtr.40771.1.S1_at | Mtr.21286.1.S1_at | 0.109728588 |
| 30 | Mtr.40771.1.S1_at | Mtr.21477.1.S1_at | 0.105818395 |
| 31 | Mtr.40771.1.S1_at | Mtr.22025.1.S1_at | 0.120665624 |
| 32 | Mtr.40771.1.S1_at | Mtr.22633.1.S1_at | 0.104514608 |
| 33 | Mtr.40771.1.S1_at | Mtr.26361.1.S1_at | 0.112628308 |
| 34 | Mtr.40771.1.S1_at | Mtr.26586.1.S1_at | 0.118323344 |
| 35 | Mtr.40771.1.S1_at | Mtr.27943.1.S1_at | 0.113813508 |
| 36 | Mtr.40771.1.S1_at | Mtr.27955.1.S1_at | 0.108194651 |
| 37 | Mtr.40771.1.S1_at | Mtr.32115.1.S1_at | 0.102601728 |
| 38 | Mtr.40771.1.S1_at | Mtr.32405.1.S1_s_at | 0.100209175 |
| 39 | Mtr.40771.1.S1_at | Mtr.35844.1.S1_at | 0.105223075 |
| 40 | Mtr.40771.1.S1_at | Mtr.37218.1.S1_at | 0.120485636 |
| 41 | Mtr.40771.1.S1_at | Mtr.37360.1.S1_at | 0.113451742 |
| 42 | Mtr.40771.1.S1_at | Mtr.37507.1.S1_at | 0.115553008 |
| 43 | Mtr.40771.1.S1_at | Mtr.37733.1.S1_at | 0.132152641 |
| 44 | Mtr.40771.1.S1_at | Mtr.37954.1.S1_at | 0.123173687 |
| 45 | Mtr.40771.1.S1_at | Mtr.39106.1.S1_at | 0.131863989 |
| 46 | Mtr.40771.1.S1_at | Mtr.39761.1.S1_at | 0.109864733 |
| 47 | Mtr.40771.1.S1_at | Mtr.40019.1.S1_at | 0.102736328 |
| 48 | Mtr.40771.1.S1_at | Mtr.40044.1.S1_at | 0.127397853 |
| 49 | Mtr.40771.1.S1_at | Mtr.40107.1.S1_at | 0.120821476 |
| 50 | Mtr.40771.1.S1_at | Mtr.40170.1.S1_at | 0.115884809 |
| 51 | Mtr.40771.1.S1_at | Mtr.40171.1.S1_at | 0.165531501 |
| 52 | Mtr.40771.1.S1_at | Mtr.40186.1.S1_at | 0.10704866 |
| 53 | Mtr.40771.1.S1_at | Mtr.40200.1.S1_at | 0.107280491 |
| 54 | Mtr.40771.1.S1_at | Mtr.40217.1.S1_at | 0.102778335 |
| 55 | Mtr.40771.1.S1_at | Mtr.40294.1.S1_at | 0.106832662 |
| 56 | Mtr.40771.1.S1_at | Mtr.40317.1.S1_at | 0.105871477 |
| 57 | Mtr.40771.1.S1_at | Mtr.40365.1.S1_s_at | 0.127318452 |
| 58 | Mtr.40771.1.S1_at | Mtr.40878.1.S1_at | 0.106642485 |
| 59 | Mtr.40771.1.S1_at | Mtr.41044.1.S1_at | 0.104981267 |
| 60 | Mtr.40771.1.S1_at | Mtr.42243.1.S1_at | 0.113257029 |
| 61 | Mtr.40771.1.S1_at | Mtr.42637.1.S1_at | 0.10845182 |
| 62 | Mtr.40771.1.S1_at | Mtr.42995.1.S1_at | 0.103659743 |
| 63 | Mtr.40771.1.S1_at | Mtr.43086.1.S1_at | 0.122152287 |
| 64 | Mtr.40771.1.S1_at | Mtr.43092.1.S1_at | 0.125502425 |
| 65 | Mtr.40771.1.S1_at | Mtr.43218.1.S1_at | 0.112958221 |
| 66 | Mtr.40771.1.S1_at | Mtr.43222.1.S1_at | 0.125697946 |
| 67 | Mtr.40771.1.S1_at | Mtr.43226.1.S1_at | 0.111711966 |
| 68 | Mtr.40771.1.S1_at | Mtr.43388.1.S1_at | 0.110417742 |
| 69 | Mtr.40771.1.S1_at | Mtr.43484.1.S1_at | 0.107646634 |
| 70 | Mtr.40771.1.S1_at | Mtr.45862.1.S1_at | 0.13022786 |
| 71 | Mtr.40771.1.S1_at | Mtr.47189.1.S1_at | 0.116484725 |
| 72 | Mtr.40771.1.S1_at | Mtr.47396.1.S1_at | 0.1451741 |
| 73 | Mtr.40771.1.S1_at | Mtr.47646.1.S1_s_at | 0.115192148 |
| 74 | Mtr.40771.1.S1_at | Mtr.48223.1.S1_at | 0.10647167 |
| 75 | Mtr.40771.1.S1_at | Mtr.48856.1.S1_at | 0.156448598 |
| 76 | Mtr.40771.1.S1_at | Mtr.49097.1.S1_at | 0.120806794 |
| 77 | Mtr.40771.1.S1_at | Mtr.607.1.S1_s_at | 0.139910673 |
| 78 | Mtr.40771.1.S1_at | Mtr.8600.1.S1_at | 0.150116705 |
| 79 | Mtr.40771.1.S1_at | Mtr.8610.1.S1_at | 0.148966877 |
| 80 | Mtr.40771.1.S1_at | Mtr.9025.1.S1_at | 0.100354711 |
| 81 | Mtr.40771.1.S1_at | Mtr.9484.1.S1_at | 0.10853934 |
| 82 | Mtr.42438.1.S1_at | Mtr.42464.1.S1_at | 0.202610124 |
| 83 | Mtr.42438.1.S1_at | Mtr.43030.1.S1_at | 0.127066486 |
| 84 | Mtr.42438.1.S1_at | Mtr.44108.1.S1_at | 0.130083642 |
| 85 | Mtr.42438.1.S1_at | Mtr.44312.1.S1_at | 0.160588494 |
| 86 | Mtr.42438.1.S1_at | Mtr.6871.1.S1_at | 0.142709664 |
| 87 | Mtr.42438.1.S1_at | Mtr.7739.1.S1_at | 0.220529838 |
| 88 | Mtr.42438.1.S1_at | Mtr.8335.1.S1_at | 0.128614382 |
| 89 | Mtr.42438.1.S1_at | Mtr.17709.1.S1_at | 0.115041476 |
| 90 | Mtr.3419.1.S1_at | Mtr.43961.1.S1_at | 0.123955505 |
| 91 | Mtr.3419.1.S1_at | Mtr.9613.1.S1_at | 0.106729997 |
| 92 | Mtr.34174.1.S1_at | Mtr.34980.1.S1_s_at | 0.148905485 |
| 93 | Mtr.34174.1.S1_at | Mtr.35144.1.S1_s_at | 0.108449585 |
| 94 | Mtr.34174.1.S1_at | Mtr.35759.1.S1_at | 0.115608112 |
| 95 | Mtr.34174.1.S1_at | Mtr.36854.1.S1_a_at | 0.104032081 |
| 96 | Mtr.34174.1.S1_at | Mtr.37168.1.S1_at | 0.122844068 |
| 97 | Mtr.34174.1.S1_at | Mtr.37710.1.S1_at | 0.146287371 |
| 98 | Mtr.34174.1.S1_at | Mtr.386.1.S1_at | 0.12577757 |
| 99 | Mtr.34174.1.S1_at | Mtr.38776.1.S1_at | 0.122831099 |
| 100 | Mtr.34174.1.S1_at | Mtr.39341.1.S1_at | 0.110654856 |
| 101 | Mtr.34174.1.S1_at | Mtr.39465.1.S1_at | 0.102392756 |
| 102 | Mtr.34174.1.S1_at | Mtr.39830.1.S1_at | 0.113479979 |
| 103 | Mtr.34174.1.S1_at | Mtr.40429.1.S1_at | 0.106850814 |
| 104 | Mtr.34174.1.S1_at | Mtr.40763.1.S1_at | 0.109578659 |
| 105 | Mtr.34174.1.S1_at | Mtr.41307.1.S1_at | 0.109255475 |
| 106 | Mtr.34174.1.S1_at | Mtr.41386.1.S1_at | 0.120766022 |
| 107 | Mtr.34174.1.S1_at | Mtr.41883.1.S1_at | 0.104149041 |
| 108 | Mtr.34174.1.S1_at | Mtr.42120.1.S1_at | 0.104600599 |
| 109 | Mtr.34174.1.S1_at | Mtr.42207.1.S1_at | 0.102192068 |
| 110 | Mtr.34174.1.S1_at | Mtr.42389.1.S1_at | 0.106761607 |
| 111 | Mtr.34174.1.S1_at | Mtr.42814.1.S1_at | 0.131842123 |
| 112 | Mtr.34174.1.S1_at | Mtr.43204.1.S1_at | 0.128270087 |
| 113 | Mtr.34174.1.S1_at | Mtr.43577.1.S1_s_at | 0.122138695 |
| 114 | Mtr.34174.1.S1_at | Mtr.43657.1.S1_at | 0.103562347 |
| 115 | Mtr.34174.1.S1_at | Mtr.43784.1.S1_at | 0.126942137 |
| 116 | Mtr.34174.1.S1_at | Mtr.43832.1.S1_at | 0.106564122 |
| 117 | Mtr.34174.1.S1_at | Mtr.44074.1.S1_at | 0.118987421 |
| 118 | Mtr.34174.1.S1_at | Mtr.44309.1.S1_at | 0.105191953 |
| 119 | Mtr.34174.1.S1_at | Mtr.44823.1.S1_at | 0.156188893 |
| 120 | Mtr.34174.1.S1_at | Mtr.44894.1.S1_at | 0.127787327 |
| 121 | Mtr.34174.1.S1_at | Mtr.45351.1.S1_s_at | 0.11980928 |
| 122 | Mtr.34174.1.S1_at | Mtr.45394.1.S1_at | 0.136458958 |
| 123 | Mtr.34174.1.S1_at | Mtr.45407.1.S1_at | 0.10560925 |
| 124 | Mtr.34174.1.S1_at | Mtr.45465.1.S1_at | 0.191578105 |
| 125 | Mtr.34174.1.S1_at | Mtr.4790.1.S1_at | 0.131003285 |
| 126 | Mtr.34174.1.S1_at | Mtr.5072.1.S1_s_at | 0.15879394 |
| 127 | Mtr.34174.1.S1_at | Mtr.5249.1.S1_at | 0.143037457 |
| 128 | Mtr.34174.1.S1_at | Mtr.5284.1.S1_at | 0.103744572 |
| 129 | Mtr.34174.1.S1_at | Mtr.5340.1.S1_at | 0.11302368 |
| 130 | Mtr.34174.1.S1_at | Mtr.5477.1.S1_at | 0.122076788 |
| 131 | Mtr.34174.1.S1_at | Mtr.5517.1.S1_at | 0.136819478 |
| 132 | Mtr.34174.1.S1_at | Mtr.5571.1.S1_s_at | 0.102315693 |
| 133 | Mtr.34174.1.S1_at | Mtr.5717.1.S1_at | 0.15342436 |
| 134 | Mtr.34174.1.S1_at | Mtr.5902.1.S1_at | 0.131087171 |
| 135 | Mtr.34174.1.S1_at | Mtr.6833.1.S1_at | 0.133582729 |
| 136 | Mtr.34174.1.S1_at | Mtr.6936.1.S1_s_at | 0.116534041 |
| 137 | Mtr.34174.1.S1_at | Mtr.7058.1.S1_at | 0.144168578 |
| 138 | Mtr.34174.1.S1_at | Mtr.8698.1.S1_at | 0.104509489 |
| 139 | Mtr.34174.1.S1_at | Mtr.8975.1.S1_at | 0.115501038 |
| 140 | Mtr.34174.1.S1_at | Mtr.9474.1.S1_at | 0.113875345 |
| 141 | Mtr.34174.1.S1_at | Mtr.9668.1.S1_at | 0.147469747 |
| 142 | Mtr.34174.1.S1_at | Mtr.9704.1.S1_at | 0.105365786 |
| 143 | Mtr.34174.1.S1_at | Mtr.35805.1.S1_at | 0.107168498 |
| 144 | Mtr.9321.1.S1_at | Mtr.9474.1.S1_at | 0.103458168 |
| 145 | Mtr.9321.1.S1_at | Mtr.12699.1.S1_s_at | 0.127291676 |
| 146 | Mtr.9321.1.S1_at | Mtr.29364.1.S1_at | 0.12180802 |
| 147 | Mtr.9321.1.S1_at | Mtr.33617.1.S1_at | 0.1354002 |
| 148 | Mtr.9321.1.S1_at | Mtr.39091.1.S1_at | 0.118050856 |
| 149 | Mtr.9321.1.S1_at | Mtr.42208.1.S1_at | 0.199656789 |
| 150 | Mtr.9321.1.S1_at | Mtr.45457.1.S1_at | 0.118097046 |
| 151 | Mtr.9321.1.S1_at | Mtr.5431.1.S1_at | 0.134643041 |
| 152 | Mtr.9321.1.S1_at | Mtr.6966.1.S1_at | 0.145555343 |
| 153 | Mtr.9321.1.S1_at | Mtr.31455.1.S1_at | 0.13503825 |
| 154 | Mtr.40771.1.S1_at | Mtr.37508.1.S1_at | 0.174231359 |
| 155 | Mtr.40771.1.S1_at | Mtr.40417.1.S1_at | 0.128251263 |
| 156 | Mtr.42438.1.S1_at | Mtr.10236.1.S1_at | 0.109018655 |
| 157 | Mtr.42438.1.S1_at | Mtr.12318.1.S1_at | 0.118184581 |
| 158 | Mtr.42438.1.S1_at | Mtr.13266.1.S1_at | 0.130859438 |
| 159 | Mtr.42438.1.S1_at | Mtr.1509.1.S1_at | 0.142535656 |
| 160 | Mtr.42438.1.S1_at | Mtr.1671.1.S1_at | 0.192809622 |
| 161 | Mtr.42438.1.S1_at | Mtr.17610.1.S1_at | 0.148405236 |
| 162 | Mtr.42438.1.S1_at | Mtr.25525.1.S1_at | 0.195047657 |
| 163 | Mtr.42438.1.S1_at | Mtr.26960.1.S1_at | 0.119693498 |
| 164 | Mtr.42438.1.S1_at | Mtr.27524.1.S1_at | 0.151520247 |
| 165 | Mtr.42438.1.S1_at | Mtr.28181.1.S1_at | 0.179988431 |
| 166 | Mtr.42438.1.S1_at | Mtr.28251.1.S1_at | 0.123876132 |
| 167 | Mtr.42438.1.S1_at | Mtr.28379.1.S1_at | 0.158062421 |
| 168 | Mtr.42438.1.S1_at | Mtr.28977.1.S1_at | 0.213678965 |
| 169 | Mtr.42438.1.S1_at | Mtr.29115.1.S1_at | 0.112588635 |
| 170 | Mtr.42438.1.S1_at | Mtr.29543.1.S1_at | 0.110178119 |
| 171 | Mtr.42438.1.S1_at | Mtr.29708.1.S1_at | 0.147149083 |
| 172 | Mtr.42438.1.S1_at | Mtr.29719.1.S1_at | 0.170067509 |
| 173 | Mtr.42438.1.S1_at | Mtr.29773.1.S1_at | 0.114317637 |
| 174 | Mtr.42438.1.S1_at | Mtr.29789.1.S1_at | 0.168639684 |
| 175 | Mtr.42438.1.S1_at | Mtr.29865.1.S1_at | 0.139743152 |
| 176 | Mtr.42438.1.S1_at | Mtr.29880.1.S1_at | 0.168788667 |
| 177 | Mtr.42438.1.S1_at | Mtr.29898.1.S1_at | 0.129786457 |
| 178 | Mtr.42438.1.S1_at | Mtr.30114.1.S1_at | 0.180649123 |
| 179 | Mtr.42438.1.S1_at | Mtr.30136.1.S1_at | 0.125758033 |
| 180 | Mtr.42438.1.S1_at | Mtr.30257.1.S1_at | 0.177551841 |
| 181 | Mtr.42438.1.S1_at | Mtr.30372.1.S1_at | 0.226556632 |
| 182 | Mtr.42438.1.S1_at | Mtr.30439.1.S1_at | 0.12218568 |
| 183 | Mtr.42438.1.S1_at | Mtr.30490.1.S1_at | 0.153976002 |
| 184 | Mtr.42438.1.S1_at | Mtr.30650.1.S1_at | 0.125180314 |
| 185 | Mtr.42438.1.S1_at | Mtr.3357.1.S1_at | 0.147045352 |
| 186 | Mtr.42438.1.S1_at | Mtr.3434.1.S1_at | 0.209734254 |
| 187 | Mtr.42438.1.S1_at | Mtr.35477.1.S1_at | 0.102054722 |
| 188 | Mtr.42438.1.S1_at | Mtr.36289.1.S1_at | 0.154412397 |
| 189 | Mtr.42438.1.S1_at | Mtr.3632.1.S1_at | 0.185375477 |
| 190 | Mtr.42438.1.S1_at | Mtr.36441.1.S1_at | 0.121767596 |
| 191 | Mtr.42438.1.S1_at | Mtr.3667.1.S1_at | 0.125624385 |
| 192 | Mtr.42438.1.S1_at | Mtr.3670.1.S1_at | 0.200865306 |
| 193 | Mtr.42438.1.S1_at | Mtr.36783.1.S1_at | 0.172215325 |
| 194 | Mtr.42438.1.S1_at | Mtr.36922.1.S1_at | 0.133560447 |
| 195 | Mtr.42438.1.S1_at | Mtr.36943.1.S1_at | 0.106366242 |
| 196 | Mtr.42438.1.S1_at | Mtr.36954.1.S1_at | 0.118926194 |
| 197 | Mtr.42438.1.S1_at | Mtr.36993.1.S1_at | 0.138182448 |
| 198 | Mtr.42438.1.S1_at | Mtr.37029.1.S1_at | 0.109027678 |
| 199 | Mtr.42438.1.S1_at | Mtr.37365.1.S1_at | 0.100176005 |
| 200 | Mtr.42438.1.S1_at | Mtr.37578.1.S1_at | 0.110913235 |
| 201 | Mtr.42438.1.S1_at | Mtr.3774.1.S1_at | 0.20352075 |
| 202 | Mtr.42438.1.S1_at | Mtr.3883.1.S1_at | 0.12569484 |
| 203 | Mtr.42438.1.S1_at | Mtr.3940.1.S1_at | 0.105170073 |
| 204 | Mtr.42438.1.S1_at | Mtr.39492.1.S1_at | 0.101435132 |
| 205 | Mtr.42438.1.S1_at | Mtr.41675.1.S1_at | 0.175424017 |
| 206 | Mtr.3419.1.S1_at | Mtr.10029.1.S1_at | 0.106329377 |
| 207 | Mtr.3419.1.S1_at | Mtr.10029.1.S1_s_at | 0.169995162 |
| 208 | Mtr.3419.1.S1_at | Mtr.12373.1.S1_at | 0.113046449 |
| 209 | Mtr.3419.1.S1_at | Mtr.22365.1.S1_at | 0.11557277 |
| 210 | Mtr.3419.1.S1_at | Mtr.25128.1.S1_at | 0.117245909 |
| 211 | Mtr.3419.1.S1_at | Mtr.32772.1.S1_at | 0.159119724 |
| 212 | Mtr.3419.1.S1_at | Mtr.40734.1.S1_at | 0.121502011 |
| 213 | Mtr.3419.1.S1_at | Mtr.44790.1.S1_at | 0.1063237 |
| 214 | Mtr.3419.1.S1_at | Mtr.27178.1.S1_at | 0.117206113 |
| 215 | Mtr.3419.1.S1_at | Mtr.38235.1.S1_at | 0.105184508 |
| 216 | Mtr.9321.1.S1_at | Mtr.10148.1.S1_at | 0.170199526 |
| 217 | Mtr.9321.1.S1_at | Mtr.10165.1.S1_at | 0.119091725 |
| 218 | Mtr.34174.1.S1_at | Mtr.10246.1.S1_at | 0.116573294 |
| 219 | Mtr.34174.1.S1_at | Mtr.10616.1.S1_at | 0.123482145 |
| 220 | Mtr.34174.1.S1_at | Mtr.10951.1.S1_at | 0.104762682 |
| 221 | Mtr.34174.1.S1_at | Mtr.11105.1.S1_at | 0.118985124 |
| 222 | Mtr.9321.1.S1_at | Mtr.11112.1.S1_at | 0.132999537 |
| 223 | Mtr.9321.1.S1_at | Mtr.11294.1.S1_at | 0.107570149 |
| 224 | Mtr.9321.1.S1_at | Mtr.11334.1.S1_at | 0.1145908 |
| 225 | Mtr.34174.1.S1_at | Mtr.11338.1.S1_at | 0.103517832 |
| 226 | Mtr.9321.1.S1_at | Mtr.11389.1.S1_at | 0.148354354 |
| 227 | Mtr.9321.1.S1_at | Mtr.11466.1.S1_at | 0.167090281 |
| 228 | Mtr.34174.1.S1_at | Mtr.11573.1.S1_at | 0.106740422 |
| 229 | Mtr.9321.1.S1_at | Mtr.11712.1.S1_at | 0.139462778 |
| 230 | Mtr.9321.1.S1_at | Mtr.12185.1.S1_at | 0.148661421 |
| 231 | Mtr.9321.1.S1_at | Mtr.12578.1.S1_at | 0.115540421 |
| 232 | Mtr.34174.1.S1_at | Mtr.12684.1.S1_at | 0.110458405 |
| 233 | Mtr.9321.1.S1_at | Mtr.12730.1.S1_at | 0.121723936 |
| 234 | Mtr.9321.1.S1_at | Mtr.12764.1.S1_at | 0.129922364 |
| 235 | Mtr.9321.1.S1_at | Mtr.13014.1.S1_at | 0.116848967 |
| 236 | Mtr.9321.1.S1_at | Mtr.13081.1.S1_at | 0.178537983 |
| 237 | Mtr.9321.1.S1_at | Mtr.13437.1.S1_at | 0.209951416 |
| 238 | Mtr.34174.1.S1_at | Mtr.13486.1.S1_at | 0.12283006 |
| 239 | Mtr.34174.1.S1_at | Mtr.13536.1.S1_at | 0.116700784 |
| 240 | Mtr.9321.1.S1_at | Mtr.13544.1.S1_at | 0.125330301 |
| 241 | Mtr.9321.1.S1_at | Mtr.13558.1.S1_at | 0.123863112 |
| 242 | Mtr.34174.1.S1_at | Mtr.1359.1.S1_at | 0.162458582 |
| 243 | Mtr.9321.1.S1_at | Mtr.13631.1.S1_at | 0.119657183 |
| 244 | Mtr.9321.1.S1_at | Mtr.13804.1.S1_at | 0.104289305 |
| 245 | Mtr.9321.1.S1_at | Mtr.14053.1.S1_s_at | 0.224765159 |
| 246 | Mtr.9321.1.S1_at | Mtr.14472.1.S1_s_at | 0.113037083 |
| 247 | Mtr.34174.1.S1_at | Mtr.1851.1.S1_s_at | 0.15583554 |
| 248 | Mtr.34174.1.S1_at | Mtr.19376.1.S1_at | 0.121407151 |
| 249 | Mtr.9321.1.S1_at | Mtr.19376.1.S1_at | 0.108178641 |
| 250 | Mtr.34174.1.S1_at | Mtr.19483.1.S1_at | 0.107412364 |
| 251 | Mtr.9321.1.S1_at | Mtr.19606.1.S1_at | 0.157070634 |
| 252 | Mtr.34174.1.S1_at | Mtr.2020.1.S1_at | 0.103082715 |
| 253 | Mtr.34174.1.S1_at | Mtr.2068.1.S1_at | 0.105201865 |
| 254 | Mtr.34174.1.S1_at | Mtr.20769.1.S1_at | 0.100968881 |
| 255 | Mtr.9321.1.S1_at | Mtr.20769.1.S1_at | 0.168063004 |
| 256 | Mtr.34174.1.S1_at | Mtr.22683.1.S1_s_at | 0.132235739 |
| 257 | Mtr.9321.1.S1_at | Mtr.22779.1.S1_s_at | 0.116422993 |
| 258 | Mtr.9321.1.S1_at | Mtr.2289.1.S1_at | 0.217113465 |
| 259 | Mtr.34174.1.S1_at | Mtr.23085.1.S1_s_at | 0.108953771 |
| 260 | Mtr.34174.1.S1_at | Mtr.23121.1.S1_at | 0.124094979 |
| 261 | Mtr.9321.1.S1_at | Mtr.2364.1.S1_at | 0.125838327 |
| 262 | Mtr.9321.1.S1_at | Mtr.23843.1.S1_at | 0.177426093 |
| 263 | Mtr.34174.1.S1_at | Mtr.24424.1.S1_s_at | 0.117809516 |
| 264 | Mtr.9321.1.S1_at | Mtr.25749.1.S1_at | 0.12325006 |
| 265 | Mtr.9321.1.S1_at | Mtr.27042.1.S1_at | 0.152360566 |
| 266 | Mtr.34174.1.S1_at | Mtr.27061.1.S1_at | 0.134423894 |
| 267 | Mtr.34174.1.S1_at | Mtr.27566.1.S1_at | 0.133436142 |
| 268 | Mtr.9321.1.S1_at | Mtr.27613.1.S1_at | 0.178268651 |
| 269 | Mtr.9321.1.S1_at | Mtr.27624.1.S1_at | 0.135893222 |
| 270 | Mtr.9321.1.S1_at | Mtr.28200.1.S1_at | 0.122798662 |
| 271 | Mtr.34174.1.S1_at | Mtr.2857.1.S1_s_at | 0.104992426 |
| 272 | Mtr.34174.1.S1_at | Mtr.28614.1.S1_s_at | 0.116146302 |
| 273 | Mtr.34174.1.S1_at | Mtr.28663.1.S1_at | 0.108321954 |
| 274 | Mtr.34174.1.S1_at | Mtr.28840.1.S1_at | 0.118106715 |
| 275 | Mtr.34174.1.S1_at | Mtr.28938.1.S1_at | 0.138154198 |
| 276 | Mtr.9321.1.S1_at | Mtr.29907.1.S1_at | 0.111393347 |
| 277 | Mtr.9321.1.S1_at | Mtr.29991.1.S1_at | 0.138979487 |
| 278 | Mtr.9321.1.S1_at | Mtr.30026.1.S1_at | 0.199594168 |
| 279 | Mtr.9321.1.S1_at | Mtr.30231.1.S1_at | 0.152090423 |
| 280 | Mtr.9321.1.S1_at | Mtr.30324.1.S1_at | 0.112048179 |
| 281 | Mtr.9321.1.S1_at | Mtr.3042.1.S1_at | 0.130843812 |
| 282 | Mtr.9321.1.S1_at | Mtr.30452.1.S1_at | 0.106331376 |
| 283 | Mtr.9321.1.S1_at | Mtr.31452.1.S1_at | 0.159930744 |
| 284 | Mtr.34174.1.S1_at | Mtr.31950.1.S1_at | 0.14218749 |
| 285 | Mtr.34174.1.S1_at | Mtr.32071.1.S1_at | 0.129942908 |
| 286 | Mtr.9321.1.S1_at | Mtr.32195.1.S1_at | 0.156452609 |
| 287 | Mtr.34174.1.S1_at | Mtr.32208.1.S1_at | 0.172214173 |
| 288 | Mtr.9321.1.S1_at | Mtr.32315.1.S1_at | 0.168058703 |
| 289 | Mtr.34174.1.S1_at | Mtr.32357.1.S1_at | 0.131003686 |
| 290 | Mtr.9321.1.S1_at | Mtr.32409.1.S1_at | 0.209450295 |
| 291 | Mtr.34174.1.S1_at | Mtr.32486.1.S1_at | 0.126643467 |
| 292 | Mtr.34174.1.S1_at | Mtr.32556.1.S1_at | 0.101304372 |
| 293 | Mtr.34174.1.S1_at | Mtr.32646.1.S1_at | 0.128164051 |
| 294 | Mtr.9321.1.S1_at | Mtr.32646.1.S1_at | 0.118676514 |
| 295 | Mtr.34174.1.S1_at | Mtr.32930.1.S1_at | 0.101556817 |
| 296 | Mtr.34174.1.S1_at | Mtr.33098.1.S1_at | 0.149751493 |
| 297 | Mtr.34174.1.S1_at | Mtr.33102.1.S1_at | 0.1235188 |
| 298 | Mtr.9321.1.S1_at | Mtr.33179.1.S1_at | 0.195711132 |
| 299 | Mtr.9321.1.S1_at | Mtr.33189.1.S1_s_at | 0.244145449 |
| 300 | Mtr.9321.1.S1_at | Mtr.33334.1.S1_at | 0.107500879 |
| 301 | Mtr.34174.1.S1_at | Mtr.33420.1.S1_at | 0.133907514 |
| 302 | Mtr.9321.1.S1_at | Mtr.33544.1.S1_at | 0.197135923 |
| 303 | Mtr.9321.1.S1_at | Mtr.33626.1.S1_at | 0.104917284 |
| 304 | Mtr.9321.1.S1_at | Mtr.33797.1.S1_at | 0.108864225 |
| 305 | Mtr.34174.1.S1_at | Mtr.34126.1.S1_at | 0.126507704 |
| 306 | Mtr.9321.1.S1_at | Mtr.34147.1.S1_at | 0.114306542 |
| 307 | Mtr.34174.1.S1_at | Mtr.34163.1.S1_at | 0.123234377 |
| 308 | Mtr.9321.1.S1_at | Mtr.34217.1.S1_at | 0.239428014 |
| 309 | Mtr.9321.1.S1_at | Mtr.34901.1.S1_s_at | 0.183873925 |
| 310 | Mtr.9321.1.S1_at | Mtr.34940.1.S1_at | 0.194975865 |
| 311 | Mtr.9321.1.S1_at | Mtr.34967.1.S1_at | 0.107777934 |
| 312 | Mtr.9321.1.S1_at | Mtr.35042.1.S1_at | 0.122258221 |
| 313 | Mtr.9321.1.S1_at | Mtr.35045.1.S1_s_at | 0.21280258 |
| 314 | Mtr.9321.1.S1_at | Mtr.35047.1.S1_s_at | 0.117788734 |
| 315 | Mtr.9321.1.S1_at | Mtr.35137.1.S1_at | 0.103314559 |
| 316 | Mtr.9321.1.S1_at | Mtr.36393.1.S1_at | 0.102827758 |
| 317 | Mtr.9321.1.S1_at | Mtr.36891.1.S1_at | 0.154587908 |
| 318 | Mtr.9321.1.S1_at | Mtr.3692.1.S1_at | 0.1397637 |
| 319 | Mtr.9321.1.S1_at | Mtr.37208.1.S1_at | 0.103765067 |
| 320 | Mtr.9321.1.S1_at | Mtr.37268.1.S1_s_at | 0.133375333 |
| 321 | Mtr.9321.1.S1_at | Mtr.37284.1.S1_at | 0.134348857 |
| 322 | Mtr.9321.1.S1_at | Mtr.3795.1.S1_at | 0.157131983 |
| 323 | Mtr.9321.1.S1_at | Mtr.38070.1.S1_at | 0.146871205 |
| 324 | Mtr.9321.1.S1_at | Mtr.38596.1.S1_at | 0.170585288 |
| 325 | Mtr.9321.1.S1_at | Mtr.38802.1.S1_at | 0.10765824 |
| 326 | Mtr.9321.1.S1_at | Mtr.38830.1.S1_at | 0.136960062 |
| 327 | Mtr.9321.1.S1_at | Mtr.39465.1.S1_at | 0.113846313 |
| 328 | Mtr.9321.1.S1_at | Mtr.40523.1.S1_at | 0.11932184 |
| 329 | Mtr.9321.1.S1_at | Mtr.40627.1.S1_at | 0.128311597 |
| 330 | Mtr.9321.1.S1_at | Mtr.41056.1.S1_at | 0.105095289 |
| 331 | Mtr.9321.1.S1_at | Mtr.4130.1.S1_at | 0.17935456 |
| 332 | Mtr.9321.1.S1_at | Mtr.41400.1.S1_s_at | 0.110322924 |
| 333 | Mtr.9321.1.S1_at | Mtr.4154.1.S1_at | 0.210079478 |
| 334 | Mtr.9321.1.S1_at | Mtr.42024.1.S1_at | 0.113323998 |
| 335 | Mtr.9321.1.S1_at | Mtr.42224.1.S1_s_at | 0.111132837 |
| 336 | Mtr.9321.1.S1_at | Mtr.42470.1.S1_at | 0.107807682 |
| 337 | Mtr.9321.1.S1_at | Mtr.42470.1.S1_x_at | 0.110276406 |
| 338 | Mtr.9321.1.S1_at | Mtr.42722.1.S1_at | 0.121167634 |
| 339 | Mtr.9321.1.S1_at | Mtr.43657.1.S1_at | 0.14888079 |
| 340 | Mtr.9321.1.S1_at | Mtr.43784.1.S1_at | 0.125867121 |
| 341 | Mtr.9321.1.S1_at | Mtr.43799.1.S1_at | 0.219168673 |
| 342 | Mtr.9321.1.S1_at | Mtr.43957.1.S1_s_at | 0.141713656 |
| 343 | Mtr.9321.1.S1_at | Mtr.44568.1.S1_at | 0.19499284 |
| 344 | Mtr.9321.1.S1_at | Mtr.44941.1.S1_s_at | 0.162840298 |
| 345 | Mtr.9321.1.S1_at | Mtr.45054.1.S1_at | 0.128760096 |
| 346 | Mtr.9321.1.S1_at | Mtr.45133.1.S1_at | 0.177065773 |
| 347 | Mtr.9321.1.S1_at | Mtr.45139.1.S1_at | 0.122424165 |
| 348 | Mtr.9321.1.S1_at | Mtr.45323.1.S1_at | 0.148828887 |
| 349 | Mtr.9321.1.S1_at | Mtr.45351.1.S1_s_at | 0.127482886 |
| 350 | Mtr.9321.1.S1_at | Mtr.45407.1.S1_at | 0.102815281 |
| 351 | Mtr.9321.1.S1_at | Mtr.45563.1.S1_at | 0.172627579 |
| 352 | Mtr.9321.1.S1_at | Mtr.45587.1.S1_at | 0.121202255 |
| 353 | Mtr.9321.1.S1_at | Mtr.46373.1.S1_at | 0.160263401 |
| 354 | Mtr.9321.1.S1_at | Mtr.49221.1.S1_at | 0.138861226 |
| 355 | Mtr.9321.1.S1_at | Mtr.49236.1.S1_at | 0.125832891 |
| 356 | Mtr.9321.1.S1_at | Mtr.5557.1.S1_at | 0.168611869 |
| 357 | Mtr.9321.1.S1_at | Mtr.5686.1.S1_at | 0.191491303 |
| 358 | Mtr.9321.1.S1_at | Mtr.5976.1.S1_at | 0.180008264 |
| 359 | Mtr.9321.1.S1_at | Mtr.6046.1.S1_at | 0.171096673 |
| 360 | Mtr.9321.1.S1_at | Mtr.6324.1.S1_at | 0.151790395 |
| 361 | Mtr.9321.1.S1_at | Mtr.6769.1.S1_at | 0.169089209 |
| 362 | Mtr.9321.1.S1_at | Mtr.6769.1.S1_s_at | 0.159871051 |
| 363 | Mtr.9321.1.S1_at | Mtr.6774.1.S1_at | 0.166851659 |
| 364 | Mtr.9321.1.S1_at | Mtr.6950.1.S1_at | 0.214539392 |
| 365 | Mtr.9321.1.S1_at | Mtr.8975.1.S1_at | 0.157824177 |
| 366 | Mtr.9321.1.S1_at | Mtr.9016.1.S1_at | 0.172616221 |
| 367 | Mtr.9321.1.S1_at | Mtr.9101.1.S1_at | 0.220632914 |
| 368 | Mtr.9321.1.S1_at | Mtr.9235.1.S1_s_at | 0.106035337 |

|  | Supplementary Table 5-2. The co-expression network of MtHAK genes in DEGs under drought stress. | | |
| --- | --- | --- | --- |
|  | fromNode | toNode | weight |
| 1 | Mtr.3419.1.S1_at | Mtr.34485.1.S1_at | 0.202594623 |
| 2 | Mtr.3419.1.S1_at | Mtr.34605.1.S1_at | 0.148200524 |
| 3 | Mtr.3419.1.S1_at | Mtr.34705.1.S1_at | 0.146408264 |
| 4 | Mtr.3419.1.S1_at | Mtr.34709.1.S1_at | 0.218797914 |
| 5 | Mtr.3419.1.S1_at | Mtr.34746.1.S1_at | 0.14183835 |
| 6 | Mtr.3419.1.S1_at | Mtr.34752.1.S1_s_at | 0.157220744 |
| 7 | Mtr.3419.1.S1_at | Mtr.34757.1.S1_at | 0.156226349 |
| 8 | Mtr.3419.1.S1_at | Mtr.34842.1.S1_at | 0.116055013 |
| 9 | Mtr.3419.1.S1_at | Mtr.35282.1.S1_at | 0.187942431 |
| 10 | Mtr.3419.1.S1_at | Mtr.35296.1.S1_at | 0.110802547 |
| 11 | Mtr.3419.1.S1_at | Mtr.35311.1.S1_at | 0.125660042 |
| 12 | Mtr.3419.1.S1_at | Mtr.3534.1.S1_at | 0.138070893 |
| 13 | Mtr.3419.1.S1_at | Mtr.35894.1.S1_at | 0.127647689 |
| 14 | Mtr.3419.1.S1_at | Mtr.36075.1.S1_x_at | 0.179374717 |
| 15 | Mtr.3419.1.S1_at | Mtr.36454.1.S1_at | 0.106931953 |
| 16 | Mtr.3419.1.S1_at | Mtr.36908.1.S1_at | 0.144219507 |
| 17 | Mtr.3419.1.S1_at | Mtr.36929.1.S1_s_at | 0.163952401 |
| 18 | Mtr.3419.1.S1_at | Mtr.36936.1.S1_at | 0.181056491 |
| 19 | Mtr.3419.1.S1_at | Mtr.37175.1.S1_at | 0.252829738 |
| 20 | Mtr.3419.1.S1_at | Mtr.37286.1.S1_at | 0.271477348 |
| 21 | Mtr.3419.1.S1_at | Mtr.37287.1.S1_s_at | 0.233798603 |
| 22 | Mtr.3419.1.S1_at | Mtr.37288.1.S1_at | 0.205938011 |
| 23 | Mtr.3419.1.S1_at | Mtr.37296.1.S1_at | 0.176364846 |
| 24 | Mtr.3419.1.S1_at | Mtr.37341.1.S1_at | 0.105283179 |
| 25 | Mtr.3419.1.S1_at | Mtr.37363.1.S1_at | 0.116008875 |
| 26 | Mtr.3419.1.S1_at | Mtr.37596.1.S1_at | 0.136103279 |
| 27 | Mtr.3419.1.S1_at | Mtr.37642.1.S1_at | 0.118135815 |
| 28 | Mtr.3419.1.S1_at | Mtr.37681.1.S1_at | 0.11949544 |
| 29 | Mtr.3419.1.S1_at | Mtr.37812.1.S1_at | 0.160781064 |
| 30 | Mtr.3419.1.S1_at | Mtr.37903.1.S1_at | 0.138007727 |
| 31 | Mtr.3419.1.S1_at | Mtr.38025.1.S1_at | 0.239056281 |
| 32 | Mtr.3419.1.S1_at | Mtr.38432.1.S1_at | 0.121642864 |
| 33 | Mtr.3419.1.S1_at | Mtr.38462.1.S1_at | 0.103295622 |
| 34 | Mtr.3419.1.S1_at | Mtr.38548.1.S1_at | 0.204959624 |
| 35 | Mtr.3419.1.S1_at | Mtr.38665.1.S1_at | 0.239690938 |
| 36 | Mtr.3419.1.S1_at | Mtr.38707.1.S1_at | 0.144674837 |
| 37 | Mtr.3419.1.S1_at | Mtr.38756.1.S1_at | 0.133034354 |
| 38 | Mtr.3419.1.S1_at | Mtr.38785.1.S1_at | 0.197751686 |
| 39 | Mtr.3419.1.S1_at | Mtr.38837.1.S1_s_at | 0.103020735 |
| 40 | Mtr.3419.1.S1_at | Mtr.38983.1.S1_at | 0.120262588 |
| 41 | Mtr.3419.1.S1_at | Mtr.38984.1.S1_at | 0.192165599 |
| 42 | Mtr.3419.1.S1_at | Mtr.38995.1.S1_at | 0.191554496 |
| 43 | Mtr.3419.1.S1_at | Mtr.38999.1.S1_at | 0.178693243 |
| 44 | Mtr.3419.1.S1_at | Mtr.39229.1.S1_at | 0.153754689 |
| 45 | Mtr.3419.1.S1_at | Mtr.39289.1.S1_at | 0.161178148 |
| 46 | Mtr.3419.1.S1_at | Mtr.39384.1.S1_at | 0.168307395 |
| 47 | Mtr.3419.1.S1_at | Mtr.39491.1.S1_at | 0.197237315 |
| 48 | Mtr.3419.1.S1_at | Mtr.39766.1.S1_s_at | 0.13252062 |
| 49 | Mtr.3419.1.S1_at | Mtr.39776.1.S1_at | 0.105322773 |
| 50 | Mtr.3419.1.S1_at | Mtr.39777.1.S1_at | 0.176945056 |
| 51 | Mtr.3419.1.S1_at | Mtr.39844.1.S1_at | 0.127030662 |
| 52 | Mtr.3419.1.S1_at | Mtr.39899.1.S1_at | 0.128278687 |
| 53 | Mtr.3419.1.S1_at | Mtr.39944.1.S1_at | 0.178015666 |
| 54 | Mtr.3419.1.S1_at | Mtr.39946.1.S1_s_at | 0.124258046 |
| 55 | Mtr.3419.1.S1_at | Mtr.40028.1.S1_at | 0.149007992 |
| 56 | Mtr.3419.1.S1_at | Mtr.40052.1.S1_at | 0.151758793 |
| 57 | Mtr.3419.1.S1_at | Mtr.40121.1.S1_at | 0.156870518 |
| 58 | Mtr.3419.1.S1_at | Mtr.40206.1.S1_at | 0.175495167 |
| 59 | Mtr.3419.1.S1_at | Mtr.40245.1.S1_s_at | 0.269964239 |
| 60 | Mtr.3419.1.S1_at | Mtr.40436.1.S1_at | 0.203834807 |
| 61 | Mtr.3419.1.S1_at | Mtr.40496.1.S1_at | 0.163328068 |
| 62 | Mtr.3419.1.S1_at | Mtr.40706.1.S1_s_at | 0.119135413 |
| 63 | Mtr.3419.1.S1_at | Mtr.40710.1.S1_at | 0.176531103 |
| 64 | Mtr.3419.1.S1_at | Mtr.41162.1.S1_at | 0.16608621 |
| 65 | Mtr.3419.1.S1_at | Mtr.41373.1.S1_at | 0.14244986 |
| 66 | Mtr.3419.1.S1_at | Mtr.41387.1.S1_at | 0.20123973 |
| 67 | Mtr.3419.1.S1_at | Mtr.41445.1.S1_at | 0.102919237 |
| 68 | Mtr.3419.1.S1_at | Mtr.41601.1.S1_at | 0.175767952 |
| 69 | Mtr.3419.1.S1_at | Mtr.41645.1.S1_at | 0.193555591 |
| 70 | Mtr.3419.1.S1_at | Mtr.41661.1.S1_s_at | 0.118777642 |
| 71 | Mtr.3419.1.S1_at | Mtr.41681.1.S1_at | 0.127231972 |
| 72 | Mtr.3419.1.S1_at | Mtr.41836.1.S1_at | 0.126331808 |
| 73 | Mtr.3419.1.S1_at | Mtr.42289.1.S1_at | 0.125504559 |
| 74 | Mtr.3419.1.S1_at | Mtr.42470.1.S1_x_at | 0.127077649 |
| 75 | Mtr.3419.1.S1_at | Mtr.42474.1.S1_at | 0.145934795 |
| 76 | Mtr.3419.1.S1_at | Mtr.42552.1.S1_at | 0.186351636 |
| 77 | Mtr.3419.1.S1_at | Mtr.42601.1.S1_at | 0.247539339 |
| 78 | Mtr.3419.1.S1_at | Mtr.42682.1.S1_at | 0.163565477 |
| 79 | Mtr.3419.1.S1_at | Mtr.42697.1.S1_at | 0.165639723 |
| 80 | Mtr.3419.1.S1_at | Mtr.42713.1.S1_at | 0.150931004 |
| 81 | Mtr.3419.1.S1_at | Mtr.42770.1.S1_at | 0.157146595 |
| 82 | Mtr.3419.1.S1_at | Mtr.42855.1.S1_s_at | 0.125557705 |
| 83 | Mtr.3419.1.S1_at | Mtr.42933.1.S1_x_at | 0.159219131 |
| 84 | Mtr.3419.1.S1_at | Mtr.43044.1.S1_at | 0.267891559 |
| 85 | Mtr.3419.1.S1_at | Mtr.43055.1.S1_s_at | 0.166150629 |
| 86 | Mtr.3419.1.S1_at | Mtr.43283.1.S1_at | 0.153517016 |
| 87 | Mtr.3419.1.S1_at | Mtr.43567.1.S1_at | 0.134265996 |
| 88 | Mtr.3419.1.S1_at | Mtr.43628.1.S1_at | 0.185783173 |
| 89 | Mtr.3419.1.S1_at | Mtr.43641.1.S1_at | 0.187165277 |
| 90 | Mtr.3419.1.S1_at | Mtr.43736.1.S1_at | 0.197773273 |
| 91 | Mtr.3419.1.S1_at | Mtr.43741.1.S1_at | 0.149939003 |
| 92 | Mtr.3419.1.S1_at | Mtr.4375.1.S1_at | 0.134935588 |
| 93 | Mtr.3419.1.S1_at | Mtr.43912.1.S1_at | 0.236830766 |
| 94 | Mtr.3419.1.S1_at | Mtr.43915.1.S1_at | 0.143177865 |
| 95 | Mtr.3419.1.S1_at | Mtr.43962.1.S1_at | 0.17863383 |
| 96 | Mtr.3419.1.S1_at | Mtr.44078.1.S1_at | 0.107401049 |
| 97 | Mtr.3419.1.S1_at | Mtr.44126.1.S1_s_at | 0.182916791 |
| 98 | Mtr.3419.1.S1_at | Mtr.44134.1.S1_at | 0.127622394 |
| 99 | Mtr.3419.1.S1_at | Mtr.44169.1.S1_at | 0.166530903 |
| 100 | Mtr.3419.1.S1_at | Mtr.44217.1.S1_at | 0.19961518 |
| 101 | Mtr.3419.1.S1_at | Mtr.44239.1.S1_at | 0.130573103 |
| 102 | Mtr.3419.1.S1_at | Mtr.44337.1.S1_at | 0.13705009 |
| 103 | Mtr.3419.1.S1_at | Mtr.44410.1.S1_at | 0.17011863 |
| 104 | Mtr.3419.1.S1_at | Mtr.4454.1.S1_at | 0.161034047 |
| 105 | Mtr.3419.1.S1_at | Mtr.44614.1.S1_at | 0.158082443 |
| 106 | Mtr.3419.1.S1_at | Mtr.44666.1.S1_at | 0.135471657 |
| 107 | Mtr.3419.1.S1_at | Mtr.44672.1.S1_at | 0.204738543 |
| 108 | Mtr.3419.1.S1_at | Mtr.44857.1.S1_at | 0.102162439 |
| 109 | Mtr.3419.1.S1_at | Mtr.44889.1.S1_at | 0.110772614 |
| 110 | Mtr.3419.1.S1_at | Mtr.44919.1.S1_at | 0.185608967 |
| 111 | Mtr.3419.1.S1_at | Mtr.44931.1.S1_at | 0.193926638 |
| 112 | Mtr.3419.1.S1_at | Mtr.44967.1.S1_at | 0.112126126 |
| 113 | Mtr.3419.1.S1_at | Mtr.44995.1.S1_at | 0.120576951 |
| 114 | Mtr.3419.1.S1_at | Mtr.45056.1.S1_s_at | 0.206897161 |
| 115 | Mtr.3419.1.S1_at | Mtr.45131.1.S1_at | 0.195955009 |
| 116 | Mtr.3419.1.S1_at | Mtr.45154.1.S1_at | 0.138764883 |
| 117 | Mtr.3419.1.S1_at | Mtr.45251.1.S1_at | 0.14298782 |
| 118 | Mtr.3419.1.S1_at | Mtr.45276.1.S1_at | 0.108945627 |
| 119 | Mtr.3419.1.S1_at | Mtr.45295.1.S1_s_at | 0.18124358 |
| 120 | Mtr.3419.1.S1_at | Mtr.45337.1.S1_at | 0.1667743 |
| 121 | Mtr.3419.1.S1_at | Mtr.45511.1.S1_at | 0.165412442 |
| 122 | Mtr.3419.1.S1_at | Mtr.4567.1.S1_at | 0.143009291 |
| 123 | Mtr.3419.1.S1_at | Mtr.45971.1.S1_at | 0.163424198 |
| 124 | Mtr.3419.1.S1_at | Mtr.46110.1.S1_at | 0.17025771 |
| 125 | Mtr.3419.1.S1_at | Mtr.4626.1.S1_at | 0.199282377 |
| 126 | Mtr.3419.1.S1_at | Mtr.46591.1.S1_at | 0.163989879 |
| 127 | Mtr.3419.1.S1_at | Mtr.46615.1.S1_at | 0.1759781 |
| 128 | Mtr.3419.1.S1_at | Mtr.4727.1.S1_a_at | 0.134846304 |
| 129 | Mtr.3419.1.S1_at | Mtr.47857.1.S1_at | 0.149683403 |
| 130 | Mtr.3419.1.S1_at | Mtr.48400.1.S1_at | 0.112144993 |
| 131 | Mtr.3419.1.S1_at | Mtr.48401.1.S1_at | 0.127884865 |
| 132 | Mtr.3419.1.S1_at | Mtr.48778.1.S1_x_at | 0.202338925 |
| 133 | Mtr.3419.1.S1_at | Mtr.48782.1.S1_s_at | 0.151631724 |
| 134 | Mtr.3419.1.S1_at | Mtr.48912.1.S1_s_at | 0.158302391 |
| 135 | Mtr.3419.1.S1_at | Mtr.49063.1.S1_s_at | 0.11349355 |
| 136 | Mtr.3419.1.S1_at | Mtr.498.1.S1_s_at | 0.204059476 |
| 137 | Mtr.3419.1.S1_at | Mtr.50217.1.S1_s_at | 0.149640978 |
| 138 | Mtr.3419.1.S1_at | Mtr.5029.1.S1_at | 0.149296888 |
| 139 | Mtr.3419.1.S1_at | Mtr.50708.1.S1_at | 0.14835436 |
| 140 | Mtr.3419.1.S1_at | Mtr.50846.1.S1_at | 0.112645815 |
| 141 | Mtr.3419.1.S1_at | Mtr.51200.1.S1_at | 0.194306307 |
| 142 | Mtr.3419.1.S1_at | Mtr.51375.1.S1_at | 0.16455547 |
| 143 | Mtr.3419.1.S1_at | Mtr.51423.1.S1_at | 0.111418316 |
| 144 | Mtr.3419.1.S1_at | Mtr.51891.1.S1_at | 0.111884304 |
| 145 | Mtr.3419.1.S1_at | Mtr.52010.1.S1_at | 0.175225603 |
| 146 | Mtr.3419.1.S1_at | Mtr.52124.1.S1_s_at | 0.11576854 |
| 147 | Mtr.3419.1.S1_at | Mtr.5238.1.S1_at | 0.168838806 |
| 148 | Mtr.3419.1.S1_at | Mtr.5357.1.S1_at | 0.180347202 |
| 149 | Mtr.3419.1.S1_at | Mtr.5531.1.S1_at | 0.187373023 |
| 150 | Mtr.3419.1.S1_at | Mtr.5558.1.S1_at | 0.160418742 |
| 151 | Mtr.3419.1.S1_at | Mtr.5628.1.S1_s_at | 0.128429918 |
| 152 | Mtr.3419.1.S1_at | Mtr.5636.1.S1_at | 0.167734219 |
| 153 | Mtr.3419.1.S1_at | Mtr.5655.1.S1_s_at | 0.117779414 |
| 154 | Mtr.3419.1.S1_at | Mtr.5846.1.S1_at | 0.163697249 |
| 155 | Mtr.3419.1.S1_at | Mtr.5858.1.S1_at | 0.12348543 |
| 156 | Mtr.3419.1.S1_at | Mtr.588.1.S1_at | 0.185336546 |
| 157 | Mtr.3419.1.S1_at | Mtr.5964.1.S1_at | 0.106921538 |
| 158 | Mtr.3419.1.S1_at | Mtr.6066.1.S1_x_at | 0.16891847 |
| 159 | Mtr.3419.1.S1_at | Mtr.6070.1.S1_at | 0.16741153 |
| 160 | Mtr.3419.1.S1_at | Mtr.6104.1.S1_at | 0.205968363 |
| 161 | Mtr.3419.1.S1_at | Mtr.6135.1.S1_at | 0.215993389 |
| 162 | Mtr.3419.1.S1_at | Mtr.6521.1.S1_at | 0.263403175 |
| 163 | Mtr.3419.1.S1_at | Mtr.6553.1.S1_s_at | 0.161178632 |
| 164 | Mtr.3419.1.S1_at | Mtr.6632.1.S1_at | 0.186862581 |
| 165 | Mtr.3419.1.S1_at | Mtr.6696.1.S1_at | 0.102587153 |
| 166 | Mtr.3419.1.S1_at | Mtr.6698.1.S1_at | 0.198437769 |
| 167 | Mtr.3419.1.S1_at | Mtr.6699.1.S1_at | 0.244045133 |
| 168 | Mtr.3419.1.S1_at | Mtr.6737.1.S1_s_at | 0.158619432 |
| 169 | Mtr.3419.1.S1_at | Mtr.6812.1.S1_at | 0.124344044 |
| 170 | Mtr.3419.1.S1_at | Mtr.6863.1.S1_at | 0.140523643 |
| 171 | Mtr.3419.1.S1_at | Mtr.766.1.S1_at | 0.177394598 |
| 172 | Mtr.3419.1.S1_at | Mtr.8427.1.S1_at | 0.156738023 |
| 173 | Mtr.3419.1.S1_at | Mtr.8454.1.S1_at | 0.186654851 |
| 174 | Mtr.3419.1.S1_at | Mtr.8470.1.S1_s_at | 0.182451595 |
| 175 | Mtr.3419.1.S1_at | Mtr.8562.1.S1_at | 0.142868425 |
| 176 | Mtr.3419.1.S1_at | Mtr.8613.1.S1_at | 0.119338736 |
| 177 | Mtr.3419.1.S1_at | Mtr.8659.1.S1_s_at | 0.128166572 |
| 178 | Mtr.3419.1.S1_at | Mtr.8832.1.S1_at | 0.10191032 |
| 179 | Mtr.3419.1.S1_at | Mtr.8887.1.S1_at | 0.177498545 |
| 180 | Mtr.3419.1.S1_at | Mtr.9055.1.S1_at | 0.181846017 |
| 181 | Mtr.3419.1.S1_at | Mtr.9086.1.S1_at | 0.118678057 |
| 182 | Mtr.3419.1.S1_at | Mtr.9154.1.S1_at | 0.124366711 |
| 183 | Mtr.3419.1.S1_at | Mtr.9231.1.S1_at | 0.1833203 |
| 184 | Mtr.3419.1.S1_at | Mtr.9273.1.S1_at | 0.144990753 |
| 185 | Mtr.3419.1.S1_at | Mtr.929.1.S1_at | 0.179275192 |
| 186 | Mtr.3419.1.S1_at | Mtr.929.1.S1_s_at | 0.187268562 |
| 187 | Mtr.3419.1.S1_at | Mtr.9306.1.S1_at | 0.163684371 |
| 188 | Mtr.3419.1.S1_at | Mtr.9315.1.S1_at | 0.167615227 |
| 189 | Mtr.3419.1.S1_at | Mtr.9518.1.S1_at | 0.195997615 |
| 190 | Mtr.3419.1.S1_at | Mtr.9570.1.S1_at | 0.153572169 |
| 191 | Mtr.3419.1.S1_at | Mtr.9651.1.S1_at | 0.19797745 |
| 192 | Mtr.3419.1.S1_at | Mtr.9750.1.S1_at | 0.140036738 |
| 193 | Mtr.40771.1.S1_at | Mtr.41703.1.S1_at | 0.122759482 |
| 194 | Mtr.40771.1.S1_at | Mtr.42216.1.S1_at | 0.123651038 |
| 195 | Mtr.40771.1.S1_at | Mtr.4264.1.S1_at | 0.106524434 |
| 196 | Mtr.40771.1.S1_at | Mtr.42738.1.S1_at | 0.132363304 |
| 197 | Mtr.40771.1.S1_at | Mtr.42902.1.S1_at | 0.137652763 |
| 198 | Mtr.40771.1.S1_at | Mtr.43885.1.S1_at | 0.127925635 |
| 199 | Mtr.40771.1.S1_at | Mtr.43953.1.S1_at | 0.139432553 |
| 200 | Mtr.40771.1.S1_at | Mtr.43953.1.S1_s_at | 0.148896431 |
| 201 | Mtr.40771.1.S1_at | Mtr.43954.1.S1_at | 0.149439806 |
| 202 | Mtr.40771.1.S1_at | Mtr.44255.1.S1_at | 0.132429356 |
| 203 | Mtr.40771.1.S1_at | Mtr.44492.1.S1_at | 0.104580915 |
| 204 | Mtr.40771.1.S1_at | Mtr.44579.1.S1_at | 0.127276307 |
| 205 | Mtr.40771.1.S1_at | Mtr.44613.1.S1_at | 0.110289677 |
| 206 | Mtr.40771.1.S1_at | Mtr.44708.1.S1_at | 0.137276861 |
| 207 | Mtr.40771.1.S1_at | Mtr.44958.1.S1_at | 0.130705154 |
| 208 | Mtr.40771.1.S1_at | Mtr.5022.1.S1_s_at | 0.124342517 |
| 209 | Mtr.40771.1.S1_at | Mtr.51458.1.S1_at | 0.103551755 |
| 210 | Mtr.40771.1.S1_at | Mtr.52114.1.S1_s_at | 0.119446964 |
| 211 | Mtr.40771.1.S1_at | Mtr.5323.1.S1_at | 0.139091789 |
| 212 | Mtr.40771.1.S1_at | Mtr.5415.1.S1_s_at | 0.129448548 |
| 213 | Mtr.40771.1.S1_at | Mtr.548.1.S1_s_at | 0.141158145 |
| 214 | Mtr.40771.1.S1_at | Mtr.5494.1.S1_at | 0.125427323 |
| 215 | Mtr.40771.1.S1_at | Mtr.5706.1.S1_at | 0.13446939 |
| 216 | Mtr.40771.1.S1_at | Mtr.6088.1.S1_at | 0.104535343 |
| 217 | Mtr.40771.1.S1_at | Mtr.6659.1.S1_at | 0.133791808 |
| 218 | Mtr.40771.1.S1_at | Mtr.6789.1.S1_s_at | 0.123765508 |
| 219 | Mtr.40771.1.S1_at | Mtr.6855.1.S1_at | 0.122786798 |
| 220 | Mtr.40771.1.S1_at | Mtr.6910.1.S1_at | 0.10934917 |
| 221 | Mtr.40771.1.S1_at | Mtr.7235.1.S1_at | 0.148699238 |
| 222 | Mtr.40771.1.S1_at | Mtr.7326.1.S1_at | 0.109598964 |
| 223 | Mtr.40771.1.S1_at | Mtr.7394.1.S1_at | 0.138182911 |
| 224 | Mtr.40771.1.S1_at | Mtr.7576.1.S1_at | 0.131838491 |
| 225 | Mtr.40771.1.S1_at | Mtr.9822.1.S1_at | 0.136287319 |
| 226 | Mtr.40771.1.S1_at | Mtr.9968.1.S1_at | 0.127139277 |
| 227 | Mtr.3419.1.S1_at | Mtr.10048.1.S1_at | 0.115198187 |
| 228 | Mtr.40771.1.S1_at | Mtr.10348.1.S1_at | 0.133936606 |
| 229 | Mtr.3419.1.S1_at | Mtr.10456.1.S1_at | 0.237762344 |
| 230 | Mtr.3419.1.S1_at | Mtr.10457.1.S1_at | 0.182363076 |
| 231 | Mtr.3419.1.S1_at | Mtr.10458.1.S1_at | 0.207451826 |
| 232 | Mtr.40771.1.S1_at | Mtr.10619.1.S1_at | 0.10203932 |
| 233 | Mtr.3419.1.S1_at | Mtr.1068.1.S1_at | 0.142467763 |
| 234 | Mtr.3419.1.S1_at | Mtr.1068.1.S1_x_at | 0.135129107 |
| 235 | Mtr.3419.1.S1_at | Mtr.10929.1.S1_at | 0.149179084 |
| 236 | Mtr.3419.1.S1_at | Mtr.1093.1.S1_at | 0.12354384 |
| 237 | Mtr.3419.1.S1_at | Mtr.10942.1.S1_at | 0.130100769 |
| 238 | Mtr.3419.1.S1_at | Mtr.10967.1.S1_at | 0.130735926 |
| 239 | Mtr.3419.1.S1_at | Mtr.11068.1.S1_at | 0.193641335 |
| 240 | Mtr.3419.1.S1_at | Mtr.11102.1.S1_at | 0.212322729 |
| 241 | Mtr.3419.1.S1_at | Mtr.11119.1.S1_at | 0.188823692 |
| 242 | Mtr.3419.1.S1_at | Mtr.11243.1.S1_at | 0.23238004 |
| 243 | Mtr.40771.1.S1_at | Mtr.11392.1.S1_at | 0.106075295 |
| 244 | Mtr.3419.1.S1_at | Mtr.11444.1.S1_at | 0.196341895 |
| 245 | Mtr.40771.1.S1_at | Mtr.11635.1.S1_at | 0.106767644 |
| 246 | Mtr.40771.1.S1_at | Mtr.11685.1.S1_s_at | 0.13559148 |
| 247 | Mtr.3419.1.S1_at | Mtr.11711.1.S1_at | 0.184229753 |
| 248 | Mtr.3419.1.S1_at | Mtr.1172.1.S1_s_at | 0.127270206 |
| 249 | Mtr.3419.1.S1_at | Mtr.11741.1.S1_at | 0.165437734 |
| 250 | Mtr.3419.1.S1_at | Mtr.11763.1.S1_at | 0.190530873 |
| 251 | Mtr.3419.1.S1_at | Mtr.11811.1.S1_s_at | 0.112209318 |
| 252 | Mtr.3419.1.S1_at | Mtr.11817.1.S1_at | 0.178812779 |
| 253 | Mtr.3419.1.S1_at | Mtr.1193.1.S1_at | 0.171857062 |
| 254 | Mtr.3419.1.S1_at | Mtr.11968.1.S1_at | 0.151661267 |
| 255 | Mtr.3419.1.S1_at | Mtr.11979.1.S1_at | 0.176348531 |
| 256 | Mtr.3419.1.S1_at | Mtr.11989.1.S1_at | 0.153084947 |
| 257 | Mtr.3419.1.S1_at | Mtr.12010.1.S1_at | 0.194798308 |
| 258 | Mtr.40771.1.S1_at | Mtr.12146.1.S1_at | 0.150654592 |
| 259 | Mtr.3419.1.S1_at | Mtr.12148.1.S1_s_at | 0.164814194 |
| 260 | Mtr.3419.1.S1_at | Mtr.12181.1.S1_at | 0.142168047 |
| 261 | Mtr.3419.1.S1_at | Mtr.12318.1.S1_at | 0.124759875 |
| 262 | Mtr.3419.1.S1_at | Mtr.12319.1.S1_at | 0.115162683 |
| 263 | Mtr.3419.1.S1_at | Mtr.12360.1.S1_at | 0.170321641 |
| 264 | Mtr.3419.1.S1_at | Mtr.12397.1.S1_at | 0.101761037 |
| 265 | Mtr.3419.1.S1_at | Mtr.12506.1.S1_x_at | 0.115094698 |
| 266 | Mtr.40771.1.S1_at | Mtr.12512.1.S1_at | 0.127164142 |
| 267 | Mtr.3419.1.S1_at | Mtr.12552.1.S1_at | 0.131831759 |
| 268 | Mtr.3419.1.S1_at | Mtr.12617.1.S1_at | 0.219609206 |
| 269 | Mtr.3419.1.S1_at | Mtr.12625.1.S1_at | 0.13671498 |
| 270 | Mtr.3419.1.S1_at | Mtr.12798.1.S1_at | 0.129881796 |
| 271 | Mtr.3419.1.S1_at | Mtr.12840.1.S1_at | 0.210390677 |
| 272 | Mtr.3419.1.S1_at | Mtr.12905.1.S1_at | 0.223717026 |
| 273 | Mtr.3419.1.S1_at | Mtr.12922.1.S1_at | 0.227538403 |
| 274 | Mtr.3419.1.S1_at | Mtr.12940.1.S1_at | 0.147023555 |
| 275 | Mtr.3419.1.S1_at | Mtr.12951.1.S1_at | 0.13392787 |
| 276 | Mtr.3419.1.S1_at | Mtr.12979.1.S1_at | 0.125928537 |
| 277 | Mtr.3419.1.S1_at | Mtr.13038.1.S1_at | 0.202933071 |
| 278 | Mtr.3419.1.S1_at | Mtr.13089.1.S1_at | 0.120867757 |
| 279 | Mtr.40771.1.S1_at | Mtr.13194.1.S1_s_at | 0.11058467 |
| 280 | Mtr.3419.1.S1_at | Mtr.13209.1.S1_at | 0.143552435 |
| 281 | Mtr.3419.1.S1_at | Mtr.13245.1.S1_at | 0.12236547 |
| 282 | Mtr.3419.1.S1_at | Mtr.13245.1.S1_s_at | 0.140938192 |
| 283 | Mtr.3419.1.S1_at | Mtr.13255.1.S1_at | 0.136174234 |
| 284 | Mtr.40771.1.S1_at | Mtr.13347.1.S1_at | 0.127576451 |
| 285 | Mtr.3419.1.S1_at | Mtr.13440.1.S1_at | 0.158859869 |
| 286 | Mtr.3419.1.S1_at | Mtr.13442.1.S1_at | 0.169576528 |
| 287 | Mtr.3419.1.S1_at | Mtr.13461.1.S1_at | 0.145061959 |
| 288 | Mtr.3419.1.S1_at | Mtr.13528.1.S1_at | 0.178305801 |
| 289 | Mtr.3419.1.S1_at | Mtr.13541.1.S1_at | 0.188012144 |
| 290 | Mtr.3419.1.S1_at | Mtr.13553.1.S1_at | 0.1642059 |
| 291 | Mtr.3419.1.S1_at | Mtr.13614.1.S1_at | 0.155438532 |
| 292 | Mtr.40771.1.S1_at | Mtr.13858.1.S1_at | 0.108106321 |
| 293 | Mtr.3419.1.S1_at | Mtr.14152.1.S1_at | 0.147918261 |
| 294 | Mtr.3419.1.S1_at | Mtr.1424.1.S1_at | 0.199491109 |
| 295 | Mtr.3419.1.S1_at | Mtr.14617.1.S1_at | 0.108677495 |
| 296 | Mtr.40771.1.S1_at | Mtr.14763.1.S1_at | 0.145438993 |
| 297 | Mtr.3419.1.S1_at | Mtr.15021.1.S1_at | 0.154375608 |
| 298 | Mtr.3419.1.S1_at | Mtr.15021.1.S1_x_at | 0.15215453 |
| 299 | Mtr.40771.1.S1_at | Mtr.15101.1.S1_at | 0.124676789 |
| 300 | Mtr.3419.1.S1_at | Mtr.15316.1.S1_s_at | 0.193559059 |
| 301 | Mtr.3419.1.S1_at | Mtr.15326.1.S1_at | 0.199785107 |
| 302 | Mtr.3419.1.S1_at | Mtr.15473.1.S1_at | 0.147373752 |
| 303 | Mtr.3419.1.S1_at | Mtr.15473.1.S1_s_at | 0.152959633 |
| 304 | Mtr.40771.1.S1_at | Mtr.15895.1.S1_s_at | 0.148327784 |
| 305 | Mtr.40771.1.S1_at | Mtr.16190.1.S1_s_at | 0.139899826 |
| 306 | Mtr.3419.1.S1_at | Mtr.16274.1.S1_at | 0.104894653 |
| 307 | Mtr.3419.1.S1_at | Mtr.1670.1.S1_at | 0.159052554 |
| 308 | Mtr.3419.1.S1_at | Mtr.17273.1.S1_s_at | 0.17319298 |
| 309 | Mtr.3419.1.S1_at | Mtr.17285.1.S1_s_at | 0.211293341 |
| 310 | Mtr.40771.1.S1_at | Mtr.1855.1.S1_at | 0.13318849 |
| 311 | Mtr.40771.1.S1_at | Mtr.18858.1.S1_at | 0.11666848 |
| 312 | Mtr.3419.1.S1_at | Mtr.18874.1.S1_at | 0.20482593 |
| 313 | Mtr.3419.1.S1_at | Mtr.19083.1.S1_at | 0.186283775 |
| 314 | Mtr.3419.1.S1_at | Mtr.1918.1.S1_at | 0.185279966 |
| 315 | Mtr.3419.1.S1_at | Mtr.19347.1.S1_at | 0.107539726 |
| 316 | Mtr.3419.1.S1_at | Mtr.19347.1.S1_s_at | 0.114028244 |
| 317 | Mtr.3419.1.S1_at | Mtr.19408.1.S1_at | 0.185951335 |
| 318 | Mtr.3419.1.S1_at | Mtr.19547.1.S1_s_at | 0.183349363 |
| 319 | Mtr.40771.1.S1_at | Mtr.20470.1.S1_at | 0.120709388 |
| 320 | Mtr.40771.1.S1_at | Mtr.20478.1.S1_at | 0.119257956 |
| 321 | Mtr.3419.1.S1_at | Mtr.2062.1.S1_at | 0.211892509 |
| 322 | Mtr.40771.1.S1_at | Mtr.20650.1.S1_at | 0.12303107 |
| 323 | Mtr.3419.1.S1_at | Mtr.20901.1.S1_s_at | 0.146788888 |
| 324 | Mtr.3419.1.S1_at | Mtr.21025.1.S1_at | 0.114018697 |
| 325 | Mtr.3419.1.S1_at | Mtr.21518.1.S1_s_at | 0.155096603 |
| 326 | Mtr.3419.1.S1_at | Mtr.21615.1.S1_at | 0.174488407 |
| 327 | Mtr.3419.1.S1_at | Mtr.21739.1.S1_at | 0.11464767 |
| 328 | Mtr.3419.1.S1_at | Mtr.21815.1.S1_s_at | 0.149192551 |
| 329 | Mtr.3419.1.S1_at | Mtr.21838.1.S1_at | 0.154654318 |
| 330 | Mtr.40771.1.S1_at | Mtr.21924.1.S1_at | 0.100540722 |
| 331 | Mtr.3419.1.S1_at | Mtr.21944.1.S1_s_at | 0.183651427 |
| 332 | Mtr.3419.1.S1_at | Mtr.22141.1.S1_at | 0.186014683 |
| 333 | Mtr.40771.1.S1_at | Mtr.23609.1.S1_at | 0.130475149 |
| 334 | Mtr.3419.1.S1_at | Mtr.2380.1.S1_at | 0.106586416 |
| 335 | Mtr.3419.1.S1_at | Mtr.23850.1.S1_at | 0.16713584 |
| 336 | Mtr.3419.1.S1_at | Mtr.24085.1.S1_at | 0.165664899 |
| 337 | Mtr.3419.1.S1_at | Mtr.24367.1.S1_at | 0.135391397 |
| 338 | Mtr.3419.1.S1_at | Mtr.25649.1.S1_at | 0.116649759 |
| 339 | Mtr.3419.1.S1_at | Mtr.25710.1.S1_at | 0.124712278 |
| 340 | Mtr.3419.1.S1_at | Mtr.25980.1.S1_s_at | 0.15252651 |
| 341 | Mtr.3419.1.S1_at | Mtr.26245.1.S1_at | 0.113983512 |
| 342 | Mtr.3419.1.S1_at | Mtr.26672.1.S1_s_at | 0.196118215 |
| 343 | Mtr.40771.1.S1_at | Mtr.26713.1.S1_s_at | 0.116825916 |
| 344 | Mtr.40771.1.S1_at | Mtr.26950.1.S1_at | 0.151352865 |
| 345 | Mtr.3419.1.S1_at | Mtr.27086.1.S1_at | 0.1806386 |
| 346 | Mtr.3419.1.S1_at | Mtr.27132.1.S1_s_at | 0.113143105 |
| 347 | Mtr.3419.1.S1_at | Mtr.27277.1.S1_at | 0.111750367 |
| 348 | Mtr.40771.1.S1_at | Mtr.27288.1.S1_at | 0.113577563 |
| 349 | Mtr.3419.1.S1_at | Mtr.27305.1.S1_at | 0.117234835 |
| 350 | Mtr.3419.1.S1_at | Mtr.27391.1.S1_at | 0.134733155 |
| 351 | Mtr.3419.1.S1_at | Mtr.27432.1.S1_at | 0.154925498 |
| 352 | Mtr.3419.1.S1_at | Mtr.27440.1.S1_at | 0.198431964 |
| 353 | Mtr.3419.1.S1_at | Mtr.27646.1.S1_s_at | 0.147313431 |
| 354 | Mtr.3419.1.S1_at | Mtr.27823.1.S1_at | 0.177072901 |
| 355 | Mtr.3419.1.S1_at | Mtr.27854.1.S1_s_at | 0.145139861 |
| 356 | Mtr.3419.1.S1_at | Mtr.27925.1.S1_at | 0.109502764 |
| 357 | Mtr.40771.1.S1_at | Mtr.28091.1.S1_at | 0.145853675 |
| 358 | Mtr.3419.1.S1_at | Mtr.28449.1.S1_at | 0.118921793 |
| 359 | Mtr.3419.1.S1_at | Mtr.28450.1.S1_at | 0.120775192 |
| 360 | Mtr.3419.1.S1_at | Mtr.28459.1.S1_at | 0.101043719 |
| 361 | Mtr.3419.1.S1_at | Mtr.28551.1.S1_at | 0.103488877 |
| 362 | Mtr.3419.1.S1_at | Mtr.28655.1.S1_at | 0.102212978 |
| 363 | Mtr.3419.1.S1_at | Mtr.28661.1.S1_at | 0.213415818 |
| 364 | Mtr.3419.1.S1_at | Mtr.28734.1.S1_at | 0.177093136 |
| 365 | Mtr.40771.1.S1_at | Mtr.28741.1.S1_at | 0.113625394 |
| 366 | Mtr.3419.1.S1_at | Mtr.2878.1.S1_x_at | 0.126151759 |
| 367 | Mtr.3419.1.S1_at | Mtr.29178.1.S1_at | 0.200854028 |
| 368 | Mtr.3419.1.S1_at | Mtr.29219.1.S1_at | 0.103716128 |
| 369 | Mtr.3419.1.S1_at | Mtr.2925.1.S1_at | 0.175194761 |
| 370 | Mtr.40771.1.S1_at | Mtr.29288.1.S1_at | 0.100877341 |
| 371 | Mtr.3419.1.S1_at | Mtr.29388.1.S1_s_at | 0.142222604 |
| 372 | Mtr.3419.1.S1_at | Mtr.29389.1.S1_at | 0.193470684 |
| 373 | Mtr.3419.1.S1_at | Mtr.29465.1.S1_at | 0.170750762 |
| 374 | Mtr.3419.1.S1_at | Mtr.29496.1.S1_at | 0.125092899 |
| 375 | Mtr.3419.1.S1_at | Mtr.29885.1.S1_at | 0.132569451 |
| 376 | Mtr.3419.1.S1_at | Mtr.30038.1.S1_at | 0.202250615 |
| 377 | Mtr.3419.1.S1_at | Mtr.30482.1.S1_at | 0.180384394 |
| 378 | Mtr.40771.1.S1_at | Mtr.30492.1.S1_at | 0.10253052 |
| 379 | Mtr.3419.1.S1_at | Mtr.3071.1.S1_at | 0.13731922 |
| 380 | Mtr.3419.1.S1_at | Mtr.30722.1.S1_at | 0.169056511 |
| 381 | Mtr.40771.1.S1_at | Mtr.30771.1.S1_at | 0.115698856 |
| 382 | Mtr.3419.1.S1_at | Mtr.31155.1.S1_at | 0.153788198 |
| 383 | Mtr.3419.1.S1_at | Mtr.3117.1.S1_at | 0.183556693 |
| 384 | Mtr.3419.1.S1_at | Mtr.31302.1.S1_at | 0.13486938 |
| 385 | Mtr.40771.1.S1_at | Mtr.31313.1.S1_at | 0.124192111 |
| 386 | Mtr.3419.1.S1_at | Mtr.31336.1.S1_at | 0.195689083 |
| 387 | Mtr.3419.1.S1_at | Mtr.31576.1.S1_at | 0.215531082 |
| 388 | Mtr.40771.1.S1_at | Mtr.32053.1.S1_at | 0.123215923 |
| 389 | Mtr.3419.1.S1_at | Mtr.32162.1.S1_at | 0.163076298 |
| 390 | Mtr.40771.1.S1_at | Mtr.32412.1.S1_at | 0.128894585 |
| 391 | Mtr.3419.1.S1_at | Mtr.32528.1.S1_s_at | 0.155391875 |
| 392 | Mtr.3419.1.S1_at | Mtr.32558.1.S1_at | 0.115176613 |
| 393 | Mtr.3419.1.S1_at | Mtr.32569.1.S1_at | 0.172009188 |
| 394 | Mtr.3419.1.S1_at | Mtr.32581.1.S1_at | 0.163873259 |
| 395 | Mtr.40771.1.S1_at | Mtr.32653.1.S1_at | 0.137534962 |
| 396 | Mtr.3419.1.S1_at | Mtr.32703.1.S1_at | 0.212756778 |
| 397 | Mtr.3419.1.S1_at | Mtr.32796.1.S1_at | 0.103122084 |
| 398 | Mtr.3419.1.S1_at | Mtr.32802.1.S1_at | 0.134369572 |
| 399 | Mtr.3419.1.S1_at | Mtr.32824.1.S1_at | 0.145783938 |
| 400 | Mtr.3419.1.S1_at | Mtr.32925.1.S1_at | 0.136722719 |
| 401 | Mtr.3419.1.S1_at | Mtr.32939.1.S1_at | 0.18125761 |
| 402 | Mtr.3419.1.S1_at | Mtr.33282.1.S1_at | 0.125885256 |
| 403 | Mtr.3419.1.S1_at | Mtr.33398.1.S1_s_at | 0.102888043 |
| 404 | Mtr.40771.1.S1_at | Mtr.33424.1.S1_at | 0.11861534 |
| 405 | Mtr.3419.1.S1_at | Mtr.33537.1.S1_at | 0.139402183 |
| 406 | Mtr.3419.1.S1_at | Mtr.33639.1.S1_at | 0.123749084 |
| 407 | Mtr.3419.1.S1_at | Mtr.33654.1.S1_at | 0.152527547 |
| 408 | Mtr.3419.1.S1_at | Mtr.33678.1.S1_at | 0.114340562 |
| 409 | Mtr.3419.1.S1_at | Mtr.33745.1.S1_s_at | 0.194818491 |
| 410 | Mtr.3419.1.S1_at | Mtr.33795.1.S1_at | 0.133853169 |
| 411 | Mtr.3419.1.S1_at | Mtr.33831.1.S1_at | 0.155727263 |
| 412 | Mtr.40771.1.S1_at | Mtr.33878.1.S1_at | 0.134756205 |
| 413 | Mtr.40771.1.S1_at | Mtr.33909.1.S1_at | 0.131246953 |
| 414 | Mtr.40771.1.S1_at | Mtr.35998.1.S1_at | 0.133278289 |
| 415 | Mtr.40771.1.S1_at | Mtr.38022.1.S1_at | 0.100905773 |
| 416 | Mtr.40771.1.S1_at | Mtr.38041.1.S1_at | 0.136219578 |
| 417 | Mtr.40771.1.S1_at | Mtr.38139.1.S1_s_at | 0.128531211 |
| 418 | Mtr.40771.1.S1_at | Mtr.38518.1.S1_at | 0.112302044 |
| 419 | Mtr.40771.1.S1_at | Mtr.38758.1.S1_at | 0.126717099 |
| 420 | Mtr.40771.1.S1_at | Mtr.38988.1.S1_at | 0.111117841 |
| 421 | Mtr.40771.1.S1_at | Mtr.39077.1.S1_at | 0.129264211 |
| 422 | Mtr.40771.1.S1_at | Mtr.39186.1.S1_at | 0.142885268 |
| 423 | Mtr.40771.1.S1_at | Mtr.40034.1.S1_at | 0.139565015 |
| 424 | Mtr.40771.1.S1_at | Mtr.40484.1.S1_at | 0.101275487 |
| 425 | Mtr.40771.1.S1_at | Mtr.40657.1.S1_at | 0.143758467 |
| 426 | Mtr.40771.1.S1_at | Mtr.40749.1.S1_at | 0.14132258 |
| 427 | Mtr.18323.1.S1_at | Mtr.10008.1.S1_at | 0.14375014 |
| 428 | Mtr.18323.1.S1_at | Mtr.10022.1.S1_at | 0.217881965 |
| 429 | Mtr.18323.1.S1_at | Mtr.10028.1.S1_at | 0.189192054 |
| 430 | Mtr.18323.1.S1_at | Mtr.10044.1.S1_at | 0.23942249 |
| 431 | Mtr.18323.1.S1_at | Mtr.10059.1.S1_at | 0.115798337 |
| 432 | Mtr.34174.1.S1_at | Mtr.10071.1.S1_at | 0.10600922 |
| 433 | Mtr.34174.1.S1_at | Mtr.10071.1.S1_s_at | 0.117905723 |
| 434 | Mtr.18323.1.S1_at | Mtr.10082.1.S1_at | 0.173579964 |
| 435 | Mtr.18323.1.S1_at | Mtr.10091.1.S1_at | 0.140716125 |
| 436 | Mtr.18323.1.S1_at | Mtr.10113.1.S1_at | 0.250659876 |
| 437 | Mtr.18323.1.S1_at | Mtr.10115.1.S1_at | 0.183505795 |
| 438 | Mtr.18323.1.S1_at | Mtr.10132.1.S1_at | 0.153177081 |
| 439 | Mtr.18323.1.S1_at | Mtr.10153.1.S1_s_at | 0.21079818 |
| 440 | Mtr.18323.1.S1_at | Mtr.10196.1.S1_at | 0.163087794 |
| 441 | Mtr.18323.1.S1_at | Mtr.10199.1.S1_at | 0.19947423 |
| 442 | Mtr.18323.1.S1_at | Mtr.10205.1.S1_at | 0.189697754 |
| 443 | Mtr.18323.1.S1_at | Mtr.10266.1.S1_at | 0.116435413 |
| 444 | Mtr.18323.1.S1_at | Mtr.1027.1.S1_at | 0.190533401 |
| 445 | Mtr.18323.1.S1_at | Mtr.10275.1.S1_s_at | 0.156463178 |
| 446 | Mtr.34174.1.S1_at | Mtr.10291.1.S1_at | 0.105892647 |
| 447 | Mtr.18323.1.S1_at | Mtr.10294.1.S1_at | 0.208578093 |
| 448 | Mtr.18323.1.S1_at | Mtr.10330.1.S1_s_at | 0.219205726 |
| 449 | Mtr.18323.1.S1_at | Mtr.10339.1.S1_at | 0.204530079 |
| 450 | Mtr.18323.1.S1_at | Mtr.10340.1.S1_at | 0.205258419 |
| 451 | Mtr.34174.1.S1_at | Mtr.10344.1.S1_at | 0.106560461 |
| 452 | Mtr.18323.1.S1_at | Mtr.10349.1.S1_at | 0.130181889 |
| 453 | Mtr.18323.1.S1_at | Mtr.10359.1.S1_at | 0.202414339 |
| 454 | Mtr.18323.1.S1_at | Mtr.10376.1.S1_at | 0.158900313 |
| 455 | Mtr.18323.1.S1_at | Mtr.1038.1.S1_at | 0.179988548 |
| 456 | Mtr.18323.1.S1_at | Mtr.1039.1.S1_at | 0.113587055 |
| 457 | Mtr.18323.1.S1_at | Mtr.10394.1.S1_at | 0.177272993 |
| 458 | Mtr.18323.1.S1_at | Mtr.10419.1.S1_at | 0.105755767 |
| 459 | Mtr.18323.1.S1_at | Mtr.10436.1.S1_at | 0.15317632 |
| 460 | Mtr.18323.1.S1_at | Mtr.10439.1.S1_at | 0.152598875 |
| 461 | Mtr.18323.1.S1_at | Mtr.10445.1.S1_at | 0.237327171 |
| 462 | Mtr.18323.1.S1_at | Mtr.10455.1.S1_at | 0.244437492 |
| 463 | Mtr.18323.1.S1_at | Mtr.10489.1.S1_s_at | 0.164650473 |
| 464 | Mtr.18323.1.S1_at | Mtr.10495.1.S1_at | 0.208983787 |
| 465 | Mtr.18323.1.S1_at | Mtr.10526.1.S1_at | 0.165789323 |
| 466 | Mtr.18323.1.S1_at | Mtr.10548.1.S1_at | 0.215554903 |
| 467 | Mtr.18323.1.S1_at | Mtr.10561.1.S1_at | 0.239973431 |
| 468 | Mtr.34174.1.S1_at | Mtr.10591.1.S1_at | 0.119614928 |
| 469 | Mtr.18323.1.S1_at | Mtr.10595.1.S1_at | 0.229771118 |
| 470 | Mtr.18323.1.S1_at | Mtr.10602.1.S1_at | 0.155006766 |
| 471 | Mtr.18323.1.S1_at | Mtr.10605.1.S1_at | 0.128224624 |
| 472 | Mtr.18323.1.S1_at | Mtr.10606.1.S1_at | 0.151966498 |
| 473 | Mtr.18323.1.S1_at | Mtr.10625.1.S1_at | 0.16977398 |
| 474 | Mtr.18323.1.S1_at | Mtr.10635.1.S1_at | 0.130804729 |
| 475 | Mtr.18323.1.S1_at | Mtr.10641.1.S1_at | 0.218764503 |
| 476 | Mtr.18323.1.S1_at | Mtr.10656.1.S1_at | 0.128627862 |
| 477 | Mtr.18323.1.S1_at | Mtr.10668.1.S1_at | 0.12785497 |
| 478 | Mtr.18323.1.S1_at | Mtr.10679.1.S1_at | 0.218263466 |
| 479 | Mtr.18323.1.S1_at | Mtr.10686.1.S1_at | 0.104259499 |
| 480 | Mtr.18323.1.S1_at | Mtr.10687.1.S1_at | 0.225327056 |
| 481 | Mtr.18323.1.S1_at | Mtr.10722.1.S1_at | 0.213496397 |
| 482 | Mtr.18323.1.S1_at | Mtr.10754.1.S1_at | 0.234034214 |
| 483 | Mtr.34174.1.S1_at | Mtr.10761.1.S1_s_at | 0.105805229 |
| 484 | Mtr.18323.1.S1_at | Mtr.10811.1.S1_at | 0.142549357 |
| 485 | Mtr.18323.1.S1_at | Mtr.10814.1.S1_at | 0.126288949 |
| 486 | Mtr.18323.1.S1_at | Mtr.10818.1.S1_at | 0.128993524 |
| 487 | Mtr.18323.1.S1_at | Mtr.1082.1.S1_at | 0.197424046 |
| 488 | Mtr.18323.1.S1_at | Mtr.10829.1.S1_at | 0.124777924 |
| 489 | Mtr.18323.1.S1_at | Mtr.10861.1.S1_at | 0.202565341 |
| 490 | Mtr.18323.1.S1_at | Mtr.10872.1.S1_s_at | 0.198946486 |
| 491 | Mtr.18323.1.S1_at | Mtr.10877.1.S1_at | 0.152060311 |
| 492 | Mtr.18323.1.S1_at | Mtr.10897.1.S1_at | 0.108843941 |
| 493 | Mtr.18323.1.S1_at | Mtr.10919.1.S1_at | 0.172497523 |
| 494 | Mtr.18323.1.S1_at | Mtr.10937.1.S1_at | 0.181659933 |
| 495 | Mtr.34174.1.S1_at | Mtr.10941.1.S1_at | 0.117825916 |
| 496 | Mtr.18323.1.S1_at | Mtr.10949.1.S1_at | 0.197414768 |
| 497 | Mtr.18323.1.S1_at | Mtr.10960.1.S1_at | 0.18047496 |
| 498 | Mtr.18323.1.S1_at | Mtr.1097.1.S1_at | 0.233782658 |
| 499 | Mtr.34174.1.S1_at | Mtr.10978.1.S1_s_at | 0.123101017 |
| 500 | Mtr.18323.1.S1_at | Mtr.10991.1.S1_at | 0.164869231 |
| 501 | Mtr.18323.1.S1_at | Mtr.10994.1.S1_at | 0.164502737 |
| 502 | Mtr.18323.1.S1_at | Mtr.11027.1.S1_at | 0.240923453 |
| 503 | Mtr.18323.1.S1_at | Mtr.11028.1.S1_at | 0.108996703 |
| 504 | Mtr.18323.1.S1_at | Mtr.11044.1.S1_at | 0.113962307 |
| 505 | Mtr.18323.1.S1_at | Mtr.11046.1.S1_at | 0.116943779 |
| 506 | Mtr.18323.1.S1_at | Mtr.11054.1.S1_at | 0.163895684 |
| 507 | Mtr.18323.1.S1_at | Mtr.11070.1.S1_s_at | 0.208497189 |
| 508 | Mtr.18323.1.S1_at | Mtr.11079.1.S1_at | 0.166187549 |
| 509 | Mtr.18323.1.S1_at | Mtr.11087.1.S1_at | 0.10317726 |
| 510 | Mtr.18323.1.S1_at | Mtr.11105.1.S1_at | 0.105994657 |
| 511 | Mtr.18323.1.S1_at | Mtr.11133.1.S1_at | 0.145866045 |
| 512 | Mtr.18323.1.S1_at | Mtr.1115.1.S1_at | 0.142983306 |
| 513 | Mtr.18323.1.S1_at | Mtr.11169.1.S1_at | 0.163040718 |
| 514 | Mtr.18323.1.S1_at | Mtr.11193.1.S1_at | 0.192661083 |
| 515 | Mtr.18323.1.S1_at | Mtr.11199.1.S1_at | 0.230563626 |
| 516 | Mtr.18323.1.S1_at | Mtr.11209.1.S1_at | 0.118893949 |
| 517 | Mtr.18323.1.S1_at | Mtr.11213.1.S1_at | 0.188745905 |
| 518 | Mtr.18323.1.S1_at | Mtr.11215.1.S1_at | 0.155533466 |
| 519 | Mtr.18323.1.S1_at | Mtr.11221.1.S1_at | 0.138538578 |
| 520 | Mtr.18323.1.S1_at | Mtr.11250.1.S1_at | 0.159387379 |
| 521 | Mtr.34174.1.S1_at | Mtr.11294.1.S1_at | 0.103274833 |
| 522 | Mtr.18323.1.S1_at | Mtr.11296.1.S1_at | 0.190974388 |
| 523 | Mtr.18323.1.S1_at | Mtr.11303.1.S1_at | 0.178356322 |
| 524 | Mtr.18323.1.S1_at | Mtr.11312.1.S1_at | 0.110216743 |
| 525 | Mtr.18323.1.S1_at | Mtr.11318.1.S1_at | 0.141052512 |
| 526 | Mtr.18323.1.S1_at | Mtr.11323.1.S1_at | 0.208467995 |
| 527 | Mtr.18323.1.S1_at | Mtr.11339.1.S1_at | 0.246577668 |
| 528 | Mtr.18323.1.S1_at | Mtr.11358.1.S1_at | 0.179373698 |
| 529 | Mtr.18323.1.S1_at | Mtr.11370.1.S1_at | 0.208095137 |
| 530 | Mtr.18323.1.S1_at | Mtr.11388.1.S1_at | 0.249176304 |
| 531 | Mtr.18323.1.S1_at | Mtr.11433.1.S1_at | 0.12114662 |
| 532 | Mtr.18323.1.S1_at | Mtr.11446.1.S1_at | 0.240140924 |
| 533 | Mtr.18323.1.S1_at | Mtr.11446.1.S1_s_at | 0.180124716 |
| 534 | Mtr.18323.1.S1_at | Mtr.11447.1.S1_at | 0.172735101 |
| 535 | Mtr.18323.1.S1_at | Mtr.115.1.S1_at | 0.113323426 |
| 536 | Mtr.18323.1.S1_at | Mtr.11512.1.S1_at | 0.116208251 |
| 537 | Mtr.18323.1.S1_at | Mtr.11527.1.S1_at | 0.18973096 |
| 538 | Mtr.18323.1.S1_at | Mtr.11557.1.S1_at | 0.242382666 |
| 539 | Mtr.18323.1.S1_at | Mtr.11560.1.S1_s_at | 0.184688331 |
| 540 | Mtr.18323.1.S1_at | Mtr.11567.1.S1_at | 0.217383594 |
| 541 | Mtr.18323.1.S1_at | Mtr.11604.1.S1_at | 0.216600598 |
| 542 | Mtr.18323.1.S1_at | Mtr.11605.1.S1_s_at | 0.124657709 |
| 543 | Mtr.18323.1.S1_at | Mtr.11606.1.S1_at | 0.200282419 |
| 544 | Mtr.18323.1.S1_at | Mtr.11607.1.S1_at | 0.200256235 |
| 545 | Mtr.18323.1.S1_at | Mtr.11611.1.S1_at | 0.146187823 |
| 546 | Mtr.18323.1.S1_at | Mtr.11624.1.S1_at | 0.108695098 |
| 547 | Mtr.18323.1.S1_at | Mtr.11652.1.S1_at | 0.13216729 |
| 548 | Mtr.18323.1.S1_at | Mtr.11655.1.S1_at | 0.239822243 |
| 549 | Mtr.18323.1.S1_at | Mtr.11656.1.S1_at | 0.110114069 |
| 550 | Mtr.18323.1.S1_at | Mtr.11689.1.S1_at | 0.118484097 |
| 551 | Mtr.18323.1.S1_at | Mtr.1169.1.S1_s_at | 0.111710895 |
| 552 | Mtr.18323.1.S1_at | Mtr.11695.1.S1_at | 0.185691372 |
| 553 | Mtr.18323.1.S1_at | Mtr.11696.1.S1_at | 0.108548716 |
| 554 | Mtr.18323.1.S1_at | Mtr.11707.1.S1_at | 0.13553354 |
| 555 | Mtr.18323.1.S1_at | Mtr.11764.1.S1_at | 0.212965896 |
| 556 | Mtr.18323.1.S1_at | Mtr.11799.1.S1_at | 0.1170088 |
| 557 | Mtr.18323.1.S1_at | Mtr.11802.1.S1_at | 0.115840457 |
| 558 | Mtr.18323.1.S1_at | Mtr.11808.1.S1_at | 0.121072201 |
| 559 | Mtr.18323.1.S1_at | Mtr.11852.1.S1_at | 0.209570382 |
| 560 | Mtr.18323.1.S1_at | Mtr.11854.1.S1_at | 0.155787992 |
| 561 | Mtr.18323.1.S1_at | Mtr.11864.1.S1_at | 0.191642348 |
| 562 | Mtr.18323.1.S1_at | Mtr.11899.1.S1_at | 0.188048895 |
| 563 | Mtr.18323.1.S1_at | Mtr.11904.1.S1_at | 0.176311885 |
| 564 | Mtr.18323.1.S1_at | Mtr.11908.1.S1_at | 0.22465807 |
| 565 | Mtr.18323.1.S1_at | Mtr.11908.1.S1_s_at | 0.247562953 |
| 566 | Mtr.18323.1.S1_at | Mtr.11912.1.S1_at | 0.216394622 |
| 567 | Mtr.18323.1.S1_at | Mtr.11925.1.S1_at | 0.103529266 |
| 568 | Mtr.18323.1.S1_at | Mtr.11943.1.S1_at | 0.123063183 |
| 569 | Mtr.18323.1.S1_at | Mtr.11954.1.S1_at | 0.134285974 |
| 570 | Mtr.18323.1.S1_at | Mtr.11956.1.S1_at | 0.213120768 |
| 571 | Mtr.18323.1.S1_at | Mtr.11962.1.S1_at | 0.241452591 |
| 572 | Mtr.18323.1.S1_at | Mtr.11965.1.S1_at | 0.158433388 |
| 573 | Mtr.18323.1.S1_at | Mtr.11969.1.S1_at | 0.176389701 |
| 574 | Mtr.18323.1.S1_at | Mtr.11986.1.S1_at | 0.162101434 |
| 575 | Mtr.18323.1.S1_at | Mtr.12.1.S1_at | 0.139347913 |
| 576 | Mtr.18323.1.S1_at | Mtr.12027.1.S1_at | 0.141407924 |
| 577 | Mtr.18323.1.S1_at | Mtr.12034.1.S1_at | 0.194075383 |
| 578 | Mtr.18323.1.S1_at | Mtr.12048.1.S1_at | 0.102093018 |
| 579 | Mtr.18323.1.S1_at | Mtr.12054.1.S1_at | 0.125662202 |
| 580 | Mtr.18323.1.S1_at | Mtr.12057.1.S1_at | 0.229739654 |
| 581 | Mtr.18323.1.S1_at | Mtr.12058.1.S1_at | 0.183765294 |
| 582 | Mtr.18323.1.S1_at | Mtr.12079.1.S1_at | 0.194098534 |
| 583 | Mtr.18323.1.S1_at | Mtr.12094.1.S1_at | 0.117748724 |
| 584 | Mtr.18323.1.S1_at | Mtr.12121.1.S1_at | 0.181970877 |
| 585 | Mtr.18323.1.S1_at | Mtr.12136.1.S1_at | 0.212408217 |
| 586 | Mtr.18323.1.S1_at | Mtr.12154.1.S1_at | 0.136792546 |
| 587 | Mtr.18323.1.S1_at | Mtr.12156.1.S1_at | 0.13790226 |
| 588 | Mtr.18323.1.S1_at | Mtr.12158.1.S1_at | 0.153124816 |
| 589 | Mtr.18323.1.S1_at | Mtr.12192.1.S1_at | 0.186367456 |
| 590 | Mtr.18323.1.S1_at | Mtr.12219.1.S1_at | 0.220988734 |
| 591 | Mtr.18323.1.S1_at | Mtr.12224.1.S1_at | 0.116386959 |
| 592 | Mtr.18323.1.S1_at | Mtr.12232.1.S1_at | 0.23265467 |
| 593 | Mtr.18323.1.S1_at | Mtr.12232.1.S1_x_at | 0.22744962 |
| 594 | Mtr.18323.1.S1_at | Mtr.12238.1.S1_at | 0.129489873 |
| 595 | Mtr.18323.1.S1_at | Mtr.12239.1.S1_x_at | 0.162842715 |
| 596 | Mtr.18323.1.S1_at | Mtr.12244.1.S1_s_at | 0.20497851 |
| 597 | Mtr.18323.1.S1_at | Mtr.12261.1.S1_s_at | 0.23092413 |
| 598 | Mtr.18323.1.S1_at | Mtr.12280.1.S1_at | 0.211520122 |
| 599 | Mtr.18323.1.S1_at | Mtr.12289.1.S1_at | 0.213831349 |
| 600 | Mtr.18323.1.S1_at | Mtr.12293.1.S1_at | 0.168979517 |
| 601 | Mtr.18323.1.S1_at | Mtr.12296.1.S1_at | 0.148352897 |
| 602 | Mtr.34174.1.S1_at | Mtr.12327.1.S1_s_at | 0.10202158 |
| 603 | Mtr.18323.1.S1_at | Mtr.1236.1.S1_at | 0.152828224 |
| 604 | Mtr.18323.1.S1_at | Mtr.12378.1.S1_at | 0.11519695 |
| 605 | Mtr.18323.1.S1_at | Mtr.12394.1.S1_at | 0.234169524 |
| 606 | Mtr.18323.1.S1_at | Mtr.12423.1.S1_at | 0.124106792 |
| 607 | Mtr.18323.1.S1_at | Mtr.12442.1.S1_at | 0.138319016 |
| 608 | Mtr.18323.1.S1_at | Mtr.12443.1.S1_at | 0.155629807 |
| 609 | Mtr.34174.1.S1_at | Mtr.12446.1.S1_at | 0.127150357 |
| 610 | Mtr.18323.1.S1_at | Mtr.12453.1.S1_at | 0.239717156 |
| 611 | Mtr.18323.1.S1_at | Mtr.12487.1.S1_at | 0.10233394 |
| 612 | Mtr.18323.1.S1_at | Mtr.12491.1.S1_at | 0.219804701 |
| 613 | Mtr.18323.1.S1_at | Mtr.12494.1.S1_at | 0.114964178 |
| 614 | Mtr.18323.1.S1_at | Mtr.12551.1.S1_at | 0.232932768 |
| 615 | Mtr.18323.1.S1_at | Mtr.12558.1.S1_at | 0.168350363 |
| 616 | Mtr.18323.1.S1_at | Mtr.12581.1.S1_s_at | 0.166697525 |
| 617 | Mtr.18323.1.S1_at | Mtr.12589.1.S1_at | 0.164069819 |
| 618 | Mtr.18323.1.S1_at | Mtr.12591.1.S1_at | 0.260083209 |
| 619 | Mtr.18323.1.S1_at | Mtr.12613.1.S1_at | 0.109202358 |
| 620 | Mtr.18323.1.S1_at | Mtr.12630.1.S1_at | 0.165461245 |
| 621 | Mtr.18323.1.S1_at | Mtr.12645.1.S1_at | 0.22161943 |
| 622 | Mtr.18323.1.S1_at | Mtr.12648.1.S1_at | 0.143659031 |
| 623 | Mtr.18323.1.S1_at | Mtr.12665.1.S1_at | 0.135613993 |
| 624 | Mtr.18323.1.S1_at | Mtr.12683.1.S1_at | 0.179681995 |
| 625 | Mtr.18323.1.S1_at | Mtr.12684.1.S1_at | 0.195938156 |
| 626 | Mtr.18323.1.S1_at | Mtr.12686.1.S1_at | 0.181985692 |
| 627 | Mtr.18323.1.S1_at | Mtr.12695.1.S1_at | 0.119494534 |
| 628 | Mtr.18323.1.S1_at | Mtr.12719.1.S1_at | 0.174047568 |
| 629 | Mtr.18323.1.S1_at | Mtr.12728.1.S1_at | 0.172379282 |
| 630 | Mtr.18323.1.S1_at | Mtr.12745.1.S1_at | 0.13210093 |
| 631 | Mtr.18323.1.S1_at | Mtr.12763.1.S1_at | 0.191751862 |
| 632 | Mtr.18323.1.S1_at | Mtr.12766.1.S1_at | 0.141157083 |
| 633 | Mtr.18323.1.S1_at | Mtr.12776.1.S1_at | 0.166785341 |
| 634 | Mtr.18323.1.S1_at | Mtr.12809.1.S1_at | 0.107916932 |
| 635 | Mtr.18323.1.S1_at | Mtr.12810.1.S1_at | 0.114263719 |
| 636 | Mtr.18323.1.S1_at | Mtr.12824.1.S1_at | 0.134638144 |
| 637 | Mtr.18323.1.S1_at | Mtr.12839.1.S1_at | 0.219069944 |
| 638 | Mtr.18323.1.S1_at | Mtr.12876.1.S1_at | 0.103601424 |
| 639 | Mtr.18323.1.S1_at | Mtr.12891.1.S1_at | 0.175537071 |
| 640 | Mtr.18323.1.S1_at | Mtr.12901.1.S1_at | 0.165441825 |
| 641 | Mtr.18323.1.S1_at | Mtr.12911.1.S1_at | 0.161823028 |
| 642 | Mtr.34174.1.S1_at | Mtr.12915.1.S1_at | 0.14130449 |
| 643 | Mtr.18323.1.S1_at | Mtr.12936.1.S1_at | 0.212316976 |
| 644 | Mtr.18323.1.S1_at | Mtr.12955.1.S1_at | 0.131946801 |
| 645 | Mtr.18323.1.S1_at | Mtr.12982.1.S1_at | 0.175331367 |
| 646 | Mtr.18323.1.S1_at | Mtr.12990.1.S1_at | 0.223892716 |
| 647 | Mtr.18323.1.S1_at | Mtr.12997.1.S1_at | 0.220962557 |
| 648 | Mtr.18323.1.S1_at | Mtr.12999.1.S1_at | 0.126861716 |
| 649 | Mtr.18323.1.S1_at | Mtr.13015.1.S1_at | 0.140081246 |
| 650 | Mtr.18323.1.S1_at | Mtr.13025.1.S1_at | 0.185231411 |
| 651 | Mtr.18323.1.S1_at | Mtr.13025.1.S1_s_at | 0.105954128 |
| 652 | Mtr.18323.1.S1_at | Mtr.13047.1.S1_at | 0.193444732 |
| 653 | Mtr.18323.1.S1_at | Mtr.13085.1.S1_at | 0.136714609 |
| 654 | Mtr.18323.1.S1_at | Mtr.13101.1.S1_at | 0.158824747 |
| 655 | Mtr.18323.1.S1_at | Mtr.13162.1.S1_s_at | 0.100468102 |
| 656 | Mtr.18323.1.S1_at | Mtr.13195.1.S1_at | 0.173737292 |
| 657 | Mtr.18323.1.S1_at | Mtr.13197.1.S1_at | 0.18407027 |
| 658 | Mtr.18323.1.S1_at | Mtr.13231.1.S1_s_at | 0.19906726 |
| 659 | Mtr.18323.1.S1_at | Mtr.13268.1.S1_at | 0.100147791 |
| 660 | Mtr.18323.1.S1_at | Mtr.13289.1.S1_at | 0.241152872 |
| 661 | Mtr.18323.1.S1_at | Mtr.1331.1.S1_at | 0.137135543 |
| 662 | Mtr.18323.1.S1_at | Mtr.13331.1.S1_at | 0.146123916 |
| 663 | Mtr.18323.1.S1_at | Mtr.13335.1.S1_at | 0.163673223 |
| 664 | Mtr.18323.1.S1_at | Mtr.13337.1.S1_at | 0.15526153 |
| 665 | Mtr.18323.1.S1_at | Mtr.13342.1.S1_at | 0.214628351 |
| 666 | Mtr.18323.1.S1_at | Mtr.13373.1.S1_at | 0.145027117 |
| 667 | Mtr.18323.1.S1_at | Mtr.13383.1.S1_at | 0.109704224 |
| 668 | Mtr.18323.1.S1_at | Mtr.13387.1.S1_at | 0.168238299 |
| 669 | Mtr.18323.1.S1_at | Mtr.13398.1.S1_at | 0.174029198 |
| 670 | Mtr.18323.1.S1_at | Mtr.13406.1.S1_at | 0.13976313 |
| 671 | Mtr.18323.1.S1_at | Mtr.13408.1.S1_at | 0.148708617 |
| 672 | Mtr.18323.1.S1_at | Mtr.13428.1.S1_at | 0.19519806 |
| 673 | Mtr.18323.1.S1_at | Mtr.13447.1.S1_at | 0.194957459 |
| 674 | Mtr.18323.1.S1_at | Mtr.13454.1.S1_at | 0.141067569 |
| 675 | Mtr.18323.1.S1_at | Mtr.13502.1.S1_at | 0.212502544 |
| 676 | Mtr.18323.1.S1_at | Mtr.13507.1.S1_at | 0.160844071 |
| 677 | Mtr.18323.1.S1_at | Mtr.13537.1.S1_at | 0.156434274 |
| 678 | Mtr.18323.1.S1_at | Mtr.1354.1.S1_at | 0.120928359 |
| 679 | Mtr.18323.1.S1_at | Mtr.13562.1.S1_at | 0.226439831 |
| 680 | Mtr.18323.1.S1_at | Mtr.13568.1.S1_at | 0.171557849 |
| 681 | Mtr.18323.1.S1_at | Mtr.13576.1.S1_at | 0.243027963 |
| 682 | Mtr.18323.1.S1_at | Mtr.13580.1.S1_at | 0.154261783 |
| 683 | Mtr.18323.1.S1_at | Mtr.13615.1.S1_at | 0.157259785 |
| 684 | Mtr.18323.1.S1_at | Mtr.13617.1.S1_at | 0.237767533 |
| 685 | Mtr.34174.1.S1_at | Mtr.13625.1.S1_at | 0.112413706 |
| 686 | Mtr.18323.1.S1_at | Mtr.13637.1.S1_at | 0.198078369 |
| 687 | Mtr.18323.1.S1_at | Mtr.13647.1.S1_at | 0.227054047 |
| 688 | Mtr.18323.1.S1_at | Mtr.13658.1.S1_at | 0.113830605 |
| 689 | Mtr.18323.1.S1_at | Mtr.13730.1.S1_at | 0.146672958 |
| 690 | Mtr.18323.1.S1_at | Mtr.13793.1.S1_at | 0.152468281 |
| 691 | Mtr.18323.1.S1_at | Mtr.13794.1.S1_at | 0.218101826 |
| 692 | Mtr.18323.1.S1_at | Mtr.13797.1.S1_at | 0.192279112 |
| 693 | Mtr.18323.1.S1_at | Mtr.13811.1.S1_at | 0.131054251 |
| 694 | Mtr.18323.1.S1_at | Mtr.13821.1.S1_at | 0.217465248 |
| 695 | Mtr.18323.1.S1_at | Mtr.13822.1.S1_at | 0.139417417 |
| 696 | Mtr.18323.1.S1_at | Mtr.13828.1.S1_at | 0.149260528 |
| 697 | Mtr.18323.1.S1_at | Mtr.13856.1.S1_at | 0.212198855 |
| 698 | Mtr.18323.1.S1_at | Mtr.13859.1.S1_at | 0.184440034 |
| 699 | Mtr.18323.1.S1_at | Mtr.13948.1.S1_at | 0.197136507 |
| 700 | Mtr.18323.1.S1_at | Mtr.13957.1.S1_s_at | 0.189241118 |
| 701 | Mtr.18323.1.S1_at | Mtr.13961.1.S1_at | 0.230504199 |
| 702 | Mtr.18323.1.S1_at | Mtr.1397.1.S1_at | 0.138401588 |
| 703 | Mtr.18323.1.S1_at | Mtr.13986.1.S1_at | 0.107737219 |
| 704 | Mtr.18323.1.S1_at | Mtr.13993.1.S1_at | 0.209189841 |
| 705 | Mtr.18323.1.S1_at | Mtr.14012.1.S1_at | 0.101609919 |
| 706 | Mtr.18323.1.S1_at | Mtr.14186.1.S1_at | 0.14808607 |
| 707 | Mtr.18323.1.S1_at | Mtr.14243.1.S1_at | 0.234265645 |
| 708 | Mtr.18323.1.S1_at | Mtr.14251.1.S1_at | 0.227408603 |
| 709 | Mtr.18323.1.S1_at | Mtr.14251.1.S1_s_at | 0.231563607 |
| 710 | Mtr.18323.1.S1_at | Mtr.14298.1.S1_s_at | 0.162924345 |
| 711 | Mtr.18323.1.S1_at | Mtr.14315.1.S1_at | 0.122148876 |
| 712 | Mtr.18323.1.S1_at | Mtr.1432.1.S1_at | 0.179247919 |
| 713 | Mtr.18323.1.S1_at | Mtr.14322.1.S1_at | 0.181939086 |
| 714 | Mtr.18323.1.S1_at | Mtr.14440.1.S1_at | 0.195701368 |
| 715 | Mtr.18323.1.S1_at | Mtr.1452.1.S1_at | 0.202200211 |
| 716 | Mtr.18323.1.S1_at | Mtr.14560.1.S1_at | 0.214596893 |
| 717 | Mtr.18323.1.S1_at | Mtr.14679.1.S1_x_at | 0.119503595 |
| 718 | Mtr.18323.1.S1_at | Mtr.14785.1.S1_at | 0.115591555 |
| 719 | Mtr.18323.1.S1_at | Mtr.14809.1.S1_a_at | 0.194608662 |
| 720 | Mtr.18323.1.S1_at | Mtr.14817.1.S1_at | 0.160181706 |
| 721 | Mtr.18323.1.S1_at | Mtr.15010.1.S1_s_at | 0.186882157 |
| 722 | Mtr.18323.1.S1_at | Mtr.15058.1.S1_at | 0.213563394 |
| 723 | Mtr.18323.1.S1_at | Mtr.15257.1.S1_s_at | 0.187967804 |
| 724 | Mtr.18323.1.S1_at | Mtr.15260.1.S1_at | 0.11929965 |
| 725 | Mtr.18323.1.S1_at | Mtr.15284.1.S1_at | 0.169946776 |
| 726 | Mtr.18323.1.S1_at | Mtr.1538.1.S1_at | 0.123743124 |
| 727 | Mtr.18323.1.S1_at | Mtr.15417.1.S1_at | 0.181389235 |
| 728 | Mtr.18323.1.S1_at | Mtr.15443.1.S1_at | 0.171996296 |
| 729 | Mtr.18323.1.S1_at | Mtr.1547.1.S1_at | 0.201669119 |
| 730 | Mtr.18323.1.S1_at | Mtr.15470.1.S1_at | 0.107648118 |
| 731 | Mtr.18323.1.S1_at | Mtr.15470.1.S1_s_at | 0.117362892 |
| 732 | Mtr.18323.1.S1_at | Mtr.15471.1.S1_x_at | 0.148368537 |
| 733 | Mtr.18323.1.S1_at | Mtr.15479.1.S1_s_at | 0.168100887 |
| 734 | Mtr.18323.1.S1_at | Mtr.15540.1.S1_at | 0.162822869 |
| 735 | Mtr.18323.1.S1_at | Mtr.15549.1.S1_at | 0.148692418 |
| 736 | Mtr.18323.1.S1_at | Mtr.15550.1.S1_at | 0.122104269 |
| 737 | Mtr.34174.1.S1_at | Mtr.15656.1.S1_at | 0.102092876 |
| 738 | Mtr.18323.1.S1_at | Mtr.15716.1.S1_at | 0.100701608 |
| 739 | Mtr.18323.1.S1_at | Mtr.15741.1.S1_s_at | 0.124397178 |
| 740 | Mtr.18323.1.S1_at | Mtr.15849.1.S1_at | 0.183681717 |
| 741 | Mtr.18323.1.S1_at | Mtr.15877.1.S1_s_at | 0.244720303 |
| 742 | Mtr.18323.1.S1_at | Mtr.15880.1.S1_at | 0.188916485 |
| 743 | Mtr.18323.1.S1_at | Mtr.15920.1.S1_at | 0.125336947 |
| 744 | Mtr.18323.1.S1_at | Mtr.15961.1.S1_s_at | 0.116238465 |
| 745 | Mtr.18323.1.S1_at | Mtr.15976.1.S1_at | 0.159606555 |
| 746 | Mtr.18323.1.S1_at | Mtr.16110.1.S1_at | 0.199112499 |
| 747 | Mtr.18323.1.S1_at | Mtr.16111.1.S1_at | 0.194952962 |
| 748 | Mtr.18323.1.S1_at | Mtr.1619.1.S1_s_at | 0.120544275 |
| 749 | Mtr.18323.1.S1_at | Mtr.16232.1.S1_at | 0.19298652 |
| 750 | Mtr.34174.1.S1_at | Mtr.16278.1.S1_at | 0.124368698 |
| 751 | Mtr.18323.1.S1_at | Mtr.16417.1.S1_at | 0.156569407 |
| 752 | Mtr.34174.1.S1_at | Mtr.16505.1.S1_at | 0.149530972 |
| 753 | Mtr.18323.1.S1_at | Mtr.16547.1.S1_at | 0.129032953 |
| 754 | Mtr.18323.1.S1_at | Mtr.16726.1.S1_s_at | 0.12684132 |
| 755 | Mtr.18323.1.S1_at | Mtr.16792.1.S1_s_at | 0.218671313 |
| 756 | Mtr.18323.1.S1_at | Mtr.1682.1.S1_at | 0.106639152 |
| 757 | Mtr.18323.1.S1_at | Mtr.16882.1.S1_at | 0.105094098 |
| 758 | Mtr.18323.1.S1_at | Mtr.16959.1.S1_s_at | 0.196128647 |
| 759 | Mtr.18323.1.S1_at | Mtr.17078.1.S1_at | 0.206600472 |
| 760 | Mtr.18323.1.S1_at | Mtr.17079.1.S1_s_at | 0.209779973 |
| 761 | Mtr.18323.1.S1_at | Mtr.17091.1.S1_at | 0.114443419 |
| 762 | Mtr.18323.1.S1_at | Mtr.17107.1.S1_at | 0.135701764 |
| 763 | Mtr.18323.1.S1_at | Mtr.17167.1.S1_at | 0.160305951 |
| 764 | Mtr.18323.1.S1_at | Mtr.17280.1.S1_at | 0.204440173 |
| 765 | Mtr.18323.1.S1_at | Mtr.17362.1.S1_at | 0.110001397 |
| 766 | Mtr.18323.1.S1_at | Mtr.1740.1.S1_at | 0.233411789 |

|  | Supplementary Table 5-3. The co-expression network of *MsHAK* genes in DEGs under salt stress. | | |
| --- | --- | --- | --- |
|  | fromNode | toNode | weight |
| 1 | MsG0580029633.01.T01 | MsG0380014934.01.T01 | 0.105773961 |
| 2 | MsG0580029633.01.T01 | MsG0380016546.01.T01 | 0.323129391 |
| 3 | MsG0580029633.01.T01 | MsG0680035558.01.T01 | 0.102942528 |
| 4 | MsG0580029633.01.T01 | MsG0580029257.01.T01 | 0.208352524 |
| 5 | MsG0580029633.01.T01 | MsG0280008659.01.T01 | 0.141329429 |
| 6 | MsG0580029633.01.T01 | MsG0580024636.01.T01 | 0.115994341 |
| 7 | MsG0580029633.01.T01 | MsG0180002884.01.T01 | 0.116533014 |
| 8 | MsG0580029633.01.T01 | MsG0880046889.01.T01 | 0.189274134 |
| 9 | MsG0580029633.01.T01 | MsG0480018607.01.T01 | 0.151694094 |
| 10 | MsG0580029633.01.T01 | MsG0380015949.01.T01 | 0.105443114 |
| 11 | MsG0580029633.01.T01 | MsG0480023374.01.T01 | 0.321946382 |
| 12 | MsG0580029633.01.T01 | MsG0780040658.01.T01 | 0.142731979 |
| 13 | MsG0580029633.01.T01 | MsG0380012540.01.T01 | 0.119614738 |
| 14 | MsG0580029633.01.T01 | MsG0180004650.01.T01 | 0.122390462 |
| 15 | MsG0580029633.01.T01 | MsG0280007277.01.T01 | 0.231127458 |
| 16 | MsG0580029633.01.T01 | MsG0780039536.01.T01 | 0.113011542 |
| 17 | MsG0580029633.01.T01 | MsG0180001276.01.T01 | 0.226709006 |
| 18 | MsG0580029633.01.T01 | MsG0280007365.01.T01 | 0.303825418 |
| 19 | MsG0580029633.01.T01 | MsG0580028760.01.T01 | 0.172905441 |
| 20 | MsG0580029633.01.T01 | MsG0280007278.01.T01 | 0.155340826 |
| 21 | MsG0580029633.01.T01 | MsG0580025611.01.T01 | 0.123219405 |
| 22 | MsG0580029633.01.T01 | MsG0580024846.01.T01 | 0.19163405 |
| 23 | MsG0580029633.01.T01 | MsG0480018089.01.T04 | 0.12142631 |
| 24 | MsG0380016724.01.T01 | MsG0880043144.01.T01 | 0.116041082 |
| 25 | MsG0380016724.01.T01 | MsG0680034663.01.T01 | 0.127015605 |
| 26 | MsG0380016724.01.T01 | MsG0580024275.01.T01 | 0.224631761 |
| 27 | MsG0380016724.01.T01 | MsG0480022752.01.T02 | 0.132064087 |
| 28 | MsG0380016724.01.T01 | MsG0880045118.01.T01 | 0.112904914 |
| 29 | MsG0380016724.01.T01 | MsG0280010146.01.T01 | 0.164988779 |
| 30 | MsG0380016724.01.T01 | MsG0380015441.01.T01 | 0.144519258 |
| 31 | MsG0380016724.01.T01 | MsG0580025153.01.T01 | 0.170061911 |
| 32 | MsG0380016724.01.T01 | MsG0580028253.01.T01 | 0.102482449 |
| 33 | MsG0380016724.01.T01 | MsG0880042238.01.T01 | 0.134011697 |
| 34 | MsG0380016724.01.T01 | MsG0780039462.01.T01 | 0.151587823 |
| 35 | MsG0380016724.01.T01 | MsG0380016523.01.T01 | 0.117717138 |
| 36 | MsG0380016724.01.T01 | MsG0780040858.01.T01 | 0.109356271 |
| 37 | MsG0380016724.01.T01 | MsG0380017247.01.T01 | 0.15123638 |
| 38 | MsG0380016724.01.T01 | MsG0180002066.01.T01 | 0.145228599 |
| 39 | MsG0380016724.01.T01 | MsG0780036662.01.T01 | 0.109895685 |
| 40 | MsG0380016724.01.T01 | MsG0380016670.01.T01 | 0.116808689 |
| 41 | MsG0380016724.01.T01 | MsG0480022032.01.T01 | 0.198830345 |
| 42 | MsG0380016724.01.T01 | MsG0780040173.01.T02 | 0.145123935 |
| 43 | MsG0380016724.01.T01 | MsG0580027345.01.T02 | 0.110150348 |
| 44 | MsG0380016724.01.T01 | MsG0880046415.01.T01 | 0.129833903 |
| 45 | MsG0380016724.01.T01 | MsG0380017544.01.T03 | 0.148454934 |
| 46 | MsG0380016724.01.T01 | MsG0480022732.01.T01 | 0.100053842 |
| 47 | MsG0380016724.01.T01 | MsG0780040374.01.T01 | 0.111232331 |
| 48 | MsG0380016724.01.T01 | MsG0480021837.01.T01 | 0.123026032 |
| 49 | MsG0380016724.01.T01 | MsG0280007073.01.T01 | 0.140389721 |
| 50 | MsG0280006462.01.T01 | MsG0480020129.01.T01 | 0.224614738 |
| 51 | MsG0280006462.01.T01 | MsG0280007872.01.T01 | 0.174319897 |
| 52 | MsG0280006462.01.T01 | MsG0880046222.01.T01 | 0.134369767 |
| 53 | MsG0280006462.01.T01 | MsG0380011606.01.T01 | 0.101445453 |
| 54 | MsG0280006462.01.T01 | MsG0680034491.01.T01 | 0.121065207 |
| 55 | MsG0280006462.01.T01 | MsG0180001256.01.T01 | 0.103667296 |
| 56 | MsG0280006462.01.T01 | MsG0780040904.01.T02 | 0.130577883 |
| 57 | MsG0280006462.01.T01 | MsG0480021114.01.T01 | 0.137382012 |
| 58 | MsG0280006462.01.T01 | MsG0580025232.01.T01 | 0.128743582 |
| 59 | MsG0280006462.01.T01 | MsG0180004758.01.T02 | 0.207207095 |
| 60 | MsG0280006462.01.T01 | MsG0780041276.01.T06 | 0.155094397 |
| 61 | MsG0280006462.01.T01 | MsG0580024258.01.T01 | 0.103802907 |
| 62 | MsG0280006462.01.T01 | MsG0380016254.01.T01 | 0.140971243 |
| 63 | MsG0280006462.01.T01 | MsG0680035560.01.T01 | 0.136210086 |
| 64 | MsG0280006462.01.T01 | MsG0480021708.01.T01 | 0.151793212 |
| 65 | MsG0280006462.01.T01 | MsG0880044540.01.T01 | 0.15023905 |
| 66 | MsG0280006462.01.T01 | MsG0380016661.01.T01 | 0.189240429 |
| 67 | MsG0280006462.01.T01 | MsG0880046227.01.T01 | 0.113204188 |
| 68 | MsG0280006462.01.T01 | MsG0780037920.01.T01 | 0.170899249 |
| 69 | MsG0280006462.01.T01 | MsG0280008281.01.T01 | 0.17836376 |
| 70 | MsG0280006462.01.T01 | MsG0780039747.01.T01 | 0.237067134 |
| 71 | MsG0280006462.01.T01 | MsG0780040173.01.T03 | 0.127080759 |
| 72 | MsG0280006462.01.T01 | MsG0580024915.01.T01 | 0.132950981 |
| 73 | MsG0280006462.01.T01 | MsG0880044798.01.T01 | 0.10591844 |
| 74 | MsG0280006462.01.T01 | MsG0480020883.01.T01 | 0.137629273 |
| 75 | MsG0280006462.01.T01 | MsG0580029491.01.T01 | 0.117001842 |
| 76 | MsG0280006462.01.T01 | MsG0180006251.01.T01 | 0.127161439 |
| 77 | MsG0280006462.01.T01 | MsG0380017942.01.T01 | 0.119700907 |
| 78 | MsG0280006462.01.T01 | MsG0180005779.01.T01 | 0.123879665 |
| 79 | MsG0280006462.01.T01 | MsG0880042370.01.T01 | 0.11130371 |
| 80 | MsG0280006462.01.T01 | MsG0180002451.01.T01 | 0.167427567 |
| 81 | MsG0280006462.01.T01 | MsG0380017764.01.T01 | 0.12255609 |
| 82 | MsG0280006462.01.T01 | MsG0180001256.01.T02 | 0.103667296 |
| 83 | MsG0280006462.01.T01 | MsG0480023228.01.T01 | 0.109846135 |
| 84 | MsG0280006462.01.T01 | MsG0280007713.01.T01 | 0.170561459 |
| 85 | MsG0280006462.01.T01 | MsG0880044466.01.T01 | 0.147801177 |
| 86 | MsG0280006462.01.T01 | MsG0180004833.01.T01 | 0.145482433 |
| 87 | MsG0280006462.01.T01 | MsG0880045379.01.T01 | 0.101338626 |
| 88 | MsG0280006462.01.T01 | MsG0380017420.01.T03 | 0.107767035 |
| 89 | MsG0280006462.01.T01 | MsG0380018067.01.T03 | 0.118942436 |
| 90 | MsG0280006462.01.T01 | MsG0780036024.01.T01 | 0.146055735 |
| 91 | MsG0280006462.01.T01 | MsG0080048885.01.T01 | 0.110441539 |
| 92 | MsG0280006462.01.T01 | MsG0580024822.01.T01 | 0.182084527 |
| 93 | MsG0280006462.01.T01 | MsG0180000621.01.T01 | 0.133886264 |
| 94 | MsG0280006462.01.T01 | MsG0780039996.01.T01 | 0.109021734 |
| 95 | MsG0280006462.01.T01 | MsG0280007830.01.T01 | 0.135826127 |
| 96 | MsG0280006462.01.T01 | MsG0880046864.01.T01 | 0.171912107 |
| 97 | MsG0280006462.01.T01 | MsG0480022907.01.T01 | 0.159857508 |
| 98 | MsG0280006462.01.T01 | MsG0480022436.01.T01 | 0.12359132 |
| 99 | MsG0280006462.01.T01 | MsG0180004810.01.T01 | 0.155636113 |
| 100 | MsG0280006462.01.T01 | MsG0280008417.01.T01 | 0.124280871 |
| 101 | MsG0280006462.01.T01 | MsG0180000052.01.T01 | 0.100121953 |
| 102 | MsG0280006462.01.T01 | MsG0180001867.01.T01 | 0.127433223 |
| 103 | MsG0280006462.01.T01 | MsG0580025649.01.T01 | 0.170519918 |
| 104 | MsG0280006462.01.T01 | MsG0680035601.01.T01 | 0.119844896 |
| 105 | MsG0280006462.01.T01 | MsG0480018660.01.T01 | 0.20788797 |
| 106 | MsG0280006462.01.T01 | MsG0480018671.01.T01 | 0.104080337 |
| 107 | MsG0280006462.01.T01 | MsG0680032830.01.T01 | 0.176195047 |
| 108 | MsG0280006462.01.T01 | MsG0380015162.01.T01 | 0.100744515 |
| 109 | MsG0280006462.01.T01 | MsG0880046258.01.T01 | 0.110327426 |
| 110 | MsG0280006462.01.T01 | MsG0480023373.01.T01 | 0.109621893 |
| 111 | MsG0280006462.01.T01 | MsG0180006053.01.T01 | 0.108193539 |
| 112 | MsG0280006462.01.T01 | MsG0780038756.01.T01 | 0.146641469 |
| 113 | MsG0280006462.01.T01 | MsG0280010692.01.T01 | 0.121676005 |
| 114 | MsG0280006462.01.T01 | MsG0180005632.01.T01 | 0.147592823 |
| 115 | MsG0280006462.01.T01 | MsG0880044691.01.T01 | 0.167390832 |
| 116 | MsG0280006462.01.T01 | MsG0580030199.01.T02 | 0.112626418 |
| 117 | MsG0280006462.01.T01 | MsG0480022438.01.T01 | 0.104997624 |
| 118 | MsG0280006462.01.T01 | MsG0280011165.01.T01 | 0.147592234 |
| 119 | MsG0280006462.01.T01 | MsG0480023339.01.T01 | 0.114104364 |
| 120 | MsG0280006462.01.T01 | MsG0780040742.01.T01 | 0.163807391 |
| 121 | MsG0280006462.01.T01 | MsG0180005790.01.T01 | 0.104797715 |
| 122 | MsG0280006462.01.T01 | MsG0680033071.01.T01 | 0.182701916 |
| 123 | MsG0280006462.01.T01 | MsG0480019543.01.T01 | 0.122137475 |
| 124 | MsG0280006462.01.T01 | MsG0280008765.01.T01 | 0.143572943 |
| 125 | MsG0280006462.01.T01 | MsG0080048948.01.T01 | 0.172229607 |
| 126 | MsG0280006462.01.T01 | MsG0280008128.01.T02 | 0.179037547 |
| 127 | MsG0280006462.01.T01 | MsG0580028922.01.T01 | 0.138860434 |
| 128 | MsG0280006462.01.T01 | MsG0780040272.01.T01 | 0.149939117 |
| 129 | MsG0280006462.01.T01 | MsG0780038756.01.T02 | 0.146641469 |
| 130 | MsG0280006462.01.T01 | MsG0580025649.01.T02 | 0.170519918 |
| 131 | MsG0280006462.01.T01 | MsG0680031642.01.T01 | 0.103258443 |
| 132 | MsG0280006462.01.T01 | MsG0680031790.01.T01 | 0.117171157 |
| 133 | MsG0280006462.01.T01 | MsG0280010450.01.T03 | 0.200982954 |
| 134 | MsG0280006462.01.T01 | MsG0280008650.01.T01 | 0.114900335 |
| 135 | MsG0280006462.01.T01 | MsG0580024364.01.T02 | 0.166157724 |
| 136 | MsG0280006462.01.T01 | MsG0380014562.01.T01 | 0.10717793 |
| 137 | MsG0280006462.01.T01 | MsG0680034413.01.T02 | 0.173240464 |
| 138 | MsG0280006462.01.T01 | MsG0880045894.01.T01 | 0.100347732 |
| 139 | MsG0280006462.01.T01 | MsG0480023950.01.T01 | 0.15001082 |
| 140 | MsG0280006462.01.T01 | MsG0480019543.01.T02 | 0.122137475 |
| 141 | MsG0280006462.01.T01 | MsG0180000996.01.T01 | 0.105383719 |
| 142 | MsG0280006462.01.T01 | MsG0480024021.01.T01 | 0.122820148 |
| 143 | MsG0280006462.01.T01 | MsG0380017614.01.T01 | 0.149302741 |
| 144 | MsG0280006462.01.T01 | MsG0780037442.01.T01 | 0.110581984 |
| 145 | MsG0280006462.01.T01 | MsG0780036029.01.T01 | 0.156974497 |
| 146 | MsG0280006462.01.T01 | MsG0780036170.01.T01 | 0.10626831 |
| 147 | MsG0280006462.01.T01 | MsG0580025652.01.T01 | 0.155973228 |
| 148 | MsG0280006462.01.T01 | MsG0080048964.01.T01 | 0.100300712 |
| 149 | MsG0280006462.01.T01 | MsG0280010439.01.T01 | 0.144451654 |
| 150 | MsG0280006462.01.T01 | MsG0380015815.01.T01 | 0.120790699 |
| 151 | MsG0280006462.01.T01 | MsG0180001712.01.T01 | 0.109085577 |
| 152 | MsG0280006462.01.T01 | MsG0780040373.01.T01 | 0.113239098 |
| 153 | MsG0280006462.01.T01 | MsG0380016768.01.T01 | 0.105438345 |
| 154 | MsG0280006462.01.T01 | MsG0480023266.01.T01 | 0.132822553 |
| 155 | MsG0280006462.01.T01 | MsG0780041277.01.T01 | 0.15889657 |
| 156 | MsG0280006462.01.T01 | MsG0680034112.01.T01 | 0.124630456 |
| 157 | MsG0280006462.01.T01 | MsG0180005228.01.T01 | 0.141669527 |
| 158 | MsG0280006462.01.T01 | MsG0180000534.01.T01 | 0.100539068 |
| 159 | MsG0280006462.01.T01 | MsG0480023100.01.T01 | 0.118041374 |
| 160 | MsG0280006462.01.T01 | MsG0880047429.01.T01 | 0.174596702 |
| 161 | MsG0280006462.01.T01 | MsG0180000663.01.T01 | 0.170720735 |
| 162 | MsG0280006462.01.T01 | MsG0180005260.01.T01 | 0.172279791 |
| 163 | MsG0280006462.01.T01 | MsG0280007621.01.T01 | 0.139185732 |
| 164 | MsG0280006462.01.T01 | MsG0780041276.01.T05 | 0.155094397 |
| 165 | MsG0280006462.01.T01 | MsG0880043665.01.T01 | 0.128922212 |
| 166 | MsG0280006462.01.T01 | MsG0880042880.01.T01 | 0.128191501 |
| 167 | MsG0280006462.01.T01 | MsG0180003620.01.T01 | 0.157090039 |
| 168 | MsG0280006462.01.T01 | MsG0280010365.01.T01 | 0.179994728 |
| 169 | MsG0280006462.01.T01 | MsG0380016843.01.T01 | 0.110562959 |
| 170 | MsG0280006462.01.T01 | MsG0180000694.01.T01 | 0.136780141 |
| 171 | MsG0280006462.01.T01 | MsG0380015739.01.T03 | 0.12187668 |
| 172 | MsG0280006462.01.T01 | MsG0380012221.01.T01 | 0.121751243 |
| 173 | MsG0280006462.01.T01 | MsG0680034675.01.T01 | 0.195642169 |
| 174 | MsG0280006462.01.T01 | MsG0280008128.01.T05 | 0.179037547 |
| 175 | MsG0280006462.01.T01 | MsG0780039591.01.T01 | 0.151282199 |
| 176 | MsG0280006462.01.T01 | MsG0880045146.01.T01 | 0.102615121 |
| 177 | MsG0280006462.01.T01 | MsG0680034489.01.T01 | 0.116324195 |
| 178 | MsG0280006462.01.T01 | MsG0280008128.01.T03 | 0.179037547 |
| 179 | MsG0280006462.01.T01 | MsG0680030334.01.T01 | 0.129663595 |
| 180 | MsG0280006462.01.T01 | MsG0780039969.01.T01 | 0.109292022 |
| 181 | MsG0280006462.01.T01 | MsG0180004996.01.T02 | 0.203698446 |
| 182 | MsG0280006462.01.T01 | MsG0180000688.01.T02 | 0.113359694 |
| 183 | MsG0280006462.01.T01 | MsG0080047986.01.T01 | 0.173490554 |
| 184 | MsG0280006462.01.T01 | MsG0880042436.01.T01 | 0.168083177 |
| 185 | MsG0280006462.01.T01 | MsG0480023572.01.T01 | 0.122273947 |
| 186 | MsG0280006462.01.T01 | MsG0180003986.01.T01 | 0.15385661 |
| 187 | MsG0280006462.01.T01 | MsG0580027343.01.T01 | 0.125116147 |
| 188 | MsG0280006462.01.T01 | MsG0780041828.01.T01 | 0.101471839 |
| 189 | MsG0280006462.01.T01 | MsG0180003940.01.T02 | 0.188914021 |
| 190 | MsG0280006462.01.T01 | MsG0380016012.01.T01 | 0.140384118 |
| 191 | MsG0280006462.01.T01 | MsG0180005867.01.T01 | 0.110539252 |
| 192 | MsG0280006462.01.T01 | MsG0780041276.01.T04 | 0.155094397 |
| 193 | MsG0280006462.01.T01 | MsG0180000670.01.T01 | 0.138196158 |
| 194 | MsG0280006462.01.T01 | MsG0680035676.01.T01 | 0.149887611 |
| 195 | MsG0280006462.01.T01 | MsG0280011203.01.T01 | 0.122523638 |
| 196 | MsG0280006462.01.T01 | MsG0380018067.01.T02 | 0.118942436 |
| 197 | MsG0280006462.01.T01 | MsG0580029614.01.T01 | 0.220483903 |
| 198 | MsG0280006462.01.T01 | MsG0880045382.01.T01 | 0.102239888 |
| 199 | MsG0280006462.01.T01 | MsG0780041330.01.T01 | 0.123220303 |
| 200 | MsG0280006462.01.T01 | MsG0480023087.01.T01 | 0.13992169 |
| 201 | MsG0280006462.01.T01 | MsG0080048397.01.T01 | 0.12024834 |
| 202 | MsG0280006462.01.T01 | MsG0580028602.01.T01 | 0.115332679 |
| 203 | MsG0280006462.01.T01 | MsG0480023127.01.T01 | 0.120622136 |
| 204 | MsG0280006462.01.T01 | MsG0280011214.01.T03 | 0.119871693 |
| 205 | MsG0280006462.01.T01 | MsG0280008400.01.T01 | 0.103008036 |
| 206 | MsG0280006462.01.T01 | MsG0180003940.01.T01 | 0.188914021 |
| 207 | MsG0280006462.01.T01 | MsG0780036014.01.T01 | 0.142308753 |
| 208 | MsG0280006462.01.T01 | MsG0380016187.01.T02 | 0.182393413 |
| 209 | MsG0280006462.01.T01 | MsG0580027881.01.T01 | 0.129958763 |
| 210 | MsG0280006462.01.T01 | MsG0280010692.01.T02 | 0.121676005 |
| 211 | MsG0280006462.01.T01 | MsG0780039876.01.T01 | 0.141802598 |
| 212 | MsG0280006462.01.T01 | MsG0880046077.01.T01 | 0.125183972 |
| 213 | MsG0280006462.01.T01 | MsG0580030199.01.T01 | 0.112626418 |
| 214 | MsG0280006462.01.T01 | MsG0380016072.01.T01 | 0.131794238 |
| 215 | MsG0280006462.01.T01 | MsG0180005395.01.T03 | 0.112320364 |
| 216 | MsG0280006462.01.T01 | MsG0880045670.01.T01 | 0.142388683 |
| 217 | MsG0280006462.01.T01 | MsG0180004758.01.T01 | 0.207207095 |
| 218 | MsG0280006462.01.T01 | MsG0380017585.01.T01 | 0.16209443 |
| 219 | MsG0280006462.01.T01 | MsG0280008386.01.T01 | 0.108715716 |
| 220 | MsG0280006462.01.T01 | MsG0580028912.01.T01 | 0.160952308 |
| 221 | MsG0280006462.01.T01 | MsG0480022552.01.T01 | 0.159760282 |
| 222 | MsG0280006462.01.T01 | MsG0280009702.01.T01 | 0.180170406 |
| 223 | MsG0280006462.01.T01 | MsG0680033744.01.T01 | 0.121361069 |
| 224 | MsG0280006462.01.T01 | MsG0280010753.01.T01 | 0.146313503 |
| 225 | MsG0280006462.01.T01 | MsG0480022938.01.T01 | 0.169291411 |
| 226 | MsG0280006462.01.T01 | MsG0580028281.01.T01 | 0.175785015 |
| 227 | MsG0280006462.01.T01 | MsG0580025207.01.T01 | 0.204208065 |
| 228 | MsG0280006462.01.T01 | MsG0280007873.01.T01 | 0.107261169 |
| 229 | MsG0280006462.01.T01 | MsG0380017641.01.T01 | 0.134339497 |
| 230 | MsG0280006462.01.T01 | MsG0280008128.01.T01 | 0.179037547 |
| 231 | MsG0280006462.01.T01 | MsG0480019130.01.T01 | 0.135660255 |
| 232 | MsG0280006462.01.T01 | MsG0180003428.01.T01 | 0.120816157 |
| 233 | MsG0280006462.01.T01 | MsG0180004002.01.T01 | 0.182816029 |
| 234 | MsG0280006462.01.T01 | MsG0580028226.01.T01 | 0.111336148 |
| 235 | MsG0280006462.01.T01 | MsG0380017054.01.T01 | 0.178362992 |
| 236 | MsG0280006462.01.T01 | MsG0480018955.01.T01 | 0.125714144 |
| 237 | MsG0280006462.01.T01 | MsG0280010692.01.T03 | 0.121676005 |
| 238 | MsG0280006462.01.T01 | MsG0280010450.01.T02 | 0.200982954 |
| 239 | MsG0280006462.01.T01 | MsG0480021390.01.T01 | 0.158505537 |
| 240 | MsG0280006462.01.T01 | MsG0580028226.01.T02 | 0.111336148 |
| 241 | MsG0280006462.01.T01 | MsG0880044555.01.T01 | 0.132766678 |
| 242 | MsG0280006462.01.T01 | MsG0580024523.01.T01 | 0.156779176 |
| 243 | MsG0280006462.01.T01 | MsG0780038756.01.T03 | 0.146641469 |
| 244 | MsG0280006462.01.T01 | MsG0180000688.01.T01 | 0.113359694 |
| 245 | MsG0280006462.01.T01 | MsG0880047296.01.T01 | 0.112622999 |
| 246 | MsG0280006462.01.T01 | MsG0380017768.01.T01 | 0.20539244 |
| 247 | MsG0280006462.01.T01 | MsG0780041159.01.T01 | 0.113691678 |
| 248 | MsG0280006462.01.T01 | MsG0480023340.01.T01 | 0.120431235 |
| 249 | MsG0280006462.01.T01 | MsG0380017470.01.T01 | 0.174307926 |
| 250 | MsG0280006462.01.T01 | MsG0580028033.01.T01 | 0.190658035 |
| 251 | MsG0280006462.01.T01 | MsG0380017942.01.T02 | 0.119700907 |
| 252 | MsG0280006462.01.T01 | MsG0180003950.01.T01 | 0.153238662 |
| 253 | MsG0280006462.01.T01 | MsG0280010554.01.T01 | 0.184921853 |
| 254 | MsG0280006462.01.T01 | MsG0380012991.01.T01 | 0.121547838 |
| 255 | MsG0280006462.01.T01 | MsG0780040405.01.T01 | 0.104583008 |
| 256 | MsG0280006462.01.T01 | MsG0480023877.01.T01 | 0.15970902 |
| 257 | MsG0280006462.01.T01 | MsG0280008750.01.T01 | 0.169412168 |
| 258 | MsG0280006462.01.T01 | MsG0280006618.01.T01 | 0.101548492 |
| 259 | MsG0280006462.01.T01 | MsG0580024531.01.T01 | 0.129402511 |
| 260 | MsG0280006462.01.T01 | MsG0480021783.01.T01 | 0.145661322 |
| 261 | MsG0280006462.01.T01 | MsG0480020880.01.T01 | 0.1070222 |
| 262 | MsG0280006462.01.T01 | MsG0280010439.01.T02 | 0.144451654 |
| 263 | MsG0280006462.01.T01 | MsG0480023994.01.T01 | 0.16287624 |
| 264 | MsG0280006462.01.T01 | MsG0380017695.01.T01 | 0.127783123 |
| 265 | MsG0280006462.01.T01 | MsG0880043311.01.T01 | 0.137216427 |
| 266 | MsG0280006462.01.T01 | MsG0780039603.01.T01 | 0.102192202 |
| 267 | MsG0280006462.01.T01 | MsG0280010862.01.T01 | 0.146264015 |
| 268 | MsG0280006462.01.T01 | MsG0880045591.01.T01 | 0.144437 |
| 269 | MsG0280006462.01.T01 | MsG0380016181.01.T01 | 0.142899685 |
| 270 | MsG0280006462.01.T01 | MsG0580026924.01.T01 | 0.102780217 |
| 271 | MsG0280006462.01.T01 | MsG0880047745.01.T01 | 0.161137472 |
| 272 | MsG0280006462.01.T01 | MsG0180003620.01.T02 | 0.189157675 |
| 273 | MsG0280006462.01.T01 | MsG0680030842.01.T01 | 0.146062854 |
| 274 | MsG0280006462.01.T01 | MsG0080048825.01.T01 | 0.13018802 |
| 275 | MsG0280006462.01.T01 | MsG0280011055.01.T01 | 0.192007081 |
| 276 | MsG0280006462.01.T01 | MsG0780040173.01.T01 | 0.127080759 |
| 277 | MsG0280006462.01.T01 | MsG0580025931.01.T01 | 0.112711761 |
| 278 | MsG0280006462.01.T01 | MsG0780036362.01.T01 | 0.102372287 |
| 279 | MsG0280006462.01.T01 | MsG0780039144.01.T01 | 0.194782427 |
| 280 | MsG0280006462.01.T01 | MsG0880043303.01.T01 | 0.157220731 |
| 281 | MsG0280006462.01.T01 | MsG0780040916.01.T01 | 0.115702145 |
| 282 | MsG0280006462.01.T01 | MsG0180000594.01.T01 | 0.101244762 |
| 283 | MsG0280006462.01.T01 | MsG0880045371.01.T01 | 0.11626045 |
| 284 | MsG0280006462.01.T01 | MsG0580026158.01.T01 | 0.141096292 |
| 285 | MsG0280006462.01.T01 | MsG0280008128.01.T04 | 0.179037547 |
| 286 | MsG0280006462.01.T01 | MsG0280006605.01.T01 | 0.140314695 |
| 287 | MsG0280006462.01.T01 | MsG0780036806.01.T02 | 0.113400139 |
| 288 | MsG0280006462.01.T01 | MsG0180005395.01.T01 | 0.112320364 |
| 289 | MsG0280006462.01.T01 | MsG0180003444.01.T01 | 0.179211806 |
| 290 | MsG0280006462.01.T01 | MsG0780041625.01.T01 | 0.101276898 |
| 291 | MsG0280006462.01.T01 | MsG0580025906.01.T01 | 0.124851317 |
| 292 | MsG0280006462.01.T01 | MsG0380016070.01.T01 | 0.130571761 |
| 293 | MsG0280006462.01.T01 | MsG0480019816.01.T01 | 0.174161002 |
| 294 | MsG0280006462.01.T01 | MsG0280011054.01.T01 | 0.142819694 |
| 295 | MsG0280006462.01.T01 | MsG0280011253.01.T01 | 0.128657079 |
| 296 | MsG0280006462.01.T01 | MsG0280010450.01.T01 | 0.200982954 |
| 297 | MsG0280006462.01.T01 | MsG0480021204.01.T01 | 0.120907058 |
| 298 | MsG0280006462.01.T01 | MsG0280006596.01.T01 | 0.120172943 |
| 299 | MsG0280006462.01.T01 | MsG0680031577.01.T01 | 0.137798316 |
| 300 | MsG0280006462.01.T01 | MsG0280010489.01.T01 | 0.120383504 |
| 301 | MsG0280006462.01.T01 | MsG0780037364.01.T01 | 0.148481178 |
| 302 | MsG0280006462.01.T01 | MsG0280010146.01.T01 | 0.108020073 |
| 303 | MsG0280006462.01.T01 | MsG0380015441.01.T01 | 0.113036584 |
| 304 | MsG0280006462.01.T01 | MsG0780036806.01.T01 | 0.113400139 |
| 305 | MsG0280006462.01.T01 | MsG0680031739.01.T01 | 0.137837147 |
| 306 | MsG0280006462.01.T01 | MsG0180005395.01.T02 | 0.112320364 |
| 307 | MsG0280006462.01.T01 | MsG0180001243.01.T01 | 0.129645088 |
| 308 | MsG0280006462.01.T01 | MsG0580025768.01.T01 | 0.140133136 |
| 309 | MsG0280006462.01.T01 | MsG0880047342.01.T01 | 0.138456524 |
| 310 | MsG0280006462.01.T01 | MsG0480023028.01.T01 | 0.131773295 |
| 311 | MsG0280006462.01.T01 | MsG0780040173.01.T02 | 0.127080759 |
| 312 | MsG0280006462.01.T01 | MsG0580027345.01.T02 | 0.138240332 |
| 313 | MsG0280006462.01.T01 | MsG0880042881.01.T01 | 0.170014105 |
| 314 | MsG0280006462.01.T01 | MsG0880047509.01.T01 | 0.104497043 |
| 315 | MsG0280006462.01.T01 | MsG0480023787.01.T01 | 0.111705819 |
| 316 | MsG0280006462.01.T01 | MsG0180003767.01.T01 | 0.136672728 |
| 317 | MsG0280006462.01.T01 | MsG0280007073.01.T01 | 0.107386309 |
| 318 | MsG0280006462.01.T01 | MsG0580025151.01.T01 | 0.184513956 |
| 319 | MsG0580028415.01.T01 | MsG0280006616.01.T01 | 0.11639324 |
| 320 | MsG0580028415.01.T01 | MsG0180005982.01.T02 | 0.115281139 |
| 321 | MsG0580028415.01.T01 | MsG0680034617.01.T01 | 0.114836976 |
| 322 | MsG0580028415.01.T01 | MsG0280009971.01.T01 | 0.109924156 |
| 323 | MsG0580028415.01.T01 | MsG0580024890.01.T01 | 0.100150899 |
| 324 | MsG0580028415.01.T01 | MsG0680030660.01.T01 | 0.122160952 |
| 325 | MsG0580028415.01.T01 | MsG0180005350.01.T01 | 0.102828543 |
| 326 | MsG0580028415.01.T01 | MsG0380016111.01.T01 | 0.125755865 |
| 327 | MsG0580028415.01.T01 | MsG0880046298.01.T01 | 0.117165451 |
| 328 | MsG0580028415.01.T01 | MsG0580029028.01.T01 | 0.119410694 |
| 329 | MsG0580028415.01.T01 | MsG0580025295.01.T01 | 0.104639741 |
| 330 | MsG0580028415.01.T01 | MsG0580024631.01.T01 | 0.101753852 |
| 331 | MsG0580028415.01.T01 | MsG0180005982.01.T01 | 0.115281139 |
| 332 | MsG0580028415.01.T01 | MsG0480020884.01.T01 | 0.108980837 |
| 333 | MsG0580028415.01.T01 | MsG0480022624.01.T01 | 0.116595179 |
| 334 | MsG0580028415.01.T01 | MsG0680032495.01.T01 | 0.113914295 |
| 335 | MsG0580028415.01.T01 | MsG0580026596.01.T01 | 0.120433356 |
| 336 | MsG0580028415.01.T01 | MsG0380016473.01.T01 | 0.138009275 |
| 337 | MsG0580028415.01.T01 | MsG0280011056.01.T01 | 0.106121444 |
| 338 | MsG0580028415.01.T01 | MsG0280007691.01.T01 | 0.122872796 |
| 339 | MsG0580028415.01.T01 | MsG0480022410.01.T01 | 0.117481736 |
| 340 | MsG0580028415.01.T01 | MsG0780040123.01.T01 | 0.118476824 |
| 341 | MsG0480022802.01.T01 | MsG0880044567.01.T01 | 0.152766217 |
| 342 | MsG0480022802.01.T01 | MsG0680035614.01.T01 | 0.153028682 |
| 343 | MsG0480022802.01.T01 | MsG0180003776.01.T01 | 0.167649363 |
| 344 | MsG0480022802.01.T01 | MsG0880047375.01.T01 | 0.15906635 |
| 345 | MsG0480022802.01.T01 | MsG0580025229.01.T01 | 0.155963804 |
| 346 | MsG0480022802.01.T01 | MsG0580024213.01.T01 | 0.152311098 |
| 347 | MsG0480022802.01.T01 | MsG0780041752.01.T01 | 0.150483532 |
| 348 | MsG0480022802.01.T01 | MsG0680035696.01.T01 | 0.171207145 |
| 349 | MsG0480022802.01.T01 | MsG0180004020.01.T01 | 0.153608269 |
| 350 | MsG0480022802.01.T01 | MsG0880047416.01.T01 | 0.163920916 |
| 351 | MsG0480022802.01.T01 | MsG0380015373.01.T01 | 0.151091989 |
| 352 | MsG0480022802.01.T01 | MsG0680031966.01.T02 | 0.112585834 |
| 353 | MsG0480022802.01.T01 | MsG0480018473.01.T01 | 0.170288512 |
| 354 | MsG0280008140.01.T01 | MsG0580026085.01.T01 | 0.188294289 |
| 355 | MsG0280008140.01.T01 | MsG0180003253.01.T04 | 0.185996239 |
| 356 | MsG0280008140.01.T01 | MsG0880044638.01.T01 | 0.16715518 |
| 357 | MsG0280008140.01.T01 | MsG0880047075.01.T01 | 0.1762791 |
| 358 | MsG0280008140.01.T01 | MsG0880047571.01.T01 | 0.162765741 |
| 359 | MsG0280008140.01.T01 | MsG0180004770.01.T01 | 0.173268698 |
| 360 | MsG0580025786.01.T01 | MsG0480018513.01.T01 | 0.159794879 |
| 361 | MsG0580025786.01.T01 | MsG0780036611.01.T01 | 0.157334132 |
| 362 | MsG0580025786.01.T01 | MsG0280009552.01.T01 | 0.150073162 |
| 363 | MsG0580025786.01.T01 | MsG0480020811.01.T01 | 0.15476942 |
| 364 | MsG0580025786.01.T01 | MsG0380016261.01.T01 | 0.14453436 |
| 365 | MsG0580025786.01.T01 | MsG0780038116.01.T01 | 0.143042431 |
| 366 | MsG0580025786.01.T01 | MsG0480018513.01.T01 | 0.159794879 |
| 367 | MsG0580025786.01.T01 | MsG0780038943.01.T01 | 0.13678483 |
| 368 | MsG0580025786.01.T01 | MsG0680033159.01.T01 | 0.120167399 |
| 369 | MsG0580025786.01.T01 | MsG0280009456.01.T01 | 0.120481646 |
| 370 | MsG0580025786.01.T01 | MsG0780036611.01.T01 | 0.157334132 |
| 371 | MsG0580025786.01.T01 | MsG0780040208.01.T01 | 0.124945925 |
| 372 | MsG0580025786.01.T01 | MsG0580029667.01.T01 | 0.134314505 |
| 373 | MsG0580025786.01.T01 | MsG0180001148.01.T01 | 0.125429738 |
| 374 | MsG0580025786.01.T01 | MsG0580029670.01.T01 | 0.144694136 |
| 375 | MsG0580025786.01.T01 | MsG0080048387.01.T01 | 0.126218634 |
| 376 | MsG0580025786.01.T01 | MsG0780038135.01.T01 | 0.12248519 |
| 377 | MsG0580025786.01.T01 | MsG0180005023.01.T01 | 0.122045579 |
| 378 | MsG0580025786.01.T01 | MsG0180004947.01.T01 | 0.121325615 |
| 379 | MsG0580025786.01.T01 | MsG0580025609.01.T01 | 0.130250469 |
| 380 | MsG0580025786.01.T01 | MsG0780039897.01.T01 | 0.120000119 |
| 381 | MsG0580025786.01.T01 | MsG0180003993.01.T01 | 0.129359776 |
| 382 | MsG0580025786.01.T01 | MsG0480021690.01.T01 | 0.126185306 |
| 383 | MsG0580025786.01.T01 | MsG0880046003.01.T01 | 0.128397709 |
| 384 | MsG0580025786.01.T01 | MsG0880047210.01.T01 | 0.127390161 |
| 385 | MsG0580025786.01.T01 | MsG0280009552.01.T01 | 0.150073162 |
| 386 | MsG0580025786.01.T01 | MsG0480022751.01.T02 | 0.122505436 |
| 387 | MsG0580025786.01.T01 | MsG0580025610.01.T06 | 0.133753063 |
| 388 | MsG0580025786.01.T01 | MsG0480020811.01.T01 | 0.15476942 |
| 389 | MsG0580025786.01.T01 | MsG0780038123.01.T01 | 0.129107108 |
| 390 | MsG0580025786.01.T01 | MsG0580025610.01.T04 | 0.133753063 |
| 391 | MsG0580025786.01.T01 | MsG0280007256.01.T01 | 0.124833577 |
| 392 | MsG0580025786.01.T01 | MsG0880042036.01.T01 | 0.122993904 |
| 393 | MsG0580025786.01.T01 | MsG0580024168.01.T01 | 0.121350664 |
| 394 | MsG0580025786.01.T01 | MsG0180003734.01.T01 | 0.13451375 |
| 395 | MsG0580025786.01.T01 | MsG0380017151.01.T01 | 0.126314474 |
| 396 | MsG0580025786.01.T01 | MsG0580025610.01.T05 | 0.133753063 |
| 397 | MsG0580025786.01.T01 | MsG0180000844.01.T01 | 0.12244854 |
| 398 | MsG0580025786.01.T01 | MsG0780041437.01.T01 | 0.124817799 |
| 399 | MsG0580025786.01.T01 | MsG0780038115.01.T01 | 0.12336303 |
| 400 | MsG0580025786.01.T01 | MsG0280006737.01.T01 | 0.129314157 |
| 401 | MsG0580025786.01.T01 | MsG0280008717.01.T01 | 0.13121068 |
| 402 | MsG0580025786.01.T01 | MsG0780038940.01.T01 | 0.12337034 |
| 403 | MsG0580025786.01.T01 | MsG0880046345.01.T01 | 0.12608539 |
| 404 | MsG0580025786.01.T01 | MsG0380014967.01.T01 | 0.138202323 |
| 405 | MsG0580025786.01.T01 | MsG0180005934.01.T01 | 0.123446477 |
| 406 | MsG0580025786.01.T01 | MsG0780038132.01.T05 | 0.122741753 |
| 407 | MsG0580025786.01.T01 | MsG0280010428.01.T01 | 0.140121337 |
| 408 | MsG0380016724.01.T01 | MsG0680031642.01.T01 | 0.117860923 |
| 409 | MsG0380016724.01.T01 | MsG0180004759.01.T01 | 0.176162168 |
| 410 | MsG0380016724.01.T01 | MsG0580026580.01.T01 | 0.127131387 |
| 411 | MsG0380016724.01.T01 | MsG0180000002.01.T01 | 0.160246613 |
| 412 | MsG0380016724.01.T01 | MsG0180006245.01.T01 | 0.140136548 |
| 413 | MsG0380016724.01.T01 | MsG0580024688.01.T01 | 0.129117028 |
| 414 | MsG0380016724.01.T01 | MsG0480022053.01.T01 | 0.221416389 |
| 415 | MsG0580029633.01.T01 | MsG0580028328.01.T01 | 0.121599123 |
| 416 | MsG0580029633.01.T01 | MsG0380017899.01.T02 | 0.138803707 |
| 417 | MsG0580029633.01.T01 | MsG0480020041.01.T01 | 0.127743647 |
| 418 | MsG0380016724.01.T01 | MsG0580025270.01.T01 | 0.198816416 |
| 419 | MsG0380016724.01.T01 | MsG0680030898.01.T01 | 0.147480056 |
| 420 | MsG0580029633.01.T01 | MsG0180004371.01.T01 | 0.28504887 |
| 421 | MsG0380016724.01.T01 | MsG0280010687.01.T01 | 0.160642747 |
| 422 | MsG0380016724.01.T01 | MsG0480023950.01.T01 | 0.104891683 |
| 423 | MsG0380016724.01.T01 | MsG0880047584.01.T01 | 0.222247132 |
| 424 | MsG0380016724.01.T01 | MsG0480018237.01.T01 | 0.142695808 |
| 425 | MsG0580029633.01.T01 | MsG0180004802.01.T01 | 0.250842876 |
| 426 | MsG0380016724.01.T01 | MsG0680033149.01.T01 | 0.131445647 |
| 427 | MsG0580029633.01.T01 | MsG0480019494.01.T01 | 0.105615525 |
| 428 | MsG0380016724.01.T01 | MsG0780036774.01.T01 | 0.124605944 |
| 429 | MsG0580029633.01.T01 | MsG0280010539.01.T01 | 0.21411373 |
| 430 | MsG0380016724.01.T01 | MsG0280008573.01.T02 | 0.113615481 |
| 431 | MsG0380016724.01.T01 | MsG0480021245.01.T01 | 0.186271902 |
| 432 | MsG0380016724.01.T01 | MsG0280007530.01.T01 | 0.151389879 |
| 433 | MsG0380016724.01.T01 | MsG0180004730.01.T01 | 0.106623212 |
| 434 | MsG0380016724.01.T01 | MsG0880046899.01.T01 | 0.203594259 |
| 435 | MsG0580029633.01.T01 | MsG0180001663.01.T01 | 0.118358864 |
| 436 | MsG0380016724.01.T01 | MsG0280006560.01.T01 | 0.148333854 |
| 437 | MsG0380016724.01.T01 | MsG0280006440.01.T01 | 0.123240533 |
| 438 | MsG0580029633.01.T01 | MsG0180005757.01.T01 | 0.270451349 |
| 439 | MsG0380016724.01.T01 | MsG0780035950.01.T01 | 0.144091071 |
| 440 | MsG0380016724.01.T01 | MsG0780040324.01.T01 | 0.118804851 |
| 441 | MsG0380016724.01.T01 | MsG0380015815.01.T01 | 0.129611358 |
| 442 | MsG0580029633.01.T01 | MsG0780041426.01.T01 | 0.159344265 |
| 443 | MsG0380016724.01.T01 | MsG0880045291.01.T01 | 0.14023849 |
| 444 | MsG0380016724.01.T01 | MsG0780040373.01.T01 | 0.14518747 |
| 445 | MsG0380016724.01.T01 | MsG0280011471.01.T01 | 0.140014155 |
| 446 | MsG0580029633.01.T01 | MsG0480022788.01.T01 | 0.160552545 |
| 447 | MsG0380016724.01.T01 | MsG0580028036.01.T01 | 0.114755072 |
| 448 | MsG0380016724.01.T01 | MsG0080048904.01.T01 | 0.132444334 |
| 449 | MsG0380016724.01.T01 | MsG0180004867.01.T01 | 0.17700312 |
| 450 | MsG0380016724.01.T01 | MsG0380016148.01.T01 | 0.137426285 |
| 451 | MsG0580029633.01.T01 | MsG0380017187.01.T01 | 0.1020828 |
| 452 | MsG0380016724.01.T01 | MsG0180005616.01.T01 | 0.113986504 |
| 453 | MsG0380016724.01.T01 | MsG0780036954.01.T01 | 0.13814681 |
| 454 | MsG0580029633.01.T01 | MsG0380016568.01.T01 | 0.120698384 |
| 455 | MsG0380016724.01.T01 | MsG0380016843.01.T01 | 0.183964037 |
| 456 | MsG0380016724.01.T01 | MsG0880044418.01.T01 | 0.164862763 |
| 457 | MsG0580029633.01.T01 | MsG0380017767.01.T01 | 0.142306241 |
| 458 | MsG0380016724.01.T01 | MsG0880045006.01.T01 | 0.100400182 |
| 459 | MsG0580029633.01.T01 | MsG0880046463.01.T01 | 0.156600971 |
| 460 | MsG0380016724.01.T01 | MsG0680030316.01.T01 | 0.189068892 |
| 461 | MsG0380016724.01.T01 | MsG0880047064.01.T01 | 0.190312658 |
| 462 | MsG0580029633.01.T01 | MsG0280010645.01.T01 | 0.22405382 |
| 463 | MsG0580029633.01.T01 | MsG0880046072.01.T01 | 0.17720197 |
| 464 | MsG0580029633.01.T01 | MsG0480019322.01.T01 | 0.146619465 |
| 465 | MsG0380016724.01.T01 | MsG0580029544.01.T01 | 0.233556229 |
| 466 | MsG0380016724.01.T01 | MsG0480021769.01.T02 | 0.114081294 |
| 467 | MsG0380016724.01.T01 | MsG0480021395.01.T01 | 0.102651583 |
| 468 | MsG0580029633.01.T01 | MsG0280007396.01.T01 | 0.226407905 |
| 469 | MsG0380016724.01.T01 | MsG0880047545.01.T01 | 0.128800476 |
| 470 | MsG0380016724.01.T01 | MsG0780039969.01.T01 | 0.104312587 |
| 471 | MsG0380016724.01.T01 | MsG0780039984.01.T01 | 0.12428456 |
| 472 | MsG0380016724.01.T01 | MsG0480022481.01.T01 | 0.140598717 |
| 473 | MsG0580029633.01.T01 | MsG0380012740.01.T01 | 0.143605809 |
| 474 | MsG0380016724.01.T01 | MsG0280007013.01.T01 | 0.153786297 |
| 475 | MsG0380016724.01.T01 | MsG0780036263.01.T01 | 0.160559912 |
| 476 | MsG0380016724.01.T01 | MsG0280011304.01.T01 | 0.12994676 |
| 477 | MsG0380016724.01.T01 | MsG0280010113.01.T01 | 0.109075839 |
| 478 | MsG0380016724.01.T01 | MsG0480018583.01.T01 | 0.124429908 |
| 479 | MsG0580029633.01.T01 | MsG0880043148.01.T01 | 0.203939809 |
| 480 | MsG0380016724.01.T01 | MsG0780041361.01.T01 | 0.119620162 |
| 481 | MsG0380016724.01.T01 | MsG0380017780.01.T01 | 0.142797477 |
| 482 | MsG0380016724.01.T01 | MsG0580027343.01.T01 | 0.101900899 |
| 483 | MsG0380016724.01.T01 | MsG0380016456.01.T01 | 0.106897282 |
| 484 | MsG0380016724.01.T01 | MsG0280008381.01.T01 | 0.110568476 |
| 485 | MsG0380016724.01.T01 | MsG0380016659.01.T01 | 0.155607913 |
| 486 | MsG0380016724.01.T01 | MsG0180004524.01.T01 | 0.157845593 |
| 487 | MsG0380016724.01.T01 | MsG0080048563.01.T01 | 0.101116946 |
| 488 | MsG0380016724.01.T01 | MsG0880045191.01.T01 | 0.168743185 |
| 489 | MsG0380016724.01.T01 | MsG0780039333.01.T01 | 0.177254729 |
| 490 | MsG0380016724.01.T01 | MsG0880047197.01.T01 | 0.120487726 |
| 491 | MsG0380016724.01.T01 | MsG0880043271.01.T01 | 0.167664607 |
| 492 | MsG0380016724.01.T01 | MsG0780040917.01.T01 | 0.215407932 |
| 493 | MsG0580029633.01.T01 | MsG0380012552.01.T01 | 0.132337891 |
| 494 | MsG0380016724.01.T01 | MsG0680030459.01.T01 | 0.11175262 |
| 495 | MsG0580029633.01.T01 | MsG0380017658.01.T01 | 0.103626396 |
| 496 | MsG0380016724.01.T01 | MsG0080048072.01.T01 | 0.161853333 |
| 497 | MsG0380016724.01.T01 | MsG0580026959.01.T01 | 0.142427309 |
| 498 | MsG0380016724.01.T01 | MsG0280011203.01.T01 | 0.136306471 |
| 499 | MsG0380016724.01.T01 | MsG0380014600.01.T01 | 0.179559698 |
| 500 | MsG0380016724.01.T01 | MsG0580024746.01.T01 | 0.156084347 |
| 501 | MsG0380016724.01.T01 | MsG0480022254.01.T01 | 0.104324702 |
| 502 | MsG0380016724.01.T01 | MsG0380016229.01.T01 | 0.102024316 |
| 503 | MsG0380016724.01.T01 | MsG0280008222.01.T01 | 0.119926207 |
| 504 | MsG0380016724.01.T01 | MsG0780041330.01.T01 | 0.135787968 |
| 505 | MsG0380016724.01.T01 | MsG0680034884.01.T01 | 0.105117264 |
| 506 | MsG0380016724.01.T01 | MsG0380017483.01.T01 | 0.159413257 |
| 507 | MsG0380016724.01.T01 | MsG0780035950.01.T02 | 0.144091071 |
| 508 | MsG0380016724.01.T01 | MsG0780041103.01.T01 | 0.172017987 |
| 509 | MsG0380016724.01.T01 | MsG0780040408.01.T01 | 0.129361574 |
| 510 | MsG0380016724.01.T01 | MsG0280007117.01.T01 | 0.144664959 |
| 511 | MsG0580029633.01.T01 | MsG0480019954.01.T01 | 0.256387027 |
| 512 | MsG0380016724.01.T01 | MsG0880044439.01.T01 | 0.102965036 |
| 513 | MsG0580029633.01.T01 | MsG0780039536.01.T02 | 0.113011542 |
| 514 | MsG0580029633.01.T01 | MsG0380017115.01.T01 | 0.239695821 |
| 515 | MsG0380016724.01.T01 | MsG0580026213.01.T01 | 0.119590086 |
| 516 | MsG0380016724.01.T01 | MsG0180000751.01.T01 | 0.172492732 |
| 517 | MsG0380016724.01.T01 | MsG0480021976.01.T01 | 0.137381286 |
| 518 | MsG0380016724.01.T01 | MsG0880046135.01.T01 | 0.122967217 |
| 519 | MsG0380016724.01.T01 | MsG0180005813.01.T01 | 0.158061449 |
| 520 | MsG0380016724.01.T01 | MsG0580030055.01.T01 | 0.132007338 |
| 521 | MsG0380016724.01.T01 | MsG0880046559.01.T01 | 0.102309315 |
| 522 | MsG0380016724.01.T01 | MsG0880047308.01.T01 | 0.198860558 |
| 523 | MsG0580029633.01.T01 | MsG0080048537.01.T02 | 0.171700572 |
| 524 | MsG0380016724.01.T01 | MsG0380011895.01.T01 | 0.128291873 |
| 525 | MsG0580029633.01.T01 | MsG0880045458.01.T01 | 0.156239462 |
| 526 | MsG0380016724.01.T01 | MsG0880043457.01.T01 | 0.154837594 |
| 527 | MsG0380016724.01.T01 | MsG0180004055.01.T01 | 0.101944869 |
| 528 | MsG0580029633.01.T01 | MsG0880047741.01.T01 | 0.252957821 |
| 529 | MsG0380016724.01.T01 | MsG0380017515.01.T01 | 0.123172417 |
| 530 | MsG0580029633.01.T01 | MsG0380016968.01.T01 | 0.114617858 |
| 531 | MsG0380016724.01.T01 | MsG0880045165.01.T01 | 0.170322791 |
| 532 | MsG0380016724.01.T01 | MsG0080048703.01.T01 | 0.180202303 |
| 533 | MsG0380016724.01.T01 | MsG0380016554.01.T01 | 0.192102771 |
| 534 | MsG0380016724.01.T01 | MsG0280008597.01.T01 | 0.141775316 |
| 535 | MsG0580029633.01.T01 | MsG0580024144.01.T01 | 0.116626241 |
| 536 | MsG0380016724.01.T01 | MsG0880047145.01.T01 | 0.11522189 |
| 537 | MsG0380016724.01.T01 | MsG0580025185.01.T01 | 0.111192957 |
| 538 | MsG0580029633.01.T01 | MsG0880044312.01.T01 | 0.104327766 |
| 539 | MsG0380016724.01.T01 | MsG0380017432.01.T01 | 0.108377474 |
| 540 | MsG0380016724.01.T01 | MsG0380017552.01.T01 | 0.110221452 |
| 541 | MsG0380016724.01.T01 | MsG0880045830.01.T01 | 0.126165017 |
| 542 | MsG0380016724.01.T01 | MsG0380017544.01.T01 | 0.148454934 |
| 543 | MsG0380016724.01.T01 | MsG0880045832.01.T01 | 0.13582796 |
| 544 | MsG0580029633.01.T01 | MsG0080048150.01.T01 | 0.163297419 |
| 545 | MsG0380016724.01.T01 | MsG0580024233.01.T01 | 0.164310825 |
| 546 | MsG0580029633.01.T01 | MsG0580024798.01.T05 | 0.142617907 |
| 547 | MsG0380016724.01.T01 | MsG0480021117.01.T01 | 0.140769583 |
| 548 | MsG0380016724.01.T01 | MsG0180003428.01.T01 | 0.126545096 |
| 549 | MsG0380016724.01.T01 | MsG0380016347.01.T01 | 0.10211648 |
| 550 | MsG0380016724.01.T01 | MsG0280009839.01.T01 | 0.103761639 |
| 551 | MsG0380016724.01.T01 | MsG0680035665.01.T01 | 0.104562263 |
| 552 | MsG0580029633.01.T01 | MsG0880045257.01.T01 | 0.205659476 |
| 553 | MsG0380016724.01.T01 | MsG0480021769.01.T04 | 0.114081294 |
| 554 | MsG0580029633.01.T01 | MsG0180003958.01.T01 | 0.10378067 |
| 555 | MsG0380016724.01.T01 | MsG0480022895.01.T01 | 0.162117349 |
| 556 | MsG0380016724.01.T01 | MsG0880045042.01.T01 | 0.135610796 |
| 557 | MsG0380016724.01.T01 | MsG0480022953.01.T02 | 0.221338825 |
| 558 | MsG0380016724.01.T01 | MsG0780039462.01.T03 | 0.151587823 |
| 559 | MsG0580029633.01.T01 | MsG0280010111.01.T04 | 0.120659239 |
| 560 | MsG0580029633.01.T01 | MsG0380014774.01.T01 | 0.193633452 |
| 561 | MsG0380016724.01.T01 | MsG0880042832.01.T01 | 0.166308336 |
| 562 | MsG0380016724.01.T01 | MsG0180000461.01.T01 | 0.182063977 |
| 563 | MsG0380016724.01.T01 | MsG0180004439.01.T01 | 0.137945238 |
| 564 | MsG0380016724.01.T01 | MsG0780039901.01.T01 | 0.117231526 |
| 565 | MsG0380016724.01.T01 | MsG0180006076.01.T01 | 0.239955343 |
| 566 | MsG0380016724.01.T01 | MsG0580024312.01.T01 | 0.128603441 |
| 567 | MsG0380016724.01.T01 | MsG0580025319.01.T01 | 0.111995342 |
| 568 | MsG0380016724.01.T01 | MsG0280010244.01.T02 | 0.128801697 |
| 569 | MsG0380016724.01.T01 | MsG0780039622.01.T01 | 0.111340789 |
| 570 | MsG0380016724.01.T01 | MsG0480022860.01.T01 | 0.110806997 |
| 571 | MsG0380016724.01.T01 | MsG0880047296.01.T01 | 0.108255005 |
| 572 | MsG0380016724.01.T01 | MsG0580024872.01.T01 | 0.128473279 |
| 573 | MsG0380016724.01.T01 | MsG0880042768.01.T01 | 0.106045108 |
| 574 | MsG0380016724.01.T01 | MsG0480023340.01.T01 | 0.186242007 |
| 575 | MsG0580029633.01.T01 | MsG0380013118.01.T01 | 0.196508291 |
| 576 | MsG0380016724.01.T01 | MsG0780041360.01.T01 | 0.117422403 |
| 577 | MsG0380016724.01.T01 | MsG0480021959.01.T01 | 0.114578136 |
| 578 | MsG0380016724.01.T01 | MsG0580026047.01.T01 | 0.101042682 |
| 579 | MsG0380016724.01.T01 | MsG0880044391.01.T01 | 0.228066922 |
| 580 | MsG0380016724.01.T01 | MsG0380017254.01.T01 | 0.110353341 |
| 581 | MsG0380016724.01.T01 | MsG0380015213.01.T01 | 0.116417477 |
| 582 | MsG0380016724.01.T01 | MsG0280011259.01.T01 | 0.157529786 |
| 583 | MsG0380016724.01.T01 | MsG0780038860.01.T01 | 0.121706383 |
| 584 | MsG0380016724.01.T01 | MsG0780040405.01.T01 | 0.140144283 |
| 585 | MsG0380016724.01.T01 | MsG0480022752.01.T01 | 0.132064087 |
| 586 | MsG0380016724.01.T01 | MsG0280006595.01.T01 | 0.164620186 |
| 587 | MsG0380016724.01.T01 | MsG0880047497.01.T01 | 0.133909608 |
| 588 | MsG0380016724.01.T01 | MsG0580030097.01.T01 | 0.122027241 |
| 589 | MsG0380016724.01.T01 | MsG0880045567.01.T01 | 0.110222636 |
| 590 | MsG0380016724.01.T01 | MsG0480020880.01.T01 | 0.150379315 |
| 591 | MsG0380016724.01.T01 | MsG0480022899.01.T01 | 0.132357834 |
| 592 | MsG0380016724.01.T01 | MsG0180004403.01.T01 | 0.148686816 |
| 593 | MsG0380016724.01.T01 | MsG0380015547.01.T01 | 0.173302602 |
| 594 | MsG0380016724.01.T01 | MsG0180001090.01.T01 | 0.204094216 |
| 595 | MsG0380016724.01.T01 | MsG0580028035.01.T01 | 0.117844067 |
| 596 | MsG0380016724.01.T01 | MsG0480021244.01.T01 | 0.167831671 |
| 597 | MsG0380016724.01.T01 | MsG0280006700.01.T01 | 0.110262192 |
| 598 | MsG0380016724.01.T01 | MsG0480022258.01.T01 | 0.17988438 |
| 599 | MsG0380016724.01.T01 | MsG0780036244.01.T01 | 0.129529518 |
| 600 | MsG0380016724.01.T01 | MsG0880045591.01.T01 | 0.107466261 |
| 601 | MsG0380016724.01.T01 | MsG0580028433.01.T01 | 0.132556005 |
| 602 | MsG0380016724.01.T01 | MsG0880045285.01.T01 | 0.126045404 |
| 603 | MsG0380016724.01.T01 | MsG0780040173.01.T01 | 0.145123935 |
| 604 | MsG0380016724.01.T01 | MsG0280009789.01.T01 | 0.135102724 |
| 605 | MsG0380016724.01.T01 | MsG0780036390.01.T01 | 0.109802195 |
| 606 | MsG0380016724.01.T01 | MsG0480020912.01.T01 | 0.151208846 |
| 607 | MsG0380016724.01.T01 | MsG0780037701.01.T01 | 0.221768097 |
| 608 | MsG0380016724.01.T01 | MsG0380017380.01.T01 | 0.151344063 |
| 609 | MsG0380016724.01.T01 | MsG0580029544.01.T02 | 0.233556229 |
| 610 | MsG0380016724.01.T01 | MsG0780036362.01.T01 | 0.168950211 |
| 611 | MsG0380016724.01.T01 | MsG0680031713.01.T01 | 0.155877862 |
| 612 | MsG0380016724.01.T01 | MsG0180000594.01.T01 | 0.136133607 |
| 613 | MsG0380016724.01.T01 | MsG0580028021.01.T01 | 0.188160839 |
| 614 | MsG0380016724.01.T01 | MsG0080047991.01.T01 | 0.147948423 |
| 615 | MsG0380016724.01.T01 | MsG0080049121.01.T01 | 0.141326825 |
| 616 | MsG0380016724.01.T01 | MsG0580026644.01.T01 | 0.177707253 |
| 617 | MsG0380016724.01.T01 | MsG0680030315.01.T01 | 0.186331384 |
| 618 | MsG0380016724.01.T01 | MsG0080048886.01.T01 | 0.129930803 |
| 619 | MsG0380016724.01.T01 | MsG0480022258.01.T02 | 0.17988438 |
| 620 | MsG0380016724.01.T01 | MsG0580026269.01.T01 | 0.107320978 |
| 621 | MsG0380016724.01.T01 | MsG0180000148.01.T01 | 0.103126754 |
| 622 | MsG0380016724.01.T01 | MsG0680034391.01.T01 | 0.122771282 |
| 623 | MsG0380016724.01.T01 | MsG0780039595.01.T01 | 0.180322035 |
| 624 | MsG0380016724.01.T01 | MsG0280008886.01.T01 | 0.122766831 |
| 625 | MsG0380016724.01.T01 | MsG0480021204.01.T01 | 0.120217627 |
| 626 | MsG0380016724.01.T01 | MsG0480021124.01.T01 | 0.119790309 |
| 627 | MsG0380016724.01.T01 | MsG0280007023.01.T01 | 0.109240951 |
| 628 | MsG0380016724.01.T01 | MsG0480022469.01.T01 | 0.192623705 |
| 629 | MsG0380016724.01.T01 | MsG0280011036.01.T01 | 0.111532335 |
| 630 | MsG0380016724.01.T01 | MsG0280007486.01.T01 | 0.17703515 |
| 631 | MsG0380016724.01.T01 | MsG0880045819.01.T01 | 0.137544028 |
| 632 | MsG0280006462.01.T01 | MsG0180004349.01.T01 | 0.112199362 |
| 633 | MsG0380016724.01.T01 | MsG0180004349.01.T01 | 0.175794396 |
| 634 | MsG0280006462.01.T01 | MsG0380016187.01.T01 | 0.182393413 |
| 635 | MsG0580029633.01.T01 | MsG0680032436.01.T01 | 0.258703654 |
| 636 | MsG0280006462.01.T01 | MsG0180005030.01.T01 | 0.133306358 |
| 637 | MsG0280006462.01.T01 | MsG0880046862.01.T01 | 0.125075743 |
| 638 | MsG0380016724.01.T01 | MsG0680033911.01.T01 | 0.14937959 |
| 639 | MsG0280006462.01.T01 | MsG0480022035.01.T01 | 0.166352764 |
| 640 | MsG0280006462.01.T01 | MsG0180000274.01.T01 | 0.13289619 |
| 641 | MsG0380016724.01.T01 | MsG0580026960.01.T01 | 0.117859504 |
| 642 | MsG0280006462.01.T01 | MsG0380015902.01.T01 | 0.102848596 |
| 643 | MsG0580029633.01.T01 | MsG0580024998.01.T01 | 0.17875011 |
| 644 | MsG0280006462.01.T01 | MsG0180001816.01.T01 | 0.145942393 |
| 645 | MsG0580029633.01.T01 | MsG0580027777.01.T01 | 0.102577471 |
| 646 | MsG0380016724.01.T01 | MsG0880044381.01.T01 | 0.132003657 |
| 647 | MsG0580029633.01.T01 | MsG0380016384.01.T01 | 0.209450932 |
| 648 | MsG0380016724.01.T01 | MsG0880045173.01.T01 | 0.137879595 |
| 649 | MsG0380016724.01.T01 | MsG0880043525.01.T01 | 0.15161698 |
| 650 | MsG0380016724.01.T01 | MsG0580029697.01.T01 | 0.182676589 |
| 651 | MsG0280006462.01.T01 | MsG0580027345.01.T01 | 0.138240332 |
| 652 | MsG0380016724.01.T01 | MsG0580027345.01.T01 | 0.110150348 |
| 653 | MsG0380016724.01.T01 | MsG0880044763.01.T01 | 0.10394473 |
| 654 | MsG0580029633.01.T01 | MsG0480018089.01.T02 | 0.12142631 |
| 655 | MsG0380016724.01.T01 | MsG0180005479.01.T01 | 0.179290195 |
| 656 | MsG0580029633.01.T01 | MsG0580028977.01.T01 | 0.105649764 |
| 657 | MsG0580029633.01.T01 | MsG0180004949.01.T01 | 0.100872255 |
| 658 | MsG0380016724.01.T01 | MsG0780041105.01.T01 | 0.154983248 |
| 659 | MsG0380016724.01.T01 | MsG0880043473.01.T01 | 0.129559999 |
| 660 | MsG0380016724.01.T01 | MsG0080048816.01.T01 | 0.135418008 |
| 661 | MsG0380016724.01.T01 | MsG0280010159.01.T01 | 0.130962236 |
| 662 | MsG0380016724.01.T01 | MsG0680031853.01.T01 | 0.140459453 |
| 663 | MsG0580029633.01.T01 | MsG0880045101.01.T01 | 0.197482353 |
| 664 | MsG0380016724.01.T01 | MsG0480023374.01.T02 | 0.1447523 |
| 665 | MsG0380016724.01.T01 | MsG0480022610.01.T01 | 0.141797193 |
| 666 | MsG0380016724.01.T01 | MsG0580029566.01.T01 | 0.142156677 |
| 667 | MsG0380016724.01.T01 | MsG0880044413.01.T01 | 0.242881152 |
| 668 | MsG0580029633.01.T01 | MsG0780039834.01.T01 | 0.10038859 |
| 669 | MsG0380016724.01.T01 | MsG0480018184.01.T01 | 0.114859726 |
| 670 | MsG0380016724.01.T01 | MsG0480018673.01.T01 | 0.107405804 |
| 671 | MsG0380016724.01.T01 | MsG0180001256.01.T01 | 0.176453078 |
| 672 | MsG0380016724.01.T01 | MsG0680034766.01.T02 | 0.10006749 |
| 673 | MsG0380016724.01.T01 | MsG0780040904.01.T02 | 0.128586344 |
| 674 | MsG0380016724.01.T01 | MsG0480021114.01.T01 | 0.112335247 |
| 675 | MsG0380016724.01.T01 | MsG0480021769.01.T01 | 0.114081294 |
| 676 | MsG0380016724.01.T01 | MsG0780039250.01.T01 | 0.184118698 |
| 677 | MsG0380016724.01.T01 | MsG0580030022.01.T01 | 0.143817452 |
| 678 | MsG0380016724.01.T01 | MsG0480021940.01.T01 | 0.206740031 |
| 679 | MsG0380016724.01.T01 | MsG0180005624.01.T01 | 0.152852722 |
| 680 | MsG0380016724.01.T01 | MsG0380017707.01.T01 | 0.134000687 |
| 681 | MsG0580029633.01.T01 | MsG0580028899.01.T01 | 0.202249138 |
| 682 | MsG0380016724.01.T01 | MsG0880046227.01.T01 | 0.106592649 |
| 683 | MsG0580029633.01.T01 | MsG0480018089.01.T03 | 0.12142631 |
| 684 | MsG0580029633.01.T01 | MsG0180000340.01.T01 | 0.164749208 |
| 685 | MsG0380016724.01.T01 | MsG0380017544.01.T02 | 0.148454934 |
| 686 | MsG0380016724.01.T01 | MsG0780040173.01.T03 | 0.145123935 |
| 687 | MsG0380016724.01.T01 | MsG0180000462.01.T01 | 0.165777911 |
| 688 | MsG0380016724.01.T01 | MsG0780039901.01.T02 | 0.117231526 |
| 689 | MsG0380016724.01.T01 | MsG0080047996.01.T01 | 0.137344053 |
| 690 | MsG0380016724.01.T01 | MsG0580024915.01.T01 | 0.112218686 |
| 691 | MsG0380016724.01.T01 | MsG0480020827.01.T01 | 0.193377672 |
| 692 | MsG0580029633.01.T01 | MsG0280009023.01.T01 | 0.238350217 |
| 693 | MsG0380016724.01.T01 | MsG0480020883.01.T01 | 0.120832802 |
| 694 | MsG0580029633.01.T01 | MsG0080047976.01.T01 | 0.206640098 |
| 695 | MsG0380016724.01.T01 | MsG0780036866.01.T01 | 0.171839305 |
| 696 | MsG0380016724.01.T01 | MsG0180005779.01.T01 | 0.119208019 |
| 697 | MsG0380016724.01.T01 | MsG0380017764.01.T01 | 0.13597369 |
| 698 | MsG0380016724.01.T01 | MsG0280009238.01.T01 | 0.106240466 |
| 699 | MsG0580029633.01.T01 | MsG0480018089.01.T01 | 0.12142631 |
| 700 | MsG0380016724.01.T01 | MsG0180001256.01.T02 | 0.176453078 |
| 701 | MsG0380016724.01.T01 | MsG0280010688.01.T01 | 0.191381808 |
| 702 | MsG0380016724.01.T01 | MsG0280007430.01.T01 | 0.168367846 |
| 703 | MsG0580029633.01.T01 | MsG0180003603.01.T01 | 0.306289802 |
| 704 | MsG0580029633.01.T01 | MsG0080049017.01.T01 | 0.105615525 |
| 705 | MsG0380016724.01.T01 | MsG0780039388.01.T01 | 0.106516395 |
| 706 | MsG0580029633.01.T01 | MsG0880045790.01.T01 | 0.227265638 |
| 707 | MsG0380016724.01.T01 | MsG0180000597.01.T01 | 0.132930742 |
| 708 | MsG0380016724.01.T01 | MsG0380017247.01.T02 | 0.15123638 |
| 709 | MsG0380016724.01.T01 | MsG0780039841.01.T01 | 0.136731101 |
| 710 | MsG0380016724.01.T01 | MsG0880042765.01.T01 | 0.104454256 |
| 711 | MsG0380016724.01.T01 | MsG0580025632.01.T01 | 0.180272322 |
| 712 | MsG0380016724.01.T01 | MsG0280006417.01.T01 | 0.18067606 |
| 713 | MsG0380016724.01.T01 | MsG0380017516.01.T01 | 0.111703543 |
| 714 | MsG0380016724.01.T01 | MsG0080048885.01.T01 | 0.139112689 |
| 715 | MsG0380016724.01.T01 | MsG0580025152.01.T01 | 0.203457118 |
| 716 | MsG0380016724.01.T01 | MsG0680032308.01.T01 | 0.110876485 |
| 717 | MsG0380016724.01.T01 | MsG0780040904.01.T01 | 0.158084618 |
| 718 | MsG0380016724.01.T01 | MsG0180005041.01.T02 | 0.185346287 |
| 719 | MsG0380016724.01.T01 | MsG0880046884.01.T01 | 0.148872293 |
| 720 | MsG0380016724.01.T01 | MsG0780039996.01.T01 | 0.160207997 |
| 721 | MsG0380016724.01.T01 | MsG0480018561.01.T01 | 0.12158792 |
| 722 | MsG0380016724.01.T01 | MsG0280008417.01.T01 | 0.116218911 |
| 723 | MsG0380016724.01.T01 | MsG0180000764.01.T01 | 0.116270737 |
| 724 | MsG0380016724.01.T01 | MsG0180004223.01.T01 | 0.131697803 |
| 725 | MsG0380016724.01.T01 | MsG0180005368.01.T01 | 0.128094614 |
| 726 | MsG0380016724.01.T01 | MsG0380015670.01.T01 | 0.181549767 |
| 727 | MsG0580029633.01.T01 | MsG0880046788.01.T01 | 0.104993512 |
| 728 | MsG0380016724.01.T01 | MsG0280006700.01.T02 | 0.110262192 |
| 729 | MsG0380016724.01.T01 | MsG0480018227.01.T01 | 0.148088408 |
| 730 | MsG0580029633.01.T01 | MsG0280009326.01.T01 | 0.228522746 |
| 731 | MsG0380016724.01.T01 | MsG0380016103.01.T01 | 0.10663274 |
| 732 | MsG0580029633.01.T01 | MsG0080048186.01.T01 | 0.118410275 |
| 733 | MsG0580029633.01.T01 | MsG0180005756.01.T01 | 0.265813392 |
| 734 | MsG0380016724.01.T01 | MsG0680032830.01.T01 | 0.103384641 |
| 735 | MsG0380016724.01.T01 | MsG0580024537.01.T01 | 0.12013167 |
| 736 | MsG0380016724.01.T01 | MsG0180001940.01.T01 | 0.101031823 |
| 737 | MsG0580029633.01.T01 | MsG0880042059.01.T01 | 0.14888895 |
| 738 | MsG0380016724.01.T01 | MsG0880046258.01.T01 | 0.141007698 |
| 739 | MsG0580029633.01.T01 | MsG0880044312.01.T02 | 0.104327766 |
| 740 | MsG0380016724.01.T01 | MsG0280011140.01.T01 | 0.117452149 |
| 741 | MsG0580029633.01.T01 | MsG0280006647.01.T01 | 0.102944671 |
| 742 | MsG0380016724.01.T01 | MsG0480023641.01.T01 | 0.136664901 |
| 743 | MsG0380016724.01.T01 | MsG0380016436.01.T01 | 0.121036016 |
| 744 | MsG0380016724.01.T01 | MsG0480022383.01.T01 | 0.131528034 |
| 745 | MsG0380016724.01.T01 | MsG0180005041.01.T01 | 0.185346287 |
| 746 | MsG0380016724.01.T01 | MsG0880046042.01.T01 | 0.111120367 |
| 747 | MsG0380016724.01.T01 | MsG0180003599.01.T01 | 0.14800953 |
| 748 | MsG0380016724.01.T01 | MsG0380016878.01.T01 | 0.151567744 |
| 749 | MsG0380016724.01.T01 | MsG0480022953.01.T01 | 0.221338825 |
| 750 | MsG0380016724.01.T01 | MsG0480023604.01.T01 | 0.192388602 |
| 751 | MsG0380016724.01.T01 | MsG0580025035.01.T01 | 0.11226845 |
| 752 | MsG0380016724.01.T01 | MsG0180006063.01.T01 | 0.115804315 |
| 753 | MsG0380016724.01.T01 | MsG0680030731.01.T01 | 0.110678932 |
| 754 | MsG0380016724.01.T01 | MsG0480023339.01.T01 | 0.129139345 |
| 755 | MsG0380016724.01.T01 | MsG0480020898.01.T01 | 0.106581215 |
| 756 | MsG0380016724.01.T01 | MsG0880044397.01.T01 | 0.143484585 |
| 757 | MsG0380016724.01.T01 | MsG0180000291.01.T01 | 0.103150488 |
| 758 | MsG0380016724.01.T01 | MsG0280007916.01.T01 | 0.10137462 |
| 759 | MsG0380016724.01.T01 | MsG0580026643.01.T01 | 0.198179359 |
| 760 | MsG0580029633.01.T01 | MsG0080048336.01.T01 | 0.285727144 |
| 761 | MsG0580029633.01.T01 | MsG0180002008.01.T01 | 0.138759241 |
| 762 | MsG0380016724.01.T01 | MsG0280010244.01.T01 | 0.128801697 |
| 763 | MsG0380016724.01.T01 | MsG0880047555.01.T01 | 0.107633572 |
| 764 | MsG0580029633.01.T01 | MsG0180001276.01.T02 | 0.226709006 |
| 765 | MsG0380016724.01.T01 | MsG0480020473.01.T01 | 0.171945473 |
| 766 | MsG0380016724.01.T01 | MsG0880045006.01.T02 | 0.100400182 |
| 767 | MsG0380016724.01.T01 | MsG0480018982.01.T01 | 0.169236323 |
| 768 | MsG0580029633.01.T01 | MsG0380014734.01.T01 | 0.253317235 |
| 769 | MsG0580029633.01.T01 | MsG0280008414.01.T01 | 0.118575617 |
| 770 | MsG0580029633.01.T01 | MsG0680031310.01.T01 | 0.117680674 |
| 771 | MsG0380016724.01.T01 | MsG0780039462.01.T02 | 0.151587823 |
| 772 | MsG0380016724.01.T01 | MsG0280007916.01.T01 | 0.10137462 |
| 773 | MsG0380016724.01.T01 | MsG0580026643.01.T01 | 0.198179359 |
| 774 | MsG0580029633.01.T01 | MsG0080048336.01.T01 | 0.285727144 |
| 775 | MsG0580029633.01.T01 | MsG0180002008.01.T01 | 0.138759241 |
| 776 | MsG0380016724.01.T01 | MsG0280010244.01.T01 | 0.128801697 |
| 777 | MsG0380016724.01.T01 | MsG0880047555.01.T01 | 0.107633572 |
| 778 | MsG0580029633.01.T01 | MsG0180001276.01.T02 | 0.226709006 |
| 779 | MsG0380016724.01.T01 | MsG0480020473.01.T01 | 0.171945473 |
| 780 | MsG0380016724.01.T01 | MsG0880045006.01.T02 | 0.100400182 |
| 781 | MsG0380016724.01.T01 | MsG0480018982.01.T01 | 0.169236323 |
| 782 | MsG0580029633.01.T01 | MsG0380014734.01.T01 | 0.253317235 |
| 783 | MsG0580029633.01.T01 | MsG0280008414.01.T01 | 0.118575617 |
| 784 | MsG0580029633.01.T01 | MsG0680031310.01.T01 | 0.117680674 |
| 785 | MsG0380016724.01.T01 | MsG0780039462.01.T02 | 0.151587823 |
| 786 | MsG0580028415.01.T01 | MsG0180001201.01.T01 | 0.103369131 |
| 787 | MsG0580028415.01.T01 | MsG0280010361.01.T01 | 0.117332784 |
| 788 | MsG0580028415.01.T01 | MsG0280007830.01.T02 | 0.148279023 |
| 789 | MsG0580028415.01.T01 | MsG0180005635.01.T01 | 0.106447144 |
| 790 | MsG0580028415.01.T01 | MsG0880047389.01.T01 | 0.149723531 |
| 791 | MsG0580028415.01.T01 | MsG0880047390.01.T01 | 0.109666516 |
| 792 | MsG0480022802.01.T01 | MsG0780041534.01.T01 | 0.153007814 |
| 793 | MsG0480022802.01.T01 | MsG0580025674.01.T01 | 0.15407891 |
| 794 | MsG0480022802.01.T01 | MsG0480018449.01.T01 | 0.154160287 |
| 795 | MsG0480022802.01.T01 | MsG0680031966.01.T01 | 0.112585834 |
| 796 | MsG0480022802.01.T01 | MsG0680035325.01.T01 | 0.168792786 |
| 797 | MsG0480022802.01.T01 | MsG0580029277.01.T01 | 0.1524898 |
| 798 | MsG0480022802.01.T01 | MsG0780040510.01.T01 | 0.170828563 |
| 799 | MsG0480022802.01.T01 | MsG0780041534.01.T02 | 0.153007814 |
| 800 | MsG0480022802.01.T01 | MsG0580029277.01.T02 | 0.1524898 |
| 801 | MsG0480022802.01.T01 | MsG0280008344.01.T01 | 0.155100998 |
| 802 | MsG0480022802.01.T01 | MsG0680031240.01.T01 | 0.159436166 |
| 803 | MsG0480022802.01.T01 | MsG0180004904.01.T01 | 0.158819589 |
| 804 | MsG0480022802.01.T01 | MsG0780041056.01.T01 | 0.154338811 |
| 805 | MsG0480022802.01.T01 | MsG0880043870.01.T01 | 0.158753906 |
| 806 | MsG0480022802.01.T01 | MsG0580024929.01.T01 | 0.150608448 |
| 807 | MsG0480022802.01.T01 | MsG0680031240.01.T02 | 0.159436166 |
| 808 | MsG0480022802.01.T01 | MsG0580024823.01.T01 | 0.159408788 |
| 809 | MsG0480022802.01.T01 | MsG0280010945.01.T01 | 0.168799443 |
| 810 | MsG0280008140.01.T01 | MsG0180003253.01.T03 | 0.185996239 |
| 811 | MsG0280008140.01.T01 | MsG0380016053.01.T01 | 0.15246283 |
| 812 | MsG0280008140.01.T01 | MsG0380012161.01.T01 | 0.157223462 |
| 813 | MsG0280008140.01.T01 | MsG0580025112.01.T01 | 0.15023481 |
| 814 | MsG0280008140.01.T01 | MsG0180002247.01.T01 | 0.153346831 |
| 815 | MsG0280008140.01.T01 | MsG0780041761.01.T01 | 0.162567679 |
| 816 | MsG0280008140.01.T01 | MsG0380015145.01.T01 | 0.153398087 |
| 817 | MsG0280008140.01.T01 | MsG0280009750.01.T01 | 0.173716783 |
| 818 | MsG0280008140.01.T01 | MsG0880047531.01.T01 | 0.17524161 |
| 819 | MsG0280008140.01.T01 | MsG0880045958.01.T01 | 0.15750838 |
| 820 | MsG0280008140.01.T01 | MsG0580024871.01.T01 | 0.151827469 |
| 821 | MsG0280008140.01.T01 | MsG0880045943.01.T01 | 0.15750838 |
| 822 | MsG0280008140.01.T01 | MsG0580028560.01.T01 | 0.184289511 |
| 823 | MsG0280008140.01.T01 | MsG0780039783.01.T01 | 0.151777731 |
| 824 | MsG0280008140.01.T01 | MsG0380016053.01.T02 | 0.15246283 |
| 825 | MsG0280008140.01.T01 | MsG0280010861.01.T01 | 0.150727426 |
| 826 | MsG0280008140.01.T01 | MsG0180003253.01.T02 | 0.185996239 |
| 827 | MsG0280008140.01.T01 | MsG0880047214.01.T01 | 0.151022096 |
| 828 | MsG0280008140.01.T01 | MsG0180003253.01.T01 | 0.185996239 |
| 829 | MsG0280008140.01.T01 | MsG0580029256.01.T01 | 0.160378806 |
| 830 | MsG0280008140.01.T01 | MsG0880045029.01.T01 | 0.183407138 |
| 831 | MsG0280008140.01.T01 | MsG0880047215.01.T01 | 0.166914111 |
| 832 | MsG0280008140.01.T01 | MsG0580025221.01.T01 | 0.169089759 |
| 833 | MsG0280008140.01.T01 | MsG0880044769.01.T01 | 0.151494607 |

|  | Supplementary Table 5-4. The co-expression network of *MsHAK* genes in DEGs under drought stress. | | |
| --- | --- | --- | --- |
|  | fromNode | toNode | weight |
| 1 | MsG0280006462.01.T01 | MsG0280006554.01.T01 | 0.135050051 |
| 2 | MsG0280006462.01.T01 | MsG0280006596.01.T01 | 0.131515098 |
| 3 | MsG0280006462.01.T01 | MsG0280006616.01.T01 | 0.106322062 |
| 4 | MsG0280006462.01.T01 | MsG0280006617.01.T01 | 0.123600753 |
| 5 | MsG0280006462.01.T01 | MsG0280006618.01.T01 | 0.114568984 |
| 6 | MsG0280006462.01.T01 | MsG0280006636.01.T01 | 0.104739636 |
| 7 | MsG0280006462.01.T01 | MsG0280006649.01.T01 | 0.146443083 |
| 8 | MsG0280006462.01.T01 | MsG0280006708.01.T01 | 0.201139054 |
| 9 | MsG0280006462.01.T01 | MsG0280006941.01.T01 | 0.157940926 |
| 10 | MsG0280006462.01.T01 | MsG0280006947.01.T01 | 0.126812307 |
| 11 | MsG0280006462.01.T01 | MsG0280006947.01.T02 | 0.126812307 |
| 12 | MsG0280006462.01.T01 | MsG0280006947.01.T03 | 0.126812307 |
| 13 | MsG0280006462.01.T01 | MsG0280007053.01.T01 | 0.121955649 |
| 14 | MsG0280006462.01.T01 | MsG0280007072.01.T01 | 0.144005025 |
| 15 | MsG0280006462.01.T01 | MsG0280007073.01.T01 | 0.129079852 |
| 16 | MsG0280006462.01.T01 | MsG0280007083.01.T01 | 0.151150291 |
| 17 | MsG0280006462.01.T01 | MsG0280007111.01.T01 | 0.218510807 |
| 18 | MsG0280006462.01.T01 | MsG0280007120.01.T01 | 0.12111898 |
| 19 | MsG0280006462.01.T01 | MsG0280007240.01.T01 | 0.149424395 |
| 20 | MsG0280006462.01.T01 | MsG0280007391.01.T01 | 0.106572857 |
| 21 | MsG0280006462.01.T01 | MsG0280007618.01.T01 | 0.122017294 |
| 22 | MsG0280006462.01.T01 | MsG0280007713.01.T01 | 0.189595297 |
| 23 | MsG0280006462.01.T01 | MsG0280007777.01.T01 | 0.119926612 |
| 24 | MsG0280006462.01.T01 | MsG0280007830.01.T01 | 0.222278765 |
| 25 | MsG0280006462.01.T01 | MsG0280007830.01.T02 | 0.209845784 |
| 26 | MsG0280006462.01.T01 | MsG0280007872.01.T01 | 0.14178417 |
| 27 | MsG0280006462.01.T01 | MsG0280007937.01.T01 | 0.185406867 |
| 28 | MsG0280006462.01.T01 | MsG0280007940.01.T01 | 0.17743425 |
| 29 | MsG0280006462.01.T01 | MsG0280008128.01.T01 | 0.117671743 |
| 30 | MsG0280006462.01.T01 | MsG0280008128.01.T02 | 0.117671743 |
| 31 | MsG0280006462.01.T01 | MsG0280008128.01.T03 | 0.117671743 |
| 32 | MsG0280006462.01.T01 | MsG0280008128.01.T04 | 0.117671743 |
| 33 | MsG0280006462.01.T01 | MsG0280008128.01.T05 | 0.117671743 |
| 34 | MsG0280006462.01.T01 | MsG0280008281.01.T01 | 0.122639242 |
| 35 | MsG0280006462.01.T01 | MsG0280008386.01.T01 | 0.145744584 |
| 36 | MsG0280006462.01.T01 | MsG0280008417.01.T01 | 0.130908385 |
| 37 | MsG0280006462.01.T01 | MsG0280008519.01.T01 | 0.140963031 |
| 38 | MsG0280006462.01.T01 | MsG0280008650.01.T01 | 0.134461457 |
| 39 | MsG0280006462.01.T01 | MsG0280008742.01.T01 | 0.110221377 |
| 40 | MsG0280006462.01.T01 | MsG0280008742.01.T02 | 0.110221377 |
| 41 | MsG0280006462.01.T01 | MsG0280008742.01.T03 | 0.110221377 |
| 42 | MsG0280006462.01.T01 | MsG0280008750.01.T01 | 0.206047172 |
| 43 | MsG0280006462.01.T01 | MsG0280008851.01.T01 | 0.156591848 |
| 44 | MsG0280006462.01.T01 | MsG0280008887.01.T01 | 0.173110153 |
| 45 | MsG0280006462.01.T01 | MsG0280009342.01.T01 | 0.177011926 |
| 46 | MsG0280006462.01.T01 | MsG0280009352.01.T01 | 0.153954493 |
| 47 | MsG0280006462.01.T01 | MsG0280009352.01.T02 | 0.120654056 |
| 48 | MsG0280006462.01.T01 | MsG0280009448.01.T01 | 0.210438679 |
| 49 | MsG0280006462.01.T01 | MsG0280009461.01.T01 | 0.12464908 |
| 50 | MsG0280006462.01.T01 | MsG0280009510.01.T01 | 0.101585877 |
| 51 | MsG0280006462.01.T01 | MsG0280009550.01.T01 | 0.124024809 |
| 52 | MsG0280006462.01.T01 | MsG0280009678.01.T01 | 0.187061553 |
| 53 | MsG0280006462.01.T01 | MsG0280009702.01.T01 | 0.152103331 |
| 54 | MsG0280006462.01.T01 | MsG0280009719.01.T01 | 0.108509468 |
| 55 | MsG0280006462.01.T01 | MsG0280009750.01.T01 | 0.17285928 |
| 56 | MsG0280006462.01.T01 | MsG0280009750.01.T02 | 0.103875727 |
| 57 | MsG0280006462.01.T01 | MsG0280009777.01.T01 | 0.148829331 |
| 58 | MsG0280006462.01.T01 | MsG0280009789.01.T01 | 0.100864791 |
| 59 | MsG0280006462.01.T01 | MsG0280009812.01.T01 | 0.196134646 |
| 60 | MsG0280006462.01.T01 | MsG0280009858.01.T01 | 0.137713866 |
| 61 | MsG0280006462.01.T01 | MsG0280009859.01.T01 | 0.120752613 |
| 62 | MsG0280006462.01.T01 | MsG0280009971.01.T01 | 0.185463927 |
| 63 | MsG0280006462.01.T01 | MsG0280009979.01.T01 | 0.149586198 |
| 64 | MsG0280006462.01.T01 | MsG0280010149.01.T01 | 0.205901789 |
| 65 | MsG0280006462.01.T01 | MsG0280010245.01.T01 | 0.139357746 |
| 66 | MsG0280006462.01.T01 | MsG0280010283.01.T01 | 0.154100001 |
| 67 | MsG0280006462.01.T01 | MsG0280010361.01.T01 | 0.142248137 |
| 68 | MsG0280006462.01.T01 | MsG0280010365.01.T01 | 0.158793273 |
| 69 | MsG0280006462.01.T01 | MsG0280010375.01.T01 | 0.14166643 |
| 70 | MsG0280006462.01.T01 | MsG0280010408.01.T01 | 0.152649752 |
| 71 | MsG0280006462.01.T01 | MsG0280010439.01.T01 | 0.225207298 |
| 72 | MsG0280006462.01.T01 | MsG0280010439.01.T02 | 0.225207298 |
| 73 | MsG0280006462.01.T01 | MsG0280010450.01.T01 | 0.214118252 |
| 74 | MsG0280006462.01.T01 | MsG0280010450.01.T02 | 0.214118252 |
| 75 | MsG0280006462.01.T01 | MsG0280010450.01.T03 | 0.214118252 |
| 76 | MsG0280006462.01.T01 | MsG0280010531.01.T01 | 0.152250719 |
| 77 | MsG0280006462.01.T01 | MsG0280010540.01.T01 | 0.161759431 |
| 78 | MsG0280006462.01.T01 | MsG0280010554.01.T01 | 0.181246946 |
| 79 | MsG0280006462.01.T01 | MsG0280010609.01.T01 | 0.126673085 |
| 80 | MsG0280006462.01.T01 | MsG0280010627.01.T01 | 0.218855261 |
| 81 | MsG0280006462.01.T01 | MsG0280010677.01.T01 | 0.151423639 |
| 82 | MsG0280006462.01.T01 | MsG0280010692.01.T01 | 0.173346285 |
| 83 | MsG0280006462.01.T01 | MsG0280010692.01.T02 | 0.173346285 |
| 84 | MsG0280006462.01.T01 | MsG0280010692.01.T03 | 0.173346285 |
| 85 | MsG0280006462.01.T01 | MsG0280010724.01.T01 | 0.165159706 |
| 86 | MsG0280006462.01.T01 | MsG0280010799.01.T01 | 0.175907147 |
| 87 | MsG0280006462.01.T01 | MsG0280010799.01.T02 | 0.175907147 |
| 88 | MsG0280006462.01.T01 | MsG0280010799.01.T03 | 0.143692451 |
| 89 | MsG0280006462.01.T01 | MsG0280010799.01.T04 | 0.143692451 |
| 90 | MsG0280006462.01.T01 | MsG0280010862.01.T01 | 0.202224158 |
| 91 | MsG0280006462.01.T01 | MsG0280010862.01.T03 | 0.172288614 |
| 92 | MsG0280006462.01.T01 | MsG0280010929.01.T01 | 0.1364401 |
| 93 | MsG0280006462.01.T01 | MsG0280010941.01.T01 | 0.124260929 |
| 94 | MsG0280006462.01.T01 | MsG0280010976.01.T01 | 0.215133517 |
| 95 | MsG0280006462.01.T01 | MsG0280011019.01.T01 | 0.163834955 |
| 96 | MsG0280006462.01.T01 | MsG0280011027.01.T01 | 0.147888987 |
| 97 | MsG0280006462.01.T01 | MsG0280011032.01.T01 | 0.134876427 |
| 98 | MsG0280006462.01.T01 | MsG0280011045.01.T01 | 0.19535317 |
| 99 | MsG0280006462.01.T01 | MsG0280011054.01.T01 | 0.14496981 |
| 100 | MsG0280006462.01.T01 | MsG0280011055.01.T01 | 0.189829452 |
| 101 | MsG0280006462.01.T01 | MsG0280011056.01.T01 | 0.133740273 |
| 102 | MsG0280006462.01.T01 | MsG0280011082.01.T01 | 0.10621125 |
| 103 | MsG0280006462.01.T01 | MsG0280011117.01.T01 | 0.200200024 |
| 104 | MsG0280006462.01.T01 | MsG0280011138.01.T01 | 0.111943153 |
| 105 | MsG0280006462.01.T01 | MsG0280011165.01.T01 | 0.147750703 |
| 106 | MsG0280006462.01.T01 | MsG0280011199.01.T01 | 0.165506716 |
| 107 | MsG0280006462.01.T01 | MsG0280011203.01.T01 | 0.124993565 |
| 108 | MsG0280006462.01.T01 | MsG0280011259.01.T01 | 0.103142151 |
| 109 | MsG0280006462.01.T01 | MsG0280011266.01.T01 | 0.116823651 |
| 110 | MsG0280006462.01.T01 | MsG0280011338.01.T01 | 0.133966757 |
| 111 | MsG0280006462.01.T01 | MsG0280011344.01.T01 | 0.179706753 |
| 112 | MsG0280006462.01.T01 | MsG0280011440.01.T01 | 0.200937478 |
| 113 | MsG0280006462.01.T01 | MsG0280011463.01.T01 | 0.147472071 |
| 114 | MsG0280006462.01.T01 | MsG0380011578.01.T01 | 0.17667565 |
| 115 | MsG0280006462.01.T01 | MsG0380011656.01.T01 | 0.127127494 |
| 116 | MsG0280006462.01.T01 | MsG0380011660.01.T01 | 0.105575305 |
| 117 | MsG0280006462.01.T01 | MsG0380011706.01.T01 | 0.14314426 |
| 118 | MsG0280006462.01.T01 | MsG0380011706.01.T02 | 0.14314426 |
| 119 | MsG0280006462.01.T01 | MsG0380011840.01.T01 | 0.215587123 |
| 120 | MsG0280006462.01.T01 | MsG0380011840.01.T02 | 0.215587123 |
| 121 | MsG0280006462.01.T01 | MsG0380011870.01.T01 | 0.110676603 |
| 122 | MsG0280006462.01.T01 | MsG0380011918.01.T01 | 0.141790729 |
| 123 | MsG0280006462.01.T01 | MsG0380012063.01.T01 | 0.124372714 |
| 124 | MsG0280006462.01.T01 | MsG0380012089.01.T01 | 0.13733359 |
| 125 | MsG0280006462.01.T01 | MsG0380012100.01.T01 | 0.163878743 |
| 126 | MsG0280006462.01.T01 | MsG0380012124.01.T01 | 0.219024256 |
| 127 | MsG0280006462.01.T01 | MsG0380012131.01.T01 | 0.139846799 |
| 128 | MsG0280006462.01.T01 | MsG0380012221.01.T01 | 0.243866921 |
| 129 | MsG0280006462.01.T01 | MsG0380012237.01.T01 | 0.178577301 |
| 130 | MsG0280006462.01.T01 | MsG0380012330.01.T01 | 0.204482036 |
| 131 | MsG0280006462.01.T01 | MsG0380012624.01.T01 | 0.200302004 |
| 132 | MsG0280006462.01.T01 | MsG0380012899.01.T01 | 0.120213868 |
| 133 | MsG0280006462.01.T01 | MsG0380012991.01.T01 | 0.100901017 |
| 134 | MsG0280006462.01.T01 | MsG0380013071.01.T01 | 0.186284826 |
| 135 | MsG0280006462.01.T01 | MsG0380013474.01.T01 | 0.200828652 |
| 136 | MsG0280006462.01.T01 | MsG0380013505.01.T01 | 0.16467391 |
| 137 | MsG0280006462.01.T01 | MsG0380013644.01.T01 | 0.155454063 |
| 138 | MsG0280006462.01.T01 | MsG0380013682.01.T01 | 0.152124534 |
| 139 | MsG0280006462.01.T01 | MsG0380014160.01.T01 | 0.15480338 |
| 140 | MsG0280006462.01.T01 | MsG0380014182.01.T01 | 0.165178321 |
| 141 | MsG0280006462.01.T01 | MsG0380014270.01.T01 | 0.140094353 |
| 142 | MsG0280006462.01.T01 | MsG0380014600.01.T01 | 0.129430978 |
| 143 | MsG0280006462.01.T01 | MsG0380014615.01.T01 | 0.118755811 |
| 144 | MsG0280006462.01.T01 | MsG0380014632.01.T03 | 0.14896125 |
| 145 | MsG0280006462.01.T01 | MsG0380015007.01.T01 | 0.195473854 |
| 146 | MsG0280006462.01.T01 | MsG0380015070.01.T01 | 0.162391348 |
| 147 | MsG0280006462.01.T01 | MsG0380015134.01.T01 | 0.118804465 |
| 148 | MsG0280006462.01.T01 | MsG0380015196.01.T01 | 0.128105375 |
| 149 | MsG0280006462.01.T01 | MsG0380015307.01.T01 | 0.138586393 |
| 150 | MsG0280006462.01.T01 | MsG0380015330.01.T01 | 0.180739847 |
| 151 | MsG0280006462.01.T01 | MsG0380015339.01.T01 | 0.123300658 |
| 152 | MsG0280006462.01.T01 | MsG0380015404.01.T01 | 0.140663147 |
| 153 | MsG0280006462.01.T01 | MsG0380015419.01.T02 | 0.179692522 |
| 154 | MsG0280006462.01.T01 | MsG0380015531.01.T01 | 0.11544902 |
| 155 | MsG0280006462.01.T01 | MsG0380015553.01.T01 | 0.164926944 |
| 156 | MsG0280006462.01.T01 | MsG0380015598.01.T01 | 0.173298852 |
| 157 | MsG0280006462.01.T01 | MsG0380015614.01.T01 | 0.192536044 |
| 158 | MsG0280006462.01.T01 | MsG0380015739.01.T03 | 0.138807851 |
| 159 | MsG0280006462.01.T01 | MsG0380015787.01.T01 | 0.190433912 |
| 160 | MsG0280006462.01.T01 | MsG0380015799.01.T01 | 0.15612721 |
| 161 | MsG0280006462.01.T01 | MsG0380015968.01.T01 | 0.225495907 |
| 162 | MsG0280006462.01.T01 | MsG0380015991.01.T01 | 0.185550882 |
| 163 | MsG0280006462.01.T01 | MsG0380016025.01.T01 | 0.154698202 |
| 164 | MsG0280006462.01.T01 | MsG0380016063.01.T01 | 0.119197887 |
| 165 | MsG0280006462.01.T01 | MsG0380016072.01.T01 | 0.190607241 |
| 166 | MsG0280006462.01.T01 | MsG0380016104.01.T01 | 0.219023779 |
| 167 | MsG0280006462.01.T01 | MsG0380016166.01.T01 | 0.129310818 |
| 168 | MsG0280006462.01.T01 | MsG0380016167.01.T01 | 0.135619202 |
| 169 | MsG0280006462.01.T01 | MsG0380016181.01.T01 | 0.109059852 |
| 170 | MsG0280006462.01.T01 | MsG0380016187.01.T01 | 0.14937776 |
| 171 | MsG0280006462.01.T01 | MsG0380016187.01.T02 | 0.14937776 |
| 172 | MsG0280006462.01.T01 | MsG0380016226.01.T01 | 0.102988704 |
| 173 | MsG0280006462.01.T01 | MsG0380016275.01.T01 | 0.108283565 |
| 174 | MsG0280006462.01.T01 | MsG0380016290.01.T01 | 0.130657912 |
| 175 | MsG0280006462.01.T01 | MsG0380016397.01.T01 | 0.222182165 |
| 176 | MsG0280006462.01.T01 | MsG0380016516.01.T01 | 0.105129636 |
| 177 | MsG0280006462.01.T01 | MsG0380016531.01.T01 | 0.125059461 |
| 178 | MsG0280006462.01.T01 | MsG0380016552.01.T01 | 0.110832348 |
| 179 | MsG0280006462.01.T01 | MsG0380016593.01.T01 | 0.148445364 |
| 180 | MsG0280006462.01.T01 | MsG0380016596.01.T02 | 0.132356984 |
| 181 | MsG0280006462.01.T01 | MsG0380016627.01.T01 | 0.110599514 |
| 182 | MsG0280006462.01.T01 | MsG0380016661.01.T01 | 0.229328941 |
| 183 | MsG0280006462.01.T01 | MsG0380016768.01.T01 | 0.179046704 |
| 184 | MsG0280006462.01.T01 | MsG0380016768.01.T02 | 0.174830277 |
| 185 | MsG0280006462.01.T01 | MsG0380016877.01.T01 | 0.1227831 |
| 186 | MsG0280006462.01.T01 | MsG0380016877.01.T02 | 0.1227831 |
| 187 | MsG0280006462.01.T01 | MsG0380016878.01.T01 | 0.124472995 |
| 188 | MsG0280006462.01.T01 | MsG0380016931.01.T01 | 0.174935892 |
| 189 | MsG0280006462.01.T01 | MsG0380016948.01.T01 | 0.120036929 |
| 190 | MsG0280006462.01.T01 | MsG0380017092.01.T02 | 0.186000215 |
| 191 | MsG0280006462.01.T01 | MsG0380017169.01.T01 | 0.16754356 |
| 192 | MsG0280006462.01.T01 | MsG0380017254.01.T01 | 0.116350861 |
| 193 | MsG0280006462.01.T01 | MsG0380017281.01.T01 | 0.119283862 |
| 194 | MsG0280006462.01.T01 | MsG0380017285.01.T01 | 0.17894301 |
| 195 | MsG0280006462.01.T01 | MsG0380017354.01.T01 | 0.103839759 |
| 196 | MsG0280006462.01.T01 | MsG0380017357.01.T04 | 0.103517571 |
| 197 | MsG0280006462.01.T01 | MsG0380017470.01.T01 | 0.130950839 |
| 198 | MsG0280006462.01.T01 | MsG0380017471.01.T01 | 0.173594348 |
| 199 | MsG0280006462.01.T01 | MsG0380017495.01.T02 | 0.177691779 |
| 200 | MsG0280006462.01.T01 | MsG0380017507.01.T01 | 0.205625928 |
| 201 | MsG0280006462.01.T01 | MsG0380017517.01.T01 | 0.140811705 |
| 202 | MsG0280006462.01.T01 | MsG0380017528.01.T01 | 0.188204193 |
| 203 | MsG0280006462.01.T01 | MsG0380017536.01.T01 | 0.112091257 |
| 204 | MsG0280006462.01.T01 | MsG0380017614.01.T01 | 0.206341902 |
| 205 | MsG0280006462.01.T01 | MsG0380017641.01.T01 | 0.156080399 |
| 206 | MsG0280006462.01.T01 | MsG0380017695.01.T01 | 0.130950862 |
| 207 | MsG0280006462.01.T01 | MsG0380017764.01.T01 | 0.126962006 |
| 208 | MsG0280006462.01.T01 | MsG0380017768.01.T01 | 0.177589569 |
| 209 | MsG0280006462.01.T01 | MsG0380017789.01.T01 | 0.111982777 |
| 210 | MsG0280006462.01.T01 | MsG0380017791.01.T01 | 0.12194526 |
| 211 | MsG0280006462.01.T01 | MsG0380017827.01.T01 | 0.153743087 |
| 212 | MsG0280006462.01.T01 | MsG0380017833.01.T01 | 0.100086878 |
| 213 | MsG0280006462.01.T01 | MsG0380017893.01.T01 | 0.144738766 |
| 214 | MsG0280006462.01.T01 | MsG0380017942.01.T01 | 0.209520784 |
| 215 | MsG0280006462.01.T01 | MsG0380017942.01.T02 | 0.209520784 |
| 216 | MsG0280006462.01.T01 | MsG0380018012.01.T01 | 0.11426991 |
| 217 | MsG0280006462.01.T01 | MsG0480018086.01.T02 | 0.2161336 |
| 218 | MsG0280006462.01.T01 | MsG0480018086.01.T03 | 0.194227534 |
| 219 | MsG0280006462.01.T01 | MsG0480018116.01.T01 | 0.103383037 |
| 220 | MsG0280006462.01.T01 | MsG0480018145.01.T01 | 0.14874864 |
| 221 | MsG0280006462.01.T01 | MsG0480018318.01.T01 | 0.15262484 |
| 222 | MsG0280006462.01.T01 | MsG0480018350.01.T01 | 0.155553304 |
| 223 | MsG0280006462.01.T01 | MsG0480018417.01.T01 | 0.144314741 |
| 224 | MsG0280006462.01.T01 | MsG0480018432.01.T01 | 0.148115103 |
| 225 | MsG0280006462.01.T01 | MsG0480018441.01.T01 | 0.162725808 |
| 226 | MsG0280006462.01.T01 | MsG0480018445.01.T01 | 0.128566104 |
| 227 | MsG0280006462.01.T01 | MsG0480018446.01.T01 | 0.12443778 |
| 228 | MsG0280006462.01.T01 | MsG0480018659.01.T01 | 0.164022587 |
| 229 | MsG0280006462.01.T01 | MsG0480018660.01.T01 | 0.201455291 |
| 230 | MsG0280006462.01.T01 | MsG0480018974.01.T01 | 0.141464223 |
| 231 | MsG0280006462.01.T01 | MsG0480019217.01.T01 | 0.12319675 |
| 232 | MsG0280006462.01.T01 | MsG0480019295.01.T01 | 0.105130246 |
| 233 | MsG0280006462.01.T01 | MsG0480019619.01.T01 | 0.167824473 |
| 234 | MsG0280006462.01.T01 | MsG0480019816.01.T01 | 0.21399864 |
| 235 | MsG0280006462.01.T01 | MsG0480020126.01.T01 | 0.181803387 |
| 236 | MsG0280006462.01.T01 | MsG0480020129.01.T01 | 0.17895497 |
| 237 | MsG0280006462.01.T01 | MsG0480020373.01.T01 | 0.148808135 |
| 238 | MsG0280006462.01.T01 | MsG0480020444.01.T01 | 0.176802146 |
| 239 | MsG0280006462.01.T01 | MsG0480020445.01.T01 | 0.177558347 |
| 240 | MsG0280006462.01.T01 | MsG0480020584.01.T01 | 0.166080786 |
| 241 | MsG0280006462.01.T01 | MsG0480020704.01.T01 | 0.105506118 |
| 242 | MsG0280006462.01.T01 | MsG0480020828.01.T01 | 0.113353129 |
| 243 | MsG0280006462.01.T01 | MsG0480020828.01.T06 | 0.133317138 |
| 244 | MsG0280006462.01.T01 | MsG0480020836.01.T02 | 0.113047269 |
| 245 | MsG0280006462.01.T01 | MsG0480020859.01.T01 | 0.128617262 |
| 246 | MsG0280006462.01.T01 | MsG0480020859.01.T02 | 0.128617262 |
| 247 | MsG0280006462.01.T01 | MsG0480020880.01.T01 | 0.122074963 |
| 248 | MsG0280006462.01.T01 | MsG0480020883.01.T08 | 0.168529902 |
| 249 | MsG0280006462.01.T01 | MsG0480020884.01.T01 | 0.187849121 |
| 250 | MsG0280006462.01.T01 | MsG0480021132.01.T01 | 0.134508624 |
| 251 | MsG0280006462.01.T01 | MsG0480021134.01.T01 | 0.109318938 |
| 252 | MsG0280006462.01.T01 | MsG0480021159.01.T01 | 0.13281024 |
| 253 | MsG0280006462.01.T01 | MsG0480021197.01.T01 | 0.1020177 |
| 254 | MsG0280006462.01.T01 | MsG0480021390.01.T01 | 0.140344023 |
| 255 | MsG0280006462.01.T01 | MsG0480021450.01.T01 | 0.111688441 |
| 256 | MsG0280006462.01.T01 | MsG0480021479.01.T01 | 0.226151961 |
| 257 | MsG0280006462.01.T01 | MsG0480021479.01.T02 | 0.226151961 |
| 258 | MsG0280006462.01.T01 | MsG0480021658.01.T01 | 0.146044903 |
| 259 | MsG0280006462.01.T01 | MsG0480021708.01.T01 | 0.24149229 |
| 260 | MsG0280006462.01.T01 | MsG0480021772.01.T01 | 0.168142223 |
| 261 | MsG0280006462.01.T01 | MsG0480021784.01.T01 | 0.14448753 |
| 262 | MsG0280006462.01.T01 | MsG0480021828.01.T01 | 0.103499617 |
| 263 | MsG0280006462.01.T01 | MsG0480021876.01.T01 | 0.152378753 |
| 264 | MsG0280006462.01.T01 | MsG0480022145.01.T01 | 0.19022785 |
| 265 | MsG0280006462.01.T01 | MsG0480022233.01.T01 | 0.102639908 |
| 266 | MsG0280006462.01.T01 | MsG0480022258.01.T01 | 0.141938138 |
| 267 | MsG0280006462.01.T01 | MsG0480022258.01.T02 | 0.141938138 |
| 268 | MsG0280006462.01.T01 | MsG0480022259.01.T07 | 0.206233578 |
| 269 | MsG0280006462.01.T01 | MsG0480022378.01.T01 | 0.124978052 |
| 270 | MsG0280006462.01.T01 | MsG0480022381.01.T01 | 0.135117331 |
| 271 | MsG0280006462.01.T01 | MsG0480022396.01.T01 | 0.127610917 |
| 272 | MsG0280006462.01.T01 | MsG0480022410.01.T01 | 0.213015343 |
| 273 | MsG0280006462.01.T01 | MsG0480022436.01.T01 | 0.125462483 |
| 274 | MsG0280006462.01.T01 | MsG0480022437.01.T01 | 0.173549478 |
| 275 | MsG0280006462.01.T01 | MsG0480022474.01.T01 | 0.132300018 |
| 276 | MsG0280006462.01.T01 | MsG0480022540.01.T01 | 0.144654212 |
| 277 | MsG0280006462.01.T01 | MsG0480022552.01.T01 | 0.126728893 |
| 278 | MsG0280006462.01.T01 | MsG0480022553.01.T01 | 0.16714588 |
| 279 | MsG0280006462.01.T01 | MsG0480022611.01.T01 | 0.111526261 |
| 280 | MsG0280006462.01.T01 | MsG0480022669.01.T01 | 0.128310248 |
| 281 | MsG0280006462.01.T01 | MsG0480022669.01.T02 | 0.128310248 |
| 282 | MsG0280006462.01.T01 | MsG0480022800.01.T01 | 0.223669557 |
| 283 | MsG0280006462.01.T01 | MsG0480022801.01.T01 | 0.223414775 |
| 284 | MsG0280006462.01.T01 | MsG0480022815.01.T01 | 0.134171694 |
| 285 | MsG0280006462.01.T01 | MsG0480022816.01.T01 | 0.145289367 |
| 286 | MsG0280006462.01.T01 | MsG0480022818.01.T01 | 0.121568042 |
| 287 | MsG0280006462.01.T01 | MsG0480022853.01.T01 | 0.12304569 |
| 288 | MsG0280006462.01.T01 | MsG0480022910.01.T01 | 0.135950009 |
| 289 | MsG0280006462.01.T01 | MsG0480022938.01.T01 | 0.194117262 |
| 290 | MsG0280006462.01.T01 | MsG0480022938.01.T02 | 0.190747095 |
| 291 | MsG0280006462.01.T01 | MsG0480022939.01.T01 | 0.224037082 |
| 292 | MsG0280006462.01.T01 | MsG0480023024.01.T01 | 0.147142487 |
| 293 | MsG0280006462.01.T01 | MsG0480023050.01.T01 | 0.116758587 |
| 294 | MsG0280006462.01.T01 | MsG0480023124.01.T01 | 0.184009181 |
| 295 | MsG0280006462.01.T01 | MsG0480023127.01.T01 | 0.171000443 |
| 296 | MsG0280006462.01.T01 | MsG0480023128.01.T01 | 0.106079857 |
| 297 | MsG0280006462.01.T01 | MsG0480023156.01.T01 | 0.112225547 |
| 298 | MsG0280006462.01.T01 | MsG0480023228.01.T01 | 0.135396816 |
| 299 | MsG0280006462.01.T01 | MsG0480023244.01.T01 | 0.151211412 |
| 300 | MsG0280006462.01.T01 | MsG0480023266.01.T01 | 0.116179506 |
| 301 | MsG0280006462.01.T01 | MsG0480023339.01.T01 | 0.144673698 |
| 302 | MsG0280006462.01.T01 | MsG0480023340.01.T01 | 0.105401763 |
| 303 | MsG0280006462.01.T01 | MsG0480023393.01.T01 | 0.20902069 |
| 304 | MsG0280006462.01.T01 | MsG0480023414.01.T01 | 0.127968976 |
| 305 | MsG0280006462.01.T01 | MsG0480023414.01.T02 | 0.127968976 |
| 306 | MsG0280006462.01.T01 | MsG0480023516.01.T01 | 0.144795919 |
| 307 | MsG0280006462.01.T01 | MsG0480023557.01.T01 | 0.119452018 |
| 308 | MsG0280006462.01.T01 | MsG0480023572.01.T01 | 0.177439328 |
| 309 | MsG0280006462.01.T01 | MsG0480023608.01.T01 | 0.140067342 |
| 310 | MsG0280006462.01.T01 | MsG0480023609.01.T01 | 0.188220934 |
| 311 | MsG0280006462.01.T01 | MsG0480023702.01.T01 | 0.110490337 |
| 312 | MsG0280006462.01.T01 | MsG0480023702.01.T02 | 0.17960112 |
| 313 | MsG0280006462.01.T01 | MsG0480023781.01.T01 | 0.164626814 |
| 314 | MsG0280006462.01.T01 | MsG0480023798.01.T01 | 0.108148751 |
| 315 | MsG0280006462.01.T01 | MsG0480023877.01.T01 | 0.146285986 |
| 316 | MsG0280006462.01.T01 | MsG0480023896.01.T01 | 0.186903642 |
| 317 | MsG0280006462.01.T01 | MsG0480023918.01.T01 | 0.124125262 |
| 318 | MsG0280006462.01.T01 | MsG0480023945.01.T01 | 0.15476462 |
| 319 | MsG0280006462.01.T01 | MsG0480023950.01.T01 | 0.137454136 |
| 320 | MsG0280006462.01.T01 | MsG0480023972.01.T01 | 0.232348519 |
| 321 | MsG0280006462.01.T01 | MsG0480023994.01.T01 | 0.116677571 |
| 322 | MsG0280006462.01.T01 | MsG0480024002.01.T01 | 0.126343235 |
| 323 | MsG0280006462.01.T01 | MsG0480024021.01.T01 | 0.116232992 |
| 324 | MsG0280006462.01.T01 | MsG0580024050.01.T01 | 0.163746282 |
| 325 | MsG0280006462.01.T01 | MsG0580024077.01.T01 | 0.110101786 |
| 326 | MsG0280006462.01.T01 | MsG0580024077.01.T02 | 0.110101786 |
| 327 | MsG0280006462.01.T01 | MsG0580024077.01.T03 | 0.122196842 |
| 328 | MsG0280006462.01.T01 | MsG0580024083.01.T01 | 0.133638567 |
| 329 | MsG0280006462.01.T01 | MsG0580024119.01.T01 | 0.120076595 |
| 330 | MsG0280006462.01.T01 | MsG0580024343.01.T01 | 0.127798378 |
| 331 | MsG0280006462.01.T01 | MsG0580024392.01.T01 | 0.193649441 |
| 332 | MsG0280006462.01.T01 | MsG0580024409.01.T01 | 0.111019154 |
| 333 | MsG0280006462.01.T01 | MsG0580024411.01.T01 | 0.131332199 |
| 334 | MsG0280006462.01.T01 | MsG0580024472.01.T01 | 0.181463983 |
| 335 | MsG0280006462.01.T01 | MsG0580024519.01.T01 | 0.185451636 |
| 336 | MsG0280006462.01.T01 | MsG0580024524.01.T01 | 0.189376872 |
| 337 | MsG0280006462.01.T01 | MsG0580024531.01.T01 | 0.208703625 |
| 338 | MsG0280006462.01.T01 | MsG0580024532.01.T01 | 0.191652848 |
| 339 | MsG0280006462.01.T01 | MsG0580024584.01.T01 | 0.110321823 |
| 340 | MsG0280006462.01.T01 | MsG0580024606.01.T01 | 0.165954189 |
| 341 | MsG0280006462.01.T01 | MsG0580024607.01.T01 | 0.182974323 |
| 342 | MsG0280006462.01.T01 | MsG0580024631.01.T01 | 0.19489195 |
| 343 | MsG0280006462.01.T01 | MsG0580024688.01.T01 | 0.101696578 |
| 344 | MsG0280006462.01.T01 | MsG0580024770.01.T01 | 0.110435345 |
| 345 | MsG0280006462.01.T01 | MsG0580024778.01.T01 | 0.111827847 |
| 346 | MsG0280006462.01.T01 | MsG0580024825.01.T01 | 0.12571212 |
| 347 | MsG0280006462.01.T01 | MsG0580024872.01.T01 | 0.117826525 |
| 348 | MsG0280006462.01.T01 | MsG0580024890.01.T01 | 0.185077477 |
| 349 | MsG0280006462.01.T01 | MsG0580025027.01.T01 | 0.11870272 |
| 350 | MsG0280006462.01.T01 | MsG0580025069.01.T01 | 0.136106661 |
| 351 | MsG0280006462.01.T01 | MsG0580025151.01.T01 | 0.117336203 |
| 352 | MsG0280006462.01.T01 | MsG0580025207.01.T01 | 0.112312742 |
| 353 | MsG0280006462.01.T01 | MsG0580025222.01.T04 | 0.145855886 |
| 354 | MsG0280006462.01.T01 | MsG0580025232.01.T01 | 0.121155437 |
| 355 | MsG0280006462.01.T01 | MsG0580025295.01.T01 | 0.123259229 |
| 356 | MsG0280006462.01.T01 | MsG0580025337.01.T01 | 0.12424519 |
| 357 | MsG0280006462.01.T01 | MsG0580025340.01.T01 | 0.166865097 |
| 358 | MsG0280006462.01.T01 | MsG0580025445.01.T01 | 0.1100375 |
| 359 | MsG0280006462.01.T01 | MsG0580025490.01.T01 | 0.192312863 |
| 360 | MsG0280006462.01.T01 | MsG0580025649.01.T01 | 0.201737217 |
| 361 | MsG0280006462.01.T01 | MsG0580025649.01.T02 | 0.201737217 |
| 362 | MsG0280006462.01.T01 | MsG0580025652.01.T01 | 0.163357471 |
| 363 | MsG0280006462.01.T01 | MsG0580025657.01.T01 | 0.114530488 |
| 364 | MsG0280006462.01.T01 | MsG0580025657.01.T02 | 0.114530488 |
| 365 | MsG0280006462.01.T01 | MsG0580025657.01.T03 | 0.114530488 |
| 366 | MsG0280006462.01.T01 | MsG0580025657.01.T04 | 0.114530488 |
| 367 | MsG0280006462.01.T01 | MsG0580025657.01.T05 | 0.114530488 |
| 368 | MsG0280006462.01.T01 | MsG0580025657.01.T06 | 0.114530488 |
| 369 | MsG0280006462.01.T01 | MsG0580025657.01.T07 | 0.114530488 |
| 370 | MsG0280006462.01.T01 | MsG0580025657.01.T08 | 0.114530488 |
| 371 | MsG0280006462.01.T01 | MsG0580025657.01.T09 | 0.15623025 |
| 372 | MsG0280006462.01.T01 | MsG0580025657.01.T10 | 0.15623025 |
| 373 | MsG0280006462.01.T01 | MsG0580025657.01.T11 | 0.15623025 |
| 374 | MsG0280006462.01.T01 | MsG0580025657.01.T12 | 0.15623025 |
| 375 | MsG0280006462.01.T01 | MsG0580025690.01.T01 | 0.107600383 |
| 376 | MsG0280006462.01.T01 | MsG0580025732.01.T01 | 0.131301059 |
| 377 | MsG0280006462.01.T01 | MsG0580025768.01.T01 | 0.179441999 |
| 378 | MsG0280006462.01.T01 | MsG0580025813.01.T01 | 0.118344154 |
| 379 | MsG0280006462.01.T01 | MsG0580025906.01.T01 | 0.180088001 |
| 380 | MsG0280006462.01.T01 | MsG0580025931.01.T01 | 0.109826985 |
| 381 | MsG0280006462.01.T01 | MsG0580026049.01.T01 | 0.123281287 |
| 382 | MsG0280006462.01.T01 | MsG0580026257.01.T01 | 0.125915398 |
| 383 | MsG0280006462.01.T01 | MsG0580026580.01.T01 | 0.15185152 |
| 384 | MsG0280006462.01.T01 | MsG0580026596.01.T01 | 0.201964056 |
| 385 | MsG0280006462.01.T01 | MsG0580026962.01.T01 | 0.145235318 |
| 386 | MsG0280006462.01.T01 | MsG0580027038.01.T01 | 0.115350477 |
| 387 | MsG0280006462.01.T01 | MsG0580027343.01.T01 | 0.164381229 |
| 388 | MsG0280006462.01.T01 | MsG0580027459.01.T01 | 0.14731115 |
| 389 | MsG0280006462.01.T01 | MsG0580027464.01.T01 | 0.164081662 |
| 390 | MsG0280006462.01.T01 | MsG0580027653.01.T01 | 0.121064409 |
| 391 | MsG0280006462.01.T01 | MsG0580027733.01.T01 | 0.10708151 |
| 392 | MsG0280006462.01.T01 | MsG0580027774.01.T01 | 0.1296705 |
| 393 | MsG0280006462.01.T01 | MsG0580027881.01.T01 | 0.212455837 |
| 394 | MsG0280006462.01.T01 | MsG0580027904.01.T01 | 0.125590944 |
| 395 | MsG0280006462.01.T01 | MsG0580027926.01.T01 | 0.198408374 |
| 396 | MsG0280006462.01.T01 | MsG0580027964.01.T01 | 0.104263696 |
| 397 | MsG0280006462.01.T01 | MsG0580028033.01.T01 | 0.133379999 |
| 398 | MsG0280006462.01.T01 | MsG0580028059.01.T01 | 0.156768268 |
| 399 | MsG0280006462.01.T01 | MsG0580028090.01.T01 | 0.136981097 |
| 400 | MsG0280006462.01.T01 | MsG0580028109.01.T01 | 0.128722874 |
| 401 | MsG0280006462.01.T01 | MsG0580028127.01.T01 | 0.133683074 |
| 402 | MsG0280006462.01.T01 | MsG0580028160.01.T01 | 0.122512783 |
| 403 | MsG0280006462.01.T01 | MsG0580028236.01.T01 | 0.124422982 |
| 404 | MsG0280006462.01.T01 | MsG0580028250.01.T01 | 0.133869239 |
| 405 | MsG0280006462.01.T01 | MsG0580028266.01.T01 | 0.138342301 |
| 406 | MsG0280006462.01.T01 | MsG0580028280.01.T01 | 0.202876988 |
| 407 | MsG0280006462.01.T01 | MsG0580028280.01.T02 | 0.202876988 |
| 408 | MsG0280006462.01.T01 | MsG0580028300.01.T01 | 0.115717339 |
| 409 | MsG0280006462.01.T01 | MsG0580028328.01.T01 | 0.109254303 |
| 410 | MsG0280006462.01.T01 | MsG0580028360.01.T01 | 0.16274582 |
| 411 | MsG0280006462.01.T01 | MsG0580028433.01.T01 | 0.13335782 |
| 412 | MsG0280006462.01.T01 | MsG0580028480.01.T01 | 0.164376039 |
| 413 | MsG0280006462.01.T01 | MsG0580028495.01.T01 | 0.110758935 |
| 414 | MsG0280006462.01.T01 | MsG0580028560.01.T01 | 0.124527258 |
| 415 | MsG0280006462.01.T01 | MsG0580028602.01.T01 | 0.103722991 |
| 416 | MsG0280006462.01.T01 | MsG0580028850.01.T01 | 0.105996586 |
| 417 | MsG0280006462.01.T01 | MsG0580028912.01.T01 | 0.153093402 |
| 418 | MsG0280006462.01.T01 | MsG0580028947.01.T01 | 0.139215146 |
| 419 | MsG0280006462.01.T01 | MsG0580029028.01.T01 | 0.184602801 |
| 420 | MsG0280006462.01.T01 | MsG0580029256.01.T01 | 0.10063217 |
| 421 | MsG0280006462.01.T01 | MsG0580029305.01.T01 | 0.185342812 |
| 422 | MsG0280006462.01.T01 | MsG0580029375.01.T01 | 0.122325641 |
| 423 | MsG0280006462.01.T01 | MsG0580029376.01.T01 | 0.173663781 |
| 424 | MsG0280006462.01.T01 | MsG0580029467.01.T01 | 0.112597999 |
| 425 | MsG0280006462.01.T01 | MsG0580029477.01.T01 | 0.165513008 |
| 426 | MsG0280006462.01.T01 | MsG0580029491.01.T01 | 0.138097476 |
| 427 | MsG0280006462.01.T01 | MsG0580029570.01.T01 | 0.124725195 |
| 428 | MsG0280006462.01.T01 | MsG0580029614.01.T01 | 0.182194035 |
| 429 | MsG0280006462.01.T01 | MsG0580029639.01.T01 | 0.213557486 |
| 430 | MsG0280006462.01.T01 | MsG0580029990.01.T01 | 0.141219571 |
| 431 | MsG0280006462.01.T01 | MsG0580030182.01.T01 | 0.129491115 |
| 432 | MsG0280006462.01.T01 | MsG0580030199.01.T01 | 0.116383616 |
| 433 | MsG0280006462.01.T01 | MsG0580030199.01.T02 | 0.116383616 |
| 434 | MsG0280006462.01.T01 | MsG0680030316.01.T01 | 0.143460409 |
| 435 | MsG0280006462.01.T01 | MsG0680030361.01.T01 | 0.193372828 |
| 436 | MsG0280006462.01.T01 | MsG0680030468.01.T01 | 0.107558794 |
| 437 | MsG0280006462.01.T01 | MsG0680030504.01.T01 | 0.163160695 |
| 438 | MsG0280006462.01.T01 | MsG0680030525.01.T01 | 0.145385304 |
| 439 | MsG0280006462.01.T01 | MsG0680030619.01.T01 | 0.188865152 |
| 440 | MsG0280006462.01.T01 | MsG0680030639.01.T01 | 0.118145934 |
| 441 | MsG0280006462.01.T01 | MsG0680030660.01.T01 | 0.163265199 |
| 442 | MsG0280006462.01.T01 | MsG0680030731.01.T01 | 0.113423041 |
| 443 | MsG0280006462.01.T01 | MsG0680030780.01.T01 | 0.157599475 |
| 444 | MsG0280006462.01.T01 | MsG0680030786.01.T01 | 0.163097472 |
| 445 | MsG0280006462.01.T01 | MsG0680030842.01.T01 | 0.184227303 |
| 446 | MsG0280006462.01.T01 | MsG0680030858.01.T01 | 0.125596686 |
| 447 | MsG0280006462.01.T01 | MsG0680030975.01.T01 | 0.145276864 |
| 448 | MsG0280006462.01.T01 | MsG0680031017.01.T01 | 0.102148302 |
| 449 | MsG0280006462.01.T01 | MsG0680031055.01.T01 | 0.14353378 |
| 450 | MsG0280006462.01.T01 | MsG0680031058.01.T01 | 0.108091072 |
| 451 | MsG0280006462.01.T01 | MsG0680031139.01.T01 | 0.120649034 |
| 452 | MsG0280006462.01.T01 | MsG0680031274.01.T01 | 0.173741614 |
| 453 | MsG0280006462.01.T01 | MsG0680031363.01.T01 | 0.190712648 |
| 454 | MsG0280006462.01.T01 | MsG0680031389.01.T01 | 0.135717576 |
| 455 | MsG0280006462.01.T01 | MsG0680031638.01.T01 | 0.147077928 |
| 456 | MsG0280006462.01.T01 | MsG0680031685.01.T01 | 0.101545653 |
| 457 | MsG0280006462.01.T01 | MsG0680031716.01.T01 | 0.123403546 |
| 458 | MsG0280006462.01.T01 | MsG0680031790.01.T01 | 0.2068825 |
| 459 | MsG0280006462.01.T01 | MsG0680031805.01.T01 | 0.161204218 |
| 460 | MsG0280006462.01.T01 | MsG0680031816.01.T01 | 0.148027141 |
| 461 | MsG0280006462.01.T01 | MsG0680031926.01.T01 | 0.109106302 |
| 462 | MsG0280006462.01.T01 | MsG0680031944.01.T01 | 0.162754818 |
| 463 | MsG0280006462.01.T01 | MsG0680032017.01.T01 | 0.172245272 |
| 464 | MsG0280006462.01.T01 | MsG0680032031.01.T02 | 0.135250569 |
| 465 | MsG0280006462.01.T01 | MsG0680032031.01.T03 | 0.113784324 |
| 466 | MsG0280006462.01.T01 | MsG0680032165.01.T09 | 0.18578271 |
| 467 | MsG0280006462.01.T01 | MsG0680032758.01.T01 | 0.140606148 |
| 468 | MsG0280006462.01.T01 | MsG0680032814.01.T01 | 0.122904507 |
| 469 | MsG0280006462.01.T01 | MsG0680032830.01.T01 | 0.14271699 |
| 470 | MsG0280006462.01.T01 | MsG0680032846.01.T01 | 0.133802842 |
| 471 | MsG0280006462.01.T01 | MsG0680032994.01.T01 | 0.125790393 |
| 472 | MsG0280006462.01.T01 | MsG0680033030.01.T01 | 0.12119411 |
| 473 | MsG0280006462.01.T01 | MsG0680033250.01.T01 | 0.195960793 |
| 474 | MsG0280006462.01.T01 | MsG0680033302.01.T01 | 0.151812705 |
| 475 | MsG0280006462.01.T01 | MsG0680033563.01.T01 | 0.120758509 |
| 476 | MsG0280006462.01.T01 | MsG0680033616.01.T01 | 0.182315923 |
| 477 | MsG0280006462.01.T01 | MsG0680033744.01.T01 | 0.204159946 |
| 478 | MsG0280006462.01.T01 | MsG0680033745.01.T01 | 0.183901481 |
| 479 | MsG0280006462.01.T01 | MsG0680033752.01.T01 | 0.127707095 |
| 480 | MsG0280006462.01.T01 | MsG0680033892.01.T01 | 0.112184246 |
| 481 | MsG0280006462.01.T01 | MsG0680034112.01.T01 | 0.155966817 |
| 482 | MsG0280006462.01.T01 | MsG0680034132.01.T01 | 0.18449162 |
| 483 | MsG0280006462.01.T01 | MsG0680034269.01.T01 | 0.10272075 |
| 484 | MsG0280006462.01.T01 | MsG0680034320.01.T01 | 0.104971696 |
| 485 | MsG0280006462.01.T01 | MsG0680034482.01.T01 | 0.10012159 |
| 486 | MsG0280006462.01.T01 | MsG0680034489.01.T01 | 0.174778424 |
| 487 | MsG0280006462.01.T01 | MsG0680034617.01.T01 | 0.161268776 |
| 488 | MsG0280006462.01.T01 | MsG0680034675.01.T01 | 0.172559013 |
| 489 | MsG0280006462.01.T01 | MsG0680034866.01.T01 | 0.160623233 |
| 490 | MsG0280006462.01.T01 | MsG0680035322.01.T01 | 0.141279448 |
| 491 | MsG0280006462.01.T01 | MsG0680035351.01.T01 | 0.168826738 |
| 492 | MsG0280006462.01.T01 | MsG0680035528.01.T01 | 0.132536482 |
| 493 | MsG0280006462.01.T01 | MsG0680035552.01.T03 | 0.13290374 |
| 494 | MsG0280006462.01.T01 | MsG0680035570.01.T01 | 0.116801567 |
| 495 | MsG0280006462.01.T01 | MsG0680035614.01.T01 | 0.102057934 |
| 496 | MsG0280006462.01.T01 | MsG0680035784.01.T01 | 0.129807161 |
| 497 | MsG0280006462.01.T01 | MsG0680035832.01.T01 | 0.128688534 |
| 498 | MsG0280006462.01.T01 | MsG0680035858.01.T01 | 0.163000195 |
| 499 | MsG0280006462.01.T01 | MsG0680035893.01.T01 | 0.135080419 |
| 500 | MsG0280006462.01.T01 | MsG0680035909.01.T01 | 0.2235773 |
| 501 | MsG0280006462.01.T01 | MsG0780035991.01.T01 | 0.124903884 |
| 502 | MsG0280006462.01.T01 | MsG0780035991.01.T02 | 0.124903884 |
| 503 | MsG0280006462.01.T01 | MsG0780036017.01.T01 | 0.165252323 |
| 504 | MsG0280006462.01.T01 | MsG0780036024.01.T01 | 0.128397253 |
| 505 | MsG0280006462.01.T01 | MsG0780036029.01.T01 | 0.149395862 |
| 506 | MsG0280006462.01.T01 | MsG0780036090.01.T01 | 0.15485553 |
| 507 | MsG0280006462.01.T01 | MsG0780036090.01.T02 | 0.15485553 |
| 508 | MsG0280006462.01.T01 | MsG0780036170.01.T01 | 0.192719749 |
| 509 | MsG0280006462.01.T01 | MsG0780036239.01.T01 | 0.161973629 |
| 510 | MsG0280006462.01.T01 | MsG0780036309.01.T01 | 0.179238106 |
| 511 | MsG0280006462.01.T01 | MsG0780036322.01.T01 | 0.124439509 |
| 512 | MsG0280006462.01.T01 | MsG0780036507.01.T01 | 0.134607029 |
| 513 | MsG0280006462.01.T01 | MsG0780036514.01.T01 | 0.110630695 |
| 514 | MsG0280006462.01.T01 | MsG0780036689.01.T01 | 0.144457702 |
| 515 | MsG0280006462.01.T01 | MsG0780036851.01.T01 | 0.103311409 |
| 516 | MsG0280006462.01.T01 | MsG0780036863.01.T01 | 0.161834877 |
| 517 | MsG0280006462.01.T01 | MsG0780036866.01.T01 | 0.179405304 |
| 518 | MsG0280006462.01.T01 | MsG0780036876.01.T01 | 0.104107042 |
| 519 | MsG0280006462.01.T01 | MsG0780036876.01.T02 | 0.104107042 |
| 520 | MsG0280006462.01.T01 | MsG0780036890.01.T04 | 0.10076325 |
| 521 | MsG0280006462.01.T01 | MsG0780036925.01.T01 | 0.117727745 |
| 522 | MsG0280006462.01.T01 | MsG0780036925.01.T02 | 0.117727745 |
| 523 | MsG0280006462.01.T01 | MsG0780036939.01.T01 | 0.143578349 |
| 524 | MsG0280006462.01.T01 | MsG0780036939.01.T02 | 0.143578349 |
| 525 | MsG0280006462.01.T01 | MsG0780037054.01.T01 | 0.143486317 |
| 526 | MsG0280006462.01.T01 | MsG0780037141.01.T01 | 0.113014286 |
| 527 | MsG0280006462.01.T01 | MsG0780037189.01.T01 | 0.143352364 |
| 528 | MsG0280006462.01.T01 | MsG0780037343.01.T01 | 0.114530488 |
| 529 | MsG0280006462.01.T01 | MsG0780037343.01.T02 | 0.114530488 |
| 530 | MsG0280006462.01.T01 | MsG0780037343.01.T03 | 0.114530488 |
| 531 | MsG0280006462.01.T01 | MsG0780037343.01.T04 | 0.114530488 |
| 532 | MsG0280006462.01.T01 | MsG0780037343.01.T05 | 0.114530488 |
| 533 | MsG0280006462.01.T01 | MsG0780037343.01.T06 | 0.114530488 |
| 534 | MsG0280006462.01.T01 | MsG0780037343.01.T07 | 0.114530488 |
| 535 | MsG0280006462.01.T01 | MsG0780037343.01.T08 | 0.114530488 |
| 536 | MsG0280006462.01.T01 | MsG0780037343.01.T09 | 0.100465284 |
| 537 | MsG0280006462.01.T01 | MsG0780037364.01.T01 | 0.152772394 |
| 538 | MsG0280006462.01.T01 | MsG0780037895.01.T01 | 0.17909061 |
| 539 | MsG0280006462.01.T01 | MsG0780037919.01.T01 | 0.105176443 |
| 540 | MsG0280006462.01.T01 | MsG0780037920.01.T01 | 0.164011109 |
| 541 | MsG0280006462.01.T01 | MsG0780037927.01.T01 | 0.174267063 |
| 542 | MsG0280006462.01.T01 | MsG0780037930.01.T01 | 0.174267063 |
| 543 | MsG0280006462.01.T01 | MsG0780038113.01.T01 | 0.125834597 |
| 544 | MsG0280006462.01.T01 | MsG0780038277.01.T01 | 0.122932755 |
| 545 | MsG0280006462.01.T01 | MsG0780038277.01.T02 | 0.122932755 |
| 546 | MsG0280006462.01.T01 | MsG0780038305.01.T01 | 0.145961033 |
| 547 | MsG0280006462.01.T01 | MsG0780038389.01.T01 | 0.198887695 |
| 548 | MsG0280006462.01.T01 | MsG0780038564.01.T01 | 0.1867284 |
| 549 | MsG0280006462.01.T01 | MsG0780038570.01.T01 | 0.192651998 |
| 550 | MsG0280006462.01.T01 | MsG0780038743.01.T02 | 0.134385284 |
| 551 | MsG0280006462.01.T01 | MsG0780038770.01.T01 | 0.101223468 |
| 552 | MsG0280006462.01.T01 | MsG0780039144.01.T01 | 0.245184899 |
| 553 | MsG0280006462.01.T01 | MsG0780039171.01.T01 | 0.125954252 |
| 554 | MsG0280006462.01.T01 | MsG0780039603.01.T01 | 0.100634486 |
| 555 | MsG0280006462.01.T01 | MsG0780039740.01.T01 | 0.114076572 |
| 556 | MsG0280006462.01.T01 | MsG0780039783.01.T01 | 0.207000883 |
| 557 | MsG0280006462.01.T01 | MsG0780039848.01.T01 | 0.155940699 |
| 558 | MsG0280006462.01.T01 | MsG0780039863.01.T01 | 0.1826526 |
| 559 | MsG0280006462.01.T01 | MsG0780039994.01.T01 | 0.110657073 |
| 560 | MsG0280006462.01.T01 | MsG0780040173.01.T01 | 0.105032943 |
| 561 | MsG0280006462.01.T01 | MsG0780040173.01.T02 | 0.105032943 |
| 562 | MsG0280006462.01.T01 | MsG0780040173.01.T03 | 0.105032943 |
| 563 | MsG0280006462.01.T01 | MsG0780040257.01.T01 | 0.108868196 |
| 564 | MsG0280006462.01.T01 | MsG0780040373.01.T01 | 0.147568609 |
| 565 | MsG0280006462.01.T01 | MsG0780040418.01.T01 | 0.100584622 |
| 566 | MsG0280006462.01.T01 | MsG0780040425.01.T01 | 0.131603298 |
| 567 | MsG0280006462.01.T01 | MsG0780040531.01.T01 | 0.154354302 |
| 568 | MsG0280006462.01.T01 | MsG0780040742.01.T01 | 0.183450389 |
| 569 | MsG0280006462.01.T01 | MsG0780040806.01.T01 | 0.150854401 |
| 570 | MsG0280006462.01.T01 | MsG0780040826.01.T01 | 0.165341015 |
| 571 | MsG0280006462.01.T01 | MsG0780040827.01.T01 | 0.139270839 |
| 572 | MsG0280006462.01.T01 | MsG0780040864.01.T01 | 0.142527292 |
| 573 | MsG0280006462.01.T01 | MsG0780040904.01.T02 | 0.137314285 |
| 574 | MsG0280006462.01.T01 | MsG0780041002.01.T03 | 0.100225999 |
| 575 | MsG0280006462.01.T01 | MsG0780041019.01.T01 | 0.178036521 |
| 576 | MsG0280006462.01.T01 | MsG0780041033.01.T01 | 0.189746359 |
| 577 | MsG0280006462.01.T01 | MsG0780041241.01.T01 | 0.198158384 |
| 578 | MsG0280006462.01.T01 | MsG0780041276.01.T01 | 0.124371977 |
| 579 | MsG0280006462.01.T01 | MsG0780041276.01.T02 | 0.124371977 |
| 580 | MsG0280006462.01.T01 | MsG0780041276.01.T03 | 0.124371977 |
| 581 | MsG0280006462.01.T01 | MsG0780041276.01.T04 | 0.142061829 |
| 582 | MsG0280006462.01.T01 | MsG0780041276.01.T05 | 0.142061829 |
| 583 | MsG0280006462.01.T01 | MsG0780041276.01.T06 | 0.142061829 |
| 584 | MsG0280006462.01.T01 | MsG0780041277.01.T01 | 0.210005896 |
| 585 | MsG0280006462.01.T01 | MsG0780041278.01.T01 | 0.134597504 |
| 586 | MsG0280006462.01.T01 | MsG0780041335.01.T01 | 0.178953109 |
| 587 | MsG0280006462.01.T01 | MsG0780041594.01.T01 | 0.184416038 |
| 588 | MsG0280006462.01.T01 | MsG0780041613.01.T01 | 0.142542241 |
| 589 | MsG0280006462.01.T01 | MsG0780041696.01.T01 | 0.179218609 |
| 590 | MsG0280006462.01.T01 | MsG0780041696.01.T02 | 0.179218609 |
| 591 | MsG0280006462.01.T01 | MsG0780041696.01.T04 | 0.179218609 |
| 592 | MsG0280006462.01.T01 | MsG0780041696.01.T05 | 0.12643295 |
| 593 | MsG0280006462.01.T01 | MsG0780041790.01.T01 | 0.130715601 |
| 594 | MsG0280006462.01.T01 | MsG0780041803.01.T01 | 0.120359914 |
| 595 | MsG0280006462.01.T01 | MsG0880041901.01.T01 | 0.220335474 |
| 596 | MsG0280006462.01.T01 | MsG0880041916.01.T01 | 0.115740774 |
| 597 | MsG0280006462.01.T01 | MsG0880042055.01.T01 | 0.119979662 |
| 598 | MsG0280006462.01.T01 | MsG0880042301.01.T01 | 0.16233563 |
| 599 | MsG0280006462.01.T01 | MsG0880042370.01.T01 | 0.204235759 |
| 600 | MsG0280006462.01.T01 | MsG0880042421.01.T01 | 0.102715618 |
| 601 | MsG0280006462.01.T01 | MsG0880042523.01.T01 | 0.220288975 |
| 602 | MsG0280006462.01.T01 | MsG0880042688.01.T01 | 0.122883589 |
| 603 | MsG0280006462.01.T01 | MsG0880042762.01.T01 | 0.144649432 |
| 604 | MsG0280006462.01.T01 | MsG0880042768.01.T01 | 0.118877936 |
| 605 | MsG0280006462.01.T01 | MsG0880042825.01.T01 | 0.219369821 |
| 606 | MsG0280006462.01.T01 | MsG0880042850.01.T01 | 0.180777474 |
| 607 | MsG0280006462.01.T01 | MsG0880042880.01.T01 | 0.140248527 |
| 608 | MsG0280006462.01.T01 | MsG0880042933.01.T01 | 0.127573771 |
| 609 | MsG0280006462.01.T01 | MsG0880043003.01.T01 | 0.120460786 |
| 610 | MsG0280006462.01.T01 | MsG0880043031.01.T01 | 0.107312105 |
| 611 | MsG0280006462.01.T01 | MsG0880043119.01.T01 | 0.142001462 |
| 612 | MsG0280006462.01.T01 | MsG0880043125.01.T01 | 0.106218676 |
| 613 | MsG0280006462.01.T01 | MsG0880043125.01.T02 | 0.106218676 |
| 614 | MsG0280006462.01.T01 | MsG0880043145.01.T01 | 0.216955724 |
| 615 | MsG0280006462.01.T01 | MsG0880043204.01.T01 | 0.113316552 |
| 616 | MsG0280006462.01.T01 | MsG0880043253.01.T01 | 0.111765003 |
| 617 | MsG0280006462.01.T01 | MsG0880043303.01.T01 | 0.113181016 |
| 618 | MsG0280006462.01.T01 | MsG0880043465.01.T01 | 0.139615557 |
| 619 | MsG0280006462.01.T01 | MsG0880043466.01.T01 | 0.143175241 |
| 620 | MsG0280006462.01.T01 | MsG0880043499.01.T01 | 0.138331111 |
| 621 | MsG0280006462.01.T01 | MsG0880043500.01.T01 | 0.126398462 |
| 622 | MsG0280006462.01.T01 | MsG0880043511.01.T01 | 0.168783416 |
| 623 | MsG0280006462.01.T01 | MsG0880043653.01.T01 | 0.202254029 |
| 624 | MsG0280006462.01.T01 | MsG0880043824.01.T01 | 0.152114058 |
| 625 | MsG0280006462.01.T01 | MsG0880043915.01.T01 | 0.110946751 |
| 626 | MsG0280006462.01.T01 | MsG0880044418.01.T01 | 0.13050462 |
| 627 | MsG0280006462.01.T01 | MsG0880044452.01.T01 | 0.126297213 |
| 628 | MsG0280006462.01.T01 | MsG0880044566.01.T01 | 0.150489055 |
| 629 | MsG0280006462.01.T01 | MsG0880044575.01.T01 | 0.161744757 |
| 630 | MsG0280006462.01.T01 | MsG0880044640.01.T01 | 0.143884523 |
| 631 | MsG0280006462.01.T01 | MsG0880044691.01.T01 | 0.149801276 |
| 632 | MsG0280006462.01.T01 | MsG0880044763.01.T01 | 0.153387349 |
| 633 | MsG0280006462.01.T01 | MsG0880044861.01.T01 | 0.10988275 |
| 634 | MsG0280006462.01.T01 | MsG0880044902.01.T01 | 0.113466674 |
| 635 | MsG0280006462.01.T01 | MsG0880044946.01.T01 | 0.164777651 |
| 636 | MsG0280006462.01.T01 | MsG0880044950.01.T01 | 0.182525483 |
| 637 | MsG0280006462.01.T01 | MsG0880045006.01.T01 | 0.17257383 |
| 638 | MsG0280006462.01.T01 | MsG0880045006.01.T02 | 0.17257383 |
| 639 | MsG0280006462.01.T01 | MsG0880045029.01.T01 | 0.155643169 |
| 640 | MsG0280006462.01.T01 | MsG0880045118.01.T01 | 0.100028177 |
| 641 | MsG0280006462.01.T01 | MsG0880045240.01.T01 | 0.101260878 |
| 642 | MsG0280006462.01.T01 | MsG0880045244.01.T01 | 0.143589013 |
| 643 | MsG0280006462.01.T01 | MsG0880045291.01.T01 | 0.103268982 |
| 644 | MsG0280006462.01.T01 | MsG0880045520.01.T01 | 0.158482336 |
| 645 | MsG0280006462.01.T01 | MsG0880045551.01.T01 | 0.103974121 |
| 646 | MsG0280006462.01.T01 | MsG0880045591.01.T01 | 0.128417921 |
| 647 | MsG0280006462.01.T01 | MsG0880045657.01.T01 | 0.17066882 |
| 648 | MsG0280006462.01.T01 | MsG0880045783.01.T01 | 0.153002719 |
| 649 | MsG0280006462.01.T01 | MsG0880045818.01.T01 | 0.102676267 |
| 650 | MsG0280006462.01.T01 | MsG0880045850.01.T01 | 0.15309274 |
| 651 | MsG0280006462.01.T01 | MsG0880045894.01.T01 | 0.153272229 |
| 652 | MsG0280006462.01.T01 | MsG0880046042.01.T01 | 0.113804114 |
| 653 | MsG0280006462.01.T01 | MsG0880046077.01.T01 | 0.14051965 |
| 654 | MsG0280006462.01.T01 | MsG0880046127.01.T01 | 0.160599051 |
| 655 | MsG0280006462.01.T01 | MsG0880046127.01.T02 | 0.160599051 |
| 656 | MsG0280006462.01.T01 | MsG0880046226.01.T01 | 0.118319015 |
| 657 | MsG0280006462.01.T01 | MsG0880046298.01.T01 | 0.200793684 |
| 658 | MsG0280006462.01.T01 | MsG0880046329.01.T01 | 0.186391524 |
| 659 | MsG0280006462.01.T01 | MsG0880046368.01.T01 | 0.180371746 |
| 660 | MsG0280006462.01.T01 | MsG0880046377.01.T01 | 0.137724953 |
| 661 | MsG0280006462.01.T01 | MsG0880046436.01.T01 | 0.102771293 |
| 662 | MsG0280006462.01.T01 | MsG0880046441.01.T01 | 0.124360757 |
| 663 | MsG0280006462.01.T01 | MsG0880046542.01.T01 | 0.19798689 |
| 664 | MsG0280006462.01.T01 | MsG0880046544.01.T01 | 0.194949352 |
| 665 | MsG0280006462.01.T01 | MsG0880046559.01.T01 | 0.133631685 |
| 666 | MsG0280006462.01.T01 | MsG0880046606.01.T01 | 0.160010059 |
| 667 | MsG0280006462.01.T01 | MsG0880046662.01.T01 | 0.17507978 |
| 668 | MsG0280006462.01.T01 | MsG0880046764.01.T01 | 0.143861676 |
| 669 | MsG0280006462.01.T01 | MsG0880046767.01.T01 | 0.124450347 |
| 670 | MsG0280006462.01.T01 | MsG0880046844.01.T01 | 0.110352073 |
| 671 | MsG0280006462.01.T01 | MsG0880046849.01.T01 | 0.123840032 |
| 672 | MsG0280006462.01.T01 | MsG0880046862.01.T01 | 0.131943422 |
| 673 | MsG0280006462.01.T01 | MsG0880046864.01.T01 | 0.113495901 |
| 674 | MsG0280006462.01.T01 | MsG0880046918.01.T02 | 0.125822206 |
| 675 | MsG0280006462.01.T01 | MsG0880046990.01.T01 | 0.187475259 |
| 676 | MsG0280006462.01.T01 | MsG0880047046.01.T01 | 0.115039568 |
| 677 | MsG0280006462.01.T01 | MsG0880047074.01.T01 | 0.1206707 |
| 678 | MsG0280006462.01.T01 | MsG0880047075.01.T01 | 0.148576663 |
| 679 | MsG0280006462.01.T01 | MsG0880047099.01.T02 | 0.117122988 |
| 680 | MsG0280006462.01.T01 | MsG0880047117.01.T01 | 0.164781013 |
| 681 | MsG0280006462.01.T01 | MsG0880047136.01.T01 | 0.203990256 |
| 682 | MsG0280006462.01.T01 | MsG0880047148.01.T01 | 0.199724338 |
| 683 | MsG0280006462.01.T01 | MsG0880047216.01.T03 | 0.150013906 |
| 684 | MsG0280006462.01.T01 | MsG0880047217.01.T01 | 0.152831912 |
| 685 | MsG0280006462.01.T01 | MsG0880047219.01.T01 | 0.108900495 |
| 686 | MsG0280006462.01.T01 | MsG0880047313.01.T01 | 0.105324413 |
| 687 | MsG0280006462.01.T01 | MsG0880047323.01.T01 | 0.186964819 |
| 688 | MsG0280006462.01.T01 | MsG0880047323.01.T02 | 0.186964819 |
| 689 | MsG0280006462.01.T01 | MsG0880047323.01.T03 | 0.186688859 |
| 690 | MsG0280006462.01.T01 | MsG0880047323.01.T04 | 0.184725013 |
| 691 | MsG0280006462.01.T01 | MsG0880047376.01.T01 | 0.175331497 |
| 692 | MsG0280006462.01.T01 | MsG0880047419.01.T01 | 0.137399971 |
| 693 | MsG0280006462.01.T01 | MsG0880047601.01.T01 | 0.144750638 |
| 694 | MsG0280006462.01.T01 | MsG0880047601.01.T02 | 0.144750638 |
| 695 | MsG0280006462.01.T01 | MsG0880047672.01.T01 | 0.124558622 |
| 696 | MsG0280006462.01.T01 | MsG0880047745.01.T01 | 0.121306192 |
| 697 | MsG0280006462.01.T01 | MsG0080047786.01.T01 | 0.194541771 |
| 698 | MsG0280006462.01.T01 | MsG0080047787.01.T01 | 0.116934851 |
| 699 | MsG0280006462.01.T01 | MsG0080047906.01.T01 | 0.178892973 |
| 700 | MsG0280006462.01.T01 | MsG0080047906.01.T02 | 0.178892973 |
| 701 | MsG0280006462.01.T01 | MsG0080047992.01.T01 | 0.166185115 |
| 702 | MsG0280006462.01.T01 | MsG0080047994.01.T01 | 0.162002458 |
| 703 | MsG0280006462.01.T01 | MsG0080047996.01.T01 | 0.141185213 |
| 704 | MsG0280006462.01.T01 | MsG0080048038.01.T01 | 0.143971586 |
| 705 | MsG0280006462.01.T01 | MsG0080048132.01.T01 | 0.128885194 |
| 706 | MsG0280006462.01.T01 | MsG0080048202.01.T01 | 0.128906192 |
| 707 | MsG0280006462.01.T01 | MsG0080048202.01.T02 | 0.128906192 |
| 708 | MsG0280006462.01.T01 | MsG0080048202.01.T03 | 0.128906192 |
| 709 | MsG0280006462.01.T01 | MsG0080048202.01.T04 | 0.128906192 |
| 710 | MsG0280006462.01.T01 | MsG0080048202.01.T05 | 0.128906192 |
| 711 | MsG0280006462.01.T01 | MsG0080048202.01.T06 | 0.128906192 |
| 712 | MsG0280006462.01.T01 | MsG0080048202.01.T07 | 0.194660997 |
| 713 | MsG0280006462.01.T01 | MsG0080048202.01.T08 | 0.172090491 |
| 714 | MsG0280006462.01.T01 | MsG0080048202.01.T09 | 0.172090491 |
| 715 | MsG0280006462.01.T01 | MsG0080048215.01.T01 | 0.102991667 |
| 716 | MsG0280006462.01.T01 | MsG0080048216.01.T01 | 0.141429263 |
| 717 | MsG0280006462.01.T01 | MsG0080048337.01.T01 | 0.132176967 |
| 718 | MsG0280006462.01.T01 | MsG0080048382.01.T01 | 0.218417546 |
| 719 | MsG0280006462.01.T01 | MsG0080048440.01.T01 | 0.202012662 |
| 720 | MsG0280006462.01.T01 | MsG0080048596.01.T01 | 0.171235527 |
| 721 | MsG0280006462.01.T01 | MsG0080048634.01.T01 | 0.141562853 |
| 722 | MsG0280006462.01.T01 | MsG0080048703.01.T01 | 0.148115082 |
| 723 | MsG0280006462.01.T01 | MsG0080048858.01.T01 | 0.12830368 |
| 724 | MsG0280006462.01.T01 | MsG0080048886.01.T01 | 0.102444687 |
| 725 | MsG0280006462.01.T01 | MsG0080048938.01.T01 | 0.157432903 |
| 726 | MsG0280006462.01.T01 | MsG0080048948.01.T01 | 0.177210218 |
| 727 | MsG0280006462.01.T01 | MsG0080048964.01.T01 | 0.140988236 |
| 728 | MsG0280006462.01.T01 | MsG0080048983.01.T01 | 0.108513112 |
| 729 | MsG0280006462.01.T01 | MsG0080049108.01.T01 | 0.154587654 |
| 730 | MsG0280006462.01.T01 | MsG0080049111.01.T01 | 0.133333186 |
| 731 | MsG0280006462.01.T01 | MsG0180000002.01.T01 | 0.101885998 |
| 732 | MsG0280006462.01.T01 | MsG0180000100.01.T04 | 0.222340518 |
| 733 | MsG0280006462.01.T01 | MsG0180000181.01.T01 | 0.134917856 |
| 734 | MsG0280006462.01.T01 | MsG0180000274.01.T01 | 0.176336804 |
| 735 | MsG0280006462.01.T01 | MsG0180000305.01.T01 | 0.151483564 |
| 736 | MsG0280006462.01.T01 | MsG0180000461.01.T01 | 0.122477444 |
| 737 | MsG0280006462.01.T01 | MsG0180000462.01.T01 | 0.143328237 |
| 738 | MsG0280006462.01.T01 | MsG0180000476.01.T01 | 0.195514865 |
| 739 | MsG0280006462.01.T01 | MsG0180000554.01.T01 | 0.100409646 |
| 740 | MsG0280006462.01.T01 | MsG0180000629.01.T01 | 0.137143033 |
| 741 | MsG0280006462.01.T01 | MsG0180000663.01.T01 | 0.10769282 |
| 742 | MsG0280006462.01.T01 | MsG0180000670.01.T01 | 0.182486654 |
| 743 | MsG0280006462.01.T01 | MsG0180000674.01.T01 | 0.199366603 |
| 744 | MsG0280006462.01.T01 | MsG0180000681.01.T01 | 0.154035552 |
| 745 | MsG0280006462.01.T01 | MsG0180000681.01.T02 | 0.154035552 |
| 746 | MsG0280006462.01.T01 | MsG0180000681.01.T03 | 0.154035552 |
| 747 | MsG0280006462.01.T01 | MsG0180000681.01.T04 | 0.154944859 |
| 748 | MsG0280006462.01.T01 | MsG0180000681.01.T05 | 0.154944859 |
| 749 | MsG0280006462.01.T01 | MsG0180000707.01.T01 | 0.122106335 |
| 750 | MsG0280006462.01.T01 | MsG0180000818.01.T01 | 0.101840342 |
| 751 | MsG0280006462.01.T01 | MsG0180000974.01.T01 | 0.161668818 |
| 752 | MsG0280006462.01.T01 | MsG0180001062.01.T01 | 0.174784515 |
| 753 | MsG0280006462.01.T01 | MsG0180001126.01.T01 | 0.1080634 |
| 754 | MsG0280006462.01.T01 | MsG0180001176.01.T01 | 0.143914332 |
| 755 | MsG0280006462.01.T01 | MsG0180001176.01.T02 | 0.143914332 |
| 756 | MsG0280006462.01.T01 | MsG0180001201.01.T01 | 0.195221128 |
| 757 | MsG0280006462.01.T01 | MsG0180001373.01.T01 | 0.140046161 |
| 758 | MsG0280006462.01.T01 | MsG0180001721.01.T01 | 0.19321643 |
| 759 | MsG0280006462.01.T01 | MsG0180001732.01.T01 | 0.18260883 |
| 760 | MsG0280006462.01.T01 | MsG0180001795.01.T01 | 0.167602419 |
| 761 | MsG0280006462.01.T01 | MsG0180001816.01.T01 | 0.171978441 |
| 762 | MsG0280006462.01.T01 | MsG0180001938.01.T01 | 0.162897168 |
| 763 | MsG0280006462.01.T01 | MsG0180001938.01.T02 | 0.162897168 |
| 764 | MsG0280006462.01.T01 | MsG0180002024.01.T01 | 0.133692474 |
| 765 | MsG0280006462.01.T01 | MsG0180002050.01.T01 | 0.186117806 |
| 766 | MsG0280006462.01.T01 | MsG0180002237.01.T01 | 0.137789449 |
| 767 | MsG0280006462.01.T01 | MsG0180002249.01.T01 | 0.110450856 |
| 768 | MsG0280006462.01.T01 | MsG0180002365.01.T01 | 0.104616172 |
| 769 | MsG0280006462.01.T01 | MsG0180002451.01.T01 | 0.133789107 |
| 770 | MsG0280006462.01.T01 | MsG0180002459.01.T01 | 0.156030861 |
| 771 | MsG0280006462.01.T01 | MsG0180002477.01.T01 | 0.148549638 |
| 772 | MsG0280006462.01.T01 | MsG0180002900.01.T01 | 0.106054644 |
| 773 | MsG0280006462.01.T01 | MsG0180002911.01.T01 | 0.140326252 |
| 774 | MsG0280006462.01.T01 | MsG0180002924.01.T01 | 0.154129948 |
| 775 | MsG0280006462.01.T01 | MsG0180002976.01.T01 | 0.209101537 |
| 776 | MsG0280006462.01.T01 | MsG0180003129.01.T01 | 0.11090234 |
| 777 | MsG0280006462.01.T01 | MsG0180003214.01.T01 | 0.172003399 |
| 778 | MsG0280006462.01.T01 | MsG0180003413.01.T01 | 0.106251526 |
| 779 | MsG0280006462.01.T01 | MsG0180003425.01.T01 | 0.178130477 |
| 780 | MsG0280006462.01.T01 | MsG0180003428.01.T01 | 0.109019608 |
| 781 | MsG0280006462.01.T01 | MsG0180003464.01.T01 | 0.188598166 |
| 782 | MsG0280006462.01.T01 | MsG0180003464.01.T02 | 0.199396701 |
| 783 | MsG0280006462.01.T01 | MsG0180003471.01.T01 | 0.1191371 |
| 784 | MsG0280006462.01.T01 | MsG0180003513.01.T01 | 0.127690272 |
| 785 | MsG0280006462.01.T01 | MsG0180003527.01.T01 | 0.13780423 |
| 786 | MsG0280006462.01.T01 | MsG0180003580.01.T01 | 0.112355736 |
| 787 | MsG0280006462.01.T01 | MsG0180003599.01.T01 | 0.111003503 |
| 788 | MsG0280006462.01.T01 | MsG0180003620.01.T01 | 0.153355199 |
| 789 | MsG0280006462.01.T01 | MsG0180003620.01.T02 | 0.139220997 |
| 790 | MsG0280006462.01.T01 | MsG0180003638.01.T01 | 0.143258132 |
| 791 | MsG0280006462.01.T01 | MsG0180003674.01.T04 | 0.111986197 |
| 792 | MsG0280006462.01.T01 | MsG0180003767.01.T01 | 0.165931577 |
| 793 | MsG0280006462.01.T01 | MsG0180003862.01.T01 | 0.174586033 |
| 794 | MsG0280006462.01.T01 | MsG0180003864.01.T01 | 0.149758918 |
| 795 | MsG0280006462.01.T01 | MsG0180003940.01.T01 | 0.127685014 |
| 796 | MsG0280006462.01.T01 | MsG0180003940.01.T02 | 0.127685014 |
| 797 | MsG0280006462.01.T01 | MsG0180003950.01.T01 | 0.104387074 |
| 798 | MsG0280006462.01.T01 | MsG0180004002.01.T01 | 0.185498245 |
| 799 | MsG0280006462.01.T01 | MsG0180004003.01.T01 | 0.18646559 |
| 800 | MsG0280006462.01.T01 | MsG0180004004.01.T01 | 0.195589404 |
| 801 | MsG0280006462.01.T01 | MsG0180004021.01.T01 | 0.144215264 |
| 802 | MsG0280006462.01.T01 | MsG0180004021.01.T02 | 0.144215264 |
| 803 | MsG0280006462.01.T01 | MsG0180004021.01.T03 | 0.180882602 |
| 804 | MsG0280006462.01.T01 | MsG0180004030.01.T01 | 0.113771859 |
| 805 | MsG0280006462.01.T01 | MsG0180004030.01.T02 | 0.113771859 |
| 806 | MsG0280006462.01.T01 | MsG0180004030.01.T03 | 0.110585551 |
| 807 | MsG0280006462.01.T01 | MsG0180004030.01.T04 | 0.113771859 |
| 808 | MsG0280006462.01.T01 | MsG0180004053.01.T01 | 0.116991644 |
| 809 | MsG0280006462.01.T01 | MsG0180004055.01.T01 | 0.160989125 |
| 810 | MsG0280006462.01.T01 | MsG0180004057.01.T01 | 0.112418977 |
| 811 | MsG0280006462.01.T01 | MsG0180004372.01.T01 | 0.225890051 |
| 812 | MsG0280006462.01.T01 | MsG0180004425.01.T01 | 0.215375237 |
| 813 | MsG0280006462.01.T01 | MsG0180004483.01.T01 | 0.113409058 |
| 814 | MsG0280006462.01.T01 | MsG0180004519.01.T01 | 0.100502074 |
| 815 | MsG0280006462.01.T01 | MsG0180004525.01.T01 | 0.18982323 |
| 816 | MsG0280006462.01.T01 | MsG0180004758.01.T01 | 0.18782059 |
| 817 | MsG0280006462.01.T01 | MsG0180004758.01.T02 | 0.18782059 |
| 818 | MsG0280006462.01.T01 | MsG0180004777.01.T01 | 0.178104792 |
| 819 | MsG0280006462.01.T01 | MsG0180004807.01.T01 | 0.180592974 |
| 820 | MsG0280006462.01.T01 | MsG0180004810.01.T01 | 0.198817333 |
| 821 | MsG0280006462.01.T01 | MsG0180004843.01.T01 | 0.110672219 |
| 822 | MsG0280006462.01.T01 | MsG0180004858.01.T01 | 0.111826634 |
| 823 | MsG0280006462.01.T01 | MsG0180004874.01.T01 | 0.199463575 |
| 824 | MsG0280006462.01.T01 | MsG0180004904.01.T01 | 0.131647125 |
| 825 | MsG0280006462.01.T01 | MsG0180004908.01.T01 | 0.110141119 |
| 826 | MsG0280006462.01.T01 | MsG0180004930.01.T01 | 0.217329041 |
| 827 | MsG0280006462.01.T01 | MsG0180004965.01.T01 | 0.103374177 |
| 828 | MsG0280006462.01.T01 | MsG0180004996.01.T01 | 0.200557012 |
| 829 | MsG0280006462.01.T01 | MsG0180004996.01.T02 | 0.1873504 |
| 830 | MsG0280006462.01.T01 | MsG0180005135.01.T01 | 0.20930234 |
| 831 | MsG0280006462.01.T01 | MsG0180005136.01.T01 | 0.152264663 |
| 832 | MsG0280006462.01.T01 | MsG0180005168.01.T01 | 0.174662043 |
| 833 | MsG0280006462.01.T01 | MsG0180005179.01.T01 | 0.194817323 |
| 834 | MsG0280006462.01.T01 | MsG0180005228.01.T01 | 0.204030269 |
| 835 | MsG0280006462.01.T01 | MsG0180005260.01.T01 | 0.145838576 |
| 836 | MsG0280006462.01.T01 | MsG0180005264.01.T01 | 0.152841127 |
| 837 | MsG0280006462.01.T01 | MsG0180005359.01.T01 | 0.16730849 |
| 838 | MsG0280006462.01.T01 | MsG0180005395.01.T01 | 0.171478374 |
| 839 | MsG0280006462.01.T01 | MsG0180005395.01.T02 | 0.171478374 |
| 840 | MsG0280006462.01.T01 | MsG0180005395.01.T03 | 0.171478374 |
| 841 | MsG0280006462.01.T01 | MsG0180005628.01.T01 | 0.231692095 |
| 842 | MsG0280006462.01.T01 | MsG0180005632.01.T01 | 0.129486917 |
| 843 | MsG0280006462.01.T01 | MsG0180005635.01.T01 | 0.183008543 |
| 844 | MsG0280006462.01.T01 | MsG0180005637.01.T01 | 0.1682124 |
| 845 | MsG0280006462.01.T01 | MsG0180005744.01.T01 | 0.19611581 |
| 846 | MsG0280006462.01.T01 | MsG0180005779.01.T01 | 0.174526727 |
| 847 | MsG0280006462.01.T01 | MsG0180005789.01.T01 | 0.11522308 |
| 848 | MsG0280006462.01.T01 | MsG0180005867.01.T01 | 0.125390207 |
| 849 | MsG0280006462.01.T01 | MsG0180005982.01.T01 | 0.178821865 |
| 850 | MsG0280006462.01.T01 | MsG0180005982.01.T02 | 0.178821865 |
| 851 | MsG0280006462.01.T01 | MsG0180006050.01.T01 | 0.138220321 |
| 852 | MsG0280006462.01.T01 | MsG0180006050.01.T02 | 0.138220321 |
| 853 | MsG0280006462.01.T01 | MsG0180006051.01.T01 | 0.106260489 |
| 854 | MsG0280006462.01.T01 | MsG0180006053.01.T01 | 0.113815744 |
| 855 | MsG0280006462.01.T01 | MsG0180006085.01.T01 | 0.13796133 |
| 856 | MsG0280006462.01.T01 | MsG0180006099.01.T01 | 0.133828155 |
| 857 | MsG0280006462.01.T01 | MsG0180006099.01.T02 | 0.133828155 |
| 858 | MsG0280006462.01.T01 | MsG0180006137.01.T01 | 0.147989362 |
| 859 | MsG0280006462.01.T01 | MsG0180006233.01.T01 | 0.137277601 |
| 860 | MsG0280006462.01.T01 | MsG0180006252.01.T01 | 0.135094836 |
| 861 | MsG0280006462.01.T01 | MsG0180006265.01.T01 | 0.154019567 |
| 862 | MsG0280008140.01.T01 | MsG0380014677.01.T01 | 0.128508919 |
| 863 | MsG0280008140.01.T01 | MsG0380014802.01.T01 | 0.129276276 |
| 864 | MsG0280008140.01.T01 | MsG0480018388.01.T01 | 0.149094407 |
| 865 | MsG0280008140.01.T01 | MsG0480023366.01.T01 | 0.120174529 |
| 866 | MsG0280008140.01.T01 | MsG0080047999.01.T01 | 0.121176423 |
| 867 | MsG0280008140.01.T01 | MsG0180006081.01.T01 | 0.122703546 |
| 868 | MsG0280008140.01.T01 | MsG0280007439.01.T01 | 0.157636354 |
| 869 | MsG0480022802.01.T01 | MsG0480023783.01.T01 | 0.121078729 |
| 870 | MsG0480022802.01.T01 | MsG0780036845.01.T01 | 0.120611146 |
| 871 | MsG0480022802.01.T01 | MsG0780039793.01.T01 | 0.122025579 |
| 872 | MsG0480022802.01.T01 | MsG0780039822.01.T01 | 0.130013925 |
| 873 | MsG0480022802.01.T01 | MsG0780040951.01.T01 | 0.143515525 |
| 874 | MsG0480022802.01.T01 | MsG0880043916.01.T01 | 0.12297977 |
| 875 | MsG0480022802.01.T01 | MsG0880044977.01.T01 | 0.12672516 |
| 876 | MsG0480022802.01.T01 | MsG0880045368.01.T01 | 0.126733297 |
| 877 | MsG0480022802.01.T01 | MsG0880045445.01.T01 | 0.124407417 |
| 878 | MsG0480022802.01.T01 | MsG0880045534.01.T01 | 0.140621476 |
| 879 | MsG0480022802.01.T01 | MsG0880047097.01.T01 | 0.129718015 |
| 880 | MsG0480022802.01.T01 | MsG0880047210.01.T01 | 0.145228162 |
| 881 | MsG0480022802.01.T01 | MsG0080048255.01.T01 | 0.159077892 |
| 882 | MsG0480022802.01.T01 | MsG0080048312.01.T01 | 0.148870921 |
| 883 | MsG0480022802.01.T01 | MsG0080048312.01.T02 | 0.148870921 |
| 884 | MsG0480022802.01.T01 | MsG0180001906.01.T01 | 0.159310514 |
| 885 | MsG0480022802.01.T01 | MsG0180003762.01.T01 | 0.131791523 |
| 886 | MsG0480022802.01.T01 | MsG0180004883.01.T01 | 0.120376993 |
| 887 | MsG0480022802.01.T01 | MsG0180004884.01.T01 | 0.123025973 |
| 888 | MsG0480022802.01.T01 | MsG0280008645.01.T01 | 0.126393837 |
| 889 | MsG0480022802.01.T01 | MsG0280008765.01.T01 | 0.129236076 |
| 890 | MsG0480022802.01.T01 | MsG0280009552.01.T02 | 0.138870337 |
| 891 | MsG0480022802.01.T01 | MsG0280009554.01.T01 | 0.12588056 |
| 892 | MsG0480022802.01.T01 | MsG0380014653.01.T01 | 0.121907604 |
| 893 | MsG0480022802.01.T01 | MsG0380015646.01.T01 | 0.136358811 |
| 894 | MsG0480022802.01.T01 | MsG0380016514.01.T01 | 0.121641426 |
| 895 | MsG0480022802.01.T01 | MsG0480019403.01.T01 | 0.121968821 |
| 896 | MsG0480022802.01.T01 | MsG0480019403.01.T02 | 0.121968821 |
| 897 | MsG0480023156.01.T01 | MsG0480023339.01.T01 | 0.141782756 |
| 898 | MsG0480023156.01.T01 | MsG0480023340.01.T01 | 0.120904417 |
| 899 | MsG0480023156.01.T01 | MsG0480023393.01.T01 | 0.102689473 |
| 900 | MsG0480023156.01.T01 | MsG0480023528.01.T01 | 0.111198108 |
| 901 | MsG0480023156.01.T01 | MsG0480023764.01.T01 | 0.109058479 |
| 902 | MsG0480023156.01.T01 | MsG0480023798.01.T01 | 0.129973426 |
| 903 | MsG0480023156.01.T01 | MsG0480023950.01.T01 | 0.140358013 |
| 904 | MsG0480023156.01.T01 | MsG0580024077.01.T01 | 0.162587242 |
| 905 | MsG0480023156.01.T01 | MsG0580024077.01.T02 | 0.162587242 |
| 906 | MsG0480023156.01.T01 | MsG0580024077.01.T03 | 0.100090788 |
| 907 | MsG0480023156.01.T01 | MsG0580024119.01.T01 | 0.122347463 |
| 908 | MsG0480023156.01.T01 | MsG0580024460.01.T01 | 0.102500919 |
| 909 | MsG0480023156.01.T01 | MsG0580024770.01.T01 | 0.210661947 |
| 910 | MsG0480023156.01.T01 | MsG0580024890.01.T01 | 0.130729527 |
| 911 | MsG0480023156.01.T01 | MsG0580025069.01.T01 | 0.121938556 |
| 912 | MsG0480023156.01.T01 | MsG0580025196.01.T01 | 0.107830147 |
| 913 | MsG0480023156.01.T01 | MsG0580025337.01.T01 | 0.108278106 |
| 914 | MsG0480023156.01.T01 | MsG0580025354.01.T01 | 0.106360942 |
| 915 | MsG0480023156.01.T01 | MsG0580025568.01.T01 | 0.109762334 |
| 916 | MsG0480023156.01.T01 | MsG0580025649.01.T01 | 0.156633987 |
| 917 | MsG0480023156.01.T01 | MsG0580025649.01.T02 | 0.156633987 |
| 918 | MsG0480023156.01.T01 | MsG0580025652.01.T01 | 0.16556882 |
| 919 | MsG0480023156.01.T01 | MsG0580025657.01.T09 | 0.109252641 |
| 920 | MsG0480023156.01.T01 | MsG0580025657.01.T10 | 0.109252641 |
| 921 | MsG0480023156.01.T01 | MsG0580025657.01.T11 | 0.109252641 |
| 922 | MsG0480023156.01.T01 | MsG0580025657.01.T12 | 0.109252641 |
| 923 | MsG0480023156.01.T01 | MsG0580025813.01.T01 | 0.115691245 |
| 924 | MsG0480023156.01.T01 | MsG0580025906.01.T01 | 0.144589893 |
| 925 | MsG0480023156.01.T01 | MsG0580027319.01.T01 | 0.118014556 |
| 926 | MsG0480023156.01.T01 | MsG0580028033.01.T01 | 0.162431561 |
| 927 | MsG0480023156.01.T01 | MsG0580028160.01.T01 | 0.119759648 |
| 928 | MsG0480023156.01.T01 | MsG0580028480.01.T01 | 0.109992579 |
| 929 | MsG0480023156.01.T01 | MsG0580028912.01.T01 | 0.149434858 |
| 930 | MsG0480023156.01.T01 | MsG0580029491.01.T01 | 0.142144721 |
| 931 | MsG0480023156.01.T01 | MsG0580029544.01.T01 | 0.103601629 |
| 932 | MsG0480023156.01.T01 | MsG0580029544.01.T02 | 0.103601629 |
| 933 | MsG0480023156.01.T01 | MsG0580029614.01.T01 | 0.186092829 |
| 934 | MsG0480023156.01.T01 | MsG0680030560.01.T01 | 0.10422173 |
| 935 | MsG0480023156.01.T01 | MsG0680030561.01.T01 | 0.102041745 |
| 936 | MsG0480023156.01.T01 | MsG0680030619.01.T01 | 0.15092281 |
| 937 | MsG0480023156.01.T01 | MsG0680030660.01.T01 | 0.141524845 |
| 938 | MsG0480023156.01.T01 | MsG0680031638.01.T01 | 0.111780301 |
| 939 | MsG0480023156.01.T01 | MsG0680031790.01.T01 | 0.135295457 |
| 940 | MsG0480023156.01.T01 | MsG0680031816.01.T01 | 0.1783859 |
| 941 | MsG0480023156.01.T01 | MsG0680032017.01.T01 | 0.162433887 |
| 942 | MsG0480023156.01.T01 | MsG0680033744.01.T01 | 0.156861459 |
| 943 | MsG0480023156.01.T01 | MsG0680033745.01.T01 | 0.14022047 |
| 944 | MsG0480023156.01.T01 | MsG0680033911.01.T01 | 0.165623607 |
| 945 | MsG0480023156.01.T01 | MsG0680034110.01.T01 | 0.100409993 |
| 946 | MsG0480023156.01.T01 | MsG0680034132.01.T01 | 0.100520393 |
| 947 | MsG0480023156.01.T01 | MsG0680034675.01.T01 | 0.142392927 |
| 948 | MsG0480023156.01.T01 | MsG0680034677.01.T01 | 0.130940993 |
| 949 | MsG0480023156.01.T01 | MsG0680034866.01.T01 | 0.109363467 |
| 950 | MsG0480023156.01.T01 | MsG0680034884.01.T01 | 0.126223737 |
| 951 | MsG0480023156.01.T01 | MsG0680035047.01.T01 | 0.115869533 |
| 952 | MsG0480023156.01.T01 | MsG0680035240.01.T01 | 0.109312183 |
| 953 | MsG0480023156.01.T01 | MsG0680035362.01.T01 | 0.117323736 |
| 954 | MsG0480023156.01.T01 | MsG0680035909.01.T01 | 0.124320649 |
| 955 | MsG0480023156.01.T01 | MsG0780036029.01.T01 | 0.128883171 |
| 956 | MsG0480023156.01.T01 | MsG0780036090.01.T01 | 0.14082714 |
| 957 | MsG0480023156.01.T01 | MsG0780036090.01.T02 | 0.14082714 |
| 958 | MsG0480023156.01.T01 | MsG0780036263.01.T01 | 0.102084001 |
| 959 | MsG0480023156.01.T01 | MsG0780036954.01.T01 | 0.114575088 |
| 960 | MsG0480023156.01.T01 | MsG0780037364.01.T01 | 0.157894644 |
| 961 | MsG0480023156.01.T01 | MsG0780037701.01.T01 | 0.135589966 |
| 962 | MsG0480023156.01.T01 | MsG0780037895.01.T01 | 0.167513391 |
| 963 | MsG0480023156.01.T01 | MsG0780037919.01.T01 | 0.113525233 |
| 964 | MsG0480023156.01.T01 | MsG0780037920.01.T01 | 0.196492146 |
| 965 | MsG0480023156.01.T01 | MsG0780038564.01.T01 | 0.145764896 |
| 966 | MsG0480023156.01.T01 | MsG0780039144.01.T01 | 0.199619383 |
| 967 | MsG0480023156.01.T01 | MsG0780039171.01.T01 | 0.125576152 |
| 968 | MsG0480023156.01.T01 | MsG0780039250.01.T01 | 0.114271016 |
| 969 | MsG0480023156.01.T01 | MsG0780039439.01.T01 | 0.120325537 |
| 970 | MsG0480023156.01.T01 | MsG0780039783.01.T01 | 0.109656913 |
| 971 | MsG0480023156.01.T01 | MsG0780039901.01.T01 | 0.132263039 |
| 972 | MsG0480023156.01.T01 | MsG0780039901.01.T02 | 0.132263039 |
| 973 | MsG0480023156.01.T01 | MsG0780040173.01.T01 | 0.155242968 |
| 974 | MsG0480023156.01.T01 | MsG0780040173.01.T02 | 0.155242968 |
| 975 | MsG0480023156.01.T01 | MsG0780040173.01.T03 | 0.155242968 |
| 976 | MsG0480023156.01.T01 | MsG0780040442.01.T01 | 0.160069231 |
| 977 | MsG0480023156.01.T01 | MsG0780040742.01.T01 | 0.142674302 |
| 978 | MsG0480023156.01.T01 | MsG0780040826.01.T01 | 0.164958712 |
| 979 | MsG0480023156.01.T01 | MsG0780040827.01.T01 | 0.182812609 |
| 980 | MsG0480023156.01.T01 | MsG0780040904.01.T02 | 0.152549557 |
| 981 | MsG0480023156.01.T01 | MsG0780041019.01.T01 | 0.120087584 |
| 982 | MsG0480023156.01.T01 | MsG0780041033.01.T01 | 0.204606931 |
| 983 | MsG0480023156.01.T01 | MsG0780041276.01.T01 | 0.119355576 |
| 984 | MsG0480023156.01.T01 | MsG0780041276.01.T02 | 0.119355576 |
| 985 | MsG0480023156.01.T01 | MsG0780041276.01.T03 | 0.119355576 |
| 986 | MsG0480023156.01.T01 | MsG0780041276.01.T04 | 0.149206932 |
| 987 | MsG0480023156.01.T01 | MsG0780041276.01.T05 | 0.149206932 |
| 988 | MsG0480023156.01.T01 | MsG0780041276.01.T06 | 0.149206932 |
| 989 | MsG0480023156.01.T01 | MsG0780041330.01.T01 | 0.16894173 |
| 990 | MsG0480023156.01.T01 | MsG0780041594.01.T01 | 0.120290341 |
| 991 | MsG0480023156.01.T01 | MsG0780041613.01.T01 | 0.142002621 |
| 992 | MsG0480023156.01.T01 | MsG0880041901.01.T01 | 0.12744074 |
| 993 | MsG0480023156.01.T01 | MsG0880042271.01.T01 | 0.126476727 |
| 994 | MsG0480023156.01.T01 | MsG0880042370.01.T01 | 0.11111917 |
| 995 | MsG0480023156.01.T01 | MsG0880042421.01.T01 | 0.107700247 |
| 996 | MsG0480023156.01.T01 | MsG0880042825.01.T01 | 0.122321465 |
| 997 | MsG0480023156.01.T01 | MsG0880042880.01.T01 | 0.137893575 |
| 998 | MsG0480023156.01.T01 | MsG0880043003.01.T01 | 0.114725908 |
| 999 | MsG0480023156.01.T01 | MsG0880043119.01.T01 | 0.124843129 |
| 1000 | MsG0480023156.01.T01 | MsG0880043457.01.T01 | 0.116385072 |
| 1001 | MsG0480023156.01.T01 | MsG0880043510.01.T01 | 0.131919204 |
| 1002 | MsG0480023156.01.T01 | MsG0880044439.01.T01 | 0.150096323 |
| 1003 | MsG0480023156.01.T01 | MsG0880044763.01.T01 | 0.127353912 |
| 1004 | MsG0480023156.01.T01 | MsG0880045006.01.T01 | 0.110249056 |
| 1005 | MsG0480023156.01.T01 | MsG0880045006.01.T02 | 0.110249056 |
| 1006 | MsG0480023156.01.T01 | MsG0880045118.01.T01 | 0.120900822 |
| 1007 | MsG0480023156.01.T01 | MsG0880045146.01.T01 | 0.115712105 |
| 1008 | MsG0480023156.01.T01 | MsG0880045244.01.T01 | 0.15711328 |
| 1009 | MsG0480023156.01.T01 | MsG0880045291.01.T01 | 0.116638788 |
| 1010 | MsG0480023156.01.T01 | MsG0880045382.01.T01 | 0.126108423 |
| 1011 | MsG0480023156.01.T01 | MsG0880045551.01.T01 | 0.157404491 |
| 1012 | MsG0480023156.01.T01 | MsG0880045818.01.T01 | 0.14194069 |
| 1013 | MsG0480023156.01.T01 | MsG0880046077.01.T01 | 0.175657957 |
| 1014 | MsG0480023156.01.T01 | MsG0880046135.01.T01 | 0.160119001 |
| 1015 | MsG0480023156.01.T01 | MsG0880046152.01.T01 | 0.115827214 |
| 1016 | MsG0480023156.01.T01 | MsG0880046258.01.T01 | 0.146795178 |
| 1017 | MsG0480023156.01.T01 | MsG0880046606.01.T01 | 0.100663998 |
| 1018 | MsG0480023156.01.T01 | MsG0880046864.01.T01 | 0.110904542 |
| 1019 | MsG0480023156.01.T01 | MsG0880047064.01.T01 | 0.132585009 |
| 1020 | MsG0480023156.01.T01 | MsG0880047075.01.T01 | 0.120951065 |
| 1021 | MsG0480023156.01.T01 | MsG0880047148.01.T01 | 0.179146236 |
| 1022 | MsG0480023156.01.T01 | MsG0880047296.01.T01 | 0.135403862 |
| 1023 | MsG0480023156.01.T01 | MsG0880047313.01.T01 | 0.118838613 |
| 1024 | MsG0480023156.01.T01 | MsG0080047950.01.T01 | 0.111612455 |
| 1025 | MsG0480023156.01.T01 | MsG0080047967.01.T01 | 0.105794125 |
| 1026 | MsG0480023156.01.T01 | MsG0080047991.01.T01 | 0.118716797 |
| 1027 | MsG0480023156.01.T01 | MsG0080047996.01.T01 | 0.177056885 |
| 1028 | MsG0480023156.01.T01 | MsG0080048703.01.T01 | 0.197595368 |
| 1029 | MsG0480023156.01.T01 | MsG0080048885.01.T01 | 0.103833631 |
| 1030 | MsG0480023156.01.T01 | MsG0080048886.01.T01 | 0.126341533 |
| 1031 | MsG0480023156.01.T01 | MsG0180000002.01.T01 | 0.118472168 |
| 1032 | MsG0480023156.01.T01 | MsG0180000461.01.T01 | 0.100772047 |
| 1033 | MsG0480023156.01.T01 | MsG0180000594.01.T01 | 0.162125373 |
| 1034 | MsG0480023156.01.T01 | MsG0180000596.01.T01 | 0.105486972 |
| 1035 | MsG0480023156.01.T01 | MsG0180000663.01.T01 | 0.135942762 |
| 1036 | MsG0480023156.01.T01 | MsG0180000681.01.T01 | 0.119117656 |
| 1037 | MsG0480023156.01.T01 | MsG0180000681.01.T02 | 0.119117656 |
| 1038 | MsG0480023156.01.T01 | MsG0180000681.01.T03 | 0.119117656 |
| 1039 | MsG0480023156.01.T01 | MsG0180000688.01.T01 | 0.108929284 |
| 1040 | MsG0480023156.01.T01 | MsG0180000688.01.T02 | 0.108929284 |
| 1041 | MsG0480023156.01.T01 | MsG0180000693.01.T01 | 0.108436443 |
| 1042 | MsG0480023156.01.T01 | MsG0180000694.01.T01 | 0.138031785 |
| 1043 | MsG0480023156.01.T01 | MsG0180001721.01.T01 | 0.123963435 |
| 1044 | MsG0480023156.01.T01 | MsG0180001816.01.T01 | 0.200718396 |
| 1045 | MsG0480023156.01.T01 | MsG0180002451.01.T01 | 0.148151147 |
| 1046 | MsG0480023156.01.T01 | MsG0180002911.01.T01 | 0.16951069 |
| 1047 | MsG0480023156.01.T01 | MsG0180002976.01.T01 | 0.157034119 |
| 1048 | MsG0480023156.01.T01 | MsG0180003129.01.T01 | 0.100863545 |
| 1049 | MsG0480023156.01.T01 | MsG0180003386.01.T01 | 0.103736718 |
| 1050 | MsG0480023156.01.T01 | MsG0180003464.01.T01 | 0.108149745 |
| 1051 | MsG0480023156.01.T01 | MsG0180003620.01.T02 | 0.145478054 |
| 1052 | MsG0480023156.01.T01 | MsG0180003674.01.T04 | 0.102385333 |
| 1053 | MsG0480023156.01.T01 | MsG0180003767.01.T01 | 0.17350367 |
| 1054 | MsG0480023156.01.T01 | MsG0180003862.01.T01 | 0.140395101 |
| 1055 | MsG0480023156.01.T01 | MsG0180003864.01.T01 | 0.116053491 |
| 1056 | MsG0480023156.01.T01 | MsG0180003940.01.T01 | 0.117785667 |
| 1057 | MsG0480023156.01.T01 | MsG0180003940.01.T02 | 0.117785667 |
| 1058 | MsG0480023156.01.T01 | MsG0180003950.01.T01 | 0.142926796 |
| 1059 | MsG0480023156.01.T01 | MsG0180004002.01.T01 | 0.202999088 |
| 1060 | MsG0480023156.01.T01 | MsG0180004055.01.T01 | 0.180337195 |
| 1061 | MsG0480023156.01.T01 | MsG0180004349.01.T01 | 0.102996993 |
| 1062 | MsG0480023156.01.T01 | MsG0180004372.01.T01 | 0.128657603 |
| 1063 | MsG0480023156.01.T01 | MsG0180004525.01.T01 | 0.15808566 |
| 1064 | MsG0480023156.01.T01 | MsG0180004833.01.T01 | 0.12153166 |
| 1065 | MsG0480023156.01.T01 | MsG0180004874.01.T01 | 0.149345387 |
| 1066 | MsG0480023156.01.T01 | MsG0180004921.01.T01 | 0.106326063 |
| 1067 | MsG0480023156.01.T01 | MsG0180004996.01.T01 | 0.116012816 |
| 1068 | MsG0480023156.01.T01 | MsG0180004996.01.T02 | 0.130322823 |
| 1069 | MsG0480023156.01.T01 | MsG0180005179.01.T01 | 0.138911151 |
| 1070 | MsG0480023156.01.T01 | MsG0180005228.01.T01 | 0.100360392 |
| 1071 | MsG0480023156.01.T01 | MsG0180005395.01.T01 | 0.140734716 |
| 1072 | MsG0480023156.01.T01 | MsG0180005395.01.T02 | 0.140734716 |
| 1073 | MsG0480023156.01.T01 | MsG0180005395.01.T03 | 0.140734716 |
| 1074 | MsG0480023156.01.T01 | MsG0180005637.01.T01 | 0.126555986 |
| 1075 | MsG0480023156.01.T01 | MsG0180005867.01.T01 | 0.174880478 |
| 1076 | MsG0480023156.01.T01 | MsG0180006085.01.T01 | 0.185315492 |
| 1077 | MsG0480023156.01.T01 | MsG0180006099.01.T01 | 0.126464577 |
| 1078 | MsG0480023156.01.T01 | MsG0180006099.01.T02 | 0.126464577 |
| 1079 | MsG0480023156.01.T01 | MsG0280006803.01.T01 | 0.113217005 |
| 1080 | MsG0480023156.01.T01 | MsG0280007073.01.T01 | 0.122141956 |
| 1081 | MsG0480023156.01.T01 | MsG0280007713.01.T01 | 0.171116217 |
| 1082 | MsG0480023156.01.T01 | MsG0280007872.01.T01 | 0.162055278 |
| 1083 | MsG0480023156.01.T01 | MsG0280007937.01.T01 | 0.12722265 |
| 1084 | MsG0480023156.01.T01 | MsG0280008222.01.T01 | 0.128659487 |
| 1085 | MsG0480023156.01.T01 | MsG0280008417.01.T01 | 0.164721093 |
| 1086 | MsG0480023156.01.T01 | MsG0280008750.01.T01 | 0.132255419 |
| 1087 | MsG0480023156.01.T01 | MsG0280008887.01.T01 | 0.108581708 |
| 1088 | MsG0480023156.01.T01 | MsG0280009342.01.T01 | 0.105342847 |
| 1089 | MsG0480023156.01.T01 | MsG0280009352.01.T02 | 0.11677766 |
| 1090 | MsG0480023156.01.T01 | MsG0280009448.01.T01 | 0.103177497 |
| 1091 | MsG0480023156.01.T01 | MsG0280009868.01.T01 | 0.103375489 |
| 1092 | MsG0480023156.01.T01 | MsG0280010146.01.T01 | 0.109398884 |
| 1093 | MsG0480023156.01.T01 | MsG0280010244.01.T01 | 0.105806286 |
| 1094 | MsG0480023156.01.T01 | MsG0280010244.01.T02 | 0.105806286 |
| 1095 | MsG0480023156.01.T01 | MsG0280010361.01.T01 | 0.156055231 |
| 1096 | MsG0480023156.01.T01 | MsG0280010450.01.T01 | 0.157446069 |
| 1097 | MsG0480023156.01.T01 | MsG0280010450.01.T02 | 0.157446069 |
| 1098 | MsG0480023156.01.T01 | MsG0280010450.01.T03 | 0.157446069 |
| 1099 | MsG0480023156.01.T01 | MsG0280010476.01.T01 | 0.124564789 |
| 1100 | MsG0480023156.01.T01 | MsG0280010476.01.T02 | 0.124564789 |
| 1101 | MsG0480023156.01.T01 | MsG0280010476.01.T03 | 0.124564789 |
| 1102 | MsG0480023156.01.T01 | MsG0280010531.01.T01 | 0.188067489 |
| 1103 | MsG0480023156.01.T01 | MsG0280010540.01.T01 | 0.123074071 |
| 1104 | MsG0480023156.01.T01 | MsG0280010554.01.T01 | 0.165251881 |
| 1105 | MsG0480023156.01.T01 | MsG0280010862.01.T01 | 0.217925205 |
| 1106 | MsG0480023156.01.T01 | MsG0280011117.01.T01 | 0.196838019 |
| 1107 | MsG0480023156.01.T01 | MsG0280011165.01.T01 | 0.139945664 |
| 1108 | MsG0480023156.01.T01 | MsG0280011203.01.T01 | 0.130669742 |
| 1109 | MsG0480023156.01.T01 | MsG0280011236.01.T01 | 0.102509695 |
| 1110 | MsG0480023156.01.T01 | MsG0280011266.01.T01 | 0.141619738 |
| 1111 | MsG0480023156.01.T01 | MsG0280011440.01.T01 | 0.110510948 |
| 1112 | MsG0480023156.01.T01 | MsG0380011706.01.T01 | 0.153804745 |
| 1113 | MsG0480023156.01.T01 | MsG0380011706.01.T02 | 0.153804745 |
| 1114 | MsG0480023156.01.T01 | MsG0380011840.01.T01 | 0.109279894 |
| 1115 | MsG0480023156.01.T01 | MsG0380011840.01.T02 | 0.109279894 |
| 1116 | MsG0480023156.01.T01 | MsG0380012124.01.T01 | 0.165131326 |
| 1117 | MsG0480023156.01.T01 | MsG0380012221.01.T01 | 0.178316569 |
| 1118 | MsG0480023156.01.T01 | MsG0380013071.01.T01 | 0.106028969 |
| 1119 | MsG0480023156.01.T01 | MsG0380013644.01.T01 | 0.136536325 |
| 1120 | MsG0480023156.01.T01 | MsG0380014182.01.T01 | 0.15995205 |
| 1121 | MsG0480023156.01.T01 | MsG0380014357.01.T01 | 0.124548141 |
| 1122 | MsG0480023156.01.T01 | MsG0380014600.01.T01 | 0.146834465 |
| 1123 | MsG0480023156.01.T01 | MsG0380014934.01.T01 | 0.12742877 |
| 1124 | MsG0480023156.01.T01 | MsG0380015162.01.T01 | 0.112402209 |
| 1125 | MsG0480023156.01.T01 | MsG0380015787.01.T01 | 0.18855966 |
| 1126 | MsG0480023156.01.T01 | MsG0380016025.01.T01 | 0.100157974 |
| 1127 | MsG0480023156.01.T01 | MsG0380016072.01.T01 | 0.176878346 |
| 1128 | MsG0480023156.01.T01 | MsG0380016080.01.T01 | 0.117263298 |
| 1129 | MsG0480023156.01.T01 | MsG0380016080.01.T02 | 0.117263298 |
| 1130 | MsG0480023156.01.T01 | MsG0380016290.01.T01 | 0.10799757 |
| 1131 | MsG0480023156.01.T01 | MsG0380016397.01.T01 | 0.111235838 |
| 1132 | MsG0480023156.01.T01 | MsG0380016661.01.T01 | 0.179984402 |
| 1133 | MsG0480023156.01.T01 | MsG0380016843.01.T01 | 0.144802206 |
| 1134 | MsG0480023156.01.T01 | MsG0380016877.01.T01 | 0.148438141 |
| 1135 | MsG0480023156.01.T01 | MsG0380016877.01.T02 | 0.148438141 |
| 1136 | MsG0480023156.01.T01 | MsG0380016878.01.T01 | 0.173316642 |
| 1137 | MsG0480023156.01.T01 | MsG0380016937.01.T01 | 0.108317555 |
| 1138 | MsG0480023156.01.T01 | MsG0380016997.01.T01 | 0.164833487 |
| 1139 | MsG0480023156.01.T01 | MsG0380016997.01.T02 | 0.164833487 |
| 1140 | MsG0480023156.01.T01 | MsG0380016997.01.T03 | 0.164833487 |
| 1141 | MsG0480023156.01.T01 | MsG0380017092.01.T02 | 0.24591926 |
| 1142 | MsG0480023156.01.T01 | MsG0380017281.01.T01 | 0.102834263 |
| 1143 | MsG0480023156.01.T01 | MsG0380017528.01.T01 | 0.158287204 |
| 1144 | MsG0480023156.01.T01 | MsG0380017641.01.T01 | 0.159937195 |
| 1145 | MsG0480023156.01.T01 | MsG0380017764.01.T01 | 0.196225985 |
| 1146 | MsG0480023156.01.T01 | MsG0380017768.01.T01 | 0.180229868 |
| 1147 | MsG0480023156.01.T01 | MsG0480018086.01.T02 | 0.109898245 |
| 1148 | MsG0480023156.01.T01 | MsG0480018116.01.T01 | 0.124368565 |
| 1149 | MsG0480023156.01.T01 | MsG0480018145.01.T01 | 0.239468365 |
| 1150 | MsG0480023156.01.T01 | MsG0480018318.01.T01 | 0.115723097 |
| 1151 | MsG0480023156.01.T01 | MsG0480018350.01.T01 | 0.126046422 |
| 1152 | MsG0480023156.01.T01 | MsG0480019896.01.T01 | 0.112365293 |
| 1153 | MsG0480023156.01.T01 | MsG0480020129.01.T01 | 0.20407296 |
| 1154 | MsG0480023156.01.T01 | MsG0480020472.01.T01 | 0.115083317 |
| 1155 | MsG0480023156.01.T01 | MsG0480020827.01.T01 | 0.11019148 |
| 1156 | MsG0480023156.01.T01 | MsG0480020884.01.T01 | 0.117267163 |
| 1157 | MsG0480023156.01.T01 | MsG0480021134.01.T01 | 0.190709361 |
| 1158 | MsG0480023156.01.T01 | MsG0480021245.01.T01 | 0.109879357 |
| 1159 | MsG0480023156.01.T01 | MsG0480021390.01.T01 | 0.140556319 |
| 1160 | MsG0480023156.01.T01 | MsG0480021407.01.T01 | 0.125925611 |
| 1161 | MsG0480023156.01.T01 | MsG0480021708.01.T01 | 0.189016345 |
| 1162 | MsG0480023156.01.T01 | MsG0480021876.01.T01 | 0.11835312 |
| 1163 | MsG0480023156.01.T01 | MsG0480021940.01.T01 | 0.145871291 |
| 1164 | MsG0480023156.01.T01 | MsG0480021941.01.T01 | 0.151178503 |
| 1165 | MsG0480023156.01.T01 | MsG0480022032.01.T01 | 0.116067235 |
| 1166 | MsG0480023156.01.T01 | MsG0480022258.01.T01 | 0.118884209 |
| 1167 | MsG0480023156.01.T01 | MsG0480022258.01.T02 | 0.118884209 |
| 1168 | MsG0480023156.01.T01 | MsG0480022259.01.T07 | 0.100934687 |
| 1169 | MsG0480023156.01.T01 | MsG0480022381.01.T01 | 0.100328229 |
| 1170 | MsG0480023156.01.T01 | MsG0480022410.01.T01 | 0.117953159 |
| 1171 | MsG0480023156.01.T01 | MsG0480022436.01.T01 | 0.110010464 |
| 1172 | MsG0480023156.01.T01 | MsG0480022469.01.T01 | 0.135326536 |
| 1173 | MsG0480023156.01.T01 | MsG0480022552.01.T01 | 0.102250275 |
| 1174 | MsG0480023156.01.T01 | MsG0480022611.01.T01 | 0.107608325 |
| 1175 | MsG0480023156.01.T01 | MsG0480022939.01.T01 | 0.115658435 |
| 1176 | MsG0480023156.01.T01 | MsG0480022953.01.T01 | 0.127173811 |
| 1177 | MsG0480023156.01.T01 | MsG0480022953.01.T02 | 0.127173811 |
| 1178 | MsG0480023156.01.T01 | MsG0480023024.01.T01 | 0.151719975 |
| 1179 | MsG0580025786.01.T01 | MsG0580025819.01.T01 | 0.151458033 |
| 1180 | MsG0580025786.01.T01 | MsG0380014743.01.T01 | 0.177124598 |
| 1181 | MsG0580025786.01.T01 | MsG0480018433.01.T01 | 0.157554835 |
| 1182 | MsG0580028415.01.T01 | MsG0180003638.01.T01 | 0.103345044 |
| 1183 | MsG0580028415.01.T01 | MsG0480023781.01.T01 | 0.116495223 |
| 1184 | MsG0580025786.01.T01 | MsG0180000321.01.T01 | 0.126500035 |
| 1185 | MsG0580025786.01.T01 | MsG0180000895.01.T05 | 0.126214665 |
| 1186 | MsG0580025786.01.T01 | MsG0180000896.01.T03 | 0.132330698 |
| 1187 | MsG0580025786.01.T01 | MsG0180000920.01.T01 | 0.120230127 |
| 1188 | MsG0580025786.01.T01 | MsG0180005006.01.T01 | 0.133605371 |
| 1189 | MsG0580025786.01.T01 | MsG0280006950.01.T01 | 0.120189778 |
| 1190 | MsG0580025786.01.T01 | MsG0280007204.01.T01 | 0.142614819 |
| 1191 | MsG0580025786.01.T01 | MsG0280008554.01.T01 | 0.12677552 |
| 1192 | MsG0580025786.01.T01 | MsG0380014168.01.T01 | 0.127771497 |
| 1193 | MsG0580025786.01.T01 | MsG0380014743.01.T01 | 0.177124598 |
| 1194 | MsG0580025786.01.T01 | MsG0380016120.01.T01 | 0.133149667 |
| 1195 | MsG0580025786.01.T01 | MsG0380016197.01.T01 | 0.130912187 |
| 1196 | MsG0580025786.01.T01 | MsG0380016227.01.T01 | 0.127100429 |
| 1197 | MsG0580025786.01.T01 | MsG0380016925.01.T01 | 0.13027258 |
| 1198 | MsG0580025786.01.T01 | MsG0380017083.01.T01 | 0.121021038 |
| 1199 | MsG0580025786.01.T01 | MsG0380017394.01.T01 | 0.139708008 |
| 1200 | MsG0580025786.01.T01 | MsG0380017480.01.T01 | 0.121848022 |
| 1201 | MsG0580025786.01.T01 | MsG0380017898.01.T01 | 0.127177653 |
| 1202 | MsG0580025786.01.T01 | MsG0480018092.01.T01 | 0.122106775 |
| 1203 | MsG0580025786.01.T01 | MsG0480018093.01.T01 | 0.131636358 |
| 1204 | MsG0580025786.01.T01 | MsG0480018345.01.T01 | 0.122225138 |
| 1205 | MsG0580025786.01.T01 | MsG0480018433.01.T01 | 0.157554835 |
| 1206 | MsG0580025786.01.T01 | MsG0480018562.01.T01 | 0.14031446 |
| 1207 | MsG0580025786.01.T01 | MsG0480019305.01.T01 | 0.136862656 |
| 1208 | MsG0580025786.01.T01 | MsG0480020541.01.T01 | 0.121869654 |
| 1209 | MsG0580025786.01.T01 | MsG0480020784.01.T01 | 0.147284385 |
| 1210 | MsG0580025786.01.T01 | MsG0480020853.01.T01 | 0.129682351 |
| 1211 | MsG0580025786.01.T01 | MsG0480021319.01.T01 | 0.144861429 |
| 1212 | MsG0580025786.01.T01 | MsG0480022400.01.T01 | 0.129989098 |
| 1213 | MsG0580025786.01.T01 | MsG0480023326.01.T01 | 0.122405819 |
| 1214 | MsG0580025786.01.T01 | MsG0480023898.01.T01 | 0.147321006 |
| 1215 | MsG0580025786.01.T01 | MsG0580024576.01.T01 | 0.124841684 |
| 1216 | MsG0580025786.01.T01 | MsG0580024590.01.T01 | 0.120617413 |
| 1217 | MsG0580025786.01.T01 | MsG0580024776.01.T01 | 0.127483808 |
| 1218 | MsG0580025786.01.T01 | MsG0580025000.01.T01 | 0.131860186 |
| 1219 | MsG0580025786.01.T01 | MsG0580025819.01.T01 | 0.151458033 |
| 1220 | MsG0580028415.01.T01 | MsG0580029028.01.T01 | 0.123172618 |
| 1221 | MsG0580028415.01.T01 | MsG0580029383.01.T01 | 0.140707657 |
| 1222 | MsG0580028415.01.T01 | MsG0580029639.01.T01 | 0.129788428 |
| 1223 | MsG0580028415.01.T01 | MsG0580029990.01.T01 | 0.164679611 |
| 1224 | MsG0580028415.01.T01 | MsG0680030361.01.T01 | 0.127133096 |
| 1225 | MsG0580028415.01.T01 | MsG0680030468.01.T01 | 0.13488537 |
| 1226 | MsG0580028415.01.T01 | MsG0680030469.01.T01 | 0.128686368 |
| 1227 | MsG0580028415.01.T01 | MsG0680031363.01.T01 | 0.13441334 |
| 1228 | MsG0580028415.01.T01 | MsG0680031389.01.T01 | 0.124108134 |
| 1229 | MsG0580028415.01.T01 | MsG0680031637.01.T01 | 0.121047253 |
| 1230 | MsG0580028415.01.T01 | MsG0680031790.01.T01 | 0.123519818 |
| 1231 | MsG0580028415.01.T01 | MsG0680031829.01.T01 | 0.123283791 |
| 1232 | MsG0580028415.01.T01 | MsG0680031926.01.T01 | 0.139620113 |
| 1233 | MsG0580028415.01.T01 | MsG0680031977.01.T01 | 0.123777817 |
| 1234 | MsG0580028415.01.T01 | MsG0680032165.01.T09 | 0.128532854 |
| 1235 | MsG0580028415.01.T01 | MsG0680032814.01.T01 | 0.123860438 |
| 1236 | MsG0580028415.01.T01 | MsG0680033302.01.T01 | 0.147081644 |
| 1237 | MsG0580028415.01.T01 | MsG0680033616.01.T01 | 0.126634809 |
| 1238 | MsG0580028415.01.T01 | MsG0680034132.01.T01 | 0.121587875 |
| 1239 | MsG0580028415.01.T01 | MsG0680034320.01.T01 | 0.160465393 |
| 1240 | MsG0580028415.01.T01 | MsG0680035322.01.T01 | 0.131946829 |
| 1241 | MsG0580028415.01.T01 | MsG0680035325.01.T01 | 0.128531945 |
| 1242 | MsG0580028415.01.T01 | MsG0680035528.01.T01 | 0.150327804 |
| 1243 | MsG0580028415.01.T01 | MsG0680035696.01.T01 | 0.124826108 |
| 1244 | MsG0580028415.01.T01 | MsG0680035784.01.T01 | 0.125213443 |
| 1245 | MsG0580028415.01.T01 | MsG0680035909.01.T01 | 0.126224679 |
| 1246 | MsG0580028415.01.T01 | MsG0780036017.01.T01 | 0.132261595 |
| 1247 | MsG0580028415.01.T01 | MsG0780036239.01.T01 | 0.128802213 |
| 1248 | MsG0580028415.01.T01 | MsG0780036309.01.T01 | 0.125226933 |
| 1249 | MsG0580028415.01.T01 | MsG0780036925.01.T01 | 0.121103353 |
| 1250 | MsG0580028415.01.T01 | MsG0780036925.01.T02 | 0.121103353 |
| 1251 | MsG0580028415.01.T01 | MsG0780037189.01.T01 | 0.130711157 |
| 1252 | MsG0580028415.01.T01 | MsG0780037442.01.T01 | 0.141624173 |
| 1253 | MsG0580028415.01.T01 | MsG0780037469.01.T04 | 0.135050794 |
| 1254 | MsG0580028415.01.T01 | MsG0780038389.01.T01 | 0.127225048 |
| 1255 | MsG0580028415.01.T01 | MsG0780038570.01.T01 | 0.120947791 |
| 1256 | MsG0580028415.01.T01 | MsG0780039144.01.T01 | 0.120335167 |
| 1257 | MsG0580028415.01.T01 | MsG0780039861.01.T02 | 0.158509972 |
| 1258 | MsG0580028415.01.T01 | MsG0780039863.01.T01 | 0.145117667 |
| 1259 | MsG0580028415.01.T01 | MsG0780040239.01.T01 | 0.1243092 |
| 1260 | MsG0580028415.01.T01 | MsG0780040806.01.T01 | 0.125253946 |
| 1261 | MsG0580028415.01.T01 | MsG0780041078.01.T01 | 0.160667044 |
| 1262 | MsG0580028415.01.T01 | MsG0780041078.01.T02 | 0.159838795 |
| 1263 | MsG0580028415.01.T01 | MsG0780041078.01.T03 | 0.169169065 |
| 1264 | MsG0580028415.01.T01 | MsG0780041241.01.T01 | 0.125930129 |
| 1265 | MsG0580028415.01.T01 | MsG0780041277.01.T01 | 0.136168028 |
| 1266 | MsG0580028415.01.T01 | MsG0780041509.01.T01 | 0.129300212 |
| 1267 | MsG0580028415.01.T01 | MsG0780041534.01.T01 | 0.139511124 |
| 1268 | MsG0580028415.01.T01 | MsG0780041534.01.T02 | 0.139511124 |
| 1269 | MsG0580028415.01.T01 | MsG0780041696.01.T01 | 0.128103233 |
| 1270 | MsG0580028415.01.T01 | MsG0780041696.01.T02 | 0.128103233 |
| 1271 | MsG0580028415.01.T01 | MsG0780041696.01.T04 | 0.128103233 |
| 1272 | MsG0580028415.01.T01 | MsG0880041901.01.T01 | 0.138643853 |
| 1273 | MsG0580028415.01.T01 | MsG0880042370.01.T01 | 0.127584317 |
| 1274 | MsG0580028415.01.T01 | MsG0880042523.01.T01 | 0.127015687 |
| 1275 | MsG0580028415.01.T01 | MsG0880042825.01.T01 | 0.134205138 |
| 1276 | MsG0580028415.01.T01 | MsG0880042833.01.T01 | 0.153042512 |
| 1277 | MsG0580028415.01.T01 | MsG0880043145.01.T01 | 0.140292456 |
| 1278 | MsG0580028415.01.T01 | MsG0880043465.01.T01 | 0.124363216 |
| 1279 | MsG0580028415.01.T01 | MsG0880043511.01.T01 | 0.13691665 |
| 1280 | MsG0580028415.01.T01 | MsG0880043653.01.T01 | 0.120992411 |
| 1281 | MsG0580028415.01.T01 | MsG0880044946.01.T01 | 0.130437955 |
| 1282 | MsG0580028415.01.T01 | MsG0880045894.01.T01 | 0.121937403 |
| 1283 | MsG0580028415.01.T01 | MsG0880046298.01.T01 | 0.159695901 |
| 1284 | MsG0580028415.01.T01 | MsG0880046544.01.T01 | 0.120151919 |
| 1285 | MsG0580028415.01.T01 | MsG0880046559.01.T01 | 0.125031848 |
| 1286 | MsG0580028415.01.T01 | MsG0880046671.01.T01 | 0.147113814 |
| 1287 | MsG0580028415.01.T01 | MsG0880046849.01.T01 | 0.120102449 |
| 1288 | MsG0580028415.01.T01 | MsG0880047136.01.T01 | 0.160231228 |
| 1289 | MsG0580028415.01.T01 | MsG0880047323.01.T01 | 0.164746347 |
| 1290 | MsG0580028415.01.T01 | MsG0880047323.01.T02 | 0.164746347 |
| 1291 | MsG0580028415.01.T01 | MsG0880047323.01.T03 | 0.139150201 |
| 1292 | MsG0580028415.01.T01 | MsG0880047323.01.T04 | 0.147301517 |
| 1293 | MsG0580028415.01.T01 | MsG0880047672.01.T01 | 0.187094712 |
